# Revision of the Neotropical bark mantis genus *Liturgusa* Saussure, 1869 (Insecta, Mantodea, Liturgusini)

**DOI:** 10.3897/zookeys.390.6661

**Published:** 2014-03-18

**Authors:** Gavin J. Svenson

**Affiliations:** 1 Department of Invertebrate Zoology, Cleveland Museum of Natural History, 1 Wade Oval Drive, Cleveland, Ohio 44106

**Keywords:** Mantodea, praying mantis, *Liturgusa*, Neotropical, taxonomy, new species

## Abstract

The praying mantis genus *Liturgusa* Saussure, 1869 occurs only in Central and South America and represents the most diverse genus of Neotropical Liturgusini (Ehrmann 2002). The genus includes bark dwelling species, which live entirely on the trunks and branches of trees and run extremely fast. All species included within the genus *Liturgusa* are comprehensively revised with a distribution stretching from central Mexico, the island of Dominica to the southeastern regions of Brazil and southern Bolivia. All known species are redescribed to meet the standards of the new treatment of the genus (11 species). Three new genera are described including *Fuga* **gen. n.**, *Velox* **gen. n.**, and *Corticomantis* **gen. n.** for species previously included in *Liturgusa* as well as *Hagiomantis*. *Liturgusa mesopoda* Westwood, 1889 is moved to within the previously described genus *Hagiomantis* Audinet Serville, 1838. A total of 19 species are newly described within *Liturgusa*, *Fuga*, and *Velox* including *L. algorei* **sp. n.**, *L. bororum* **sp. n.**, *L. cameroni* **sp. n.**, *L. cura* **sp. n.**, *L. dominica* **sp. n.**, *L. fossetti* **sp. n.**, *L. kirtlandi* **sp. n.**, *L. krattorum* **sp. n.**, *L. manausensis* **sp. n.**, *L. maroni* **sp. n.**, *L. milleri* **sp. n.**, *L. neblina* **sp. n.**, *L. purus* **sp. n.**, *L. stiewei* **sp. n.**, *L. tessae* **sp. n.**, *L. trinidadensis* **sp. n.**, *L. zoae* **sp. n.**, *F. grimaldii* **sp. n.**, and *V. wielandi* **sp. n.** Four species names are synonymized: *Liturgusa peruviana* Giglio-Tos, 1914, **syn. n.** = *Liturgusa nubeculosa* Gerstaecker, 1889 and *Hagiomantis parva* Piza, 1966, **syn. n.**, *Liturgusa sinvalnetoi* Piza, 1982, **syn. n.**, and *Liturgusa parva* Giglio-Tos, 1914, **syn. n.** = *Mantis annulipes* Audinet Serville, 1838. Lectotypes are designated for the following two species: *Liturgusa maya* Saussure & Zehntner, 1894 and *Fuga annulipes* (Audinet Serville, 1838). A male neotype is designated for *Liturgusa guyanensis* La Greca, 1939. Males for eight species are described for the first time including *Liturgusa cayennensis* Saussure, 1869, *Liturgusa lichenalis* Gerstaecker, 1889, *Liturgusa guyanensis* La Greca, 1939, *Liturgusa maya* Saussure & Zehntner, 1894, *Liturgusa nubeculosa* Gerstaecker, 1889, *Fuga annulipes* (Audinet Serville, 1838), *Corticomantis atricoxata* (Beier, 1931), and *Hagiomantis mesopoda* (Westwood, 1889). The female of *Fuga fluminensis* (Piza, 1965) is described for the first time. Complete bibliographic histories are provided for previously described species. The spelling confusion surrounding *Liturgusa/Liturgousa* is resolved. Full habitus images for males and females are provided for nearly all species. Habitus and label images of type specimens are provided when possible. Diagnostic illustrations of the head and pronotum for males and females are provided for all species when possible. Illustrations of male genital structures are provided for all species for which males are known. Measurement data, including ranges and averages, are provided for males and females of all species. Combined male and female genus and species level dichotomous keys are provided with a Spanish translation. A complete table of all examined specimens lists label data, museum codes, repositories, and other specimen specific information. A KML file with all georeferenced locality records is downloadable from mantodearesearch.com for viewing in Google Earth. Natural history information is provided for species observed by the author.

## Introduction

The family Liturgusidae (*sensu* Ehrmann 2002) includes a broad assemblage of genera distributed on five continents, all members being characterized as ecomorphic specialists on tree trunks or branches. Informally called “bark mantises”, the group exhibits: heavy camouflage mottling that includes browns, black, and other earth tone colors matching tree bark or lichen substrates; dorsoventral flattening for a lower profile against a flat surface; and a ventral prothoracic femoral pit to accommodate for the distal posteroventral prothoracic tibial spine (homoplasious with other Mantodea, see Wieland 2013). However, the family, which appears to be based on a strong ecomorphic grouping, was found to be polyphyletic by Svenson and Whiting (2009) who used molecular data to reconstruct the phylogeny. They found that bark mantis genera group more closely to other, morphologically dissimilar taxa distributed within the same geographic region. This led to the conclusion that higher-level classification of Mantodea was largely confounded by morphological convergence based on similar ecomorphic adaptations in independent lineages. In addition, Wieland (2013) found the family to be non-monophyletic within a thorough morphological phylogenetic analysis.

The Neotropical Liturgusidae (*sensu* Ehrmann 2002) includes three described genera and 24 species that are distributed from the southern United States and a few Caribbean Islands to the southern regions of Brazil and Bolivia. *Liturgusa* Saussure, 1869, *Hagiomantis* Audinet Serville, 1838, and *Gonatista* Saussure, 1869 are all distinct genera, the first two forming a monophyletic lineage while *Gonatista* was not included in the phylogeny (Svenson and Whiting 2009). However, recently generated molecular data from a species of *Gonatista* from the Dominican Republic places the genus outside of Neotropical Liturgusidae (Rivera and Svenson, unpublished data). This study focuses primarily on the species included within *Liturgusa*, but the type specimens of the highly similar *Hagiomantis* species were examined to ensure species level placements within these two genera were correct.

## Taxonomic history

Jean-Guillaume Audinet Serville (1838: 199) was first to describe a Neotropical Liturgusini species with the description of *Mantis annulipes*, which De Haan later included within the *Oxypilus* subgroup within *Mantis* (De Haan 1842: 84).

More than 30 years later, Henri Louis Frédéric de Saussure (1869: 62) described another species, *Liturgusa cayennensis*, and created a new genus, *Liturgousa*, for his new species as well as *Mantis annulipes*. Saussure (1872: 260) later described *Liturgusa surinamensis*. Carl Stål (1877: 3, 40) changed the spelling of Saussure’s genus to *Liturgusa*, which is now the spelling of prevailing use (see Taxonomic History under the genus description below for nomenclature information).

In the same year, three new species were described, *Liturgusa lichenalis*, *Liturgusa superba*, and *Liturgusa nubeculosa* by Karl Eduard Adolph Gerstaecker (1889: 52–56) and *Liturgusa mesopoda* by John Obadiah Westwood (1889: 30). The publication by Westwood came slightly after that of Gerstaecker’s and included *Mantis annulipes*, *Liturgusa cayennensis*, *Liturgusa lichenalis*, *Liturgusa nubeculosa*, and his own new species *Liturgusa mesopoda*, which was the most complete reference to the species included within the group at the time.

The next species, *Liturgusa maya*, was described by Saussure and Zehntner (1894: 160) only as a variant of *Liturgusa cayennensis*, but was later elevated to a species by Samuel Hubbard Scudder (1901: 159).

William Forsell Kirby (1904: 271) included six species within *Liturgusa*: *Liturgusa annulipes*, *Liturgusa lichenalis*, *Liturgusa cayennensis*, *Liturgusa maya*, *Liturgusa mesopoda*, and *Liturgusa malagassa*. The last being a species from Madagascar and not related to the Neotropical Liturgusini (Svenson and Whiting 2009) and no longer included within the genus. Kirby also included *Liturgusa nubeculosa*, *Liturgusa superba*, and *Liturgusa surinamensis* within *Hagiomantis* without providing a justification for the move. Kirby furthermore fixed the type species for the genus to *Liturgusa cayennensis* Saussure, 1869.

Franz Werner (1906: 372) described *Liturgousa orientalis* from Tanzania, but later this species was moved to *Dactylopteryx* Karsch, 1892 with other species from the Afrotropical region.

Lucien Chopard (1911: 323, 1916: 164) recorded *Liturgusa mesopoda*, *Liturgusa cayennensis*, and *Liturgusa annulipes* from French Guiana, but his identification of *Liturgusa annulipes* is doubtful considering its currently recognized distribution.

Ermanno Giglio-Tos (1914: 77–78) described *Liturgusa peruviana* from Peru and *Liturgusa parva* from Brazil, citing that *Liturgusa parva* was similar to *Liturgusa annulipes*.

Morgan Hebard (1919b: 134) listed *Liturgusa mesopoda* as being from Jimenez Colombia, but the specimen Hebard examined is actually a female of a very large new species described herein (*Liturgusa stiewei* sp. n.), which may be why he identified it as the typically large *Liturgusa mesopoda*. Hebard (1922: 337) referenced a number of specimens of *Liturgusa cayennensis* from Central America, but this identification was probably incorrect (see below). Hebard (1924: 131) listed *Liturgusa cayennensis* as being from Ecuador, but the specimen was not located in the Academy of Natural Sciences of Drexel University, Philadelphia. In addition, the species location and identification records presented by Hebard (1929, 1932, 1933) are also not verifiable since the specimens could not be located.

Ermanno Giglio-Tos (1927: 292–295) treated the genus *Liturgusa* with a redescription of the genus, species redescriptions, and a species level key. Included within the work are eight species: *Liturgusa peruviana*, *Liturgusa cayennensis*, *Liturgusa mesopoda*, *Liturgusa nubeculosa*, *Liturgusa annulipes*, *Liturgusa parva*, and a new species, *Liturgusa charpentieri*. His new species was based on “non *Mantis annulipes*” specimens cited by Toussaint von Charpentier (1843). He also synonymized *Liturgusa lichenalis* with *Liturgusa annulipes* without justification or presentation of supporting observations. Giglio-Tos also cited *Mantis annulipes* as the type species for the genus. Interestingly, the species key provides a distinction between species with 7 or 8 posteroventral tibial spines as is done herein. However, the key is deficient in the characters used and includes *Liturgusa nubeculosa* within the 8 posteroventral spine group, which it does not exhibit.

Max Beier (1931: 14) described *Liturgusa atricoxata* based on a female specimen from the Zoological Museum in Hamburg (now named Biozentrum Grindel und Zoologisches Museum, Universität Hamburg, Germany). Then, Beier (1935: 11) provided a description of the genus as well as a list of nine species of *Liturgusa* that included *Liturgusa peruviana*, *Liturgusa cayennensis*, *Liturgusa maya*, *Liturgusa charpentieri*, *Liturgusa atricoxata*, *Liturgusa mesopoda*, *Liturgusa nubeculosa*, *Liturgusa annulipes*, and *Liturgusa parva*.

James Abram Garfield Rehn (1935) recognized early that the eight species included within *Liturgusa* were difficult to identify and that the group needed attention. Rehn went further to discredit the actions of Giglio-Tos in describing *Liturgusa peruviana*, which Rehn stated as an obvious junior synonym to *Liturgusa nubeculosa*. Rehn also examined the type of *Liturgusa lichenalis* to discredit Giglio-Tos’ action that synonymized the species with *Liturgusa annulipes*. Rehn dismissed the key provided by Giglio-Tos as it relies on color and misstated the characters of a number of species. Rehn also outlined the differences of *Liturgusa maya* and *Liturgusa cayennensis*, by which *Liturgusa maya* was described as a variant. He separated the two with morphological observations as well as stated that *Liturgusa maya* is mainly from Central America but can range into South America, while *Liturgusa cayennensis* is known from French Guiana. Rehn (1935: 202) also referenced specimens from Trinidad and identified them as *Liturgusa maya*, these turning out to be a new species described herein (*Liturgusa trinidadensis* sp. n.). Rehn (1935: 199–201) treated a species identified as *Liturgusa annulipes*, which later turned out to be *Liturgusa cursor* (see below). Rehn (1935: 204) also suggested that *Liturgusa atricoxata* may not actually be a member of the *Liturgusa*, but of another, possibly undescribed genus, a conclusion reached herein.

Marcello La Greca (1939: 2–5) thoroughly described the highly distinct species *Liturgusa guyanensis* with illustrations for two females collected in 1931 from Babooncamp (located in Guyana).

James Abram Garfield Rehn (1950) described two new species from Central America, one being previously treated as *Liturgusa annulipes* (Rehn 1935), but now named *Liturgusa cursor* (Rehn 1950: 369). The second species, *Liturgusa actuosa* (Rehn 1950: 377), was also described from Central America from specimens collected on Barro Colorado Island in Panama. Extremely thorough descriptions were provided along with figures, measurement data, and habits.

In addition to describing a new species of *Liturgusa*, Salvador de Toledo Piza Jr. described two new *Hagiomantis* species from Brazil including *Hagiomantis fluminensis* (1965) and *Hagiomantis parva* (1966). Years later he also described *Liturgusa sinvalnetoi* (1982: 94) from Piracicaba, Brazil. All three descriptions were brief, but did include measurement data as well as locality information.

Paulo S. Terra (1995) treated *Liturgusa* with a redescription as well as providing a list of 14 species in his study on Neotropical Mantodea. Included were *Liturgusa actuosa*, *Liturgusa annulipes*, *Liturgusa atricoxata*, *Liturgusa cayennensis*, *Liturgusa charpentieri*, *Liturgusa cursor*, *Liturgusa guyanensis*, *Liturgusa lichenalis*, *Liturgusa maya*, *Liturgusa mesopoda*, *Liturgusa nubeculosa*, *Liturgusa parva*, *Liturgusa peruviana*, and *Liturgusa sinvalnetoi*. Limited distributional information was also included.

Francisco J. Cerdá (1996: 75–76) listed three species in his treatment of Mantodea of Venezuela including *Liturgusa nubeculosa*, *Liturgusa maya*, and *Liturgusa cayennensis*. He also provided a description of the genus.

Julián A. Salazar E. (1998: 105, Fig. 5) listed *Liturgusa charpentieri* as a species found in Colombia, but based on the figure provided it resembles one of the new species described herein (*Liturgusa krattorum* sp. n. or *Liturgusa algorei* sp. n.). Salazar (1999: 10) also listed *Liturgusa charpentieri* and *Liturgusa mesopoda* from Para, Colombia, but the identification of *Liturgusa mesopoda* is doubtful based on that species being distributed within and around French Guiana.

In his dissertation, Lauro José Jantsch (1999: 47–48), listed both spellings of *Liturgusa*, but attributed the later spelling to Gerstaecker rather than Stål. Jantsch also provided a genus description and a list of 12 included species matching those presented by Terra (1995), but with *Liturgusa lichenalis* as a junior synonym of *Liturgusa annulipes* as well as citing *Liturgusa peruviana* (spelled as *Liturgusa peruana*) as a junior synonym of *Liturgusa nubeculosa*.

Jean-Michel Maes and Roger Roy (2000: 61) listed only one species from Nicaragua, *Liturgusa maya*.

Francesco Lombardo and Barbara Agabiti (2001: 90, 96–97) listed four species from Ecuador, three being new records for the country, which include *Liturgusa cayennensis*, *Liturgusa charpentieri*, *Liturgusa maya*, and *Liturgusa peruviana*.

Julián A. Salazar E. (2002: 124) listed three species from Para, Colombia including *Liturgusa cayennensis*, *Liturgusa charpentieri*, and *Liturgusa mesopoda*. This list reflects previous lists Salazar provided (1999) with the addition of *Liturgusa cayennensis*.

Reinhard Ehrmann (2002: 206–207) provided a genus description and a list of 14 described species of *Liturgusa* that includes *Liturgusa actuosa*, *Liturgusa annulipes*, *Liturgusa atricoxata*, *Liturgusa cayennensis*, *Liturgusa charpentieri*, *Liturgusa cursor*, *Liturgusa guyanensis*, *Liturgusa lichenalis*, *Liturgusa maya*, *Liturgusa mesopoda*, *Liturgusa nubeculosa*, *Liturgusa parva*, *Liturgusa peruviana*, and *Liturgusa sinvalnetoi*. Ehrmann also included an abbreviated bibliography for synonyms, type repository and sex, type locality and rough distribution for each species. As an aside, Ehrmann (2002: 163–164) listed *Hagiomantis fluminensis* Piza, 1965, *Mantis ornata* Stoll, 1813, *Hagiomantis pallida* Beier, 1942, *Hagiomantis parva* Piza, 1966, *Liturgusa superba* Gerstaecker, 1889, and *Liturgusa surinamensis* Saussure, 1872 for *Hagiomantis*.

Antonio Arnovis Agudelo Rondón (2004: 55) listed four species from Colombia, mostly reflecting previous lists by Salazar (1999), but with the addition of *Liturgusa maya* for the first time.

Daniel Otte and Lauren Spearman (2005: 132–133) included 14 species in their catalog identical to those listed by Ehrmann (2002), but provided additional bibliographic references for the genus and each species, type repository, and limited distributional information.

Antonio Arnovis Agudelo Rondón, Francesco Lombardo, and Lauro José Jantsch (2007: 116, 141–142) listed 13 valid species in their checklist of Neotropical Mantodea. *Liturgusa peruviana* was synonymized with *Liturgusa nubeculosa*.

David Yager and Gavin Svenson (2008: 556) included a *Liturgusa* species in a molecular based phylogeny for the first time. This was soon followed by Gavin Svenson and Michael Whiting (2009) including *Liturgusa maya* and *Liturgusa tessae* sp. n. in their molecular study. The two species were placed as sister to *Hagiomantis* within a large diversity of Neotropical Mantodea.

## Natural history

Members of the Neotropical Liturgusini are strictly associated with tree bark habitats, showing preference for smooth bark, presumably for ease of running. However, specimens have been found on trees with mossy covering across as much as fifty percent of the trunk.

**Figure 1.**
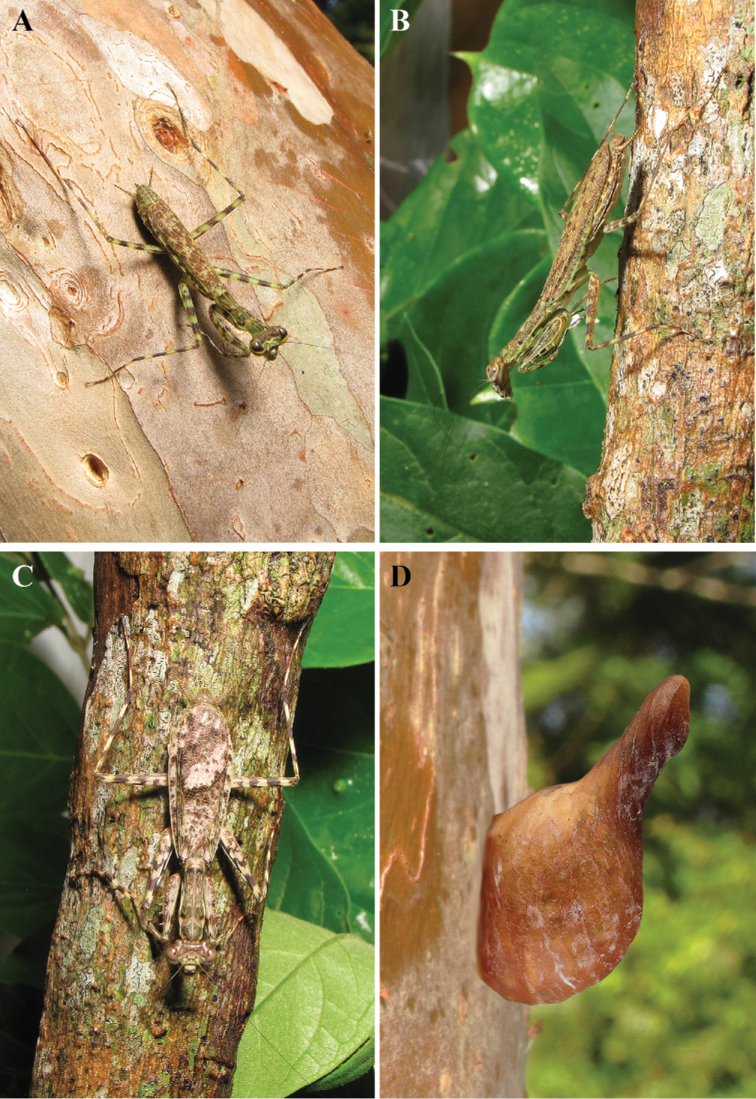
*Liturgusa* habitus of live specimens: **A** *Liturgusa cursor* Rehn, 1935, female from Nicaragua **B** *Liturgusa kirtlandi* sp. n., female from Bolivia **C** *Liturgusa nubeculosa* Gerstaecker, 1889, female from Bolivia **D** ootheca of unknown species of *Liturgusa*.

Individuals are typically found with the long axis of the body aligned with the vertical axis of the tree in a head down position (Fig. 1A–C). The posterior portions of the abdomen touch or nearly touch the bark while the head and prothoracic legs are held in an elevated position, the body angled relative to the trunk. Flight is rarely observed in all species of Neotropical Liturgusini, but males of *Liturgusa fossetti* sp. n. found in Costa Rica have been observed flying to nocturnal light traps (Lord *personal observation*), indicating some species may have more active flight in males than in females.

Being highly visual predators like other Mantodea, individuals easily detect approaching people and will run laterally to the opposite side of the tree when approached during daylight hours, although many have been observed assuming a crouching position before bolting. Individuals approached at night are far less prone to run, possibly due to low light conditions or more likely the effect of bright lights from flashlights obscuring or saturating their vision. If pursued, individuals run up the tree at a continuous gate or in spurts, but ultimately achieve unreachable heights. Although their exact speeds are not known, they run incredibly fast. Their speed and cursorial lifestyles on flat, but vertical tree trunks is reminiscent of ground hunting tiger beetles, a species of which, *Cicindela repanda* Dejean, 1825, was well characterized by Gilbert (1997). It would be interesting to conduct similar work on a species of *Liturgusa*.

Although diurnal capture is possible, nocturnal collecting proved to be far more productive. Using a spotlight or head lamp along with a large vial (50 ml), one can approach from a distance while shining the light on the mantis. They typically don’t move in this circumstance, which allows enough time to position the vial at an angle anterior to the head and either swiftly brought down over the specimen or with a touch to their posterior they run forward and inside the vial. Diurnal capture can be successful by searching trees with an insect net or curved branch, tapping the reverse side of trunks since they often have fled to the other side before being spotted. Tapping the reverse side of the trunk will drive them around to the visible side of the tree. By corralling individuals with hands and arms, which typically results in hugging trees, to lower positions on the trunk, a quick movement of a cupped hand over the specimen will achieve capture. On occasion, individuals will escape to the ground cover at the base of the tree, running rapidly and being still to avoid detection. Rarely, species have been observed taking flight directly off the tree and flying a moderate distance before dropping onto a plant or hitting the forest floor (males of *Liturgusa algorei* sp. n. in Peru). Larger females of *Liturgusa maya* have been observed to take flight, flutter a short distance and drop into leaf litter and exhibit thanatosis.

Most individuals are collected in the lower sections of tree trunks, but this may be mostly related to the limited searching ability of the collector that is walking through the forest. Specimens have been observed in higher regions of tree (*Liturgusa nubeculosa*, *Liturgusa fossetti* sp. n., and *Liturgusa krattorum* sp. n.) as well as within the crown of trees (*Liturgusa nubeculosa*) through canopy rope accession. Species are also plentiful in canopy fogging efforts like those performed by Terry Erwin in southern Peru where a large number of *Liturgusa* were sampled (*Liturgusa lichenalis*). It is not known if bark mantises have a preference for their vertical position on a tree or how extensively they use more terminal branches higher in the canopy since this is obviously difficult to observe, but would be interesting to research.

Although empirical data is lacking, a general trend of small species preferring smaller trunk diameters while larger species are typically more common on large trunked trees has been observed in Bolivia (*Liturgusa nubeculosa* and *Liturgusa maya*) and Nicaragua (*Liturgusa fossetti* sp. n. and *Liturgusa cursor*). This may be associated with a species’ size and/or speed and how quickly it can escape potential predators; slower species requiring smaller diameter trunks in order to disappear to the opposite side quickly. Trunk diameter preferences were found in *Ciulfina biseriata* Westwood, 1889, an Australian liturgusid bark mantis (O’Hanlon 2011), which could indicate similar preferences within the highly similar Neotropical bark mantises.

Specimens of *Liturgusa* have been collected by the author and colleagues in wet, low elevation rainforests in Peru, Bolivia, Nicaragua, Costa Rica, French Guiana, and Panama. However, *Liturgusa maya* was readily collected in seasonal deciduous forests in western Nicaragua during the dry season, which is interesting considering that *Liturgusa maya* has been sampled almost entirely within rainforest habitats. Although most species appear to be distributed within rainforests, there does not seem to be a strict adherence to habitat type across all of the species within *Liturgusa*. The forest types for species of *Corticomantis* gen. n., *Fuga* gen. n., and *Velox* gen. n. is unknown.

## Geographic range

With limited previous work on *Liturgusa*, other than the original species descriptions, the geographic distributions are not well known. A limited number of surveys and taxon lists have included various species of *Liturgusa*, but it is likely that many of the species identifications within these works are not congruent with the findings of this study. For example, the historical understanding of the distribution of *Liturgusa cayennensis* is far broader (Central America to French Guiana) than the current reconstruction of the species distribution (restricted to region immediately surrounding French Guiana). Therefore, most locality records of species cited within taxon lists have been ignored within this study in favor of establishing species distributions directly from georeferenced specimens examined for this study.

After gathering over 500 specimens for this study the range assessment for each species provides the most accurate measures of distribution to date. All species treated within this study are restricted to the Neoptropical region. The northernmost record is that of *Liturgusa maya* with a specimen collected in Mexico, a little north of Mexico City near the eastern coast. The southernmost record of *Liturgusa* are those of *Liturgusa nubeculosa* and *Liturgusa kirtlandi* sp. n. from just west of Santa Cruz, Bolivia. Species included within the newly created genera, *Fuga* gen. n. and *Velox* gen. n., are entirely restricted to southeast Brazil. This range is disjunct from the most eastern ranging species of *Liturgusa*.

A few of the species have broad ranges, covering thousands of square kilometers across the Amazon Basin (*Liturgusa nubeculosa* and *Liturgusa tessae* sp. n.) or with an extended north to south range from Mexico to southern Peru (*Liturgusa maya*). Most species have been found to be relatively restricted in their distribution, some only occurring within one country, island or even region of a country. Regional restriction is the consistent pattern across the group and is congruent with what would be expected of species that do not appear to fly or disperse readily. However, the broad ranges of the three aforementioned species warrants further investigation. A population genetics study would likely lead to even more diversity through discovery of cryptic species.

## Chromosomes

The chromosome composition of three species of *Liturgusa* has been characterized in multiple works by Sally Hughes-Schrader and her students (Hughes-Schrader 1943, 1948, 1950, 1951, 1953). Her interest in Mantodea for this work was likely due to a collaboration with James A.G. Rehn of the Academy of Natural Sciences of Drexel University, Philadelphia. *Liturgusa actuosa* was shown to have 23 chromosomes, *Liturgusa cursor* to have 33, and *Liturgusa maya* to have 17. All three species have a XO sex chromosome composition for males.

## Revision

This morphologically based revision assembled a large representation of *Liturgusa* specimens in order to address the impossibility of identifying most of the described species. With few keys present in the literature, most specimens of *Liturgusa* were either undetermined or misidentified. This lack of information for the genus has hampered our knowledge about their true diversity. In addition, based on their behavior, habitat usage and abundance in many environments they would make ideal study subjects for many types of ecological studies. In fact, a similar but unrelated Australasian bark mantis genus, *Ciulfina*, has received such attention with both taxonomic and ecological research, both of which have produced highly interesting results (Attard et al. 2009; Holwell 2008; Holwell and Herberstein 2010a, 2010b; Holwell et al. 2007, 2010; O’Hanlon 2011; Umbers et al. 2011).

The size of this study was not anticipated, but soon after gathering specimens and type material, it became clear that not only did synonymies exist, but a large number of new species were in need of description. The scope of this study then focused on the taxonomy of the genus alone with future plans to produce a robust morphological and molecular based phylogeny for the group.

The present study treats all described species of *Liturgusa* (11 species), describes 19 new species, identifies four synonymies, moves one species from *Liturgusa* to *Hagiomantis*, and creates three new genera (*Fuga* gen. n., *Velox* gen. n., and *Corticomantis* gen. n.) for species previously included in *Liturgusa* as well as *Hagiomantis*.

## Methods

**Specimens examined.** A total of 522 specimens of Neotropical Liturgusini were examined from 25 collections and museums. Approximately half were collected previous to 1950, thus incorporating a significant historical sampling from within the group. Each specimen was assigned a unique code to track locality, measurement data, and images. These codes will remain on the specimen pins, but it can’t be guaranteed that specimens will permanently retain the assigned code after return of loaned material. All data for examined specimens appear in tables prior to the descriptions, which include sex, type status, country of origin, locality data, georeferenced coordinates, and project code that includes repository abbreviations. In addition to the project codes, new database codes have been added to specimens from the National Museum of Natural History, Smithsonian Institution. A spreadsheet version of material examined tables can be downloaded at http://mantodearesearch.com.

**Georeferencing.** A specimen level map was created within Google Earth, each waypoint with a species specific marker and a description that includes specimen codes, label data, and type status. The KML file can be downloaded at http://mantodearesearch.com and opened in Google Earth and will enable users interactive capabilities when displaying distribution data. Specimens without GPS data included on the label were georeferenced using online map databases and Google Earth. All locality information presented in the material examined tables was reformatted and input into templates for conversion to KML format by Earth Point © (http://www.earthpoint.us/ExcelToKml.aspx).

**Descriptive conventions and character systems.** The species treated within this study are all extremely similar based on external morphology. However, key external features exist that easily separate the Neotropical genera. Species treatments within this study provide a brief diagnosis, new natural history observations, and verbal character descriptions stemming from the anterior surface of the head, the dorsal surface of the pronotum, the prothoracic leg (spine terminology following Wieland 2008, 2013), the wings, and the abdomen. The verbal descriptions are provided for males and females as well as the ootheca for one species. When females match male features, they are excluded from the female description. Therefore, only characters that differ slightly from the males are presented in the female descriptions, all other characters can be assumed to match the male.

**Male genital complex.** To extract the genital complex, terminal abdominal segments were dissected and placed in a hot weak KOH solution for 30 min to dissolve muscle tissue. Cleared genital structures were disarticulated and slide mounted with euparal or placed in glycerin inside microvials and pinned to the specimen. For species for which males are known, verbal descriptions of the male genital complex are provided. Nomenclature for the male genital complex follows Klass (1997) for the following features that were determined as informative for species diagnosis and description for this revision (see Fig. 51B.1 for labeled structure): apofisis falloid (afa), right dorsal phallomere (fallomero dorsale di destra; fda), main body of ventral left sclerite (L4A), main body of dorsal left sclerite (L4B), apical process (processo apicale; paa), distal process (processo distale; pda), ventral plate (piastra ventrale; pia), ventral process (processo ventrale sclerificato; pva), first sclerite of right phallomere (R1).

**Measurements.** A total of 335 specimens were measured using a Leica M165C stereo-microscope and an IC80 HD coaxial video camera using the live measurements module of the Leica Application Suite (LAS). Measurements captured in this study are extremely precise and approaching or matching this precision will be necessary to use the data to properly diagnose a specimen. In addition, accurate measurements are critical in the diagnostic key and without them species level identification will be much more difficult. A series of unique measurement metrics are present in the diagnostic key that are capable of separating species or groups of species. The definitions of these metrics are provided within the key itself and usually refer to one of the collected measurements or ratios described below.

All measurements presented in this study are in millimeters. A total of 21 measurement classes were captured including:

1. *Body length* = length of body from central ocelli to posterior tip of wing or abdomen (intraspecifically variable measurement, primarily for general size estimation).
2. *Forewing length* = from proximal margin of axillary sclerites to distal tip of the discoidal region.
3. *Hindwing length* = from proximal margin of axillary sclerites to distal tip of the discoidal region.
4. *Pronotum length* = from anterior margin to posterior margin.
5. *Prozone length* = anterior margin of pronotum to center of supra-coxal sulcus.
6. *Pronotum width* = from lateral margins at the widest point, the supra-coxal bulge.
7. *Pronotum narrow width* = from lateral margins of the pronotum at narrowest region of metazone.
8. *Head width* = from lateral margins of the eyes at widest point.
9. *Head vertex to clypeus* = from the vertex of the head at center to the lower margin of the frons and upper margin of clypeus.
10. *Frons width* = from lateral margins of frons, inferior to the antennal insertions, at the widest point.
11. *Frons height* = from upper margin abutting central ocellus to lower margin abutting clypeus.
12. *Prothoracic femur length* = from proximal margin abutting trochanter to distal margin of genicular lobe.
13. *Mesothoracic femur length* = from most proximal margin abutting trochanter to the distal side of the terminal spine insertion site.
14. *Mesothoracic tibia length* = from most proximal groove near joint with the femur to the distal side of the terminal spine insertion site.
15. *Mesothoracic tarsus length* = from proximal joint to the apex of the ungues curve.
16. *Metathoracic femur length* = from most proximal margin abutting trochanter to the distal side of the terminal spine insertion site.
17. *Metathoracic tibia length* = from most proximal groove near joint with the femur to the distal side of the terminal spine insertion site.
18. *Metathoracic tarsus length* = from proximal joint to the apex of the ungues curve.
19. *Anteroventral femoral spine count* = all inner marginal ridge spines and two proximal near marginal spines, but excluding the genicular spine.
20. *Anteroventral tibial spine count* = all inner marginal ridge spines, but excluding the distal terminal spur.
21. *Posteroventral tibial spine count* = all outer marginal ridge spines, but excluding the distal terminal spur.

The measurement of total body length was taken from the central ocellus to tip of posterior margin of abdomen or wing, which produced a variable measure only useful for general assessment of body size rather than species description. Since head position, abdominal expansion, and wing position are all variable, total body length should only be used as a rough measure class to initially discriminate between the small and large species when performing identifications.

A python script was written to provide measurement summaries as well as producing four ratios to provide informative measures of shape characteristics including:

1. *Pronotum elongation measure* = prozone length over total length of pronotum (a low measure indicates an elongate metazone).
2. *Pronotum shape measure* = pronotum width at the supra-coxal bulge over total length of pronotum (a low measure indicates an elongate pronotum).
3. *Head shape measure* = length of vertex of head to lower margin of frons over width of head (a low measure indicates a highly transverse head).
4. *Frons shape measure* = height of frons over width of frons (a low measure indicates a highly transverse frons).

For each species and sex, the specimen number (e.g. N=12) of measured individuals is presented prior to the summary measurements, which includes the range and the mean (for quantitative measurement classes) or mode (for meristic measurement classes) for each measurement class (e.g. 29–31 (30)). In cases where more than 15 specimens were present for any species, a subset was taken for measurements that was representative of total geographic range and size variation. Occasionally, certain class measures were not possible to collect (visibility of feature or absence of feature) and are reflected by their absence in the summary or the absence of a range in cases of multiple specimens examined.

The ultimate diagnostic characters to be used in combination include measurements, the structure of male genitalia, and shape of the pronotum. The morphology of the head and prothoracic legs are also helpful in a number of cases. Coloration patterns are used in this study, but these are conservative. If coloration is used in the description, the patterning was consistent across all examined specimens, but coloration not included was typically variable within species. Preservation of specimen, population level variation, and specimen age are all contributors to color alterations and make such characters somewhat untrustworthy. The vast majority of coloration patterning is not discussed as intraspecific variation makes them unreliable for delimiting a species.

**Imaging.** High resolution images of type and voucher specimens were captured using a Passport Storm© system (Visionary Digital™, 2012), which includes a Stackshot z-stepper, a Canon 5D SLR, macro lenses (50 mm, 100 mm, and MP-E 65 mm), three Speedlight 580EX II flash units, and an associated computer running Canon utility and Adobe Lightroom 3.6 software. The z-stepper was controlled through Zerene Stacker 1.04 and images were processed using the P-Max protocol. All images were captured over an 18% grey card background for white balance standards. Images were processed in Adobe Photoshop CS6 Extended to adjust levels, contrast, exposure, sharpness, and add scale bars (10 mm). Minor adjustments were made using the stamp tool to correct background aberrations and to remove distracting debris. Plates were constructed using Adobe Illustrator CS6.

**Figure 2.**
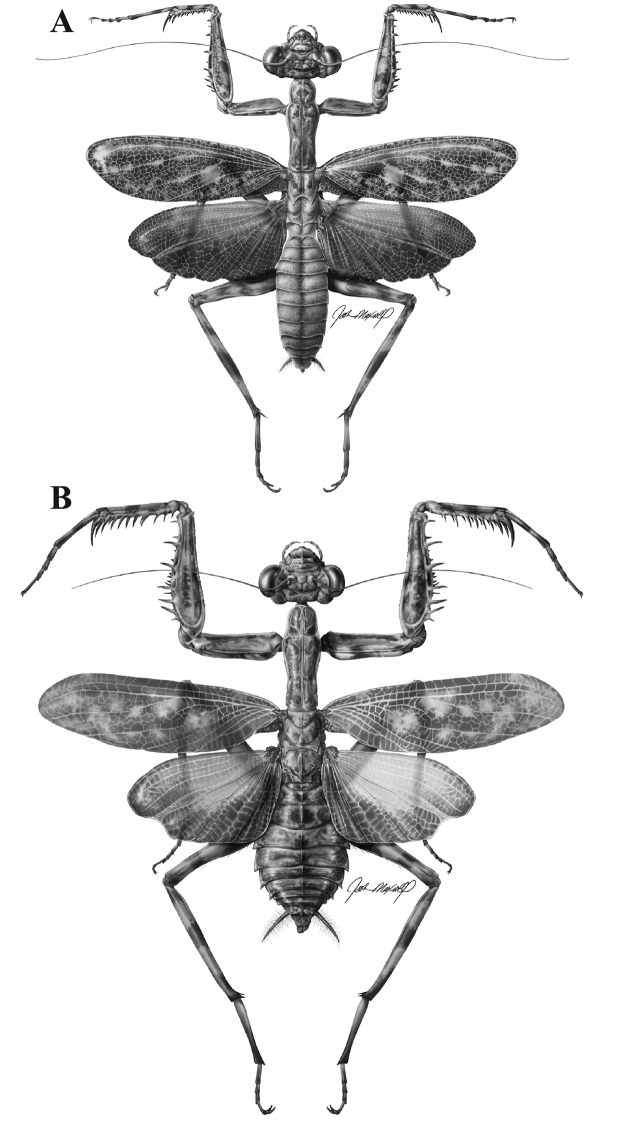
*Liturgusa fossetti* sp. n., dorsal habitus illustration: **A** holotype male from Panama (CLEV GSMC003836) **B** allotype female from Nicaragua (CLEV GSMC003425).

**Illustration.** Two dorsal habitus illustrations were produced by sketching on tracing paper before scanning into digital form with a flatbed scanner (Fig. 2). The sketches were imported into Adobe Illustrator to produce a full body fill before using Adobe Photoshop for detail work. Diagrammatic illustrations were produced by collecting reference images of the specimens using both the Leica M165C stereo microscope paired with the IC80 HD camera as well as the Passport Storm, Visionary Digital system. Images were imported into Adobe Illustrator and traced using an Intuos4 drawing tablet. Adobe Illustrator was used for all plate layouts. All illustrations (except the habitus of *Liturgusa maya* by Julio Rivera, Fig. 12B) were produced by Joshua Maxwell of the Cleveland Institute of Art.

**Specimen deposition.** Examined specimens are deposited or reside in the following institutions. New type material was deposited in accordance to specimen ownership with slide mounted male genital complex included. Museum codes are used throughout for brevity.

AMNH American Museum of Natural History, New York, NY, USA

ANSP Academy of Natural Sciences of Drexel University, Philadelphia, PA, USA

BMNH The Natural History Museum (British Museum Natural History), London, UK

CAS California Academy of Sciences, San Francisco, CA, USA

CJASE Collection Julian A. Salazar E, Manizales, Colombia

CLEV Cleveland Museum of Natural History, Cleveland, OH, USA (GSMC codes are all CLEV database record codes for the collection of Mantodea)

DZES Universidade de Sao Paulo, Piracicaba, Brazil

EMAU Ernst-Moritz-Arndt-Universität Greifswald, Germany

FMNH Field Museum of Natural History, Chicago, IL, USA

GSMC Gavin Svenson Mantodea Collection of Research, Cleveland Museum of Natural History, Cleveland, OH, USA

JAC Collection of J. Amith

LEM Lyman Entomological Museum, McGill University, Quebec, Canada

MALUZ Museo de Artrópodos de la Universidad del Zulia, Maracaibo, Venezuela

MEKRB Museo de Entomología *Klaus Raven Buller*, Universidad Nacional Agraria La Molina, Lima, Peru

MHNG Muséum d’Histoire naturelle, Geneva, Switzerland

MLBM Monte L. Bean Life Science Museum, Brigham Young University, Provo, Utah, United States of America

MNHN Muséum national d’Histoire naturelle, Paris, France

MNKM Museo de Historia Natural Noel Kempff Mercado, Santa Cruz, Bolivia

MSMC Martin Stiewe Mantodea Collection, London, United Kingdom

NYSM New York State Museum, Albany, NY

OUMNH Oxford University Museum of Natural History, Oxford, United Kingdom

ROM Royal Ontario Museum, Toronto, Canada

SDEI Senckenberg Deutsches Entomologisches Institut, Müncheberg, Germany

USNM National Museum of Natural History, Smithsonian Institution, Washington, DC, USA

ZMHB Museum für Naturkunde der Humboldt-Universität, Berlin, Germany

ZMUH Biozentrum Grindel und Zoologisches Museum, Universität Hamburg, Germany

## Taxonomic treatment

### Key to genus

**Table:** 

1	Prominent tubercles distributed across the posterior (upper) surface of the meso- and metafemora	*Hagiomantis* Audinet Serville, 1838
–	Posterior (upper) surface of the meso- and metafemora smooth	2
2	Number of posteroventral tibial spines 7, in extremely rare cases females may have 8 on one or both tibiae. The second most proximal posteroventral spines larger than others, excluding the most distal spine	*Liturgusa* Saussure, 1869
–	Number of posteroventral tibial spines 8. The third most proximal posteroventral spine larger than others, excluding the most distal spine	3
3	The prothoracic femoral pit that accommodates the terminal posteroventral tibial spine positioned between the two most proximal posteroventral spines, extending laterally abutting the posterior margin of the femur. Subgenital plate of male with pronounced styli	*Velox* gen. n.
–	The prothoracic femoral pit that accommodates the terminal posteroventral tibial spine positioned between the first most proximal posteroventral spines and the most distal discoidal spine. Subgenital plate of male without styli	4
4	Tubercles distributed across dorsal surface of the pronotum. *Pronotum shape measure* (pronotum width at the supra-coxal bulge over total length of pronotum) of male and female 0.51 or greater (observed range 0.51–0.55); pronotum short and broad. Distributed entirely in Central America or at most in northern South America (two specimens are from Colombia)	*Corticomantis* gen. n.
–	Dorsal surface of the pronotum is smooth or at most with very small tubercles widely dispersed. *Pronotum shape measure* (pronotum width at the supra-coxal bulge over total length of pronotum) of male and female 0.49 or less (observed range 0.33–0.49); pronotum more elongate. Distributed in eastern Brazil and not extending into the Amazon basin	*Fuga* gen. n.

**Clave Para Género** (*translation by Julio Rivera*)

**Table:** 

1	Meso- y metafemures con tuberculos prominentes distribuidos a lo largo de su superficie posterios (dorsal)	*Hagiomantis* Audinet Serville, 1838
–	Meso- y metafemures con superficie posterios (dorsal) lisa	2
2	Tibias anteriores con 7 espinas posteroventrales, aunque excepcionalmente las hembras pueden mostrar 8 espinas en una o ambas tibias. La 2da y 3ra espinas tibiales posteroventrales proximales más largas que las demás, excepto la más distal	*Liturgusa* Saussure, 1869
–	Tibias anteriores con 8 espinas posteroventrales. La 3ra espina tibial posteroventral más proximal es más larga que las demás, excepto la más distal	3
3	La espina posteroventral más distal de la tibia anterior se inserta, cuando flexionada contra el fémur, en una depresión ubicada entre las dos espinas posteroventrales más proximales de este último; dicha depresión se extiende lateralmente hasta alcanzar el margen posterior del fémur. Placa subgenital del macho con styli prominente	*Velox* gen. n.
–	La espina posteroventral más distal de la tibia anterior se inserta, cuando flexionada contra el fémur, en una depresión ubicada entre la primera espina posteroventral más proximal de este último y la espina discoidal más distal. Placa subgenital del macho sin styli	4
4	Superficie del pronotum con tubérculos. *Medida de la forma del pronotum* (i.e. ancho del pronotum a la altura de la dilatación supracoxal dividido por la longitud total del pronotum) del macho y hembra igual o superior a 0.51 (rango observado 0.51–0.55); pronotum corto y ancho. Distribución estrictamente Centroamericana o apenas hacia el norte de Sudamérica (dos especímens estudiados fueron obtenidos en Colombia)	*Corticomantis* gen. n.
–	Superficie del pronotum lisa o a lo mucho con túberculos muy pequeños y ampliamente dispersos. *Medida de la forma del pronotum* (i.e. ancho del pronotum a la altura de la dilatación supracoxal dividido por la longitud total del pronotum) del macho y hembra igual o menor a 0.49 (rango observado 0.33–0.49); pronotum más bien alargado. Distribución en el este de Brazil, no ocurren en la Cuenca del Amazonas	*Fuga* gen. n.

#### Liturgusa

Saussure, 1869

http://species-id.net/wiki/Liturgusa

**Figure 3.**
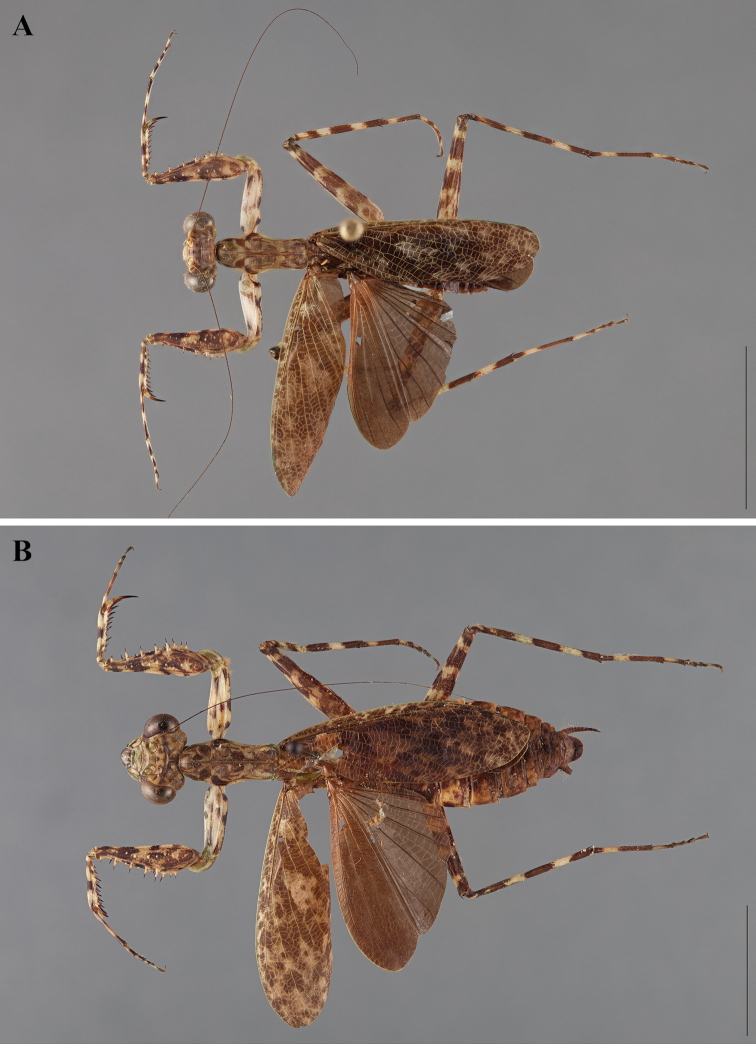
*Liturgusa cayennensis* Saussure, 1869, dorsal habitus: **A** male from French Guiana (MNHN 034) **B** female from Guyana (ANSP 049).

**Figure 4.**
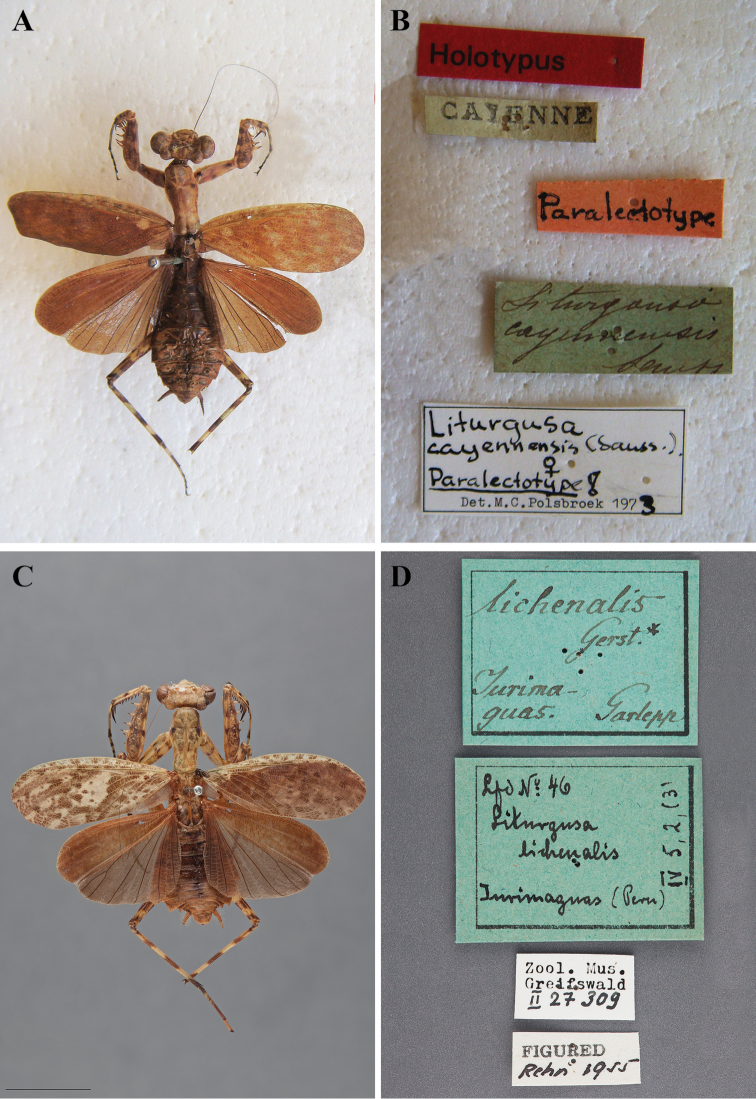
*Liturgusa cayennensis* Saussure, 1869, and *Liturgusa lichenalis* Gerstaecker, 1889, dorsal habitus of types and labels. *Liturgusa cayennensis*: **A** holotype female (MHNG) **B** labels. *Liturgusa lichenalis*: **C** holotype female (EMAU) **D** labels.

Liturgousa : Saussure 1869: 55, 62; Brauer 1870: 92; Saussure 1870: 225; Brauer 1871: 189; Saussure 1871a: 29, 51, 205; Saussure 1871b: 100; Saussure 1872a: 20, 53, 156; Saussure 1872b: 259; Stål 1877: 50; Westwood 1889: 4, 30; Brunner de Wattenwyl 1893: 63, 225; Saussure and Zehntner 1894: 129, 159; Saussure and Zehntner 1895: vii, 155, 157; Badenoch 1899: 39; Scudder 1901: 159, 407, 419; Waterhouse 1902: 202; Rehn 1903: 6; Kirby 1904: 271; Bruner 1906: 143; Werner 1906: 372; Werner 1908: 39; Werner 1909: 77–78; Chopard 1911: 323; Carl 1914: 148; Chopard 1916: 164; Werner 1916: 257, 274; Caudell 1918: 5; Hebard 1919a: 31; Hebard 1919b: 134; Hebard 1924: 131; Giglio-Tos 1927: 292 (“err. transscript.”), 699; Hebard 1929: 399; Hebard 1932: 211; Hebard 1933: 29; Rehn 1935: 172, 198–199, 201, 203–204, pl. 8, Figs 4–5; Hesse 1937: 108, 578; Tinkham 1937: 490–491; Neave 1939: 978; Hughes-Schrader 1943: 266, 280, 282–283, 290, 294, 296–297, Table 1, Figs 19–28; Hughes-Schrader 1948: 267; Rehn 1950: 369, 377; Hughes-Schrader 1950: 10–11, 13–14, 27, 38–39, 44–45, Table 1, Figs 9–11; Hughes-Schrader 1951: 178–181, 183–184, 186–187, Table 1–2, Figs 1–3; Beebe et al. 1952: 245–247, Fig. 2; Crane 1952: 259, 264, Figs 2, 3; Hughes-Schrader 1953: 544–554; Rehn 1954: 177, 179; Schrader and Hughes-Schrader 1956: 493–494, 496; Callan and Jacobs 1957: 201; Krombein 1963: 2; Henderson 1965: 206, 215; White 1965: 542; Marshall 1975: 318; Otte 1978: 76; Terra 1995: 53–54 (error); Cerdá 1996: 75–76, Fig. 1; Jantsch 1999: 47 (error transcript); Ehrmann 2002: 206 (*Liturgousa* err. descr.); Otte and Spearman 2005: 132, 481 (syn); Agudelo et al. 2007: 141 (syn); Roy and Cuche 2008: 8, 14, 21.
Liturgusa : Stål 1877: 3, 40; Gerstaecker 1889: 52–54 (*idem*); Bertkau 1889: 87; Giglio-Tos 1914: 77; Giglio-Tos 1916: 154; Giglio-Tos 1917: 64, 83; Carus 1921: 317; Hebard 1922: 337; Apolinar M. 1924: 47; Giglio-Tos 1927: 292; Beier 1931: 14; Brues and Melander 1932: 89, 644; Beier 1935: 6, 11; Rehn 1935: 198, 201, 204; Apolinar M. 1937: 226; La Greca 1939: 2, Fig. 1; Neave 1939: 978; Brues et al. 1954: 89, 878; Beier 1964: 943; Weidner 1964: 143; Beier 1968: 8, 14, 32; Marshall 1975: 322; Bazyluk 1977: 133, 169; Passerin d‘Entrèves 1981: 61; Piza 1982: 94; Terra 1995: 53–54, Figs 85–87; Salazar E. 1998: 105, Fig. 4; Edmunds and Brunner 1999: 282; Jantsch 1999: 19, 24, 30–31, 33, 35, 39, 47, Tables 4–6; Roy 1999: 30; Salazar E. 1999: 10; Maes and Roy 2000: 61; Salazar E. 2000: 67; Lombardo and Agabiti 2001: 90, 96–97; Salazar E. 2002: 121, 124; Ehrmann 2002: 26, 33, 206, 375; Agudelo and Chica 2002: 7, 20, 30, 36, 62, Fig. 8b; Agudelo and Chica 2003: 127, 130, 131, 132, 133, Tables 1, 3, Figs 1, 3, 7; Agudelo 2004: 44, 55, Table 3.1; Salazar E. 2004: 211, 213; Agudelo 2005: 3; Otte and Spearman 2005: 132, 481; Agudelo et al. 2007: 109, 116, 141; Medellín et al. 2007: 151; Roy and Cuche 2008: 8, 14, 21 (emendation by Stål); Yager and Svenson 2008: 556, 565; Wieland 2008: 158; Svenson and Whiting 2009: 503, Appendix S1; Wieland 2013: 22, 57, 87, 89, 130, 154, 158, 176, Figs 2, 4A, 20–21; Svenson in press.
Hagiomantis (*partim*): Kirby 1904: 271.
Liturguda : Jantsch 1991: 125.

##### Type species.

*Liturgusa cayennensis* Saussure, 1869 (Designation by Kirby 1904: 271).

##### Taxonomic history.

**Spelling of genus.** Two spellings of the genus name are present in the literature. The first is *Liturgousa*, established by Henri de Saussure in 1869 to include two species, *Mantis annulipes* Audinet Serville, 1838 and his newly described *Liturgousa cayennensis* Saussure, 1869. The second is *Liturgusa*, introduced by Carl Stål in 1877 while attributing the name to the original author, Saussure (1869). The name is derived from the Greek Liturgus (feminine form Liturga), meaning “celebrator of liturgy”, which indicates that Saussure’s original spelling of *Liturgousa* may have been a mistake, but under Article 32.5.1 of the ICZN (International Code of Zoological Nomenclature 4th edition) “incorrect transliteration or latinization, or use of an inappropriate connecting vowel, are not to be considered inadvertent errors” and thus is not demonstrably incorrect under Article 32.5 and stands as the correct original spelling under Article 32.2. The subsequent spelling of *Liturgusa* proposed by Stål appears to be an emendation, but since this emendation is applied to a correct original spelling and he also did not include a justification for his subsequent spelling (Art. 33.2.1), it is considered as an unjustified emendation under Article 33.2.3 of the Code. Under Article 33.2.3.1 the unjustified emendation (*Liturgusa*) becomes justified when it is in prevailing usage and is attributed to the original author and date. A comprehensive nomenclatural note pertaining to the spelling issue of *Liturgusa* has established such prevailing usage (Svenson in press), thus the unjustified emendation was determined as justified and is an available name that is used herein.

##### Type designation.

Upon creation of the genus *Liturgusa* by Saussure in 1869, two species, *Mantis annulipes* Audinet Serville, 1838 and *Liturgusa cayennensis* Saussure, 1869, were included, but neither were designated as the type species for the genus. Further, no type was established until subsequent designation, adherent to Article 69.1 of the Code, by Kirby (1904: 271) of *Liturgusa cayennensis* Saussure, 1869, which was valid under Article 67.2 of the Code as this species was an “originally included nominal species” available for fixation. However, Giglio-Tos (1927: 292) took subsequent action by designation of *Mantis annulipes* Audinet Serville, 1838, an act not valid according to Article 69.1.2 of the Code, which states that the first designation in a publication (Kirby 1904) is to be accepted. This type discrepancy was first recognized by Rehn (1935: 198) having stated in footnote “Giglio-Tos (Das Tierreich, Lief. 50, p. 292, (1927)), erroneously gives *annulipes* as the genotype. Kirby‘s fixation is the first, and, being made on one of the two originally included species, must be followed”. Unfortunately, recognition of *Liturgusa cayennensis* Saussure, 1869 as the type for the genus has not been uniform across taxonomic works (e.g. Ehrmann 2002 recognizes *Mantis annulipes* and Otte and Spearman 2005 recognizes *Liturgusa cayennensis*).

##### Redescription of the genus.

*Body*: The overall coloration of all *Liturgusa* species varies within a mottled or camouflage pattern that incorporates black, brown, pale tan, white or grey, and sometimes shades of green. The mottled patterns can be diffuse or highly contrasting with whitish regions abutting black spots or splotches. All species are dorsoventrally flattened with disproportionately long legs in comparison to body length.

*Measurement Ranges*: **Male.** Body length 18.59–29.16; forewing length 10.74–17.91; hindwing length 8.12–14.19; pronotum length 5.03–8.84; prozone length 1.55–2.49; pronotum width 1.90–3.18; pronotum narrow width 1.27–2.47; head width 4.36–6.34; head vertex to clypeus 1.55–2.67; frons width 1.39–2.39; frons height 0.53–0.93; prothoracic femur length 5.40–8.10; mesothoracic femur length 3.32–11.15; mesothoracic tibia length 4.78–8.83; mesothoracic tarsus length 4.33–8.07; metathoracic femur length 6.61–11.64; metathoracic tibia length 6.05–12.00; metathoracic tarsus length 6.24–11.52; pronotal elongation measure 0.26–0.34; pronotal shape measure 0.28–0.49; head shape measure 0.36–0.44; frons shape measure 0.30–0.47. **Female.** Body length 14.64–52.03; forewing length 12.36–26.96; hindwing length 9.29–21.20; pronotum length 5.16–13.12; prozone length 1.57–3.69; pronotum width 2.19–4.49; pronotum narrow width 1.43–3.09; head width 4.42–7.91; head vertex to clypeus 1.78–3.38; frons width 1.51–3.23; frons height 0.52–1.23; prothoracic femur length 5.22–12.24; mesothoracic femur length 6.47–14.62; mesothoracic tibia length 4.83–12.35; mesothoracic tarsus length 4.56–10.74; metathoracic femur length 6.56–14.87; metathoracic tibia length 6.90–17.08; metathoracic tarsus length 6.38–14.80; pronotal elongation measure 0.23–0.33; pronotal shape measure 0.29–0.52; head shape measure 0.38–0.47; frons shape measure 0.30–0.44.

*Head*: Wider than long with large, rounded eyes projecting outside the profile of the head both laterally and anteriorly (the anterior margin of the eyes anterior to the central surface of the head). Juxta-ocular protuberances present to varying degrees within males, but always well developed in females. The vertex between the parietal sutures is straight, concave or slightly irregular. Frontal suture with a medial carina. Ocelli present in males, but size is variable, protruding on cuticular mounds; reduced in females and laying more flatly on the surface or all three positioned laterally on a continuous curved carina. Central ocellus oriented anteriorly and lateral ocelli oriented outward, perpendicular to the central axis of the head or at most a few degrees off perpendicular. Frons narrowed between the antennal insertion sites and depressed below the central ocellus; a transverse carina present below the central ocellus, running from lateral margins under the antennal insertion sites medially in a dorsally oriented curve. Upper margin of clypeus convex, lower margin straight, concave, or convex; a transverse ridge medially; lateral margins tapering, widest at the upper margin. Labrum with minimal sculpting and a rounded terminus. Antennae filiform and with rare setae, pale or dark or a combination of both, never banded. Varying levels of black markings across the anterior surface that can include a transverse band or spots on the lower part of the frons, markings around the ocelli and the vertex, and markings on the clypeus, labrum and mandibles. Palpi are usually pale with or without a darkened terminus.

*Pronotum*: Varying from elongate (*pronotum shape measure* 0.28) to squat (*pronotum shape measure* 0.52) with a defined supra-coxal bulge; dorsal surface mostly smooth or at most with disperse tubercles, particularly in the posterior half. Prozone with lateral margins that are parallel, tapering anteriorly or rarely convex. Metazone with lateral margins that are parallel, concave, or tapering posteriorly; the dorsal surface often with laterally symmetrical bulges in the middle, which can push lateral margins outward. Coloration highly variable with pale and black markings. Supra-coxal sulcus strongly defined; posterior margin straight or medially emarginate.

*Prothoracic Legs*: Femoral spine count of male and female: anteroventral 12–17, posteroventral 4, discoidal 4. Femur robust with a straight or concave dorsal margin; anteroventral and posteroventral (internal and external, respectively) spines well developed; line of small tubercles running medially of the posteroventral spines. A continuous carina running from distal terminus of femur along dorsal margin to the base, circling the external surface of the proximal end and running along the ventral margin at the base of the posteroventral spines. Pale to dark banding on posterior (external) surface of femur; anterior (internal) surface pale with varying patterns of black markings. Posterior surface of femur smooth or with few tubercles. Well-developed femoral pit to accommodate terminal posteroventral tibial spine, positioned medial to the proximal two posteroventral spines; pit is colored black, brown or pale. Prothoracic tibial spine count of male and female: anteroventral 7–11, posteroventral 7. Prothoracic tibial spines robust; the posteroventral spines with the first smallest, the second and/or third longer, the third or fourth through sixth of similar length; the anteroventral spines longest at distal end and shortening proximally, but the seventh and eighth spines from the distal terminal spine longer than adjacent spines. Tarsus normal for Mantodea, but banded with pale and dark coloration. Prothoracic coxae smooth with no or a few very minor tubercles or setae along dorsal margin; black markings vary across species on the anterior, posterior, and ventral surfaces.

*Meso- and Metathoracic Legs*: Long and slender with pale to dark banding on the femur and tibia; dorsal surface of femora smooth. Femora with ventral (posterior) carina, some species being more pronounced than others; dorsal (anterior) carina noticeable in some species (Cayennensis Group in particular). Tibia long and rounded with well developed terminal spurs. Mesothoracic tarsi with first segment shorter, equal to or longer than remaining segments combined. Metathoracic tarsi with first segment always at least slightly longer than remaining segments combined, can be much longer.

*Wings*: Wings developed in males and females. Forewing mottled with brown, black, white, and green coloration; the costal region narrow relative to the wing length, the width between 2–5% the length, often with light to dark irregular banding; veins often marked with irregular sections of pale color. The forewings in many species may be colored asymmetrically, one being mottled as described above while the other is either dark rust or blackened with the mottled pattern still slightly visible (darker wing typically folded under the mottled wing). Hindwings hyaline, smoky opaque, and/or with rusty, yellow, or orange coloration; the terminus of the discoidal region either projecting beyond or within the profile of the distal margin of anal region.

*Abdomen*: Males and females with varying degrees of gradual widening from first segment until the beginning of the distal third (segments 5–7) at which point the lateral margins narrow to the terminus, the middle third being the broadest region. Some slender species with very slight widening, exhibiting near parallel margins before an abrupt narrowing as described above. Some species with pointed posterolateral tergal projections in the distal half of the abdomen of males and/or females, but other species with unmodified tergites. Cerci cylindrical, long and setose, tapering to a point. Supra-anal plate long or transverse, always with a rounded terminus of varying degrees. Subgenital plate of male with rounded, slightly irregular terminus; without styli.

*Male Genital Complex*: The distal end of main body of ventral left sclerite (L4A) is either smooth and rounded or with a distal process (pda) of varying size and shape. The apofisis falloid (afa) of the main body of dorsal left sclerite (L4B) well sclerotized with a highly variable terminus; the apical process (paa) cylindrical and curved, terminating in a rounded or blunt end. The right dorsal phallomere (fda) of the first sclerite of right phallomere (R1) tapers to a rounded terminus and is mostly membranous with disperse setae of varying robustness; the ventral plate (pia) strongly sclerotized with strongly defined grooves, slight grooves, or smooth; the ventral process (pva) strongly sclerotized and curved and/or tapering distally.

##### Ootheca

(Figs 1D, 21A–B). For known species, the ootheca is attached to solid substrate, usually tree bark. The main body is spherical with a tapering hollow tube originating from a dorso-medial position at about 45 degrees from a lateral perspective relative to attachment surface. Eggs positioned in a linear row medially in line with the tubicular process within an air-filled space. The size and volume of the air-filled space of the spherical body appears to vary across species (a considerably larger ootheca was examined for an unknown species in the Natural History Museum, London). Upon hatching, nymphs emerge through the tube to the outside. The number of eggs is unknown across the species.

##### Key to species

**Table:** 

1	*Pronotum shape measure* (measure = pronotum width at the supra-coxal bulge / total length of pronotum) of male 0.435 or greater, of female 0.45 or greater; pronotum short and broad (Fig. 47A–G). Metazone shape metric (metric = pronotum width at the supra-coxal bulge / length of metazone) of male 0.63 or greater, of female 0.63 or greater; short, broad metazone	2, Cayennensis Group
–	*Pronotum shape measure* of male 0.43 or less, of female 0.43 or less; pronotum slightly to highly elongate (Figs 47H–T, 48, 49). Metazone shape metric of male 0.61 or less, of female 0.615 or greater	5
**Cayennensis Group**		
2	Pronotum widened at the posterior end, the lateral margins protruding; distinct narrowing in the medial region of the metazone (Fig. 47E–F)	3
–	Pronotum with gradually narrowing lateral margins from supra-coxal bulge to posterior margin with no or very little central narrowing; posterior lateral margins not protruding and contained within the profile of the posterior terminus (Fig. 47A–D). Terminus of apofisis falloid (afa) of the main body of dorsal left sclerite (L4B) broad at base, quickly tapering to sharp or blunt point; often triangular (Fig. 51A–B)	4
3	Lateral margins of male and female prozone parallel or slightly divergent anteriorly (Fig. 47E–F). Discoidal region of hindwing of male and female with orange or rust coloration fading to smoke distally and into the anal region (Fig. 6A–B). Terminus of apofisis falloid (afa) of the main body of dorsal left sclerite (L4B) narrow and shaped like a finger (Fig. 51C.1). Distributed in northern Guyana and northern Brazil	*Liturgusa guyanensis* La Greca, 1939
–	Lateral margins of the female prozone converging anteriorly (Fig. 47G). Discoidal region of hindwing of female (only females known) yellow on proximal 90% and fading to black distally; yellow extending into the anterior portion of the anal region with strongly defined transition from yellow to hyaline with a slight smoke color along the distal margin (Fig. 7A). Only known from southern Venezuela near the Sierra de la Neblina	*Liturgusa neblina* sp. n.
4	Mesotarsi with first segment as long as the remaining segments combined. Distributed in eastern South America including French Guiana, Guyana, Suriname, and eastern Brazil. Lateral margins of female abdomen with slight or no tergal expansions	*Liturgusa cayennensis* Saussure, 1869
–	Mesotarsi with first segment shorter than the remaining segments combined. Distributed in western South America including Colombia, Ecuador, Peru, and southern Venezuela. Lateral margins of female abdomen with distinct tergal expansions that produce a posterior oriented point	*Liturgusa lichenalis* Gerstaecker, 1889
5	*Pronotum shape measure* of male 0.360 or greater, of female 0.370 or greater	6, Maya Group
–	*Pronotum shape measure* of male 0.355 or less, of female 0.36 or less; pronotum elongate and slender	15, Cursor Group
**Maya Group**		
6	Forewing length of female 26–27 mm, of male 19–20 mm. Length of pronotum of female 11–12 mm, of male 9–10 mm. Known only from Colombia	*Liturgusa stiewei* sp. n.
–	Forewing length of female 13–22 mm, of male 12–18 mm. Length of pronotum of female 6–10 mm, of male 5–8 mm	7
7	Pronotum highly sculpted in male and female with all of the following characteristics: prozone with highly convex margins; a defined supra-coxal bulge; medial region of metazonal lateral margins parallel or highly convex and bulging outward; posterior margins rounded in males, females with straightened posterior corners oriented 45 degrees from the central line of the body (Fig. 47E–F). Distributed only in Central America with records from Honduras, Guatemala, Belize, and Panama	*Liturgusa zoae* sp. n.
–	Pronotum of male and female not highly sculpted with all of the following characteristics: prozone with lateral margins that are near parallel or tapering anteriorly; a moderately defined supra-coxal bulge; medial region of metazonal lateral margins concave or bulging slightly; posterior margins in males and females rounded with a central emargination (*Liturgusa manausensis* male with straightened posterior corners oriented 45 degrees from the central line of the body) (Figs 47H–T, 48C–D)	8
8	Relatively small with a size metric range (size metric = length of forewing × length of pronotum) of 63–68 for males and 88–91 for females	9
–	Relatively large with a size metric range of 73–132 for males and 97–187 for females	10
9	Prothoracic femora of female with posteroventral genicular spine positioned proximal to the beginning of the genicular lobe. Male size metric approximately 16–17 (size metric = [length of pronotum / width of pronotum at narrow] × width of head). Distributed in northern Peru with one record just across the border in Brazil.	*Liturgusa bororum* sp. n.
–	Prothoracic femora of female with posteroventral genicular spine positioned distal to the beginning of the genicular lobe. Male size metric approximately 14. Distributed in northern Venezuela	*Liturgusa cura* sp. n.
10	Hindwings of male and female with yellow, orange or rust coloration on at least the proximal third of the discoidal region, fading to black distally (Figs 10A–B, 16A–B)	11
–	Discoidal region of the hindwings of male and female black or darkly opaque with at most the anterior costal margin with a lighter pale or rust coloration (Figs 11A–B, 12A, 13A–B, 17A–B)	12
11	Hindwings of male and female orange or yellow colored on the proximal ¾ of the discoidal region, sharply transitioning to black distally; the anal region mostly black or darkly opaque with the proximal third matching the orange color of the discoidal region (Fig. 10A–B). Size metric (metric = length of prothoracic femora / length of forewing) for males 0.45 or greater, for females 0.47 or greater. Ventral left sclerite (L4A) without distal process (pda), smoothly rounded (Fig. 51F.1). Distributed in Central America with records from Panama, Costa Rica, and Nicaragua	*Liturgusa fossetti* sp. n.
–	Hindwings of male and female orange or rust colored on the proximal ⅓ to ½ of the discoidal region, gradually fading to black distally; the anal region uniformly opaque black or smoky (Fig. 16A–B). Size metric for males 0.44 or less, for females 0.45 or less. Ventral left sclerite (L4A) with distal process (pda) (Fig. 51J.1–J.6)	*Liturgusa tessae* sp. n.
	Male genital dissection required for identification of *Liturgusa manausensis*, *Liturgusa kirtlandi*, *Liturgusa trinidadensis*, and *Liturgusa maya*.	
12	Male pronotal shape metric 0.59 (metric = [length of prozone / length of pronotum] × pronotum width at the supra-coxal bulge). Apofisis falloid (afa) of the main body of dorsal left sclerite (L4B) with a long, sharp point; ventral plate (pia) and ventral process (pva) of right phallomere (R1) large (Fig. 51H.1). Small species with asymmetrical forewing coloration, the contrast between dark and light highly distinct. Only males known and recorded only from Manaus, Brazil	*Liturgusa manausensis* sp. n.
–	Pronotal shape metric for males 0.63 or greater, for females 0.77 or greater. Apofisis falloid (afa) of the main body of dorsal left sclerite (L4B) with a short, but sharp point; ventral plate (pia) and ventral process (pva) of right phallomere (R1) small (Figs 51G.1–G.4, 51I.1–I4, 52A.1–A.5)	13
13	Apical process (paa) of dorsal left sclerite (L4B) flat, forming an angled pedestal (Fig. 51G.1–G.4). Relatively large with a pronotum length range of 6.95–7.35 mm (7.18 average) for males and 8.16–8.70 mm (8.43 average) for females. Recorded only from central Bolivia	*Liturgusa kirtlandi* sp. n.
–	Apical process (paa) of dorsal left sclerite (L4B) uniformly rounded (Figs 51I.1–I4, 52A.1–A.5). Relatively smaller overall with a pronotum range of 5.61–7.39 mm (6.25 average) for males and 6.55–8.99 mm (7.42 average) for females (only one male and two females of *Liturgusa maya* fall within the pronotum size range of *Liturgusa kirtlandi*)	14
14	Numerous tubercles in posterolateral corners of metazone in males and females. Antennae pale or gradually fade of a brown color in both sexes. Distal process (pda) of ventral left sclerite (L4A) with a sclerotized fold or slight projection centrally (Fig. 52A.1–A.5). Known only from Trinidad	*Liturgusa trinidadensis* sp. n.
–	No tubercles present on pronotum of males and females. Antennae of both sexes fade rapidly to black just distal of the base. Distal process (pda) of ventral left sclerite (L4A) with a strongly defined central process that is angled approximately 30 degrees from the central axis of the hypophallus, the tip rounded (Fig. 51I.1–I4). Broadly distributed from Mexico to southern Peru on the western side of South America	*Liturgusa maya* Saussure & Zehntner, 1894
15	Length of forewing of male less than 13.2 mm. Size metric (metric = length of forewing × length of pronotum) of male 92 or less, of female 125 or less. Ventral left sclerite (L4A) with a smooth, rounded terminus, lacking distal process (pda) (Fig. 52C.1–E.1)	16, Cursor Group A
–	Length of forewing of male 13.2 mm or greater. Size metric (metric = length of forewing × length of pronotum) of male 95 or greater, of female 130 or greater. Ventral left sclerite (L4A) with a distal process (pda) or serrated ridge (Fig. 52F.1–K.1)	18, Cursor Group B
**Cursor Group A**		
16	Supra-anal plate of male and female highly transverse, about three times as wide as long. Forewings lacking strongly pronounce white spot near the proximal end (Fig. 19A–B). Apofisis falloid (afa) of the dorsal left sclerite (L4B) finger-like with the terminus shaped like a slightly broadened bulb (Fig. 52C.1–C.3). Distributed only in Central America with records in Nicaragua, Costa Rica, and Panama	*Liturgusa cursor* Rehn, 1950
–	Supra-anal plate of male and female about twice as wide as long, the terminus rounded. Forewings with strongly pronounce white spot in the first proximal fifth, overlaying the bases of the media and cubitus veins (Figs 20A–B, 22A–B). Apofisis falloid (afa) of the dorsal left sclerite (L4B) triangular, terminating with a sharp point (Fig. 52D.1–E.1)	17
17	Anterior surface of forecoxae with a broad, black band medially in the proximal half as well as a black spot medially towards the distal terminus. Posterior margin of the prothoracic femora with a small spine between the most distal posteroventral spine and the genicular spine. Genicular spine positioned proximal to the beginning of the genicular lobe. Distributed in northern South America with records from French Guiana, but range could extend into Suriname and northern Brazil	*Liturgusa milleri* sp. n.
–	Anterior surface of forecoxa pale, without black markings. Posterior margin of the prothoracic femora without defined spine between the most distal posteroventral spine and the genicular spine. Genicular spine positioned distal to the beginning of the genicular lobe. Endemic to the island of Dominica	*Liturgusa dominica* sp. n.
**Cursor Group B**		
18	Discoidal area of the hindwings red, rust, or orange color proximally and fading to black distally (Figs 26A–B, 29A). Forewings of male and female often with asymmetrical coloration; when present, one is red or rusty	19
–	Discoidal area of the hindwings uniformly dark or smoky colored (Figs 7B, 23A–B, 24A–B, 25B, 27A–B). Forewings of male and female with or without asymmetrical coloration; when present, one is darkened black or smoky	20
19	Posteroventral prothoracic femoral spines of males and females long, most proximal spine around 0.50 mm and 1 mm, respectively (length of prothoracic femora ~14 and ~8.5 times the length of the most proximal posteroventral prothoracic femoral spine, respectively). Apofisis falloid (afa) of the dorsal left sclerite (L4B) a slender point, appearing like a needle; terminus of ventral left sclerite (L4A) with a sharp, slightly curved distal process (pda), but it can be reduced to a shorter projection with a pointed tip (Fig. 52I.1)	*Liturgusa krattorum* sp. n.
–	Posteroventral prothoracic femoral spines of male and female short, most proximal spine around 0.35 mm and 0.50 mm, respectively (length of prothoracic femora 18 and 15.5 times the length of the most proximal posteroventral prothoracic femoral spine, respectively). Apofisis falloid (afa) of the dorsal left sclerite (L4B) a broad triangle ending with a sharp point; terminus of ventral left sclerite (L4A) with a small blunt distal process (pda) (Fig. 52K.1)	*Liturgusa purus* sp. n.
20	Length of pronotum of male less than 7.1 mm, of female less than 8 mm	*Liturgusa actuosa* Rehn, 1950
–	Length of pronotum of male greater than 7.1 mm, of female greater than 8.5 mm	21
21	The length of the forewing is at most 1.80 times longer than the total length of the pronotum (observed range 1.67–1.80). Hindwings of male and female with a slight emargination between the discoidal and anal regions (Figs 7A, 25B)	22
–	The length of the forewing is at least 1.83 times longer than the total length of the pronotum (observed range 1.83–2.2). Hindwings of male and female with a prominent emargination between the discoidal and anal regions (Figs 25A–B, 27A–B)	23
22	The second most proximal posteroventral foretibial spine of female approximately 1.4 times the length of the third spine. Abdomen of female with slight posteriorolateral tergal projections that form a small posterior corner lip at most. Distributed in northern Venezuela with one record from northern Guyana	*Liturgusa cameroni* sp. n.
–	The second and third most proximal posteroventral foretibial spines of females roughly the same length, the second being slightly longer in most specimens (not more than 1.2 times the length of the third spine). Abdomen of female with pronounced posterolateral tergal projections that form small tooth-like projections between 0.15 mm and 0.21 mm in length. Distributed in northern French Guiana	*Liturgusa maroni* sp. n.
23	Mesotarsi of male with first segment shorter than the remaining segments combined. First tarsomere of female hindleg 3.5–8 times longer than the dorsal metatibial spur (spine relatively long). Distal process (pda) of ventral left sclerite (L4A) pointed, one side being serrated and heavily sclerotized; apofisis falloid (afa) of the dorsal left sclerite (L4B) with a broad, triangular terminus (Fig. 52J.1–J.2)	*Liturgusa nubeculosa* Gerstaecker, 1889
–	Mesotarsi of male with first segment equal to remaining segments combined. First tarsomere of female hindleg 10–12 times longer than the dorsal metatibial spur (spine relatively short). Distal process (pda) of ventral left sclerite (L4A) pointed, the tip rounded; apofisis falloid (afa) of the dorsal left sclerite (L4B) with slender, pointed terminus (Fig. 52G.1–G.2)	*Liturgusa algorei* sp. n.

##### Clave Para las Especies (translation by Julio Rivera)

**Table:** 

1	*Medida de la forma del pronotum* (medida = ancho del pronotum a la altura de la dilatación supracoxal dividido por la longitud total del pronotum) del macho igual o mayor a 0.435, el de la hembra igual o mayor a 0.45; pronotum corto y ancho. *Medida de la forma de la metazona* (i.e. ancho del pronotum a la altura de la dilatación supracoxal dividido por la longitud total de la metazona) del macho igual o superior a 0.63, en la hembra igual o superior a 0.63; metazona corta y ancha	2, Grupo Cayennensis
–	*Medida de la forma del pronotum* del macho y la hembra igual o menor a 0.43. Pronotum poco o muy elongado. *Medida de la forma de la metazona* (i.e. ancho del pronotum a la altura de la dilatación supracoxal dividido por la longitud total de la metazona) del macho igual o menor a 0.61, en la hembra igual o superior a 0.615	5
**Grupo Cayennensis**		
2	Pronotum ensanchado en su extremo posterior, márgenes laterales sobresalientes; región central de la metazona con estrechamiento notorio (Fig. 47E–F)	3
–	Pronotum con márgenes laterals estrechándose gradualmente en la sección comprendida entre la dilatación supracoxal y el márgen posterior, sin o con muy poco estrechamiento central; márgenes laterales no sobresalientes y contenidos dentro del perfil del márgen más posterior (Fig. 47A–D). Parte distal de la apófisis faloide (afa) den esclerito dorsal derecho del macho (L4B) amplio en la base, estrechándose súbitamente para formar una punta aguda o roma; frecuentemente triangular (Fig. 51A–B)	4
3	Márgenes laterales de la prozona paralela o ligeramente divergentes anteriormente en ambos sexos (Fig. 47E–F). Región discoidal de las alas posteriores de ambos sexos de color naranja oxido, tornándose ahumada distalmente y hacia la región anal (Fig. 6A–B). Ápice de la apófisis faloide (afa) del esclerito dorsal derecho del macho (L4B) estrecho y digitiforme (Fig. 51C.1). Distribución en el Norte de Guyana y Norte de Brasil	*Liturgusa guyanensis* La Greca, 1939
–	Márgenes laterales de la prozona de la hembra convergiendo anteriormente (Fig. 47G). Región discoidal de las alas posteriores de la hembra (unico sexo conocido) amarillo sobre el 90% más proximal y tornándose negro distalmente; la coloración amarilla se extiende hacia la porción anterior de la región anal, con una transición bien definida desde el amarillo hasta una condicioón más hialina, y con un tono mas ahumado hacia el márgen distal (Fig. 7A). Solo conocida para el Sur de Venezuela, cerca de la ”Sierra de la Neblina”	*Liturgusa neblina* sp. n.
4	Mesotarso con primer segmento tan largo como los demás combinados. Distribuidos en el este de Sudamérica incluyendo Guyana Francesa, Guyana, Surinam y este de Brasil. Márgenes laterales del abdomen de la hembra con ligeras expansions tergales o sin estas	*Liturgusa cayennensis* Saussure, 1869
–	Mesotarso con primer segmento más corto que los demás combinados. Distribución en el Oeste de Sudamérica, incluyendo Colombia, Ecuador, Perú y Sur de Venezuela. Márgenes laterales del abdomen de la hembra con expansions tergales notorias en forma de una punta orientada posteriormente	*Liturgusa lichenalis* Gerstaecker, 1889
5	*Medida de la forma del pronotum* del macho igual o mayor a 0.360, de la hembra igual o mayor a 0.370	6, Grupo Maya
–	*Medida de la forma del pronotum* del macho igual o menor a 0.355, de la hembra igual o menor a 0.36; pronotum alargado y delgado	15, Grupo Cursor
**Grupo Maya**		
6	Alas anteriores de la hembra 26–27 mm, del macho 19–20. Longitud del pronotum de la hembra 11–12 mm, del macho 9–10. Conocida solo para Colombia	*Liturgusa stiewei* sp. n.
–	Alas anteriores de la hembra 13–22 mm (rango observado 13.35–21.41 mm), del macho 12–18 (rango observado 12.36–17.91 mm). Longitud del pronotum de la hembra 6–10 mm (rango observado 6.26–9.53 mm), del macho 5-8 mm (rango observado 5.16–7.35 mm)	7
7	Pronotum del macho y la hembra muy esculpido, mostrando las siguientes características: prozona con márgenes fuertemente convexos; dilatación supracoxal definida; región media de los márgenes laterales de la metazona paralelas o fuertemente convexa y dilatándose posteriormente; márgenes posteriores redondeados en los machos, en las hembras con las esquinas posteriores rectas, las que forman un angulo de 45 grados con respecto a la linea central del cuerpo (Fig. 47E–F). Distribuida solo en Centro America, con registros en Honduras, Guatemala, Belize y Panama	*Liturgusa zoae* sp. n.
–	Pronotum del macho y la hembra no muy esculpido, mostrando las siguientes características: prozona con márgenes laterales casi paralelos o estrechándose anteriormente; dilatación supracoxal moderadamente definida; región media de los márgenes laterales de la metazona cóncavos o ligeramente dilatados; márgenes posteriores redondeados y con una emarginación central en ambos sexos (los machos de *Liturgusa manausensis* tienen las esquinas porteriores formando un angulo de 45 grados con respecto a la línea central del cuerpo (Figs 47H–T, 48C–D)	8
8	Relativamente pequeños. El producto de longitud de las alas anteriores multiplicado por la longitud del pronotum es igual a 63–68 en los machos, y 88–91 en las hembras	9
–	Relativamente grandes. El producto de longitud de las alas anteriores multiplicado por la longitud del pronotum es igual a 73–132 en los machos, y 97–187 en las hembras	10
9	Fémures anteriores de la hembra con espina genicular posteroventral posicionada proximalmente al origen del lóbulo genicular. Rango de la siguiente métrica relativa: (*longitud del pronotum / ancho menor del pronotum) × ancho de la cabeza*, igual a 16–17 en los machos	*Liturgusa bororum* sp. n.
–	Fémures anteriores de la hembra con espina genicular posteroventral posicionada distalmente al origen lóbulo genicular. Rango de la siguiente métrica relativa: (*longitud del pronotum / ancho menor del pronotum) × ancho de la cabeza*, aproximadamente 14 en los machos	*Liturgusa cura* sp. n.
10	Alas posteriores del macho y la hembra con una coloracion amarilla, naranja u oxido sobre al menos el tercio proximal de la region discoidal, tornandose negra distalmente (Figs 10A–B, 16A–B)	11
–	Región discoidal de las alas posteriores del macho y la hembra de color negro u oscuramente opacada, con a lo mucho el margen anterior del márgen costal de un color mas pálido o algo rojizo (Figs 11A–B, 12A, 13A–B, 17A–B)	12
11	Área discoidal de las alas posteriores del macho y la hembra naranja o amarillo en los ¾ proximales, rápidamente tornándose negro distalmente; región anal mayormente negra u oscuramente opacada, con el tercio proximal coloreado como la región discoidal (Fig. 10A–B). La métrica *longitud del fémur anterior / longitud de las alas anteriores* en los machos es igual o mayor a 0.45 (rango observado 0.45–0.48), e igual o mayor a 0.47 en las hembras (rango observado 0.47–0.52) Esclerito ventral izquierdo (L4A) sin proceso distal (pda) y suavemente redondeado. Distribución en Centro América, con registros de Panamá, Costa Rica y Nicaragua	*Liturgusa fossetti* sp. n.
–	Área discoidal de las alas posteriores del macho y la hembra naranja o rojo oxido en los 1/3 al ½ proximales, gradualmente tornandose negro hacia distalmente. Región anal uniformemente opaca, negra o ahumada (Fig. 16A–B). La métrica *longitud del fémur anterior / longitud de las alas anteriores* en los machos igual o menor a 0.44 (rango observado 0.4–0.44), e igual o menor a 0.45 en las hembras (rango observado 0.4–0.45). Esclerito ventral izquierdo (L4A) con proceso distal (pda) (Fig. 51J.1–J.6)	*Liturgusa tessae* sp. n.
	Diseccion de genitalia masculina es necessaria para la identificacion de *Liturgusa manausensis*, *Liturgusa kirtlandi*, *Liturgusa trinidadensis*, and *Liturgusa maya*.
12	Métrica de la forma del pronotum del macho 0.59 (métrica = [longitud de la prozona/longitud del pronotum] × ancho del pronotum a la altura de la dilatación supracoxal]. Apófisis faloide (afa) del esclerito dorsal izquierdo (L4B) con una punta larga y aguda; ventral plate (pia) y ventral process (pva) del falómero derecho (R1) grandes (Fig. 51H.1). Especie pequeña con coloración de las alas anteriores asimétrica y con un contraste muy notorio entre las regiones oscurecidas y las más claras. Especie conocida solo por los machos, registros solo para Manaus, Brasil	*Liturgusa manausensis* sp. n.
–	Métrica de la forma del pronotum del macho 0.63 o mayor (rango observado 0.63-0.86), en las hembras 0.77 o mayor (rango observado 0.77-1.12). Apófisis faloide (afa) del esclerito dorsal izquierdo (L4B) con una punta corta y aguda; ventral plate (pia) y ventral process (pva) del falómero derecho (R1) pequeños (Figs 51G.1–G.4, 51I.1–I4, 52A.1–A.5)	13
13	Proceso apical (paa) del esclerito dorsal izquierdo (L4B) plano, en forma de podio (Fig. 51G.1–G.4). Especie relativamente grande, pronotum del macho entre 6.95–7.35 (promedio 7.18), y hembras entre 8.16–8.7 (promedio 8.43). Registros solo para Bolivia central	*Liturgusa kirtlandi* sp. n.
–	Proceso apical (paa) del esclerito dorsal izquierdo (L4B) uniformemente redondeado (Figs 51I.1–I4, 52A.1–A.5). Tamaño relativamente pequeño, pronotum del macho entre 5.61–7.39 (promedio 6.25), y hembras 6.55–8.99 (promedio 7.42) (solo se observo un macho y dos hembras de *Liturgusa maya* con medidas del pronotum comparables a *Liturgusa kirtlandi*)	14
14	Esquinas posterolaterales de la metazona con numerosos tubérculos en ambos sexos. Antenas pálidas o gradualmente tornándose pardo en ambos sexos. Proceso distal (pda) del esclerito ventral izquierdo (L4A) centralmente con un pliegue esclerotizado o una ligera proyección (Fig. 52A.1–A.5). Registros solo para Trinidad	*Liturgusa trinidadensis* sp. n.
–	Sin tubérculos en el pronotum de ambos sexos. Antenas tornándose rápidamente negras distalmente en ambos sexos. Proceso distal (pda) del esclerito ventral izquierdo (L4A) centralmente con un proceso fuerte bien definido, el cual forma un ángulo de ca. 30 grados con el eje central del hypophallus, el ápice es redondeado (Fig. 51I.1–I4). Registros solo para Trinidad	*Liturgusa maya* Saussure & Zehntner, 1894
15	Longitud de las alas del macho igual o menor a 13.2. Métrica *longitud de las alas anteriores × longitud del pronotum* del macho igual o menos a 92, igual o menor a 125 en las hembras. Esclerito ventral izquierdo (L4A) con el ápice liso y redondeado, carente de proceso distal (pda) (Fig. 52C.1–E.1)	16, Grupo Cursor A
–	Longitud de las alas del macho igual o menor a 13.4. Métrica *longitud de las alas anteriores × longitud del pronotum* del macho igual o mayor a 95, igual o mayor a 130 en las hembras. Esclerito ventral izquierdo (L4A) con un proceso distal (pda) o un borde aserrado (Fig. 52F.1–K.1)	18, Grupo Cursor B
**Grupo Cursor A**		
16	Esclerito supra-anal del macho y la hembra fuertemente transversal, aproximadamente 3 veces tan ancho como largo. Alas anteriores sin una marca blanca fuertemete pronunciada cerca del extreme proximal (Fig. 19A–B). Apófisis faloide (afa) del esclerito dorsal derecho (L4B) digitiforme, con el ápice en forma de un bulbo ligeramente ensanchado (Fig. 52C.1–C.3). Distribución solo en Centro América, con registros para Nicaragua, Costa Rica y Panamá	*Liturgusa cursor* Rehn, 1950
–	Esclerito supra-anal del macho y la hembra aproximadamente 2 veces tan ancho como largo, con ápice redondeado. Alas anteriores con una marca blanca fuertemente pronunciada sobre el 1/5 proximal y sobre la base de las venas media y cubital (Figs 20A–B, 22A–B). Apófisis faloide (afa) del esclerito dorsal izquierdo (L4B) triangular, terminando en punta aguda (Fig. 52D.1–E.1)	17
17	Superficie anterior de la procoxa con una banda media de color negro en la mitad proximal, así como también una marca negra medial hacia el ápice. Margen posterior del fémur anterior con una espina pequeña entre la espina posteroventral más distal y la espina genicular. Espina genicular posicionada proximalmente al inicio del lóbulo genicular. Distribución en el norte de Sudamérica, con registros para Guyana Francesa, pero posiblemente distribuida también en Surinam y norte de Brasil	*Liturgusa milleri* sp. n.
–	Superficie anterior de la procoxa pálida, sin marcas negras. Margen posterior del fémur anterior sin una espina definida entre la espina posteroventral más distal y la espina genicular. Espina genicular posicionada distalmente al inicio del lóbulo genicular. Endémica de la isla Dominica	*Liturgusa dominica* sp. n.
**Grupo Cursor B**		
18	Parte proximal del área discoidal de las alas posteriores de color rojo, naranja u óxido, tornándose negro distalmente (Figs 26A–B, 29A). Ala anterior del macho y la hembra frecuentemente de coloración asimétrica, cuando esto ocurre, una de las alas tiene un tono rojizo	19
–	Área discoidal de las alas posteriores uniformemente oscurecida o ahumada (Figs 7B, 23A–B, 24A–B, 25B, 27A–B). Alas anteriores del macho y la hembra con o sin coloración asimétrica, pero cuando esta ocurre, una es oscura, negra o ahumada	20
19	Espinas posteroventrales del fémur anterior largas, tanto en machos como en las hembras, siendo la más proximal aproximadamente 0.5 y 1 mm, respectivamente (longitud del fémur anterior ~14 y ~8.5 veces la longitud de la espina posteroventral más proximal, respectivamente). Apófisis faloide (afa) del esclerito dorsal izquierdo (L4B) delgado, como una aguja; ápice del esclerito ventral izquierdo (L4A) con una proyección aguda y ligeramente curvada, aunque puede estar reducida a una distal process (pda) más corta y de ápice algo puntiagudo (Fig. 52I.1)	*Liturgusa krattorum* sp. n.
–	Espinas posteroventrales del fémur anterior cortas, tanto en machos como en las hembras, siendo la más proximal aproximadamente 0.35 y 0.5 mm, respectivamente (longitud del fémur anterior 18 y 15.5 veces la longitud de la espina posteroventral más proximal, respectivamente). Apófisis faloide (afa) del esclerito dorsal izquierdo (L4B) en forma de un triangulo amplio, terminando en una punta aguda; ápice del esclerito ventral izquierdo (L4A) con una distal process (pda) corta y roma (Fig. 52K.1)	*Liturgusa purus* sp. n.
20	Longitud del pronotum de los machos menor a 7.1, el de las hembras menor a 8	*Liturgusa actuosa* Rehn, 1950
–	Longitud del pronotum de los machos mayor a 7.1, el de las hembras mayor a 8.5	21
21	Longitud de las alas anteriores como máximo 1.80 veces más largo que la longitud total del pronotum (rango observado 1.67–1.80). Ala posterior del macho y la hembra con una emarginación entre las áreas discoidal y anal, poco o muy poco marcada (Figs 7A, 25B)	22
–	Longitud de las alas anteriores como minimo 1.83 veces más largo que la longitud total del pronotum (rango observado 1.83–2.2). Ala posterior del macho y la hembra con una obvia emarginación entre las áreas discoidal y anal (Figs 25A–B, 27A–B)	23
22	La segunda espina posteroventral más proximal de las tibias anteriores de la hembra, aproximadamente 1.4 veces la longitud de la tercera espina. Terga abdominal de la hembra con pequeñas expansiones en las esquinas latero-posteriores. Distribución en el norte de Venezuela, con un registro en el norte de Guyana	*Liturgusa cameroni* sp. n.
–	La segunda y tercera espina posteroventral mas proximales de las tibias anteriores de la hembra, casi de la misma longitud, con la segunda siendo solo ligeramente más larga en la mayoría de especimens examinados (no más de 1.2 veces la longitud de la tercera espina). Terga abdominal de la hembra con expansiones pronunciadas en las esquinas latero-posteriores de aproximadamente 0.15 y 0.21 mm de longitud. Distribución en la cuenca del Amazonas	*Liturgusa maroni* sp. n.
23	Primer tarsómero de la pata posterior de la hembra 3.5–8 veces más largo que la espina apical dorsal de la metatibia (dicha espina es relativamente larga). Proceso distal (pda) del esclerito ventral izquierdo (L4A) en punta, uno de sus lados es aserrado y fuertemente esclerotizado; apofisis faloide (afa) del esclerito dorsal izquierdo (L4B) con un apice amplio y triangular (Fig. 52J.1–J.2).	*Liturgusa nubeculosa* Gerstaecker, 1889
–	Primer tarsómero de la pata posterior de la hembra 10-12 veces más largo que la espina apical dorsal de la metatibia (dicha espina es relativamente corta). Proceso distal (pda) del esclerito ventral izquierdo (L4A) en punta redondeada; apofisis faloide (afa) del esclerito dorsal izquierdo (L4B) con un apice Delgado y en punta (Fig. 52G.1–G.2)	*Liturgusa algorei* sp. n.

#### Cayennensis Group

##### Liturgusa cayennensis

Saussure, 1869

http://species-id.net/wiki/Liturgusa cayennensis

Liturgousa cayennensis : Saussure 1869: 62; Brauer 1870: 92; Saussure 1871b: 101–102; Saussure and Zehntner 1894: 159–160; Westwood 1889: 5, 50; Scudder 1901: 159, 407; Kirby 1904: 271; Chopard 1911: 323; Chopard 1916: 164; Hebard 1919a: 31; Hebard 1924: 131; Hebard 1929: 399; Hebard 1933: 29; Rehn 1935: 198; Beebe et al. 1952: 246; Cerdá 1996: 75–76; Roy and Cuche 2008: 8, 21.
Liturgusa cayennensis : Hebard 1922: 337; Giglio-Tos 1927: 293; Beier 1935: 11; Jantsch 1991: 125; Terra 1995: 53; Jantsch 1999: 47–48; Salazar E. 2000: 67; Lombardo and Agabiti 2001: 90, 96; Salazar E. 2002: 124; Ehrmann 2002: 206; Agudelo 2004: 55, Table 3.1; Agudelo 2005: 3; Otte and Spearman 2005: 132; Agudelo et al. 2007: 116, 141.
Liturgousa cayennesis : Bruner 1906: 143.

###### Type.

Holotype Female. Muséum d’Histoire naturelle, Geneva, Switzerland

###### Type locality.

Cayenne. (French Guiana)

###### Material examined.

*Liturgusa cayennensis* Saussure, 1869.

**Table:** 

Sex	Type	Country	Label	Latitude Longitude	Code
Male	nontype	Guyana	B.G. 5-1-1923, W. Bank, Dem. R.		AMNH 030
Female	nontype	Guyana	Kartabo, Bartica District, British Guiana, 1921	6.242050, -59.306552	ANSP 046
4 Females	nontype	Guyana	Bartica, British Guiana, H.S. Parish, 1.20.1912	6.405831, -58.625444	ANSP 048-51
Male	nontype	Suriname	Ongelijk, Para, R. Surinam, May‚ 27		ANSP 054
Male	nontype	French Guiana	Guyane Francse, Nouveau Chantier, collection Le Moult, Coll. L. Chopard, 1919, Mai		ANSP 055
Female	nontype	Guyana	Guyane, 1907-247.		BMNH 007
Male	nontype	Brazil	Mato Grosso, Serra do Roncador, 264 km N. of Xavantina, near base camp, 1967-9, I.R. Bishop, RS/RGS exp. B.M. 1981-312	-12.456161, -52.054393	BMNH 076
Male	nontype	Guyana	New River, 750 ft., 10-20.III.1938, C.A. Hudson	2.543686, -57.583411	BMNH 086
Male	nontype	French Guiana	Kaw Mountain Res., Amazonas Lodge, 4°32'57.8"N, 52°12'49.7"W, 8-19 Feb 2005, Coll: K.B. Miller	4.549389, -52.213806	GSMC000262
Nymph	nontype	French Guiana	Maripasoula, Saul. Campsite Chez Fred‘s, 03°37'42.0"N, 53°12'40.0"WEle:26m, Manicured landscape surrounded by Primary tropical rainforest, Hand collected from leaf litter samples, Dec. 14-15, 2004, Coll: J. Huff	3.628333, -53.211111	GSMC003069
Female	nontype	French Guiana	St-Jean du Maroni, Collection Le Moult, Coll. L. Chopard, 1919, Fevrier	5.487038, -54.008462	MNHN 001
Female	nontype	French Guiana	Nouveau Chantier, collection Le Moult, Coll. L. Chopard, 1919, June		MNHN 002
Male	nontype	French Guiana	Cayenne	4.914356, -52.301354	MNHN 005
Female	nontype	Brazil	Amazonas Reserva Biologica do Cuieiras, 50 km. N. Manaus, 15-IV-AU 15-V-1981, M. Descamps, Bresil	-2.599556, -60.210631	MNHN 0059
Male	nontype	French Guiana	Cayenne	4.914356, -52.301354	MNHN 007
Female	nontype	French Guiana	Saut Dalles, 24-VIII-1992, P. Peters, Guyane francaise	3.291071, -53.835810	MNHN 0075
Female	nontype	French Guiana	Kourou, Guyane Franc, Coll: A. Bonhoure, 1909, Avril	5.163511, -52.656581	MNHN 011
Female	nontype	French Guiana	St-Jean du Maroni, Collection Le Moult, Coll. L. Chopard, 1919, Decembre	5.487038, -54.008462	MNHN 012
Female	nontype	French Guiana	Nouveau Chantier, collection Le Moult, Coll. L. Chopard, 1919, Novembre		MNHN 013
Female	nontype	French Guiana	St-Jean du Maroni, Collection Le Moult, Coll. L. Chopard, 1919, Fevrier	5.487038, -54.008462	MNHN 014
Male	nontype	French Guiana	Petit Saut, 8-II-1994, P.E. Rouland	5.069416, -53.047566	MNHN 034
Female	nontype	French Guiana	Nouveau Chantier, collection Le Moult, Coll. L. Chopard, 1919, Novembre		MNHN 050
Male	nontype	French Guiana	Sinnamary, VII-1977, Guyane, M. Descamps rec.	5.370512, -52.960320	MNHN 065
Male	nontype	French Guiana	Nouveau Chantier, collection Le Moult, Coll. L. Chopard, 1919, Mai		MNHN 066
Male	nontype	French Guiana	St-Jean du Maroni, Collection Le Moult, Coll. L. Chopard, 1919, Janvier	5.487038, -54.008462	MNHN 070
Male	nontype	French Guiana	St-Jean du Maroni, Collection Le Moult, Coll. L. Chopard, 1919, Janvier	5.487038, -54.008462	MNHN 071
Female	nontype	French Guiana	Pied Saut Parare, 12-VIII-1977, Guyane, M. Descamps Rec.	4.046724, -52.698087	MNHN 083
Female	nontype	Guyana	Mazaruni-Potaro: Waratuk Falls, Potaro R, 1 rainforest 300 ft, 1 Oct 1990, ROM 905028, LD Coote	5.272097, -59.397117	ROM 003
Male	nontype	Suriname	ex. coll. H. Dohrn		SDEI 002
Female	nontype	French Guiana	St-Laurent du Maroni, Collection Wm Schaus	5.487038, -54.008462	USNM 033: USNM ENT 00873000
Male	nontype	Brazil	Brasilien Amazonas, Parintins, Dr. F. Knutsen	-2.653184, -56.732240	ZMHB 001
Female	nontype	Brazil	Amazonas, oberer Solimoes., Rio Tomantins 9.26, Eing.Nr.33. 1937		ZMUH 005
Male	nontype	French Guiana	unknown		MNHN 074

###### Taxonomic history.

The oldest species described within the genus is also the type species for *Liturgusa*, designated by Kirby in 1904. Although the species was included in numerous taxonomic works, the species was likely confused with numerous others since some of these works focus on regions where *Liturgusa cayennensis* does not range. Therefore, it is impossible to tell what species they had misidentified as *Liturgusa cayennensis*. It was Rehn (1935) that first started to notice that *Liturgusa cayennensis* was more geographically restricted and that many previous records for the species in Central America were not correct. He noticed that it was being confused with *Liturgusa maya* most often. For *Liturgusa*, identification has always been a problem and most previous records of *Liturgusa cayennensis* should be viewed carefully and probably not included within distributional studies.

###### Diagnosis.

A short and stocky species with shortened, rounded forewings, a short and broad pronotum, and a broad abdomen. The forewings are usually asymmetrical in coloration, the darker being rust colored. Most similar to *Liturgusa lichenalis*, but lacking the prominent posterolateral tergal projections. Both *Liturgusa guyana* and *Liturgusa neblina* are similar, but both have a more pronounced constriction in the metazone of the pronotum. Finally, *Liturgusa lichenalis* is restricted to the western Amazon basin while *Liturgusa cayennensis* is found in central and eastern regions of the Amazon basin.

###### Description.

**Male.** (Fig. 3A) N=9: Body length 19.22–22.58 (20.93); forewing length 12.49–14.98 (13.61); hindwing length 10.26–11.37 (10.63); pronotum length 5.31–6.01 (5.60); prozone length 1.68–2.00 (1.79); pronotum width 2.38–2.70 (2.53); pronotum narrow width 1.77–1.98 (1.85); head width 4.84–5.39 (5.03); head vertex to clypeus 1.83–2.07 (1.96); frons width 1.67–1.87 (1.79); frons height 0.64–0.82 (0.72); prothoracic femur length 5.52–6.57 (6.00); mesothoracic femur length 7.14–8.20 (7.43); mesothoracic tibia length 5.34–6.38 (5.70); mesothoracic tarsus length 4.33–5.71 (5.13); metathoracic femur length 6.75–8.36 (7.43); metathoracic tibia length 6.05–8.51 (7.49); metathoracic tarsus length 6.24–8.17 (7.10); pronotal elongation measure 0.30–0.34 (0.32); pronotal shape measure 0.44–0.46 (0.45); head shape measure 0.37–0.40 (0.39); frons shape measure 0.37–0.44 (0.40); anteroventral femoral spine count 12–16 (16); anteroventral tibial spine count 10; posteroventral tibial spine count 7.

*Head* (Fig. 40A): Transverse, the juxta-ocular protuberances small, but pronounced, the apex in the lateral half; the vertex is straight, even with the dorsal margin of the eyes. Frontal suture with a medial carina forming a continuous arc, most pronounced medially, the region just ventral depressed. Ocelli small and protruding on small cuticular mounds, but the region between all three slightly raised; the lateral ocelli oriented outward. The carina on the frons pronounced, the medial region just ventral to the carina depressed. Clypeus transverse, the upper margin convex, the lower margin slightly concave; the central carina strongly pronounced and straight. Antennae pale at the base, the flagellum fading to dark brown just slightly distal to the base. Black band extending straight over the medial carina of the frontal suture, the medial portion of the carina pale; a branch of the black band extends ventro-laterally between the eye and antennal insertion at a forty five degree angle; a branch of the black band extends dorsal-medially from the lateral region of the main transverse band and terminating near the ventral terminus of the parietal suture. Lower region of frons darkened; the clypeus is pale; the mandibles and labrum mostly pale, but with some brown marks; the vertex and juxta-ocular protuberances pale and brown; the area immediately adjacent to lateral ocelli black. Palpi are pale.

*Pronotum* (Fig. 47A): Short and squat with a moderately defined supra-coxal bulge; dorsal surface entirely smooth. Prozone squat with margins gradually tapering anteriorly to a rounded anterior margin; the lateral margins smooth. Metazone with concave lateral margins, tapering posteriorly until about two thirds distance from supra-coxal bulge then widening gradually to the posterior margin; margins smooth; posterior margin with a slight medial emargination; the dorsal surface of the posterior third of the metazone depressed; two small and elongate bulges present on the dorsal surface near the posterior margin and positioned laterally. Pale with strong black marks across the surface, two prominent black marks laterally just posterior to the supra-coxal sulcus.

*Prothoracic Legs*: Femur squat and robust with a near straight dorsal margin; strongly defined pale to dark banding on posterior (external) surface; anterior (internal) surface with a very thin black band running medially from the base to terminus, a black mark dorsal to the band at the midpoint, and a dark mark dorsal to the band near the femoral brush; the ventral surface pale. Posterior surface of femur with few tubercles. A shallow femoral pit to accommodate terminal posteroventral tibial spine positioned medial to and exactly between the first two proximal posteroventral spines, but slightly distal to the most distal discoidal spine; pit is pigmented darkly. Posterior prothoracic femoral genicular spine slightly smaller than posteroventral spines, originating distal to the beginning of the genicular lobe. Prothoracic tibial posteroventral spines with the first (proximal) smallest and the fourth through sixth of similar length, the second and third are slightly longer. Prothoracic coxae smooth, the anterior surface with a very small, black mark medially in the proximal half as well as a very small black spot medially towards the distal terminus.

*Meso- and Metathoracic Legs*: Femora with ventral (posterior) carina; dorsal (anterior) carina pronounced. Mesotarsi with first segment as long as the remaining segments combined.

*Wings*: Forewings mottled with brown, pale and greenish coloration; the costal region without strongly defined banding, green and brown proximally with some low contrasting bands developing distally; veins are brown with cells being dark brown or light brown; two pale spots are positioned in the proximal quarter of the discoidal region just posterior to the first radial vein; a large pale spot is positioned centrally. Forewings often, but not always asymmetrically colored; one being mottled as described the other is darkened significantly with a black or rust tone, the mottled pattern still visible; extending just beyond or as long as the abdomen. Hindwings opaque brown, the discoidal region more pale proximally; the costal region light brown proximally, darkening distally; the terminus of the discoidal region projecting beyond the distal margin of anal region, the wing appearing slightly elongate.

*Abdomen*: Broad, widening until the fifth tergite before a gradual posterior narrowing; a smooth, brown and black colored dorsal surface. Tergites without posterolateral tergal projections. Supra-anal plate slightly transverse, a rounded terminus. Subgenital plate irregularly rounded and without styli.

*Genital Complex* (Fig. 51A.1–A.2): The main body of ventral left sclerite (L4A) with rounded terminus, but with a short, laterally positioned distal process (pda) that is rounded and sometimes projecting at an angle towards the medial axis of the L4A that can create a strongly angled transition from the terminal margin of the L4A to the medial margin of the pda; sometimes a depression on the lateral half is present. The apofisis falloid (afa) of the main body of dorsal left sclerite (L4B) short, broad and tapering to a point and heavily sclerotized, often curved; the apical process (paa) short, cylindrical and curved, the terminus a rounded end. The right dorsal phallomere (fda) of the first sclerite of right phallomere (R1) tapers to a rounded, membranous terminus; the ventral plate (pia) long with a rough surface, but mostly lacking strongly defined grooves though sometimes present; the ventral process (pva) smooth and tapering to a point distally, one edge straight and the other convex, tooth-like in appearance.

###### Redescription.

**Female.** (Figs 3B, 4A) N=13: Body length 14.64–32.95 (28.65); forewing length 17.01–20.50 (19.21); hindwing length 14.50–16.43 (15.64); pronotum length 7.03–8.11 (7.70); prozone length 2.23–2.63 (2.46); pronotum width 3.32–4.14 (3.63); pronotum narrow width 2.30–3.09 (2.60); head width 6.40–7.13 (6.81); head vertex to clypeus 2.74–3.10 (2.93); frons width 2.46–2.92 (2.73); frons height 0.98–1.14 (1.07); prothoracic femur length 7.46–9.15 (8.29); mesothoracic femur length 8.41–9.91 (9.12); mesothoracic tibia length 5.34–7.81 (7.05); mesothoracic tarsus length 5.10–7.06 (6.46); metathoracic femur length 8.62–9.78 (9.18); metathoracic tibia length 9.45–10.86 (9.97); metathoracic tarsus length 8.37–9.57 (9.13); pronotal elongation measure 0.31–0.33 (0.32); pronotal shape measure 0.45–0.52 (0.47); head shape measure 0.42–0.44 (0.43); frons shape measure 0.36–0.43 (0.39); anteroventral femoral spine count 15–16 (16); anteroventral tibial spine count 10; posteroventral tibial spine count 7.

*Head* (Fig. 40B): As long as wide, the juxta-ocular protuberances very large, the apex in the middle; the vertex is straight, well above the dorsal margin of the eyes.

*Pronotum* (Fig. 47B): Dorsal surface entirely smooth except from a rough texture in the posterior quarter, no defined tubercles. Metazone with very few, small tubercles centrally located.

*Prothoracic Legs*: A shallow femoral pit to accommodate terminal posteroventral tibial spine positioned medial to and exactly between the first two proximal posteroventral spines, and in line with the most distal discoidal spine. Prothoracic tibial posteroventral spines with the first (proximal) smallest and the fourth through sixth of similar length, the second and third are slightly longer (the second much longer than the third). Posterior prothoracic femoral genicular spine much smaller than posteroventral spines, originating distal to the beginning of the genicular lobe.

*Meso- and Metathoracic Legs*: Femora with expanded ventral (posterior) carina, almost a lamellar expansion; dorsal (anterior) carina very pronounced.

*Wings*: Forewings with a widened costal region. Forewings extending to the tip of the abdomen or shorter (depends largely on preservation of specimen). Hindwings pale along the anterior margin and the distal terminus of the discoidal region; the discoidal region broadened.

*Abdomen*: Broad, widening from first segment until the beginning of the distal half (segment 5) when the lateral margins narrow gradually to the terminus, the middle being the broadest region. Tergites without posterolateral tergal projections. Supra-anal plate slightly transverse, and evenly rounded lobe.

##### Liturgusa lichenalis

Gerstaecker, 1889

http://species-id.net/wiki/Liturgusa_lichenalis

Liturgusa lichenalis : Gerstaecker 1889: 52–53; Bertkau 1889: 87; Terra 1995: 54; Ehrmann 2002: 207; Otte and Spearman 2005: 133; Agudelo et al. 2007: 116.
Liturgousa lichenalis : Westwood 1889: 5, 51; Kirby 1904: 271; Caudell 1918: 5; Rehn 1935: 199; Rehn 1954: 179, pl. 1, fig. 1.
Liturgusa annulipes (*partim*): Giglio-Tos 1927: 294; Beier 1935: 11; Jantsch 1999: 48.

###### Type.

Holotype Female. Ernst-Moritz-Arndt-Universität Greifswald, Germany.

###### Type locality.

Peru: Jurimaguas (Lat. -5.900438, Long. -76.125298).

###### Material examined.

*Liturgusa lichenalis* Gerstaecker, 1889

**Table:** 

Sex	Type	Country	Label	Latitude Longitude	Code
Female	Holotype	Peru	Jurimaguas	-5.900438, -76.125298	EMAU
Male	nontype	Peru	Monson Valley, Tingo Maria, XII-11-1954, E.I. Schlinger & E.S. Ross collectors	-9.314153, -76.006745	CAS 001
Male	nontype	Peru	Monson Valley, Tingo Maria, XII-11-1954, E.I. Schlinger & E.S. Ross collectors	-9.314153, -76.006745	CAS 005
Male	nontype	Peru	Monson Valley, Tingo Maria, XII-11-1954, E.I. Schlinger & E.S. Ross collectors	-9.314153, -76.006745	CAS 010
Male	nontype	Peru	Chanchamayo, 04-08-05, E. Cueva	-11.135912, -75.348800	MEKRB 010
Female	nontype	Peru	Dept. Loreto Colonia, Amont Conflt. Rios Zumun & Yahuasyacu, 20-V-20-VI-1978, M. Descamps rec		MNHN 055
Female	nontype	Colombia	Dept. Amazonas, Rio Igara Parana, 30 km aval La Chorrera, VI - VII 1974, M. Descamps rec.	-1.197385, -72.937475	MNHN 081
Male	nontype	Peru	Loreto - Maynas, Picurovacu, apres Sta Clotilde, N. Iquitos, 130 mts - 10.II.2010, S03.37.04 - W 73.15.44, coll. M.Dottax	-3.617778, -73.262222	MNHN 100
Female	nontype	Ecuador	Pr. Sucumbios. Coca/L. Agrio, S.P. de los Cofanes, 415 m. 22/23 IX 1997, 77°52'W, 0°8'S, Amedegnato/Poulain rec.	-0.133333, -77.866667	MNHN 203
Male	nontype	Venezuela	Culebra, N. Duida Territ. Amazonas, April 7-16, 1950, J. Maldonado Capriles Coll.	3.198590, -65.555990	USNM 016; USNM ENT 00873001
Female	nontype	Peru	11°3'S, 75°17'W, X.08, GREENW, C. Schunke	-11.050000, -75.283333	USNM 059; USNM ENT 00873002
Female	nontype	Peru	Madre de Dios, Rio Tambopata Res., 30 km (air) SW Pto. Maldonado, 290 m, 12°50'S, 69°17'W; Smithsonian Institution Canopy Fogging Project, T.L. Erwin et al., colls. 02 Mar 84, 03/02	-12.833333, -69.283333	USNM 071; USNM ENT 00873039
Female	nontype	Peru	Madre de Dios, Rio Tambopata Res., 30 km (air) SW Pto. Maldonado, 290 m, 12°50'S, 69°17'W; Smithsonian Institution Canopy Fogging Project, T.L. Erwin et al., colls. 01 Mar 82, 01/034/02	-12.833333, -69.283333	USNM 072; USNM ENT 00873040
Female	nontype	Peru	Madre de Dios, Rio Tambopata Res., 30 km (air) SW Pto. Maldonado, 290 m, 12°50'S, 69°17'W; Smithsonian Institution Canopy Fogging Project, T.L. Erwin et al., colls. 10Sept84, 02/02/055	-12.833333, -69.283333	USNM 073; USNM ENT 00873041
Male	nontype	Peru	Madre de Dios, Rio Tambopata Res., 30 km (air) SW Pto. Maldonado, 290 m, 12°50'S, 69°17'W; Smithsonian Institution Canopy Fogging Project, T.L. Erwin et al., colls. 07Nov83, 01/02/71	-12.833333, -69.283333	USNM 074; USNM ENT 00873042
Male	nontype	Peru	Madre de Dios, Rio Tambopata Res., 30 km (air) SW Pto. Maldonado, 290 m, 12°50'S, 69°17'W; Smithsonian Institution Canopy Fogging Project, T.L. Erwin et al., colls. 10Sept84, 02/03/114	-12.833333, -69.283333	USNM 075; USNM ENT 00873043
Male	nontype	Peru	Madre de Dios, Rio Tambopata Res., 30 km (air) SW Pto. Maldonado, 290 m, 12°50'S, 69°17'W; Smithsonian Institution Canopy Fogging Project, T.L. Erwin et al., colls. 30Apr84, 03/03/089	-12.833333, -69.283333	USNM 076; USNM ENT 00873044
Male	nontype	Peru	Madre de Dios, Rio Tambopata Res., 30 km (air) SW Pto. Maldonado, 290 m, 12°50'S, 69°17'W; Smithsonian Institution Canopy Fogging Project, T.L. Erwin et al., colls. 02Mar84, 03/02/056	-12.833333, -69.283333	USNM 077; USNM ENT 00873045
Male	nontype	Peru	Madre de Dios, Rio Tambopata Res., 30 km (air) SW Pto. Maldonado, 290 m, 12°50'S, 69°17'W; Smithsonian Institution Canopy Fogging Project, T.L. Erwin et al., colls. 14Sept84, 01/03/120	-12.833333, -69.283333	USNM 078; USNM ENT 00873046
Male	nontype	Peru	Madre de Dios, Rio Tambopata Res., 30 km (air) SW Pto. Maldonado, 290 m, 12°50'S, 69°17'W; Smithsonian Institution Canopy Fogging Project, T.L. Erwin et al., colls. 04Mar84	-12.833333, -69.283333	USNM 079; USNM ENT 00873047
Male	nontype	Peru	Madre de Dios, Rio Tambopata Res., 12°50'S, 69°17'W; Coll: G.J. Svenson 2005	-12.833333, -69.283333	GSMC000259
Male	nontype	Ecuador	Pastaza; Ashuara, Rio Macuma, 10km from Rio Morona, 300m, VII:7-16:1971, leg. B. Malkin	-2.753512, -77.444899	FMNH 001
Male	nontype	Peru	Loreto; Yagua, Indian village, head-waters Rio Loreto-Yacu, IV:29-V:1:1970, leg. B. Malkin	-3.894566, -71.971608	FMNH 002
Female	nontype	Peru	Loreto; Ucayali R., Yarina Cocha, II-27-1956, leg. Peter Hocking	-5.509011, -74.377603	FMNH 007

###### Taxonomic history.

Described in 1889 by Gerstaecker, the species has been largely ignored other than inclusion in taxonomic lists and some regional studies. Giglio-Tos (1927) considered *Liturgusa lichenalis* as the synonym of *Fuga annulipes*, but this treatment is erroneous as it is apparent that no previous work had an accurate concept of *Fuga annulipes*. Rehn (1935) corrected this erroneous synonym by comparing the types of both species.

###### Diagnosis.

Almost identical to *Liturgusa cayennensis*, but primarily distributed in the western Amazon basin rather than in the Guyanas in the northeastern coastal region of South America. In addition, the abdomens of males and females are broader than *Liturgusa cayennensis* and have posterolateral tergal projections in the distal half. Another distinguishing external character for *Liturgusa lichenalis* comparison to *Liturgusa cayennensis* is that the first segment of the mesotarsi is obviously shorter than the remaining segments combined while this segment is the same length as the remaining segments combined in *Liturgusa cayennensis*.

###### Description.

**Figure 5.**
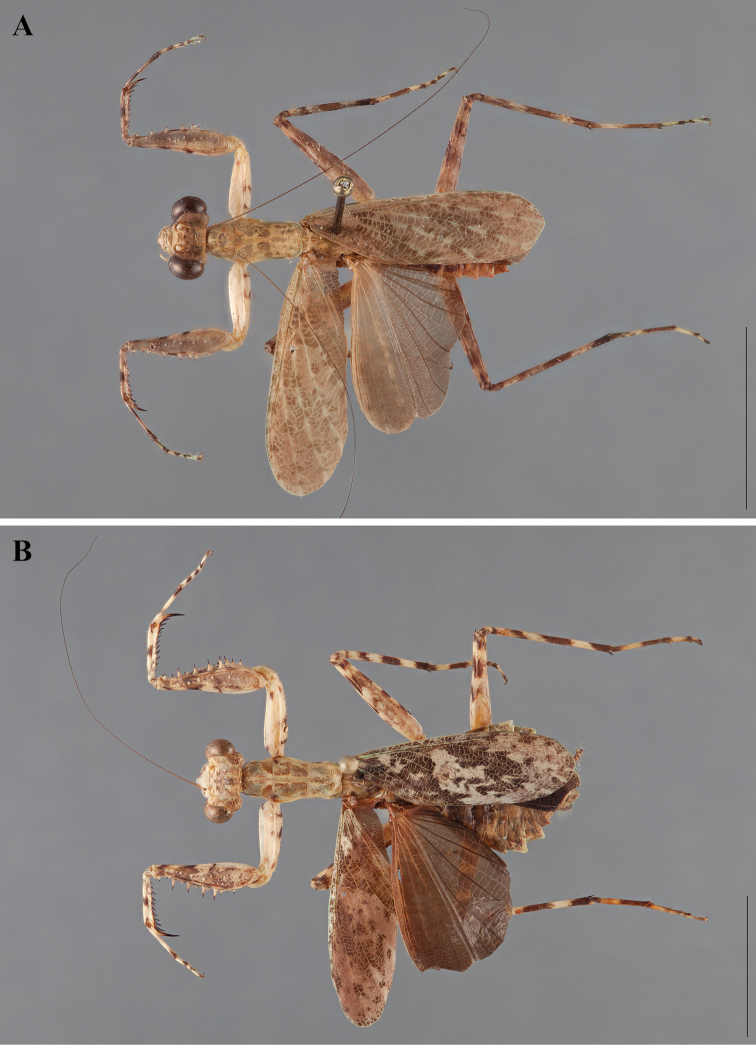
*Liturgusa lichenalis* Gerstaecker, 1889, dorsal habitus: **A** male from Tingo Maria, Peru (CAS 010) **B** female from Ecuador (MNHN 203).

**Male.** (Fig. 5A) N=12: Body length 18.59–24.79 (21.45); forewing length 12.62–16.54 (14.17); hindwing length 10.27–11.75 (11.01); pronotum length 5.03–6.59 (5.74); prozone length 1.55–2.07 (1.78); pronotum width 2.33–3.18 (2.68); pronotum narrow width 1.78–2.47 (2.07); head width 4.54–5.75 (5.03); head vertex to clypeus 1.81–2.03 (1.91); frons width 1.66–1.93 (1.78); frons height 0.59–0.75 (0.67); prothoracic femur length 5.60–7.14 (6.19); mesothoracic femur length 6.37–8.50 (7.38); mesothoracic tibia length 4.78–6.27 (5.52); mesothoracic tarsus length 4.77–6.25 (5.32); metathoracic femur length 6.71–8.92 (7.55); metathoracic tibia length 6.82–8.68 (7.64); metathoracic tarsus length 6.41–8.40 (7.34); pronotal elongation measure 0.30–0.33 (0.31); pronotal shape measure 0.44–0.49 (0.47); head shape measure 0.38–0.40 (0.39); frons shape measure 0.35–0.40 (0.38); anteroventral femoral spine count 13–17 (15); anteroventral tibial spine count 10; posteroventral tibial spine count 7.

*Head* (Fig. 40C): Transverse, the juxta-ocular protuberances small, but pronounced, the apex slightly in the lateral half; the vertex is straight, even with the dorsal margin of the eyes. Frontal suture with a medial carina forming a continuous arc, most pronounced medially, the region just ventral depressed. Ocelli small and protruding on small cuticular mounds, but the region between all three slightly raised; the lateral ocelli oriented outward. The carina on the frons present, but not highly pronounced, the medial region just ventral to the carina depressed. Clypeus transverse, the upper margin convex, the lower margin slightly concave; the central carina strongly pronounced and straight. Antennae pale at the base, the flagellum fading gradually to dark brown around a third of the way from the base. Black band extending straight over the medial carina of the frontal suture, the medial portion of the carina pale; a branch of the black band extends ventro-laterally between the eye and antennal insertion at a forty five degree angle; area around parietal sutures pale or dark brown. Lower region of frons darkened; the clypeus is pale; the mandibles and labrum mostly pale, but with some brown marks; the vertex and juxta-ocular protuberances mostly pale; the area immediately adjacent to lateral ocelli black. Palpi are pale.

*Pronotum* (Fig. 47C): Short and squat with a poorly defined supra-coxal bulge; dorsal surface entirely smooth or with few blunt tubercles in the posterior half. Prozone squat with near parallel margins before tapering anteriorly to a rounded anterior margin; the margins smooth. Metazone with barely concave lateral margins, mostly tapering consistently from the supra-coxal bulge posteriorly until a very slight widening in the posterior quarter leading to the posterior margin; margins smooth; posterior margin with a slight medial emargination; the dorsal surface of the posterior half of the metazone depressed; two small and elongate bulges barely present on the dorsal surface near the posterior margin and positioned laterally, sometimes absent. Pale with strong black marks across the surface, two prominent black marks laterally just posterior to the supra-coxal sulcus.

*Prothoracic Legs*: Femur squat and robust with a slightly concave dorsal margin; strongly defined pale to dark banding on posterior (external) surface; anterior (internal) surface with a very thin black band running medially from the base to terminus, a small black mark dorsal to the band at the midpoint, and a dark mark dorsal to the band near the femoral brush; the ventral surface pale. Posterior surface of femur with few tubercles. A shallow femoral pit to accommodate terminal posteroventral tibial spine positioned slightly distal to the first proximal posteroventral spine and in line with the most distal discoidal spine; pit is pigmented darkly or pale. Posterior prothoracic femoral genicular spine much smaller than posteroventral spines, originating distal to the beginning of the genicular lobe. Prothoracic tibial posteroventral spines with the first (proximal) smallest and the fourth through sixth of similar length, the second and third are slightly longer. Prothoracic coxae smooth, the anterior surface pale.

*Meso- and Metathoracic Legs*: Femora with ventral (posterior) carina; dorsal (anterior) carina pronounced. Mesotarsi with first segment shorter than the remaining segments combined.

*Wings*: Forewings mottled with brown, pale and greenish coloration; the costal region without strongly defined banding, green and brown proximally with some low contrasting bands developing distally; veins not contrasting from surrounding coloration; two pale spots are positioned in the proximal quarter of the discoidal region just posterior to the first radial vein; a large pale spot is positioned centrally. Forewings often, but not always asymmetrically colored; one being mottled as described the other is darkened significantly with a rust tone, the mottled pattern still visible; extending just beyond or as long as the abdomen. Hindwings with discoidal region opaque and colored brown or rust; the anal region smoky and translucent; the terminus of the discoidal region projecting beyond the distal margin of anal region, the wing appearing slightly elongate.

*Abdomen*: Broad, widening until the fifth tergite before a gradual posterior narrowing; a smooth, brown and black colored dorsal surface. Tergites with small posterolateral tergal projections beginning on the sixth segment. Supra-anal plate slightly transverse, margins tapering gradually to a broadly rounded terminus. Subgenital plate irregularly rounded and without styli.

*Genital Complex* (Fig. 51B.1–B.4): The main body of ventral left sclerite (L4A) with rounded terminus, but with a short, laterally positioned distal process (pda) that is rounded and sometimes, but not always, projecting at an angle towards the medial axis of the L4A that can create a strongly angled transition from the terminal margin of the L4A to the medial margin of the pda; sometimes a depression on the lateral half is present. The apofisis falloid (afa) of the main body of dorsal left sclerite (L4B) very short, broad, tapered to the point and heavily sclerotized, not curved; the apical process (paa) short, cylindrical and curved, the terminus with a rounded end. The right dorsal phallomere (fda) of the first sclerite of right phallomere (R1) tapers to a broad, rounded, membranous terminus, the lateral margin often folded over; the ventral plate (pia) long, broadened proximally and with strongly defined grooves; the ventral process (pva) smooth and tapering to a point distally, one edge straight and the other convex, tooth-like in appearance, but the proximal half c-shaped.

###### Redescription.

**Female.** (Figs 4C, 5B) N=8: Body length 25.68–32.17 (28.36); forewing length 16.25–19.99 (18.46); hindwing length 13.46–16.50 (14.96); pronotum length 6.68–8.26 (7.48); prozone length 2.12–2.64 (2.36); pronotum width 3.29–3.99 (3.59); pronotum narrow width 2.49–2.82 (2.71); head width 5.96–7.01 (6.48); head vertex to clypeus 2.55–3.05 (2.81); frons width 2.36–2.87 (2.62); frons height 0.93–1.13 (1.01); prothoracic femur length 7.40–9.15 (8.11); mesothoracic femur length 8.30–9.76 (8.85); mesothoracic tibia length 6.39–8.13 (7.04); mesothoracic tarsus length 5.67–6.75 (6.19); metathoracic femur length 8.18–9.91 (8.75); metathoracic tibia length 8.59–11.05 (9.60); metathoracic tarsus length 7.73–9.35 (8.51); pronotal elongation measure 0.31–0.33 (0.32); pronotal shape measure 0.47–0.51 (0.48); head shape measure 0.42–0.44 (0.43); frons shape measure 0.36–0.41 (0.39); anteroventral femoral spine count 15–16 (15); anteroventral tibial spine count 10; posteroventral tibial spine count 7.

*Head* (Fig. 40D): As long as wide, the juxta-ocular protuberances very large, the apex in the middle; the vertex is slightly concave, above the dorsal margin of the eyes. Ocelli small and protruding on a continuous carina connecting the three and extending slightly laterally; region between all three raised slightly. Black marking present over the medial carina of the frontal suture; the vertex and juxta-ocular protuberances with dark markings.

*Pronotum* (Fig. 47D): Metazone with barely concave lateral margins, tapering rapidly in the first third from the supra-coxal bulge, the middle third near parallel before widening to the posterior margin; margins smooth or with few small tubercles; posterior margin with a medial emargination; few small tubercles present.

*Prothoracic Legs*: Anterior (internal) surface of femur with a very thin black band running medially from the base to terminus that is often interrupted, a small black mark dorsal to the band at the midpoint, and a dark mark dorsal to the band near the femoral brush.

*Meso- and Metathoracic Legs*: As described for males.

*Wings*: Forewings mottled with contrasting brown, pale and greenish coloration; veins pale brown. Forewings often, but not always asymmetrically colored; one being mottled as described the other is darkened with a rust or black tone, the mottled pattern still visible; extending to the terminus of the abdomen. Hindwings with discoidal region opaque and colored brown, rust or dark yellow; the anal region smoky and translucent.

*Abdomen*: Very broad, widening until the fifth tergite before a gradual posterior narrowing, the abdomen almost circular; a smooth, brown and black colored dorsal surface. Tergites with large posterolateral tergal projections beginning on the fourth segment. Supra-anal plate slightly transverse, margins tapering gradually to a rounded terminus.

##### Liturgusa guyanensis

La Greca, 1939

http://species-id.net/wiki/Liturgusa_guyanensis

Liturgusa guyanensis : La Greca 1939: 2–5, fig. 1; Terra 1995: 54; Ehrmann 2002: 207; Otte and Spearman 2005: 133; Agudelo et al. 2007: 116.
Liturgusa guyannensis : Jantsch 1999: 48.

###### Type.

LOST, but listed repository Natural History Museum Zoological Section 'La Specola', University of Florence.

###### Original type locality.

Guyana, Babooncamp (Demerara), October 1931.

###### Neotype.

Male. California Academy of Sciences, San Francisco, CA, USA.

###### Neotype locality.

Guyana: Iwokrama, Forest Research Station, 1 km N. Kurupukari, 14-19 January 1996, canopy fog sample of Mora tree, W. Tschinkel coll. (Lat. 4.672093, Long. -58.685606).

###### Material examined.

*Liturgusa guyanensis* La Greca, 1939.

**Table:** 

Sex	Type	Country	Label	Latitude Longitude	Code
Male	Neotype	Guyana	Iwokrama, Forest Research Station, 1 km N. Kurupukari, 14-19 January 1996, canopy fog sample of Mora tree, W. Tschinkel coll.	4.672093, -58.685606	CAS 021
Female	nontype	Brazil	Manaos, II-III-43, Thomas Gilliard	-3.003866, -59.961947	AMNH 027
Male	nontype	Guyana	Iwokrama, Forest Research Station, 1 km N. Kurupukari, 14-19 January 1996, canopy fog sample of Mora tree, W. Tschinkel coll.	4.672093, -58.685606	CAS 017
Nymph	nontype	Brazil	Amazonas, Rio Taruma Mirim, 20 km nw Manaus, 02 Mar 1979, 02°53'S, 060°07'W, Montgomery, Erwin, Schimmel, Krischik, Date, Bacon colls., Black water innundation forest canopy fogged with Pyrethrum, Sample # 48.	-2.883333, -60.116667	USNM 010; USNM ENT 00873003

###### Taxonomic history.

Described in 1939 by Marcello La Greca, *Liturgusa guyanensis* was only subsequently treated in taxon lists and has received no revisionary attention. La Greca only included the type locality, but no repository for the type. It is presumed that the specimen remains in an Italian collection. In addition, the species was cited in subsequent works with the repository listed as the Natural History Museum Zoological Section 'La Specola', University of Florence. Therefore, Luca Bartolozzi was contact in hopes of locating the specimen. After an extensive search it was not located in Florence. Luca Bartolozzi contacted museums in Genoa, Milan and Rome looking for the specimen, but nothing was located in these collections. After consulting with Luca Picciau, of the Museo Regionale di Scienze Naturali, Torino, in search of the type specimen of *Liturgusa charpentieri*, described and deposited there by Giglio-Tos (1927), it was learned the specimen was loaned to La Greca, but after his death his entire collection, presumably including loans, was sold to the Museo Civico di Storia Naturale, Milan. Chasing this lead, Fabrizio Rigato and Michele Zilioli, both of the Museo Civico di Storia Naturale, Milan, were contacted, but neither the type of *Liturgusa charpentieri* nor *Liturgusa guyanensis* were located in the collection. In addition, the eminent mantodean systematist, Prof. Francesco Lombardo of the Università degli Studi di Catania, was consulted throughout this search, but he as well was not able to locate the type. Therefore, in satisfying Article 75.3 of the International Code of Zoological Nomenclature, a specimen has been located that matches the original description and illustrations provided by La Greca (1939) and represents this distinct species within *Liturgusa*. Although La Greca’s type was female and the designated neotype is male, a female specimen has also been located and is treated herein, which demonstrates a match with the original name-bearing type. A male is designated as the neotype herein as a measure of consistency across the entire genus, which now includes holotype descriptions of male genital structures. Finally, the neotype is not from the original type locality, but from just south within Guyana and still on the eastern side of the Guyana Shield, within the same elevational range, forest type, and climate conditions.

###### Diagnosis.

Similar to *Liturgusa cayennensis* and living in sympatry with at least some populations of the species, *Liturgusa guyanensis* have a far more sculpted pronotum, a unique vertex of the head (straight with lateral depressions near the parietal sutures), and evenly mottled forewings (without the large pale or whitish regions that are obviously present on *Liturgusa cayennensis* and *Liturgusa lichenalis*).

###### Description.

**Figure 6.**
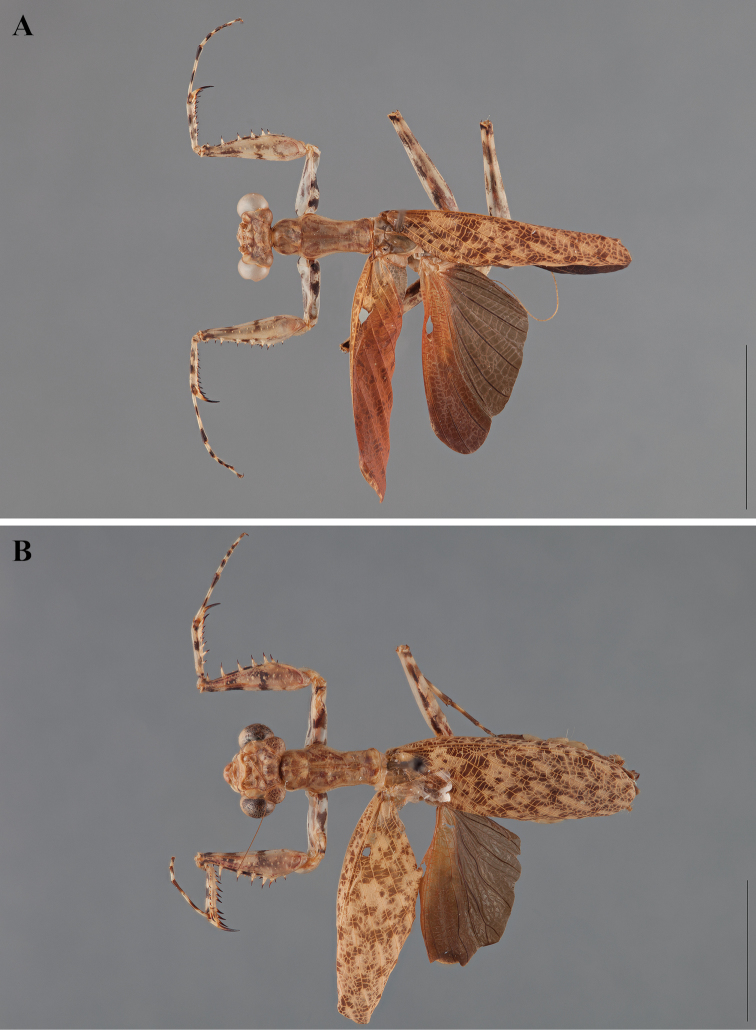
*Liturgusa guyanensis* La Greca, 1939, dorsal habitus: **A** neotype male from Iwokrama, Guyana (CAS 021) **B** female from Manaus, Brazil (AMNH 027).

**Male.** (Fig. 6A) N=1: Body length 23.28; forewing length 15.42; hindwing length 12.31; pronotum length 6.19; prozone length 1.85; pronotum width 2.83; pronotum narrow width 1.92; head width 5.46; head vertex to clypeus 2.20; frons width 2.07; frons height 0.82; prothoracic femur length 6.41; mesothoracic femur length 7.69; metathoracic femur length 7.57; pronotal elongation measure 0.30; pronotal shape measure 0.46; head shape measure 0.40; frons shape measure 0.39; anteroventral femoral spine count 14; anteroventral tibial spine count 10; posteroventral tibial spine count 7.

*Head* (Fig. 40E): Slightly transverse, the juxta-ocular protuberances moderately pronounced, the apex in the lateral half; the vertex is convex, slightly higher than the dorsal margin of the eyes. Frontal suture with a medial carina forming a continuous arc, the region just ventral depressed for most of the length. Ocelli small and protruding prominently on small cuticular mounds; the lateral ocelli oriented outward. The carina on the frons pronounced, the medial region just ventral to the carina depressed and sloped ventrally. Clypeus transverse, the upper margin convex, the lower margin straight; the central carina strongly pronounced and straight. Antennae pale at the base, the flagellum absent on specimen. The vertex and juxta-ocular protuberances pale and with brown speckled markings; the area immediately adjacent to lateral ocelli black; the ventrolateral margins of the frons with a dark brown margin. Palpi are pale.

*Pronotum* (Fig. 47E): Short and squat with a defined supra-coxal bulge; dorsal surface with disperse, small tubercles. Prozone squat with convex margins widening anterior to supra-coxal bulge before narrowing to the anterior margin; the margins smooth. Metazone with sweeping concave lateral margins, the nadir at the three quarter point from the supra-coxal bulge, then widening slightly to the posterior margin; margins with small, disperse tubercles; posterior margin with a slight medial emargination, almost straight; the dorsal surface of the posterior third of the metazone depressed; tubercles more common in the posterior half. Pale with strong black marks across the surface, two prominent black marks laterally just anterior to the supra-coxal sulcus in the prozone.

*Prothoracic Legs*: Femur squat and robust with a near straight dorsal margin; strongly defined pale to dark banding on posterior (external) surface; anterior (internal) surface with a very thin black band running medially from the base to terminus, a black mark dorsal to the band at the midpoint and some thickening of the line near the femoral brush; the ventral surface pale. Posterior surface of femur with few tubercles. A shallow femoral pit to accommodate terminal posteroventral tibial spine positioned just distal to the first most proximal posteroventral spine and in line with the most distal discoidal spine; pit is pigmented darkly. Posterior prothoracic femoral genicular spine much smaller than posteroventral spines, originating distal to the beginning of the genicular lobe. Prothoracic tibial posteroventral spines with the first (proximal) smallest and the fourth through sixth of similar length, the second and third are slightly longer. Prothoracic coxae smooth, the anterior surface with a very small, black mark in the proximal half and adjacent to the medial line.

*Meso- and Metathoracic Legs*: Femora with ventral (posterior) carina; dorsal (anterior) carina pronounced. Mesotarsi with first segment slightly longer than the remaining segments combined.

*Wings*: Forewings evenly mottled with dark and light brown coloration; the costal region without strongly defined banding, mostly matching the color patterns of the discoidal region; veins are pale, contrasting with the cell colors. Forewings asymmetrically colored; one being mottled as described the other is darkened significantly with a rust tone, the mottled pattern still visible; extending just beyond the abdomen. The discoidal region of the hindwings opaque, a pale rust color proximally that fades to a dark, rusty opaque color in the distal half; the anal region of the hindwing smoky and translucent; the terminus of the discoidal region projecting beyond the distal margin of anal region, the wing appearing elongate.

*Abdomen*: Broad, widening until the fifth tergite before a gradual posterior narrowing; a smooth, brown and black colored dorsal surface. Tergites without posterolateral tergal projections. Supra-anal plate transverse, a broadly rounded terminus with a medial emargination. Subgenital plate irregularly rounded and without styli.

*Genital Complex* (Fig. 51C.1): The main body of ventral left sclerite (L4A) with a convex terminal margin that tapers to a medially positioned, blunt point that is well sclerotized, but lacking a distal process (pda). The apofisis falloid (afa) of the main body of dorsal left sclerite (L4B) elongate, slender and curved, terminating in a blunt, but narrow point; the apical process (paa) short, cylindrical and curved, the terminus with a rounded end. The right dorsal phallomere (fda) of the first sclerite of right phallomere (R1) tapers to a rounded, membranous terminus; the ventral plate (pia) long with strongly defined grooves; the ventral process (pva) tooth-like and curved at the proximal base, the distal tip narrowing with a rapid constriction towards the end.

###### Redescription.

**Female.** (Fig. 6B) N=1: Body length 26.83; forewing length 18.06; hindwing length 14.03; pronotum length 7.10; prozone length 2.09; pronotum width 3.50; pronotum narrow width 2.32; head width 6.63; head vertex to clypeus 2.97; frons width 2.79; frons height 1.11; prothoracic femur length 7.61; mesothoracic femur length 8.01; mesothoracic tibia length 5.92; mesothoracic tarsus length 5.80; pronotal elongation measure 0.29; pronotal shape measure 0.49; head shape measure 0.45; frons shape measure 0.40; anteroventral femoral spine count 14; anteroventral tibial spine count 10; posteroventral tibial spine count 7.

*Head* (Fig. 40F): Approximately as broad as wide, the juxta-ocular protuberances very large, the apex in the middle; the vertex is straight, but with two depressions just medial to the parietal sutures, higher than the dorsal margin of the eyes. Ocelli very small and protruding prominently on small cuticular mounds, region between ocelli, ventral to the frontal suture and dorsal of the frons is depressed. Clypeus transverse, the upper margin convex, the lower margin rounded. Antennae pale at the base, the flagellum fading to brown gradually.

*Pronotum* (Fig. 47F): Prozone squat and broad with convex margins widening prominently anterior to supra-coxal bulge before narrowing quickly to the anterior margin; the margins with small tubercles. Metazone with concave lateral margins, the nadir at the three quarter point from the supra-coxal bulge, but a slight bulge in the posterior half present that pushes the margins laterally and interrupts the continuity of the concave margins; margins with small, numerous tubercles.

*Prothoracic Legs*: Femur with anterior (internal) surface with a black band running medially from the base to terminus, a black mark dorsal to the band at the midpoint and some thickening of the line near the femoral brush, but the overall band may be interrupted medially. Prothoracic tibial posteroventral spines with the first (proximal) smallest and the fourth through sixth of similar length, the second and third are longer.

*Meso- and Metathoracic Legs*: Mesotarsi with first segment shorter than the remaining segments combined.

*Wings*: Costal region of forewing widened. Forewings symmetrically colored; extending the length of the abdomen. The terminus of the discoidal region of the hindwing projecting slightly beyond the distal margin of anal region, the wing appearing slightly elongate.

*Abdomen*: Broad, widening from first segment until the beginning of the distal half (segment 5) when the lateral margins narrow gradually to the terminus, the middle being the broadest region. Tergites without posterolateral tergal projections. Supra-anal plate slightly transverse, and evenly rounded lobe.

##### Liturgusa neblina sp. n.

http://zoobank.org/C07DA375-BE0F-48A8-96B4-711F19BA6791

http://species-id.net/wiki/Liturgusa_neblina

###### Type.

Holotype Female, pinned. National Museum of Natural History, Smithsonian Institution, Washington, DC, USA.

###### Type Locality.

Venezuela: T.F. Amaz., Cerro de la Neblina, Basecamp, 140 m, 0°50'N, 66°10'W, 19 February 1985, Pyrethrin fogging of vine tangle: canopy of floodplain forest along Rio Baria; R. Cocroft & W. Steiner. (Lat. 0.833333, Long. -66.166667).

###### Material examined.

*Liturgusa neblina* sp. n.

**Table:** 

Sex	Type	Country	Label	Latitude Longitude	Code
Female	Holotype	Venezuela	T.F. Amaz., Cerro de la Neblina, Basecamp, 140 m, 0°50'N, 66°10'W, 19 February 1985, Pyrethrin fogging of vine tangle: canopy of flood plain forest aling Rio Baria; R. Cocroft & W. Steiner.	0.833333, -66.166667	USNM 006; USNM ENT 00873998

###### Diagnosis.

Most similar in general appearance to *Liturgusa cayennensis* and *Liturgusa lichenalis*. The type locality overlaps with the range of *Liturgusa lichenalis*, but *Liturgusa neblina* is distinct in a few ways. First the hindwings are yellow-orange in the discoidal region with either a rapid or slow fade to a hyaline or translucent brown in the anal region. The pronotum is short and squat as are the other species within the Cayennensis Group, but the metazone is more constricted. The forewings are evenly mottled with green and brown with regular pale marks in the distal half.

###### Description.

**Figure 7.**
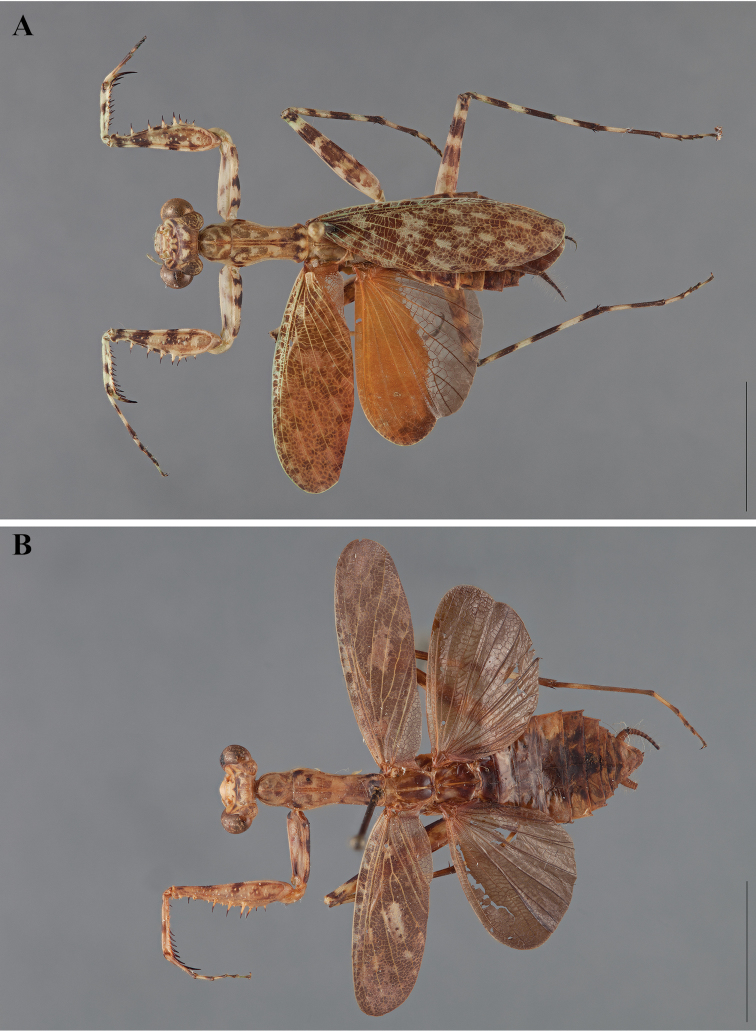
*Liturgusa*, dorsal habitus: **A** *Liturgusa neblina* sp. n., holotype female from Cerro de la Neblina, Venezuela (USNM 006) **B** *Liturgusa maroni* sp. n., holotype female from St. Laurent du Maroni, French Guiana (MNHN 019).

**Female.** (Fig. 7A) N=1: Body length 29.75; forewing length 19.54; hindwing length 16.35; pronotum length 8.15; prozone length 2.46; pronotum width 3.68; pronotum narrow width 2.46; head width 6.90; head vertex to clypeus 2.88; frons width 2.78; frons height 1.10; prothoracic femur length 8.45; mesothoracic femur length 9.50; mesothoracic tibia length 7.50; mesothoracic tarsus length 6.48; metathoracic femur length 9.42; metathoracic tibia length 10.72; metathoracic tarsus length 9.13; pronotal elongation measure 0.30; pronotal shape measure 0.45; head shape measure 0.42; frons shape measure 0.41; anteroventral femoral spine count 14; anteroventral tibial spine count 10; posteroventral tibial spine count 7.

*Head* (Fig. 40G): As long as broad, the juxta-ocular protuberances very large, the apex in the middle; the vertex is slightly concave, above the dorsal margin of the eyes. Frontal suture with a very faint medial carina forming a continuous arc, may be seen primarily because of pale coloration compared to the dark markings above and below. The region ventral to the frontal suture depressed, gradually sloping higher to the central ocellus. Ocelli small and protruding on a carina that connects all three and extends laterally a short distance; the lateral ocelli oriented outward; area just ventral to central ocellus depressed (upper region of frons). The carina of the frons pronounced, the medial region just ventral to the carina depressed, sloped ventrally. Clypeus slightly transverse, the upper margin slightly convex, the lower margin concave; the central carina strongly pronounced and straight. Antennae is pale at the base, the flagellum absent from specimen. Curved, black band extending over the frontal suture, the carina pale; projections from the band extend dorsally from the middle, ventrally toward the central ocellus, and surrounding the lateral ocelli and extending laterally above the antennal insertion. Lower region of frons darkened; the clypeus is pale; the mandibles mostly pale, but with some brown marks distally; the labrum pale but with two laterally position black marks near the dorsal margin; the vertex and juxta-ocular protuberances mottled with pale and brown. Palpi are pale.

*Pronotum* (Fig. 47G): Short and squat with a moderately defined supra-coxal bulge; dorsal surface with a few blunt tubercles in the posterior half. Prozone squat with tapering margins anteriorly to a rounded margin; the margins smooth. Metazone with posteriorly tapering margins until the posterior half where the margins are parallel before widening again prior to the posterior margin; posterior two thirds of the margins with small tubercles; posterior margin with a slight medial emargination; the dorsal surface of the posterior half of the metazone depressed. Pale with strong black marks across the surface, two prominent black marks laterally just anterior to the supra-coxal sulcus.

*Prothoracic Legs*: Femur squat and robust with a slightly concave dorsal margin; strongly defined pale to dark banding on posterior (external) surface; anterior (internal) surface mostly pale, but with a faint dark mark medially and the distal third with a black band running along the medial line with an expanded region near the femoral brush; the ventral surface pale. Posterior surface of femur with few tubercles. A shallow femoral pit to accommodate terminal posteroventral tibial spine positioned medial to the two most proximal posteroventral spines and in line with the most distal discoidal spine; pit is pigmented brown. Posterior prothoracic femoral genicular spine much smaller than posteroventral spines, originating distal to the beginning of the genicular lobe. Prothoracic tibial posteroventral spines with the first (proximal) smallest and the fourth through sixth of similar length, the second and third are longer, but the second is very long. Prothoracic coxae smooth, the anterior surface pale.

*Meso- and Metathoracic Legs*: Femora with ventral (posterior) carina; dorsal (anterior) carina pronounced. Mesotarsi with first segment shorter than the remaining segments combined.

*Wings*: Forewings evenly mottled with brown and green, without large contrasting regions of pale marks; the costal region without strongly defined banding, green and brown mottling; veins green and contrasting from surrounding coloration; two pale spots are positioned in the proximal quarter of the discoidal region just posterior to the first radial vein. Forewings asymmetrically colored, one being mottled as described the other is darkened significantly with a rust tone, the mottled pattern still visible; extending slightly beyond the abdomen. Hindwings with discoidal region opaque and colored yellow or orange, darkening distally; the anal region with a very narrow anterior margin colored as in the discoidal region, hyaline otherwise; the terminus of the discoidal region projecting beyond the distal margin of anal region, the wing appearing slightly elongate, but still broad.

*Abdomen*: Broad, widening until the fifth tergite before a gradual posterior narrowing; a smooth, brown and black colored dorsal surface. Tergites without posterolateral tergal projections. Supra-anal plate slightly transverse, margins tapering gradually to a rounded terminus.

###### Etymology.

A noun in apposition, *Liturgusa neblina* is from the Cerro de la Neblina, the geological formation giving rise to the Pico de Neblina, a tepui located in southern Venezuela near the border with Brazil. The only known female was collected at the basecamp for Pico de Neblina.

#### Maya Group

##### Liturgusa bororum sp. n.

http://zoobank.org/B4405064-2641-45FD-8EA6-682B884CDA98

http://species-id.net/wiki/Liturgusa_bororum

###### Type.

Holotype Male, pinned. Muséum national d’Histoire naturelle, Paris, France.

###### Type locality.

Peru: Loreto, Brillo Nuevo, Reg. de l‘Ampiyacu, en av. confl. des rios Zumun et Yahuasyacu, L. Desutter Rec.; Paro.Q; Sans (5A. Apres Aband.) Chasse Jour 9-XI-1985 (Lat. -3.335174, Long. -71.816805).

###### Material examined.

*Liturgusa bororum* sp. n.

**Table:** 

Sex	Type	Country	Label	Latitude Longitude	Code
Male	Holotype	Peru	Loreto, Brillo Nuevo, Reg. de l‘Ampiyacu, en av. confl. des rios Zumun et Yahuasyacu, L. Desutter Rec.; Paro.Q; Sans (5A. Apres Aband.) Chasse Jour 9-XI-1985	-3.335174, -71.816805	MNHN 038
Female	Allotype	Peru	Loreto Province, Puerto Almendra, -3.830525, -73.374, 108 m, 19-21 February 2013, Coll: G.J. Svenson, Tissue 032	-3.830525, -73.374000	GSMC004008
Male	Paratype	Peru	Loreto Province, Madre Selva Biological Research Station, -3.62096, -72.24744, 10-17 February 2013, Coll: G.J. Svenson - Tissue 005	-3.620960, -72.247440	GSMC004052
Female	Paratype	Brazil	Benjamin Constant, IX 1979, AM, B. Silva rec., C. Seabia leg.	-4.383010, -70.042251	MNHN 051

###### Natural history.

Only three individuals (one escaped) were observed in two locations in the Loreto Province, Peru. Two females, both were found less than one meter from the ground on small to medium diameter, smooth bark trees with patches of moss. The female that escaped ran into the herbaceous vegetation surrounding the tree at the base and disappeared. The collected female was easily corralled low on the tree. The male was found living in sympatry with *Liturgusa algorei*, *Liturgusa krattorum*, and *Liturgusa maya* at the Madre Selva Biological Research Station in the Loreto Province, Peru.

###### Diagnosis.

The second smallest species in *Liturgusa*, second only to *Liturgusa cura*, *Liturgusa bororum* can be distinguished from *Liturgusa cura* easily by the presence of dark pigmentation across the ventral surface of the prothoracic femora in males and two markings in females; males of *Liturgusa cura* have only two small marks and females are entirely devoid of markings. *Liturgusa bororum* is only known from northern Peru and just across the border in Brazil. Can be distinguished from *Liturgusa manausensis* by its evenly rounded posterior margins of the metazone.

###### Description.

**Figure 8.**
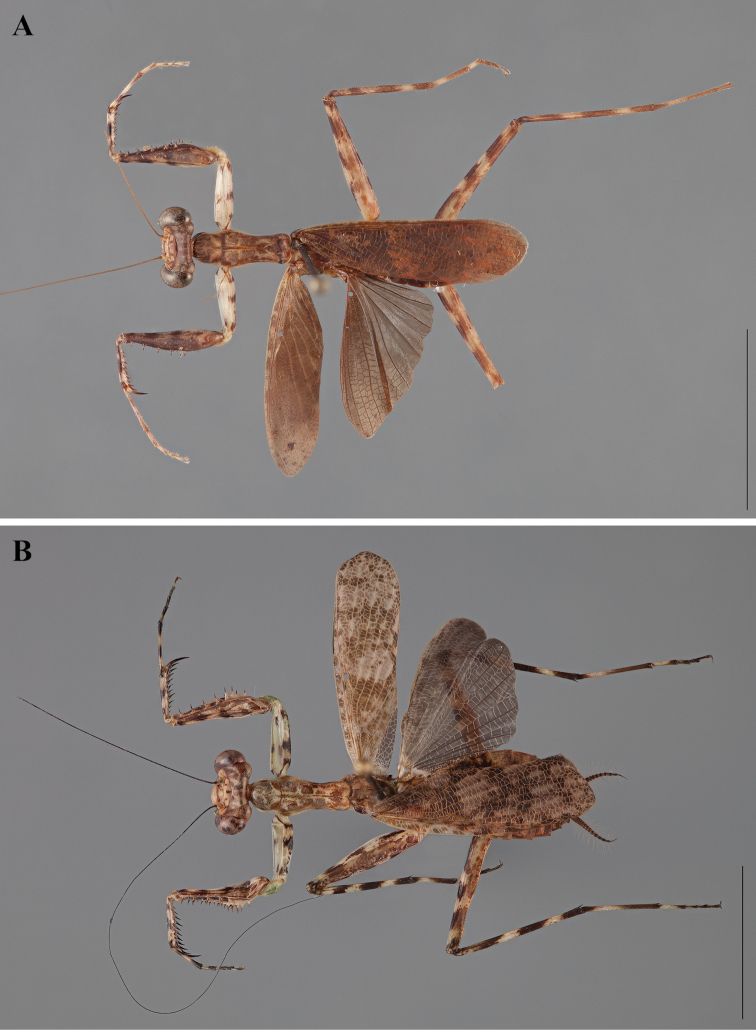
*Liturgusa bororum* sp. n., dorsal habitus: **A** holotype male from Brillo Nuevo, Peru (MNHN 038) **B** allotype female from Loreto, Peru (CLEV GSMC004008).

**Male.** (Fig. 8A) N=2: Body length 19.61–20.30 (19.95); forewing length 12.68–13.63 (13.15); hindwing length 9.88; pronotum length 5.35–5.64 (5.49); prozone length 1.58–1.61 (1.59); pronotum width 2.11–2.25 (2.18); pronotum narrow width 1.46–1.61 (1.53); head width 4.51–4.55 (4.53); head vertex to clypeus 1.80; frons width 1.59–1.71 (1.65); frons height 0.53–0.67 (0.60); prothoracic femur length 5.54–5.99 (5.77); mesothoracic femur length 7.43–7.71 (7.57); mesothoracic tibia length 5.81–6.23 (6.02); mesothoracic tarsus length 5.11; metathoracic femur length 7.27–7.59 (7.43); metathoracic tibia length 7.75–8.54 (8.15); metathoracic tarsus length 8.07; pronotal elongation measure 0.29; pronotal shape measure 0.40; head shape measure 0.40; frons shape measure 0.31–0.42 (0.36); anteroventral femoral spine count 14–15 (14); anteroventral tibial spine count 10; posteroventral tibial spine count 7.

*Head* (Fig. 41A): Transverse, the juxta-ocular protuberances small, the apex in the lateral half; the vertex is nearly straight, but with two slight bulges just medial to each parietal suture; vertex just below the dorsal margin of the eyes. Frontal suture with a slight medial carina forming a continuous arc, the region ventral to the carina, particularly in the middle, is depressed forming a deep groove. Ocelli small and protruding slightly on small cuticular mounds; the central ocellus slightly larger than lateral; than lateral ocelli oriented outward. The carina on the frons not pronounced, but present. Clypeus transverse, the upper margin slightly convex, the lower margin straight or slightly concave; the central, transverse carina pronounced, straight. Antennae scape and pedicel pale, the flagellum light brown or black. Area around frontal suture, vertex and the juxta-ocular protuberances brown with black or darker markings, no distinct transverse band. Lower region of frons dark brown, a small pale region along ventral margin; clypeus pale; the mandibles and labrum pale with darker brown markings. Palpi are pale.

*Pronotum* (Fig. 47H): A little less than three times as long as wide with a moderately defined supra-coxal bulge; dorsal surface mostly smooth, but sometimes with rough patches or small tubercles in the posterior third. Prozone square with slightly convex margins that gradually taper to an evenly rounded anterior margin; margins smooth or with blunt tubercles. Metazone with concave lateral margins; margins smooth or with small tubercles; the dorsal surface of the posterior third of the metazone depressed. Mostly pale or brown with darker brown or black markings across the surface.

*Prothoracic Legs*: Femur normal with a concave dorsal margin; strongly defined pale to dark banding on posterior (external) surface; anterior (internal) surface darkly pigmented, only two pale marks along the dorsal margin; the ventral surface darkly pigmented with brown or black. Posterior surface of femur with few tubercles. A relatively large and shallow femoral pit to accommodate terminal posteroventral tibial spine positioned medial to and just distal to the proximal most posteroventral spine, in line with the most distal discoidal spine; pit is pigmented dark brown. Posterior prothoracic femoral genicular spine slightly smaller than posteroventral spines, originating just proximal of the beginning of the genicular lobe. Prothoracic tibial posteroventral spines with the first (proximal) smallest and the third through sixth of similar length, the second longer. Prothoracic coxae smooth, the anterior surface with a small, black mark medially in the proximal half as well as a small black spot medially towards the distal terminus.

*Meso- and Metathoracic Legs*: Femora with ventral (posterior) carina; dorsal (anterior) carina present. Mesotarsi with first segment shorter or as long as the remaining segments combined.

*Wings*: Forewings mottled with pale, green, and brown coloration; the costal region mostly pale with a few dark marks, no regular banding; vein coloration is pale, but sections match darker surroundings; discoidal region darker proximally, the distal half more pale in overall color dominance. Forewings not asymmetrically colored; extending just beyond the terminus of the abdomen. Hindwings with opaque brown or black discoidal region, darker proximally; the anal region smoky black and translucent; the terminus of the discoidal region projecting far beyond the distal margin of anal region, the wing appearing elongate.

*Abdomen*: Slightly widened, the fifth or sixth tergite the widest region before a gradual posterior narrowing; a smooth, brown and black colored dorsal surface. Tergites without posterolateral tergal projections. Supra-anal plate transverse, evenly tapered terminus. Subgenital plate irregularly rounded and without styli.

*Genital Complex* (Fig. 51D.1): The main body of ventral left sclerite (L4A) asymmetrical, the left margin rounded, leading to a short, blunt nub projecting laterally; the right margin of L4A straight; lacking a distal process (pda). The apofisis falloid (afa) of the main body of dorsal left sclerite (L4B) elongate and robust, one margin being concave and connecting to a prominent subprocess of the L4B, the entire structure c-shaped; the apical process (paa) elongate and thick, the terminus evenly rounded. The right dorsal phallomere (fda) of the first sclerite of right phallomere (R1) tapers to a broadly rounded, membranous terminus; the ventral plate (pia) short and broad with a rough surface and poorly defined grooves; the ventral process (pva) small and c-shaped, the distal terminus tapered to a point.

**Female.** (Fig. 8B) N=2: Body length 24.01–24.69 (24.35); forewing length 14.54–15.55 (15.04); hindwing length 11.29–11.83 (11.56); pronotum length 6.26–6.43 (6.34); prozone length 1.90–1.96 (1.93); pronotum width 2.47–2.66 (2.56); pronotum narrow width 1.72–1.82 (1.77); head width 5.18–5.58 (5.38); head vertex to clypeus 2.19–2.43 (2.31); frons width 2.01–2.25 (2.13); frons height 0.82–0.90 (0.86); prothoracic femur length 6.33–6.58 (6.46); mesothoracic femur length 8.20–8.21 (8.20); mesothoracic tibia length 6.49–6.53 (6.51); mesothoracic tarsus length 6.01–6.24 (6.13); metathoracic femur length 8.26–8.54 (8.40); metathoracic tibia length 8.69–8.81 (8.75); metathoracic tarsus length 8.81–9.16 (8.98); pronotal elongation measure 0.30; pronotal shape measure 0.40–0.41 (0.40); head shape measure 0.42–0.44 (0.43); frons shape measure 0.40–0.41 (0.40); anteroventral femoral spine count 14–15 (14); anteroventral tibial spine count 10; posteroventral tibial spine count 7.

*Head* (Fig. 41B): Transverse, the juxta-ocular protuberances large, the apex in the lateral half; the vertex is concave; vertex just above the dorsal margin of the eyes. The central ocellus the same size as the lateral ocelli. The carina on the frons pronounced.

*Pronotum* (Fig. 47I): As described for males.

*Prothoracic Legs*: Anterior (internal) surface of femur darkly pigmented, pale regions along the ventral and dorsal margins; the ventral surface pale, but with two darkly pigmented medial spots, one in line with the second most proximal posteroventral spine and the other just proximal to the most distal posteroventral spine. Posterior prothoracic femoral genicular spine slightly smaller than posteroventral spines, originating well proximal to the beginning of the genicular lobe. Prothoracic tibial posteroventral spines with the first (proximal) and third the same size, the second significantly longer, the fourth through sixth the shortest and of similar length to each other.

*Meso- and Metathoracic Legs*: As described for males.

*Wings*: The costal region of forewing mottled with pale and dark colors, irregular banding present; mostly pale with a few dark marks, no regular banding; vein coloration matching surroundings; discoidal region darker proximally, a central pale mark, then dark, turning pale in the distal quarter. Forewings not asymmetrically colored; extending to around the terminus of the abdomen.

*Abdomen*: Widened, the fourth tergite the widest region before a gradual posterior narrowing. Seventh tergites with small posterolateral tergal projections. Supra-anal plate slightly transverse, a broadly rounded terminus.

###### Etymology.

A noun in the genitive case, *Liturgusa bororum* is named for the Bora people, a marginalized tribe of people native to parts of the Amazon basin in northern Peru, Colombia and Brazil. Their current population is estimated at 2,000 and after a devastating period during the rubber boom, they remain with no indigenous reserves.

##### Liturgusa cura sp. n.

http://zoobank.org/E647BC5B-7C9A-420D-93DD-4A5E63CC88C2

http://species-id.net/wiki/Liturgusa_cura

###### Type.

Holotype Male, pinned. Academy of Natural Sciences of Drexel University, Philadelphia, PA, USA.

###### Type locality.

Venezuela, Villa de Cura, AR. Venezuela, 1-VII-57, E. Doreste (Lat. 10.003890, Long. -67.476421).

###### Material examined.

*Liturgusa cura* sp. n.

**Table:** 

Sex	Type	Country	Label	Latitude Longitude	Code
Male	Holotype	Venezuela	Villa de Cura, AR. Venezuela, 1-VII-57, E. Doreste	10.003890, -67.476421	ANSP 082
Female	Allotype	Venezuela			MALUZ 004

###### Diagnosis.

The smallest *Liturgusa* species, *Liturgusa cura* is most similar to *Liturgusa bororum* and *Liturgusa manausensis*, but is located only in northern Venezuela, a unique distribution. Also, *Liturgusa cura* has rounded posterolateral margins of the metazone, distinct from *Liturgusa manausensis*, as well as a mostly pale ventral prothoracic femoral surface (male has a medial brown marking) that is distinguished from *Liturgusa bororum*.

###### Description.

**Figure 9.**
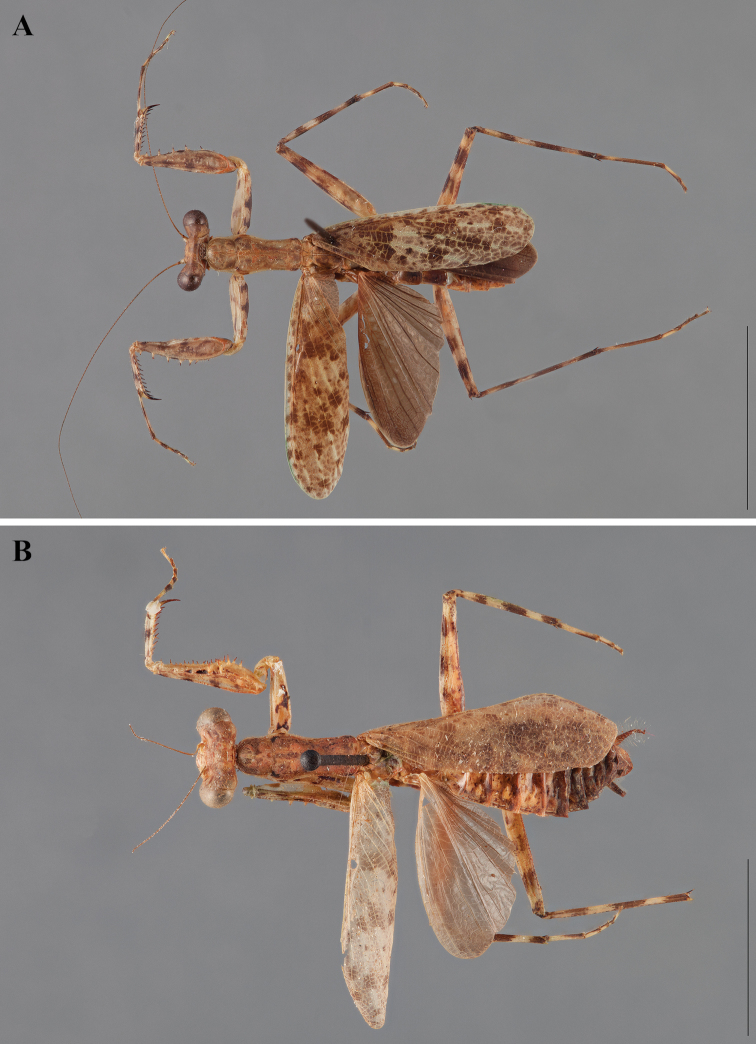
*Liturgusa cura* sp. n., dorsal habitus: **A** holotype male from Venezuela (ANSP 082) **B** allotype female from Venezuela (MALUZ 004).

**Male.** (Fig. 9A) N=1: Body length 18.74; forewing length 12.36; hindwing length 9.73; pronotum length 5.16; prozone length 1.57; pronotum width 2.19; pronotum narrow width 1.62; head width 4.42; head vertex to clypeus 1.78; frons width 1.51; frons height 0.52; prothoracic femur length 5.22; mesothoracic femur length 6.47; mesothoracic tibia length 4.83; mesothoracic tarsus length 4.56; metathoracic femur length 6.56; metathoracic tibia length 6.90; metathoracic tarsus length 6.38; pronotal elongation measure 0.30; pronotal shape measure 0.42; head shape measure 0.40; frons shape measure 0.34; anteroventral femoral spine count 15; anteroventral tibial spine count 10; posteroventral tibial spine count 7.

*Head* (Fig. 41C): Transverse, the juxta-ocular protuberances small, the apex in the lateral half; the vertex is straight; vertex just below the dorsal margin of the eyes. Frontal suture with a slight medial carina forming a continuous arc, the region ventral and dorsal to the suture not depressed. Ocelli small and protruding slightly on small cuticular mounds; the central ocellus slightly larger than the lateral ocelli; the lateral ocelli oriented outward. The carina on the frons not pronounced. Clypeus transverse, the upper margin slightly convex, the lower margin straight or slightly concave; the central, transverse carina not very pronounced, straight. Antennae scape and pedicel pale, the flagellum light brown. Area around frontal suture, vertex and the juxta-ocular protuberances brown with black or darker markings, no distinct transverse band. Lower region of frons dark brown, a small pale region along ventral margin; clypeus pale; the mandibles and labrum pale with darker brown markings. Palpi are pale.

*Pronotum* (Fig. 47J): Slightly less than three times as long as wide with a moderately defined supra-coxal bulge; dorsal surface mostly smooth, but with tubercles in the posterolateral corners of the metazone. Prozone a little broader than long, the lateral margins nearly parallel, tapering toward a broad and rounded anterior margin; margins smooth or with very small tubercles. Metazone with shallow concave lateral margins; posterior margin with an emargination; margins with small, disperse tubercles; the dorsal surface of the posterior third of the metazone slightly depressed. Mostly pale or brown with darker brown or black markings, two prominent black marks positioned anterolaterally in the metazone.

*Prothoracic Legs*: Femur normal with a concave dorsal margin; defined pale to dark banding on posterior (external) surface; anterior (internal) surface with an irregular medial band that is interrupted with pale areas; the ventral surface mostly pale, but with a dark brown mark medially between the second and third posteroventral spines. Posterior surface of femur with few tubercles. A relatively large and shallow femoral pit to accommodate terminal posteroventral tibial spine positioned medial to and just distal to the proximal most posteroventral spine, in line with the most distal discoidal spine; pit is pigmented black. Posterior prothoracic femoral genicular spine a little smaller than posteroventral spines, originating distal to the beginning of the genicular lobe. Prothoracic tibial posteroventral spines with the first (proximal) of similar size to the third through sixth, the second longer. Prothoracic coxae smooth, the anterior surface with a small, black mark medially in the proximal half as well as a small black spot medially towards the distal terminus.

*Meso- and Metathoracic Legs*: Femora with ventral (posterior) carina; dorsal (anterior) carina present. Mesotarsi with first segment shorter than the remaining segments combined.

*Wings*: Forewings mottled with pale, green, and brown coloration; the costal region with regular banding with alternating pale and dark brown, less defined proximally; vein coloration is pale and brown depending on surrounding coloration; discoidal region with a large centrally located pale marking. Forewings asymmetrically colored, one slightly darkened; extending to approximately the terminus of the abdomen. Hindwings with opaque black discoidal region, darker in the anterior half; the anal region smoky black and translucent; the terminus of the discoidal region projecting far beyond the distal margin of anal region, the wing appearing elongate.

*Abdomen*: Slightly widened, the fifth tergite the widest region before a gradual posterior narrowing; a smooth, brown and black colored dorsal surface. Tergites without posterolateral tergal projections. Supra-anal plate slightly transverse, an evenly rounded lobe. Subgenital plate irregularly rounded and without styli.

*Genital Complex* (Fig. 51E.1): The main body of ventral left sclerite (L4A) slightly elongate, the left margin with a large depression near the terminus, the left side with an elongate depression, lacking a distal process (pda). The apofisis falloid (afa) of the main body of dorsal left sclerite (L4B) short, tapering to a sharp point; the apical process (paa) elongate, the terminus evenly rounded. The right dorsal phallomere (fda) of the first sclerite of right phallomere (R1) tapers to a rounded, membranous terminus; the ventral plate (pia) small and narrow, not expanded proximally, with a smooth surface; the ventral process (pva) small and c-shaped, with irregular margins.

**Female.** (Fig. 9B) N=1: Body length 24.15; forewing length 14.24; hindwing length 10.75; pronotum length 6.84; prozone length 2.00; pronotum width 2.76; pronotum narrow width 2.09; head width 5.72; head vertex to clypeus 2.36; frons width 2.16; frons height 0.89; prothoracic femur length 6.32; mesothoracic femur length 7.68; mesothoracic tibia length 6.18; mesothoracic tarsus length 5.23; metathoracic femur length 7.57; metathoracic tibia length 8.61; pronotal elongation measure 0.29; pronotal shape measure 0.40; head shape measure 0.41; frons shape measure 0.41; anteroventral femoral spine count 15; anteroventral tibial spine count 10; posteroventral tibial spine count 7.

*Head* (Fig. 41D): Transverse, the juxta-ocular protuberances large, the apex just lateral to the middle; the vertex is convex; vertex just above the dorsal margin of the eyes. Frontal suture with a slight medial carina forming a continuous arc, the region ventral and dorsal to the suture not depressed. Entire vertex above the frontal suture projecting anteriorly, more pronounced than the carina of the frontal suture; two depressions present at the ventral terminus of the parietal suture. Ocelli small and protruding slightly on an elevated carina connecting all three. Clypeus transverse, the upper margin convex, the lower margin concave; the central, transverse carina pronounced, straight. Antennae scape and pedicel pale, the flagellum pale. Area around frontal suture, vertex and the juxta-ocular protuberances mostly pale with brown and black speckling and larger markings, no distinct transverse band. Frons, clypeus, and labrum pale with some brown speckling.

*Pronotum* (Fig. 47K): As described for males.

*Prothoracic Legs*: Anterior (internal) surface with an irregular medial band that is interrupted with pale areas; the ventral surface pale. A relatively large and shallow femoral pit to accommodate terminal posteroventral tibial spine positioned medial to and just distal to the proximal most posteroventral spine, in line with the most distal discoidal spine; the lateral margin of the pit extends to the lateral margin of the femur, therefore the margin is depressed between the two posteroventral spines; pit is pigmented black. Prothoracic tibial posteroventral spines with the first (proximal) smallest and the fourth through sixth of similar length, the second and third are longer. Prothoracic coxae smooth; the anterior surface with a small black band medially in the proximal half as well as a small black spot medially towards the distal terminus.

*Meso- and Metathoracic Legs*: As described for males.

*Wings*: The terminus of the discoidal region of the hindwing projecting beyond the distal margin of anal region, the wing appearing elongate.

*Abdomen*: Widened, the fifth tergite the widest region before a gradual posterior narrowing; a smooth, brown and black colored dorsal surface. Tergites without posterolateral tergal projections. Supra-anal plate longer than broad, an evenly rounded lobe.

###### Etymology.

A noun in apposition, *Liturgusa cura* is named from the Villa de Cura in northern Venezuela where the species was collected.

##### Liturgusa fossetti sp. n.

http://zoobank.org/3FE44B7B-B1C3-4294-982C-4FDD299D8A3C

http://species-id.net/wiki/Liturgusa_fossetti

###### Type.

Holotype Male, pinned. Cleveland Museum of Natural History, Cleveland, OH, USA.

###### Type locality.

Panama CZ, Madden Res., May 11‚ 72, R&E Froeschner (Lat. 9.119892, Long. -79.619867).

###### Material examined.

*Liturgusa fossetti* sp. n.

**Table:** 

Sex	Type	Country	Label	Latitude Longitude	Code
Male	Holotype	Panama	CZ, Madden Res., May 11 ‚72, R&E Froeschner, USNM 013	9.119892, -79.619867	GSMC003836
Female	Allotype	Nicaragua	Rio San Juan, Refugio Bartola sur le Rio San Juan, 10.974309°N, 84.338318°W, 52 m, 1-5 November, 2010, Coll: Gavin J. Svenson	10.974309, -84.338318	GSMC003425
Male	Paratype	Panama	Barro Colo Isld., Canal Zone. I-7-1929, Collector C.H. Curran	9.164966, -79.837098	AMNH 040
Female	Paratype	Costa Rica			ANSP 100
Female	Paratype	Panama	Changuinola, 1-III-1917, C.B. Williams, on cocoa	9.477669, -82.473596	BMNH 087
Male	Paratype	Panama	Gatun C.Z., March 1930, TO Zachokke, Tres Rios Plantation	9.270651, -79.909661	CAS 020
Male, Female	Paratype	Costa Rica	Puntarenas Prov. Osa pen., nr. Carate, 8.43458°N, 83.38195°W, 10m, 01.VI.2010, gen. coll. 2010 ent. Class colr. KBM01061004	8.434580, -83.381956	GSMC003075-76
2 Males, 2 Females	Paratype	Costa Rica	Puntarenas Prov. Osa pen., nr. Metapalo, 8.40765°N, 83.2820166°W, 14m, 29.V.2010, hand coll., 2010 ent. class colr. KBM02061002	8.407650, -83.282017	GSMC003078, GSMC003080-82
Male	Paratype	Panama	Darien, F. Geay, 1899	8.092070, -77.722150	MNHN 069

###### Diagnosis.

A medium size species with a moderately elongate pronotum, *Liturgusa fossetti* is most similar to *Liturgusa maya*, which is also distributed in Central America. However, *Liturgusa fossetti* can easily be distinguished from *Liturgusa maya* and other Central American species by the yellow coloration on the hindwings.

###### Description.

**Figure 10.**
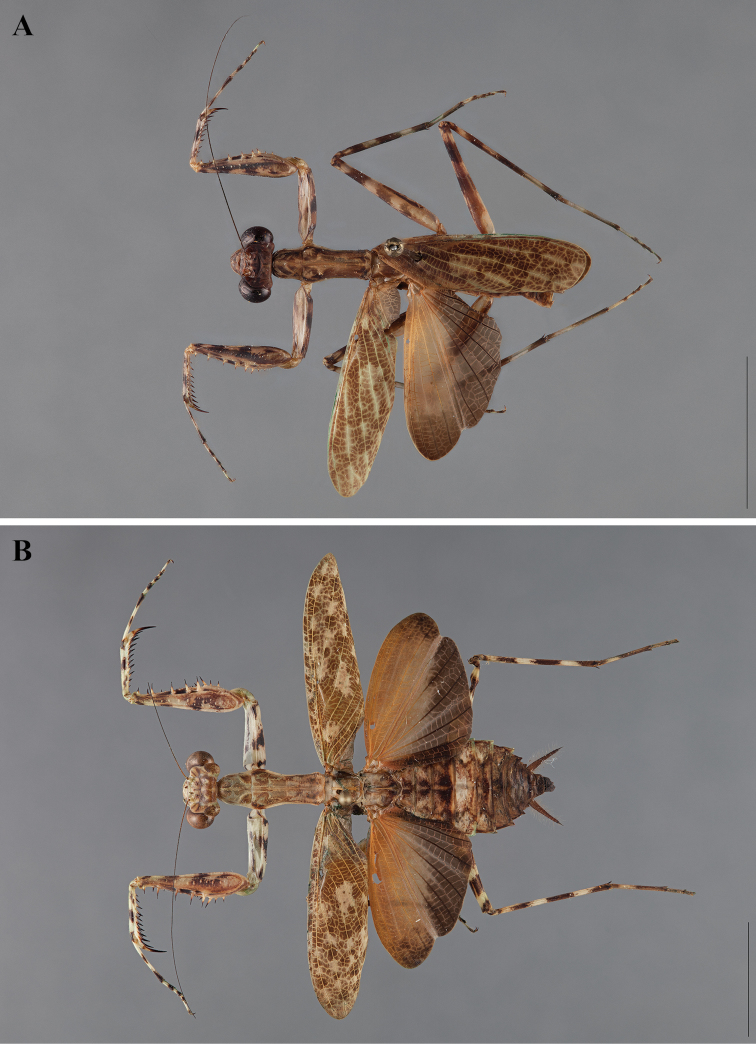
*Liturgusa fossetti* sp. n., dorsal habitus: **A** holotype male from Panama (CLEV GSMC003836) **B** allotype female from Nicaragua (CLEV GSMC003425).

**Male.** (Figs 2A, 10A) N=3: Body length 21.36–22.18 (21.76); forewing length 13.60–14.55 (14.21); hindwing length 10.26–11.53 (11.04); pronotum length 6.37–6.59 (6.49); prozone length 1.92–2.08 (1.98); pronotum width 2.47–2.54 (2.50); pronotum narrow width 1.63–1.84 (1.76); head width 4.96–5.13 (5.05); head vertex to clypeus 1.87–2.06 (1.95); frons width 1.73–1.78 (1.76); frons height 0.66–0.76 (0.69); prothoracic femur length 6.54–6.75 (6.62); mesothoracic femur length 7.92–8.27 (8.04); mesothoracic tibia length 5.94–6.28 (6.08); mesothoracic tarsus length 5.38–5.71 (5.54); metathoracic femur length 8.01–8.27 (8.14); metathoracic tibia length 7.96–8.83 (8.39); metathoracic tarsus length 7.43–8.20 (7.82); pronotal elongation measure 0.30–0.32 (0.30); pronotal shape measure 0.38–0.39 (0.39); head shape measure 0.37–0.40 (0.39); frons shape measure 0.38–0.43 (0.40); anteroventral femoral spine count 13–15 (15); anteroventral tibial spine count 10; posteroventral tibial spine count 7.

*Head* (Fig. 41E): Transverse, the juxta-ocular protuberances small, the apex just lateral to the midline; the vertex is straight, but sometimes dips just prior to the parietal sutures, even with the dorsal margin of the eyes. Frontal suture with a medial carina forming a continuous arc, the region just ventral to the carina depressed and the region just dorsal to the carina slightly depressed just lateral to the midline. Ocelli small, the central slightly enlarged, all protruding on small cuticular mounds; the lateral ocelli oriented outward. The carina on the frons not very pronounced, the medial region just ventral to the carina depressed. Clypeus transverse, the upper margin convex, the lower margin slightly concave; the central, transverse carina pronounced and curved. Antennae scape pale, pedicel partly dark brown or black, the flagellum dark brown or black just slightly distal to the base. Vertex and juxta-ocular protuberances mostly dark brown with black marks and black speckling; two prominent pale marks positioned just lateral to the lateral ocelli. Lower region of frons darkly pigmented; the clypeus, labrum, and mandibles pale; the area immediately adjacent to lateral ocelli black. Palpi are pale.

*Pronotum* (Fig. 47L): Slightly less than three times long as wide with a moderately defined supra-coxal bulge; dorsal surface smooth, but with a few small tubercles in the posterolateral corners of the metazone. Prozone slightly longer than broad with slightly convex margins that gradually taper to an evenly rounded anterior margin; margins smooth or with very few blunt tubercles. Metazone with shallow concave lateral margins without interruptions or bulges, the medial region near parallel for a short distance; margins with small tubercles; posterior margin with a medial emargination; the dorsal surface of the posterior third of the metazone slightly depressed. Mostly dark with pale and black markings across the surface, black marks laterally just posterior to the supra-coxal sulcus.

*Prothoracic Legs*: Femur robust with a slightly concave dorsal margin; strongly defined pale to dark banding on posterior (external) surface; anterior (internal) surface with a thin black band running medially from the base to terminus that may be interrupted; the ventral surface pale. Posterior surface of femur with few tubercles. A femoral pit to accommodate terminal posteroventral tibial spine positioned medial to and just distal to the first most proximal posteroventral spine, distal to the most distal discoidal spine; pit is pigmented black. Posterior prothoracic femoral genicular spine much smaller than posteroventral spines, originating distal to the beginning of the genicular lobe. Prothoracic tibial posteroventral spines with the first (proximal) smallest and the third through sixth of similar length, the second longer. Prothoracic coxae smooth, the anterior surface with a black band medially in the proximal half as well as a very small black spot medially towards the distal terminus.

*Meso- and Metathoracic Legs*: Femora with ventral (posterior) carina; dorsal (anterior) carina present. Mesotarsi with first segment as long or slightly shorter than the remaining segments combined.

*Wings*: Forewings mottled with brown, pale and greenish coloration; the costal region with defined banding distally, the proximal region mostly brown; vein coloration across discoidal region pale, not matching surrounding coloration; a pale spot positioned in the proximal quarter of the discoidal region just posterior to the first radial vein; a large pale area is positioned centrally; brown coloration dominant across the discoidal region within cells, the veins pale and appearing like a net-like pattern on the brown background. Forewings often, but not always asymmetrically colored; one being mottled as described, the other is darkened significantly with a rust tone, the mottled pattern still visible; extending just beyond or as long as the abdomen. Hindwings with an opaque yellow coloration in the proximal three quarters, yellow color extending into the anterior area of the anal region, the rest is smoky and translucent; distal quarter of the discoidal region opaque black; the terminus of the discoidal region projecting beyond the distal margin of anal region, the wing appearing elongate.

*Abdomen*: Slightly widened in the middle, the fourth tergite the widest region before a gradual posterior narrowing; a smooth, brown and black colored dorsal surface. Tergites without posterolateral tergal projections. Supra-anal plate transverse, an evenly rounded terminus with a medial emargination. Subgenital plate irregularly rounded and without styli.

*Genital Complex* (Fig. 51F.1): The main body of ventral left sclerite (L4A) slightly elongate with margins that taper rapidly to a medially pointed terminus, the left side highly sclerotized, the right membranous; the left side with an elongate depression on the surface; lacking a distal process (pda). The apofisis falloid (afa) of the main body of dorsal left sclerite (L4B) broad and heavily sclerotized with rapidly tapering margins terminating with a dull point, the concave margin strongly defined; the apical process (paa) thick and with a pronounced bulge at the base, curved and terminating with an evenly rounded tip. The right dorsal phallomere (fda) of the first sclerite of right phallomere (R1) tapers to a rounded, membranous terminus; the ventral plate (pia) long, broadened proximally with a few defined grooves; the ventral process (pva) c-shaped and broad, both ends rounded and blunt.

**Female.** (Figs 2B, 10B) N=3: Body length 30.16–32.36 (31.60); forewing length 18.38–20.15 (19.06); hindwing length 14.25–15.27 (14.76); pronotum length 9.22–9.53 (9.34); prozone length 2.93–2.95 (2.94); pronotum width 3.52–3.70 (3.60); pronotum narrow width 2.47–2.72 (2.60); head width 6.69–7.06 (6.84); head vertex to clypeus 2.75–3.00 (2.84); frons width 2.39–2.67 (2.54); frons height 0.91–1.10 (0.99); prothoracic femur length 8.85–9.75 (9.36); mesothoracic femur length 9.78–9.97 (9.88); mesothoracic tibia length 7.56–8.11 (7.83); mesothoracic tarsus length 6.94–7.11 (7.02); metathoracic femur length 9.55–9.92 (9.75); metathoracic tibia length 10.74–10.96 (10.87); metathoracic tarsus length 9.27–9.41 (9.34); pronotal elongation measure 0.31–0.32 (0.32); pronotal shape measure 0.37–0.40 (0.39); head shape measure 0.41–0.42 (0.41); frons shape measure 0.37–0.41 (0.39); anteroventral femoral spine count 15; anteroventral tibial spine count 10; posteroventral tibial spine count 7.

*Head* (Fig. 41F): Slightly transverse, the juxta-ocular protuberances large, the apex in the middle; the vertex is straight, higher than the dorsal margin of the eyes. Antennae scape pale, pedicel dark brown or black, the flagellum dark brown or black just slightly distal to the base. Black band extending straight over the medial carina of the frontal suture, the carina pale; black markings extend ventrally and dorsally from black band. Lower region of frons with dark pigmentation; dorsolateral corners of the clypeus darkly pigmented, the brown pigment extending along the ventral margin of the central carina; the mandibles and labrum with pale and brown markings; the vertex and juxta-ocular protuberances pale with brown speckles; the area immediately adjacent to lateral ocelli black. Palpi are pale.

*Pronotum* (Fig. 47M): Dorsal surface smooth, but with tubercles in the posterior half of the metazone. Prozone longer than broad with anteriorly tapering margins.

*Prothoracic Legs*: Femur robust with a nearly straight dorsal margin; anterior (internal) surface with a degraded (pale interruptions) black band running medially from the base to terminus. A deep femoral pit to accommodate terminal posteroventral tibial spine positioned medial to and between the two most proximal posteroventral spines, slightly distal to the most distal discoidal spine. Posterior prothoracic femoral genicular spine half the length of posteroventral spines, originating distal to the beginning of the genicular lobe. Prothoracic tibial posteroventral spines with the first (proximal) smallest and the third through sixth of similar length, the second much longer.

*Meso- and Metathoracic Legs*: Mesotarsi with first segment shorter than the remaining segments combined.

*Wings*: Forewings mottled with brown, pale and greenish coloration; the costal region without defined banding, mostly brown and pale mottled; vein coloration across discoidal region pale, not matching surrounding coloration; a pale spot positioned in the proximal quarter of the discoidal region just posterior to the first radial vein; a large pale area is positioned centrally; the distal half with numerous large pale spots, the background color is dark brown; costal region widened. Forewings not asymmetrically colored; extending just proximal to the terminus of the abdomen.

*Abdomen*: Widened, the fifth tergite the widest region before a gradual posterior narrowing; elliptical in shape. Tergites with expanded and triangular posterolateral tergal projections on the fifth through seventh segments. Supra-anal plate slightly transverse, a broadly rounded, blunt terminus, a small emargination present.

###### Etymology.

A noun in the genitive case, *Liturgusa fossetti* is named in honor of James Stephen Fossett for his inspirational dedication to adventure and exploration

##### Liturgusa kirtlandi sp. n.

http://zoobank.org/EDE23094-02F3-497C-B86E-0A74B582C9DD

http://species-id.net/wiki/Liturgusa_kirtlandi

###### Type.

Holotype Male, pinned. Cleveland Museum of Natural History, Cleveland, OH, USA.

###### Type locality.

Bolivia: Dpto. Santa Cruz, Reserva Natural Potrerillo del Guenda, 17°40.281'S, 063°27.451'W, 400 m, 3-9.XI.2009, at MV.UV lights & gen. coll., Coll: G.J. Svenson (Lat. -17.671350, Long. -63.457517).

###### Material examined.

*Liturgusa kirtlandi* sp. n.

**Table:** 

Sex	Type	Country	Label	Latitude Longitude	Code
Male	Holotype	Bolivia	Dpto. Santa Cruz, Reserva Natural Potrerillo del Guenda, 17°40.281'S, 063°27.451'W, 400 m, 3-9.XI.2009, at MV.UV lights & gen. coll., Coll: G.J. Svenson	-17.671350, -63.457517	GSMC000281
Female	Allotype	Bolivia	Dpto. Santa Cruz, Reserva Natural Potrerillo del Guenda, 17°40.281'S, 063°27.451'W, 400 m, 3-9.XI.2009, at MV.UV lights & gen. coll., Coll: G.J. Svenson	-17.671350, -63.457517	GSMC000274
7 Males, 6 Females	Paratype	Bolivia	Dpto. Santa Cruz, Reserva Natural Potrerillo del Guenda, 17°40.281'S, 063°27.451'W, 400 m, 3-9.XI.2009, at MV.UV lights & gen. coll., Coll: G.J. Svenson	-17.671350, -63.457517	GSMC000252-53, GSMC000267, GSMC000271, GSMC000275-76, GSMC000279-80, GSMC000282-84, GSMC000306-07
2 Females	Paratype	Bolivia	Dpto. Santa Cruz, nr. Buena Vista, 17°39.100'S, 063°35.136'W, 390 m, 14.XI.2009, at MV.UV lights in cut trees, Coll: G.J. Svenson	-17.651667, -63.585600	GSMC000272, GSMC000328

###### Diagnosis.

Most similar to *Liturgusa maya* and *Liturgusa trinidadensis*, but easily diagnosed by the elongate, thin apical process (paa) with an angled blunt tip rather than an evenly rounded terminus as is seen in the other two species. See *Liturgusa trinidadensis* for a list of external features that distinguish the species from *Liturgusa kirtlandi*. The main difference between *Liturgusa maya* and *Liturgusa kirtlandi* is the presence of tubercles on the pronotum in *Liturgusa kirtlandi* and not in *Liturgusa maya*. Finally, *Liturgusa trinidadensis* has numerous tubercles in the posterolateral corners of the metazone while *Liturgusa kirtlandi* does not.

###### Description.

**Figure 11.**
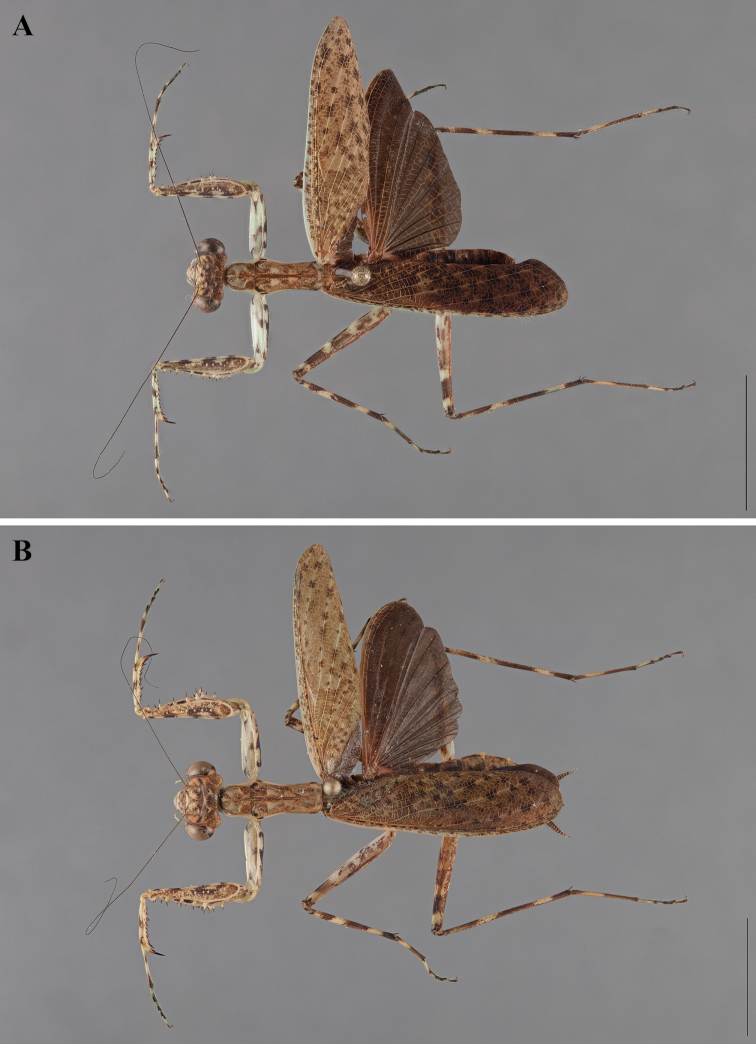
*Liturgusa kirtlandi* sp. n., dorsal habitus: **A** holotype male from Santa Cruz, Bolivia (CLEV GSMC000281) **B** allotype female from Santa Cruz, Bolivia (CLEV GSMC000274).

**Male.** (Fig. 11A) N=4: Body length 25.82–26.83 (26.2); forewing length 17.37–17.91 (17.64); hindwing length 13.49–14.19 (13.86); pronotum length 6.95–7.35 (7.18); prozone length 2.06–2.26 (2.18); pronotum width 2.63–2.78 (2.70); pronotum narrow width 1.96–2.02 (1.99); head width 5.35–5.62 (5.47); head vertex to clypeus 2.14–2.21 (2.18); frons width 1.91–2.07 (1.98); frons height 0.61–0.77 (0.67); prothoracic femur length 6.76–7.04 (6.90); mesothoracic femur length 8.46–8.88 (8.71); mesothoracic tibia length 6.73–7.09 (6.94); mesothoracic tarsus length 5.80–6.10 (5.94); metathoracic femur length 8.54–9.12 (8.83); metathoracic tibia length 10.01–10.50 (10.33); metathoracic tarsus length 8.53–8.84 (8.70); pronotal elongation measure 0.30–0.31 (0.30); pronotal shape measure 0.36–0.39 (0.38); head shape measure 0.39–0.40 (0.40); frons shape measure 0.31–0.39 (0.34); anteroventral femoral spine count 13–14 (14); anteroventral tibial spine count 9; posteroventral tibial spine count 7.

*Head* (Fig. 41G): Transverse, the juxta-ocular protuberances small, but pronounced, the apex just lateral to the midline; the vertex is slightly convex, even with the dorsal margin of the eyes. Frontal suture with a slight medial carina forming a continuous arc, the entire carina depressed into a trough. Ocelli small, the central more enlarged (about twice the size as the lateral), all protruding on small cuticular mounds; the lateral ocelli oriented outward. The carina on the frons not pronounced, the medial region just ventral to the carina depressed. Clypeus transverse, the upper margin slightly convex, the lower margin slightly concave or straight; the central, transverse carina pronounced and straight. Antennae scape and pedicel pale, the flagellum black just slightly distal to the base. Black band extending straight over the medial carina of the frontal suture, the very center of the carina pale; black markings extend ventrally and dorsally from black band; two prominent pale marks positioned just lateral to the lateral ocelli; two pale marks positioned on the lower region of the vertex. Lower region of frons darkly pigmented; a central dark band running across the middle of the clypeus, the dorsal and ventral regions pale; the mandibles and labrum with pale and brown markings; the vertex and juxta-ocular protuberances mostly black with pale speckles; the area immediately adjacent to lateral ocelli black. Palpi are pale.

*Pronotum* (Fig. 47N): A little less than three times as long as wide with a moderately defined supra-coxal bulge; dorsal surface entirely smooth or at most with very few, small tubercles. Prozone square with slightly convex margins that gradually taper to an evenly rounded anterior margin; margins smooth or with very few blunt tubercles. Metazone with concave lateral margins without interruptions or bulges; margins with numerous small tubercles; posterior margin with a medial emargination; the dorsal surface of the posterior third of the metazone not depressed. Mostly dark with pale and black markings across the surface, faint swirls present on the metazone just posterior to the supra-coxal sulcus.

*Prothoracic Legs*: Femur normal with a straight dorsal margin; strongly defined pale to dark banding on posterior (external) surface; anterior (internal) surface with a very thin black band running medially from the base to terminus, some small segments more faded, two slight dark marks present dorsal to the band, one medial and the other near the femoral brush; the ventral surface pale. Posterior surface of femur with few tubercles. A shallow femoral pit to accommodate terminal posteroventral tibial spine positioned medial to and between the first two proximal posteroventral spines, in line with the most distal discoidal spine; pit is pigmented brown. Posterior prothoracic femoral genicular spine much smaller than posteroventral spines, originating distal to the beginning of the genicular lobe. Prothoracic tibial posteroventral spines with the first (proximal) smallest and the third through sixth of similar length, the second much longer. Prothoracic coxae smooth, the anterior surface with a very small, black mark medially in the proximal half as well as a very small black spot medially towards the distal terminus.

*Meso- and Metathoracic Legs*: Femora with ventral (posterior) carina; dorsal (anterior) carina present. Mesotarsi with first segment shorter or as long as the remaining segments combined.

*Wings*: Forewings mottled with brown, pale and black coloration; the costal region without defined banding distally, proximal region mostly brown with a green/pale anterior margin; vein coloration is pale, not matching surrounding cell coloration; a larger, but slightly more pale area is positioned centrally; brown color dominant across discoidal region with small regularly dispersed irregularly shaped black marks in the distal half. Forewings asymmetrically colored; one being mottled as described the other is darkened significantly with a black or rust tone, the mottled pattern still visible; extending just beyond the terminus of the abdomen. Hindwings with opaque discoidal region, colored rust or pale brown proximally and along the anterior margin, otherwise black; the anal region smoky black and translucent; the terminus of the discoidal region projecting far beyond the distal margin of anal region, the wing appearing elongate.

*Abdomen*: Slightly widened, the fifth or sixth tergite the widest region before a gradual posterior narrowing; a smooth, brown and black colored dorsal surface. Tergites with small posterolateral tergal projections on the terminal three segments. Supra-anal plate slightly wider than long, a broad, blunt terminus with a slight medial emargination. Subgenital plate irregularly rounded and without styli.

*Genital Complex* (Fig. 51G.1–G.4): The main body of ventral left sclerite (L4A) broadly elliptical with rounded terminus, but with a rounded distal process (pda) positioned medially (may be short and rather blunt or more elongate and narrow), projecting at an angle towards the right phallomere (R1), appearing like a small, well-sclerotized tooth; sometimes a depression on the opposite lateral half from the pda is present. The apofisis falloid (afa) of the main body of dorsal left sclerite (L4B) very short, quickly narrowing to a sharp point, not curved; the apical process (paa) elongate and thin, the terminus forming an angled blunt tip. The right dorsal phallomere (fda) of the first sclerite of right phallomere (R1) tapers to a rounded, membranous terminus; the ventral plate (pia) short with strongly defined grooves; the ventral process (pva) small and tooth-like and curved at the proximal base, the distal tip irregular, but narrowing to a point.

**Female.** (Figs 1B, 11B) N=5: Body length 30.11–32.79 (30.94); forewing length 20.14–21.41 (20.55); hindwing length 15.17–16.86 (15.84); pronotum length 8.16–8.70 (8.43); prozone length 2.44–2.66 (2.57); pronotum width 3.12–3.31 (3.23); pronotum narrow width 2.36–2.41 (2.39); head width 6.31–6.76 (6.60); head vertex to clypeus 2.78–2.97 (2.85); frons width 2.46–2.71 (2.56); frons height 0.78–0.93 (0.88); prothoracic femur length 8.13–8.53 (8.25); mesothoracic femur length 9.27–9.84 (9.50); mesothoracic tibia length 7.70–8.16 (7.85); mesothoracic tarsus length 6.23–6.69 (6.43); metathoracic femur length 9.39–9.87 (9.58); metathoracic tibia length 11.41–11.75 (11.57); metathoracic tarsus length 9.16–9.79 (9.59); pronotal elongation measure 0.30–0.31 (0.30); pronotal shape measure 0.37–0.40 (0.38); head shape measure 0.42–0.45 (0.43); frons shape measure 0.31–0.36 (0.34); anteroventral femoral spine count 13–14 (13); anteroventral tibial spine count 9–10 (9); posteroventral tibial spine count 7.

*Head* (Fig. 41H): Slightly transverse, the juxta-ocular protuberances large, the apex in the middle; the vertex is straight, higher than the dorsal margin of the eyes. The vertex and juxta-ocular protuberances evenly mottled with black, brown and pale markings.

*Pronotum* (Fig. 47O): As described for males.

*Prothoracic Legs*: Femur normal with a near straight or slightly concave dorsal margin. A shallow femoral pit to accommodate terminal posteroventral tibial spine positioned medial to and just distal to the first most proximal posteroventral spine, in line with the most distal discoidal spine.

*Meso- and Metathoracic Legs*: As described for males.

*Wings*: The costal region without defined banding, mostly pale with some brown mottling. The forewing extending just shy of the terminus of the abdomen.

*Abdomen*: Slightly widened, the fifth tergite the widest region before a gradual posterior narrowing. Tergites with small posterolateral tergal projections on the terminal four segments. Supra-anal plate almost square, a broad, blunt terminus with a slight medial emargination.

###### Etymology.

A noun in the genitive case, *Liturgusa kirtlandi* is named in honor of Jared Potter Kirtland (1793-1877) for his contributions to natural science and medicine in northern Ohio as one of the founding trustees of the Cleveland Academy of Natural Science in 1845, later renamed the Kirtland Society of Natural History in his honor in 1865. The KSNH remained in existence until 1920 when the Cleveland Museum of Natural History was founded, the various natural history collections moving to the new museum.

##### Liturgusa manausensis sp. n.

http://zoobank.org/6ABFB9DE-746E-4925-852F-BEB1591303F9

http://species-id.net/wiki/Liturgusa_manausensis

###### Type.

Holotype Male, pinned. National Museum of Natural History, Smithsonian Institution, Washington, DC, USA.

###### Type locality.

Brazil: Amazonas, 18.1 km e Campinas field sta. Km 60 n Manaus, 22 Feb 1979, 02°30'S, 060°15'W, Terra firme forest canopy fogged with Pyrethrum Sample # 12, Montgomery, Erwin, Schimmel, Krischik, Date, Bacon colls (Lat. -2.500000, Long. -60.250000).

###### Material examined.

*Liturgusa manausensis* sp. n.

**Table:** 

Sex	Type	Country	Label	Latitude Longitude	Code
Male	Holotype	Brazil	Amazonas, 18.1 km e Campinas field sta. Km 60 n Manaus, 22 Feb 1979, 02°30'S, 060°15'W, Terra firme forest canopy fogged with Pyrethrum Sample # 12, Montgomery, Erwin, Schimmel, Krischik, Date, Bacon colls.	-2.500000, -60.250000	USNM 001; USNM ENT 00873997

###### Diagnosis.

One of the smaller species, *Liturgusa manausensis* is mostly black and whitish across the body with strongly asymmetrically colored forewings. Known only from north of Manaus, Brazil. The male genitalia are distinct from others in the Maya Group with its elongate ventral left sclerite (L4A) with a medial bulge.

###### Description.

**Figure 12.**
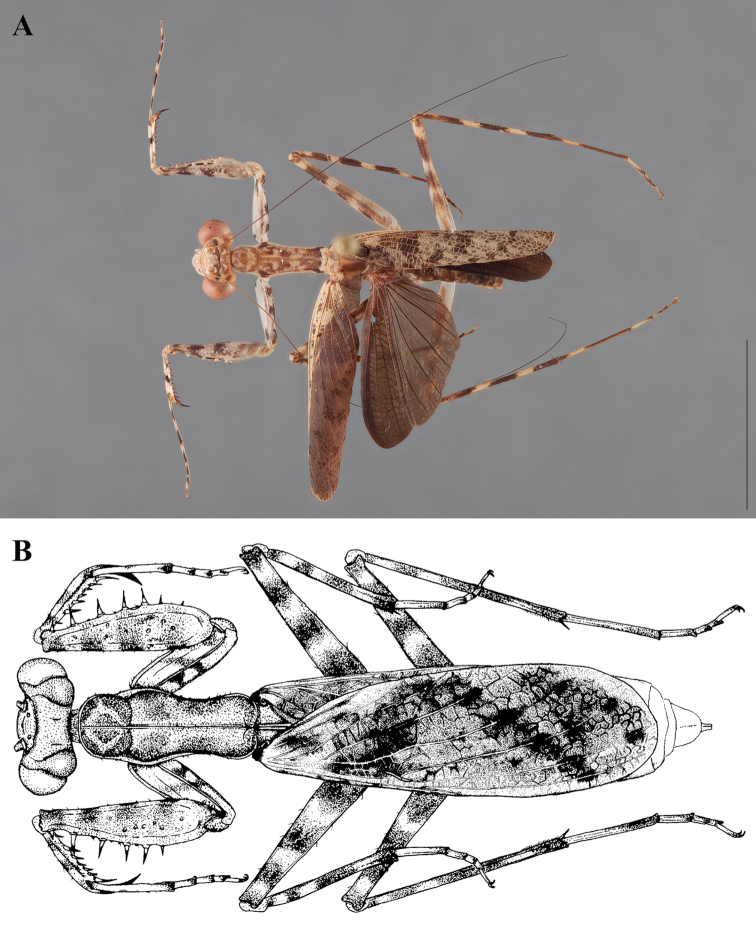
*Liturgusa*, dorsal habitus: **A** *Liturgusa manausensis* sp. n., holotype male from north of Manaus, Brazil (USNM 001) **B** *Liturgusa maya* Saussure & Zehntner, 1894, illustration of female from Peru by Julio Rivera.

**Male.** (Fig. 12A) N=1: Body length 19.40; forewing length 12.93; hindwing length 9.66; pronotum length 5.68; prozone length 1.62; pronotum width 2.07; pronotum narrow width 1.47; head width 4.66; frons width 1.59; frons height 0.54; prothoracic femur length 5.54; mesothoracic femur length 7.27; mesothoracic tibia length 5.72; mesothoracic tarsus length 4.97; metathoracic femur length 7.52; metathoracic tibia length 7.95; metathoracic tarsus length 7.83; pronotal elongation measure 0.28; pronotal shape measure 0.36; frons shape measure 0.34; anteroventral femoral spine count 15; anteroventral tibial spine count 10; posteroventral tibial spine count 7.

*Head* (Fig. 42E): Transverse, the juxta-ocular protuberances small, but pronounced, the apex in the lateral half; the vertex is straight, just below the dorsal margin of the eyes. Frontal suture with a slight medial carina forming a continuous arc, the entire carina depressed into a trough, the margins sharp transitions. Ocelli small and protruding slightly on small cuticular mounds; the lateral ocelli oriented outward. The carina on the frons not pronounced, but present. Clypeus transverse, the upper margin slightly convex, the lower margin straight; the central, transverse carina not very pronounced, straight. Antennae scape and pedicel pale, the flagellum pale proximally, fading gradually to brown, then to black distally. Black band extending straight over the medial carina of the frontal suture, the center of the carina pale; two pale marks positioned on the lower region of the vertex. Frons pale; clypeus pale; the mandibles and labrum pale; the vertex and juxta-ocular protuberances mostly pale with brown speckling; the area immediately adjacent to lateral ocelli black. Palpi are pale.

*Pronotum* (Fig. 47P): A little less than three times as long as wide with a moderately defined supra-coxal bulge; dorsal surface with disperse, small tubercles in the posterior half. Prozone square with slightly convex margins that gradually taper to an evenly rounded anterior margin; margins smooth or with very few blunt tubercles. Metazone with concave lateral margins with medial bulges; margins with numerous small tubercles; posterior margin with flat posterolateral corners and with a medial emargination; the dorsal surface of the posterior third of the metazone depressed. Mostly pale with black markings across the surface, especially just before the posterior margin and laterally just posterior to the supra-coxal sulcus.

*Prothoracic Legs*: Femur normal with a concave dorsal margin; strongly defined pale to dark banding on posterior (external) surface; anterior (internal) surface with a three black bands, one basally, one medially that connects to the dorsal margin, and one adjacent to the femoral brush; the ventral surface pale. Posterior surface of femur with few tubercles. A shallow femoral pit to accommodate terminal posteroventral tibial spine positioned medial to and between the first two proximal posteroventral spines, in line with the most distal discoidal spine; pit is pigmented brown. Posterior prothoracic femoral genicular spine half the length of the posteroventral spines, originating distal to the beginning of the genicular lobe. Prothoracic tibial posteroventral spines with the first (proximal) smallest and the third through sixth of similar length, the second much longer. Prothoracic coxae smooth, the anterior surface with a very small, black spot medially towards the distal terminus.

*Meso- and Metathoracic Legs*: Femora with ventral (posterior) carina; dorsal (anterior) carina present. Mesotarsi with first segment shorter or as long as the remaining segments combined.

*Wings*: Forewings mottled with pale (whitish) and black coloration; the costal region mostly pale with a few black marks, no regular banding; vein coloration is pale, not matching surrounding cell coloration; discoidal region evenly mottled with pale and black markings, whitish color more dominant distally. Forewings asymmetrically colored; one being mottled as described the other is darkened significantly with a black tone, the mottled pattern barely visible; extending just beyond the terminus of the abdomen. Hindwings with opaque black discoidal region; the anal region smoky black and translucent; the terminus of the discoidal region projecting far beyond the distal margin of anal region, the wing appearing elongate.

*Abdomen*: Slightly widened, the fifth or sixth tergite the widest region before a gradual posterior narrowing; a smooth, brown and black colored dorsal surface. Tergites without posterolateral tergal projections. Supra-anal plate transverse with a broadly rounded terminus. Subgenital plate irregularly rounded and without styli.

*Genital Complex* (Fig. 51H.1): The main body of ventral left sclerite (L4A) elongate, a rounded terminus with a medial bulge that could be considered as a slightly protruding and blunt distal process (pda). The apofisis falloid (afa) of the main body of dorsal left sclerite (L4B) elongate and forming a tapered sharp point with concave lateral margins, sometimes curved; the apical process (paa) elongate and thick, the terminus tapering to a narrowed, rounded tip. The right dorsal phallomere (fda) of the first sclerite of right phallomere (R1) tapers to a rounded, membranous terminus; the ventral plate (pia) long and broad with very well defined, curved grooves proximally; the ventral process (pva) small and tooth-like and curved at the proximal base, the distal tip irregular, but narrowing to a point.

###### Etymology.

Named for Manaus, Brazil, near where the species was collected.

##### Liturgusa maya

Saussure & Zehntner, 1894

http://species-id.net/wiki/Liturgusa_maya

Mantis annulipes (non *Mantis annulipes* Audinet Serville, 1838): von Charpentier 1843: 28–29, pl. 28 (*partim*).
Liturgousa annulipes : Saussure 1871: 102 (♂ only, *partim*).
Liturgousa cayennensis [Var.] *maya*: Saussure and Zehntner 1894: 160; Scudder 1901: 159, 407; Rehn 1903: 6; Marshall 1975: 318; Roy and Cuche 2008: 14, 21.
Liturgousa maya : Scudder 1901: 159, 419; Kirby 1904: 271; Hebard 1932: 211; Rehn 1935: 201, pl. 8, fig. 5; Hughes-Schrader 1950: 11–14, 38, 45, Table 1, Fig. 9; Hughes-Schrader 1951: 178, 180, 183–184, 186–187, Tables 1–2, Fig. 1; Hughes-Schrader 1953: 544–554; Henderson 1965: 215; Cerdá 1996: 76;
Liturgusa maya : Giglio-Tos 1927: 293; Beier 1935: 11; Jantsch 1991: 125; Terra 1995: 54; Jantsch 1999: 48; Maes and Roy 2000: 61; Lombardo and Agabiti 2001: 90, 96; Ehrmann 2002: 207; Agudelo 2004: 55, Table 3.1; Agudelo 2005: 3; Otte and Spearman 2005: 133; Agudelo et al. 2007: 116, 142; Svenson and Whiting 2009: Appendix S1.
Liturgusa maja : Passerin d‘Entrèves 1981: 61.
Liturgusa charpentieri : Giglio-Tos 1927: 294; Beier 1935: 11; Passerin d‘Entrèves 1981: 61; Terra 1995: 53; Salazar E. 1998: 105, Fig. 4; Jantsch 1999: 48; Salazar E. 1999: 10; Lombardo and Agabiti 2001: 90; Salazar E. 2002: 124; Ehrmann 2002: 207; Agudelo 2004: 55, Table 3.1; Agudelo 2005: 3; Otte and Spearman 2005: 133; Agudelo et al. 2007: 116, 141. syn. n.

###### Type.

Lectotype Male. The Natural History Museum (British Museum Natural History), London, UK.

###### Type locality.

Temax, N. Yucatan, Gaumer (Lat. 21.143702, Long. -88.942315).

###### Material examined.

*Liturgusa maya* Saussure & Zehntner, 1894.

**Table:** 

Sex	Type	Country	Label	Latitude Longitude	Code
Male	Lectotype	Mexico	Temax, N. Yucatan, Gaumer	21.143702, -88.942315	BMNH
Male	Paralectotype	Mexico	Temax, N. Yucatan, Gaumer	21.143702, -88.942315	MHNG
Female	Paralectotype	Mexico	Temax, N. Yucatan, Gaumer	21.143702, -88.942315	MHNG
Female	Paralectotype	Mexico	Temax, N. Yucatan, Gaumer	21.143702, -88.942315	BMNH
Female	Paralectotype	Mexico	Temax, N. Yucatan, Gaumer	21.143702, -88.942315	BMNH
Male	nontype	Colombia	S.A. Felipe Ovalle, Q., Ac. 33501		AMNH 011
Male	nontype	Mexico	Itzimna, Yucatan, IX-9-1964, Collectors J.C. & D. Pallister	21.025527, -89.682887	AMNH 020
Male	nontype	Venezuela	Rancho Grande, nr. Maracay, Ven. 8-VII 1946	10.350000, -67.683330	AMNH 023
Male	nontype	Panama	C.Z., Summit, XII - 1953, Collector N.L.H. Krauss	9.067496, -79.649077	AMNH 039
Male	nontype	Peru	El Campamiente, Col Perene, Peru, 19 June 1920, Cornell Univ. Exp.	-10.941437, -75.225954	ANSP 025
Male	nontype	Peru	Quiroz, Rio Paucartambo	-12.903073, -71.404748	ANSP 038
Male	nontype	Mexico	San Rafael, Veracruz, Townsend	20.190622, -96.882611	ANSP 076
Male	nontype	Costa Rica			ANSP 077
Female	figure voucher (Rehn 1935)	Costa Rica	Surubres, near Santa Mater, (Poe.). 250m, I-1906, P. Biolley!	9.598454, -84.302680	ANSP 078
Female	nontype	Costa Rica	Hamburg Farm, lower Rio Reventazon, C.R., VI.26.1936 (L. Navas), In banana field	10.250000, -83.450000	ANSP 079
Female	nontype	Costa Rica	Pozo Azul	10.186150, -84.901512	ANSP 087
Female	figure voucher (Rehn 1935)	Nicaragua	Mouth of Waspuk R.	14.631086, -84.438399	ANSP 088
Female	nontype	Panama	Corozal, C.Z., XI.17.1913, Morgan Hebard		ANSP 089
Male	nontype	Mexico	Teapa, Tabasco, Feb. H.H.S.	17.556197, -92.943064	BMNH 009
Female	nontype	Honduras	Cortes Provence, San Pedro Sula, foothills, ca. 800 ft., Stop 82-16, 5-13 May 1982, D.B. Weissman	15.475779, -88.054841	CAS 002
Male	nontype	Peru	Monson Valley, Tingo Maria, XII-2-1954, E.I. Schlinger & E.S. Ross collectors	-9.314153, -76.006745	CAS 009
Female	nontype	Peru	Monson Valley, Tingo Maria, XII-2-1954, E.I. Schlinger & E.S. Ross collectors	-9.314153, -76.006745	CAS 013
Female	nontype	Panama	Ft. Clayton, C.Z., XI-44, K.E. Frick Collector	9.005408, -79.574522	CAS 022
Female	nontype	Colombia	Buga - Valle, Bosque del Vinculo, 997m, 3°50'13.0"N, 76°18'00.0"W, 4-I-2006, Coll: T. Kondo	3.836944, -76.300000	GSMC000277
Female	nontype	Colombia	Cali - Valle, Chorro de Platu, 28-XII-2005, Coll: T. Kondo		GSMC000327
Female	nontype	Costa Rica	Puntarenas Prov. Osa pen., nr. Carate; gen. coll. 2010 ent. Class colr. KBM 01061004	8.434580, -83.381956	GSMC003014
Male	nontype	Guatemala	Izabal, Rio Dulce, Hotel Tijax. Collected along trails through old secondary growth tropical forest under rocks, logs & bark. N 15°40'12.2"W, 89°00'27.0", Elev: 49m, July 8, 2006. Coll: J. Huff, C. Viquez & D. Ortiz. Site 8.	15.670056, -89.007500	GSMC003070
Female	nontype	Nicaragua	Rio San Juan, Refugio Bartola sur le Rio San Juan, 10.974309°N, 84.338318°W, 52 m, 1-5 November, 2010, Coll: Gavin J. Svenson	10.974309, -84.338318	GSMC003426
Female	nontype	Nicaragua	Rio San Juan, Refugio Bartola sur le Rio San Juan, 10.974309°N, 84.338318°W, 52 m, 1-5 November, 2010, Coll: Gavin J. Svenson	10.974309, -84.338318	GSMC003427
Female	nontype	Nicaragua	Rio San Juan, Refugio Bartola sur le Rio San Juan, 10.974309°N, 84.338318°W, 52 m, 1-5 November, 2010, Coll: Gavin J. Svenson	10.974309, -84.338318	GSMC003428
Male	nontype	Nicaragua	Rio San Juan, Refugio Bartola sur le Rio San Juan, 10.974309°N, 84.338318°W, 52 m, 1-5 November, 2010, Coll: Gavin J. Svenson	10.974309, -84.338318	GSMC003429
Male	nontype	Nicaragua	Rio San Juan, Refugio Bartola sur le Rio San Juan, 10.974309°N, 84.338318°W, 52 m, 1-5 November, 2010, Coll: Gavin J. Svenson	10.974309, -84.338318	GSMC003430
Male	nontype	Nicaragua	Rio San Juan, Refugio Bartola sur le Rio San Juan, 10.974309°N, 84.338318°W, 52 m, 1-5 November, 2010, Coll: Gavin J. Svenson	10.974309, -84.338318	GSMC003431
Female	nontype	Nicaragua	Granada, Reserva Silvestre Privada Domatila, 11.709000°N, 85.953500°W, 68m, 6-9 November 2010, Coll: Gavin J Svenson	11.709000, -85.953500	GSMC003432
Male	nontype	Nicaragua	Rio San Juan Dpto., Refugio Bartola; KBM 18051201	10.972540, -84.338990	GSMC003597
Female	nontype	Venezuela	Dto Ptumba, Edo Zulia, Fecha 5-8-90, Collec Luis, U.	8.936828, -72.163996	MALUZ 001
Female	nontype	Venezuela	Dto Trujillo, Edo Trujillo, Fecha 20-3-89, Collec Eladio, K.	9.373003, -70.429807	MALUZ 002
Female	nontype	Peru	Cusco, Quillabamba, 3.VII.1996, W. Catalan leg., UA 684-1996	-12.861863, -72.699170	MEKRB 002
Male	nontype	Peru	Junin, Satipo. 16.V.1999, J. Laos leg. UA 1278-1999	-11.238653, -74.627567	MEKRB 003
Female	nontype	Peru	Timbes, Papayal, 2000, P. Castillo leg., UA 021-2000	-4.100794, -80.677040	MEKRB 006
Female	nontype	Ecuador	Cotopaxi, La Mana (Bosque secord), 7.VIII.2003, Rivera y Montgemuy, UA 103-2003	-0.934058, -79.218475	MEKRB 007
Female	nontype	Ecuador	Cotopaxi, La Mana (Bosque secord), 7.VIII.2003, Rivera y Montgemuy, UA 103-2003	-0.934058, -79.218475	MEKRB 008
Female	nontype	Ecuador	Cotopaxi, La Mana (Bosque secord), 7.VIII.2003, Rivera y Montgemuy, UA 103-2003	-0.934058, -79.218475	MEKRB 009
Female	nontype	Ecuador	Cotopaxi, La Mana (Bosque secord), 7.VIII.2003, Rivera y Montgemuy, UA 103-2003	-0.934058, -79.218475	MEKRB 011
Female	nontype	Colombia	Dept. Amazonas, Rio Igara Parana, 30 km aval La Chorrera, VI - VII 1974, M. Descamps rec.	-1.197385, -72.937475	MNHN 016
Female	nontype	Colombia	Dept. Amazonas, Rio Igara Parana, 30 km aval La Chorrera, VI - VII 1974, M. Descamps rec.	-1.197385, -72.937475	MNHN 018
Female	nontype	Colombia	Dept. Narino Entre Espriella Tumaco et Camp Experimental de I.F.A., 300m, 19-XI-1968, M. Descamps rec.	1.788077, -78.778943	MNHN 028
Male	nontype	Nicaragua	Zelaya El Recreo Foret, X-1984, C. Amedegnato, S. Poulain Rec.	12.178332, -84.344478	MNHN 078
Female	nontype	Honduras	Atlantida, Massif de Pico Bonita, 250m, VI/1995, T. Porion G. lachaume	15.692101, -86.853643	MNHN 085
Female	nontype	Mexico	Col. of J. Amith 80439 (2011-06-17), 16°48'57"N, 98°41'25"W, 600 m, Yoloxochitl, Mpio, S.L. Acatlan, Gro.	16.815833, -98.690278	JAC
Female	nontype	Mexico	Col. of J. Amith 80439 (2011-06-17), 16°48'57"N, 98°41'25"W, 600 m, Yoloxochitl, Mpio, S.L. Acatlan, Gro.	16.815833, -98.690278	JAC
Female	nontype	Peru	Madre de Dios, Tambopata Nat‘l Reserve, -12.8368° -69.2932°, 250m, 2005, Coll: G.J. Svenson	-12.836800, -69.293200	GSMC003005
Female	nontype	Peru	Madre de Dios, Tambopata Nat‘l Reserve, -12.8368° -69.2932°, 250m, 22-29.V.2011, Colr. UNM collections class (K.B. Miller)	-12.836800, -69.293200	CLEV
Female	nontype	Ecuador	Napo: Tiputini, Biodiversity Stn., vic. Yasuni Natl. Pk. 14-18 Feb 1999, 0°38'S, 76°10'W. DC Darling, ROM 991050	-0.633333, -76.166667	ROM 002
Female	nontype	Guatemala			SDEI 004
Male	nontype	Guatemala	Polochic R, 22.3, Guat., Barber & Schwarz Coll	15.336297, -89.732437	USNM 014; USNM ENT 00873004
Female	nontype	Peru	Tingo Maria, IV, V - 1952, P. Araoz	-9.314153, -76.006745	USNM 035; USNM ENT 00873012
Female	nontype	Guatemala	25, L. Thiel, S. Sebastian, Retalhuleu	14.567871, -91.649246	USNM 037; USNM ENT 00873013
Female	nontype	Panama	Cabima, May 17. 11, August Busck	9.129759, -79.527743	USNM 038; USNM ENT 00873014
Male	nontype	Panama	Pedro Diaz Farm, Las Sabanas, Nov. 1, 1918. G-349, Dietz and Zetek.	8.573002, -80.681300	USNM 053; USNM ENT 00873010
Female	nontype	Costa Rica	Quepos, C.R., III-17-1958, M.J. Stelzer, No. 58-17, on cacao	9.429236, -84.163056	USNM 067; USNM ENT 00873011
Female	nontype	Mexico	Tapachula, Chiapas	14.908892, -92.241002	ZMHB 002
Female	nontype	Costa Rica	Farm Hamburg, am Reventazon, 2.II.1932, F. Nevermann leg., Eing. Nr. 74. 1932	10.250000, -83.450000	ZMUH 018
Female	nontype	Costa Rica	Farm Hamburg, am Reventazon, 2.Juli.1934. F. Nevermann leg., Eing. Nr. 146. 1935	10.250000, -83.450000	ZMUH 019
Female	nontype	Peru	Madre de Dios; Rio Tambopata; Posada Amazonas; Canopy Tower; S12 48 16.6 W69 17 35.3; Sept. 2004; J.R. Cryan	-12.804611, -69.293139	GSMC003062
Female	nontype	Ecuador	10.VI.67, rec. MAZ, cuenca, Ecuador, A.C. Allyn, Acc. 1969-20	-3.010204, -78.204794	FMNH 005
Female	nontype	Peru	Tingo Maria, 670m, Hyanuce, Peru SA, Nov. 2006	-9.314153, -76.006745	FMNH 010

###### Taxonomic history.

One of the earliest species to be described, *Liturgusa maya* was referenced in early works as *Mantis annulipes*, but this is likely due to the lack of comparison with the type of *Mantis annulipes*. Saussure and Zehntner recognized the species as unique, but as a variant of *Liturgusa cayennensis*. The species was later formalized as being distinct. It is apparent from the references that include *Liturgusa maya*, the species name was largely used as a default species identification. This is likely due to the limited state of knowledge surrounding the species boundaries within the genus. Therefore, many of the previous records of *Liturgusa maya* cannot be accurately confirmed or falsified and using historical records for biogeographic studies or population occurrence studies should be avoided.

Five syntypes designated by Saussure and Zehntner were examined from The Natural History Museum, London, and Muséum d’Histoire naturelle, Geneva. To increase taxonomic stability within the species, under Article 74.1 of the International Code of Zoological Nomenclature a male syntype from the BMNH has been selected to become the unique bearer of the name of the nominal species-group *Liturgusa maya* (lectotype). Two syntype females from the BMNH and one additional syntype male and one additional syntype female from the MHNG all become paralectotypes under Article 74.1.3 of the Code.

An extensive search for the holotype of *Liturgusa charpentieri* was conducted in collections in Italy, but was never located (see Taxonomic History of *Liturgusa guyanensis*). However, the original description given by Giglio-Tos (1927) for *Liturgusa charpentieri* matches *Liturgusa maya* and with the expanded range of *Liturgusa maya* into South America as evidenced by specimens linking Central and South America through occurrences in Colombia, Venezuela, Ecuador and Peru, *Liturgusa charpentieri* can no longer be considered as distinct based on its southern distribution. Therefore, *Liturgusa charpentieri* Giglio-Tos, 1927, is now considered as a junior synonym to *Liturgusa maya* Saussure & Zehntner, 1894.

Interestingly, *Liturgusa maya* was included in a number of studies focused on chromosomes headed by Sally Hughes-Schrader in the 1940’s and into the 60’s.

###### Natural history.

The species has been found in wet tropical forests in Nicaragua, Costa Rica, Peru, and numerous other regions within its range. However, individuals have also been found in seasonally dry forests, open habitats as well as heavily impacted habitats such as park-land or the edges of parking lots. Perhaps the versatility of *Liturgusa maya* in habitat utilization has led to its broad geographical range that extends from mid-Mexico to southern Peru. The current thinking for why *Liturgusa* is so diverse relates to the narrow geographic ranges of species that is linked to poor dispersal ability. However, *Liturgusa maya* appears to violate this thinking. Although genitalic and external morphology are rather consistent across the entire range, genetic information may lead to the discovery of considerable new diversity by uncovering cryptic species. Without an accurate model of *Liturgusa* species origins, it can not be said whether *Liturgusa maya* is young or old relative to other *Liturgusa* species. Perhaps the species is undergoing a dispersal and speciation event that genetic information could uncover. It is suggested that a population genetics study of *Liturgusa maya* would be the logical next step to understanding how the species could have such an amazing geographic and habitat range.

As with most *Liturgusa* species, *Liturgusa maya* are adept runners and live on medium size smooth-bark tree trunks. Since they are found in wet and dry forests, some individuals have been observed on a broader diversity of tree types, some with moss or even rough bark. Size variation within *Liturgusa maya* is more extreme than any other species of *Liturgusa*. The largest female is 145% the size of the smallest, a disparity not matched in the rest of *Liturgusa* (139.5% for *Liturgusa nubeculosa*, which is a much larger species).

###### Diagnosis.

Most similar to *Liturgusa kirtlandi* and *Liturgusa trinidadensis* with a similar size, coloration and pronotum shape, *Liturgusa maya* is distinct from the other two by a number of features including male genitalia. The most obvious difference easily diagnosing *Liturgusa maya* from *Liturgusa kirtlandi* is that the apical process (paa) is elongate, thickened and the terminus forms an evenly rounded terminus rather than an angled blunt tip. In addition, *Liturgusa maya* can be differentiated from *Liturgusa trinidadensis* by the larger apofisis falloid (afa) compared to the barely present, but sharp structure seen in *Liturgusa trinidadensis*. The species has the greatest size variation across its range. The main difference between *Liturgusa maya* and *Liturgusa kirtlandi* is the presence of tubercles on the pronotum in *Liturgusa kirtlandi*.

###### Description.

**Figure 13.**
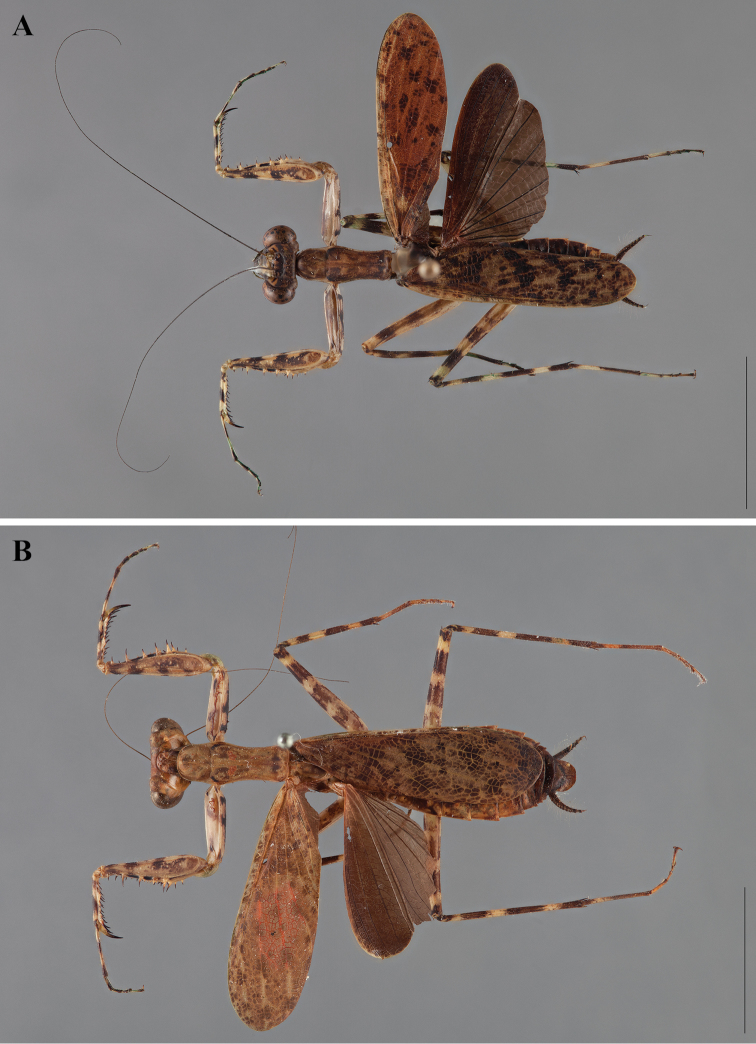
*Liturgusa maya* Saussure & Zehntner, 1894, dorsal habitus: **A** male from Nicaragua (CLEV GSMC003431) **B** female from Costa Rica (ZMUH 018).

**Figure 14.**
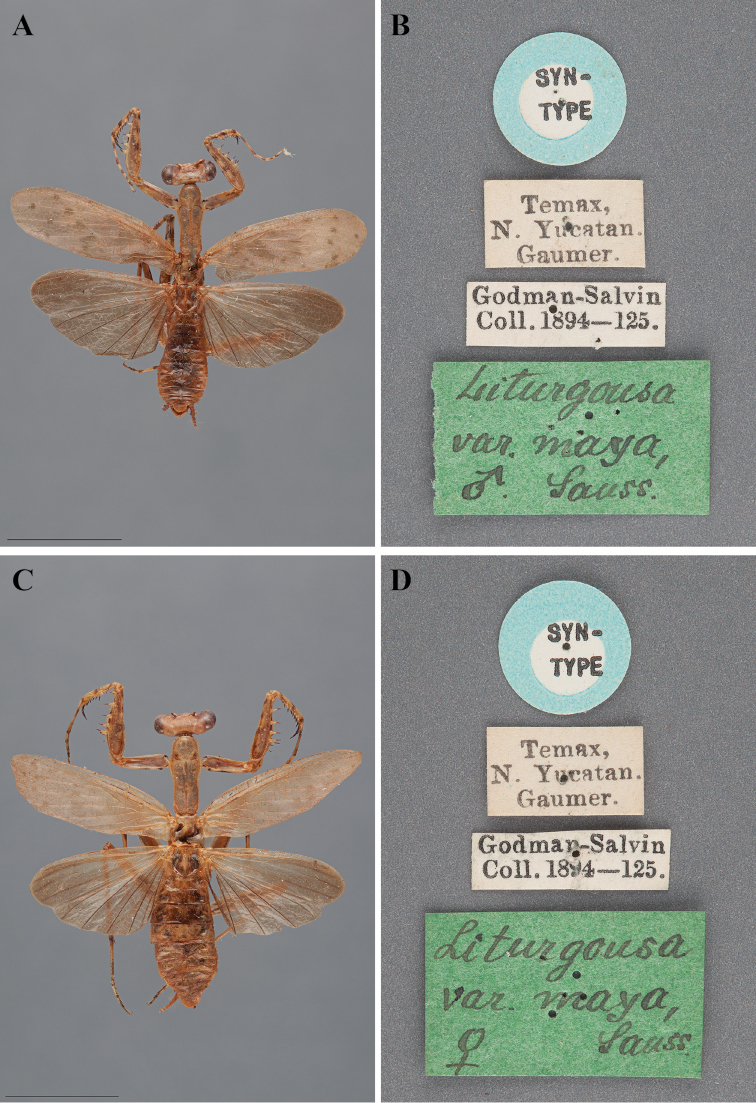
*Liturgusa maya* Saussure & Zehntner, 1894, dorsal habitus and labels: **A** lectotype male (BMNH) **B** labels of lectotype male **C** paralectotype female (BMNH) **D** labels of paralectotype female.

**Male.** (Figs 13A, 14A) N=18: Body length 19.38–25.46 (22.43); forewing length 13.16–16.56 (14.87); hindwing length 10.69–13.58 (12.28); pronotum length 5.61–7.39 (6.30); prozone length 1.68–2.36 (1.95); pronotum width 2.11–2.99 (2.44); pronotum narrow width 1.57–2.14 (1.77); head width 4.46–6.34 (4.99); head vertex to clypeus 1.79–2.67 (1.98); frons width 1.61–2.39 (1.80); frons height 0.61–0.93 (0.71); prothoracic femur length 5.40–7.42 (6.25); mesothoracic femur length 3.32–8.28 (7.34); mesothoracic tibia length 5.22–7.36 (6.10); mesothoracic tarsus length 4.58–8.07 (5.55); metathoracic femur length 6.61–9.26 (7.86); metathoracic tibia length 7.35–10.45 (8.69); metathoracic tarsus length 6.70–8.69 (7.74); pronotal elongation measure 0.30–0.33 (0.31); pronotal shape measure 0.37–0.41 (0.39); head shape measure 0.37–0.42 (0.40); frons shape measure 0.36–0.42 (0.39); anteroventral femoral spine count 14–16 (15); anteroventral tibial spine count 9–11 (10); posteroventral tibial spine count 7.

*Head* (Fig. 42A): Transverse, the juxta-ocular protuberances small, but pronounced, the apex just lateral to the midline; the vertex is straight, but sometimes dips just prior to the parietal sutures, even with the dorsal margin of the eyes. Frontal suture with a slight medial carina forming a continuous arc, the entire carina depressed into the head. Ocelli small, the central more enlarged (about twice the size as the lateral), all protruding on small cuticular mounds; the lateral ocelli oriented outward. The carina on the frons not very pronounced, the medial region just ventral to the carina depressed. Clypeus transverse, the upper margin slightly convex, the lower margin slightly concave or straight; the central, transverse carina pronounced and straight. Antennae scape and pedicel pale, the flagellum black just slightly distal to the base. Black band extending straight over the medial carina of the frontal suture, the very center of the carina pale; black markings extend ventrally and dorsally from black band; two prominent pale marks positioned just lateral to the lateral ocelli; two pale marks positioned on the lower region of the vertex. Lower region of frons darkly pigmented; the dorsal half of clypeus darkly pigmented, the ventral half pale; the mandibles and labrum with pale and brown markings; the vertex and juxta-ocular protuberances mostly black with pale speckles; the area immediately adjacent to lateral ocelli black. Palpi are pale.

*Pronotum* (Fig. 47Q): About three times long as wide with a moderately defined supra-coxal bulge; dorsal surface entirely smooth. Prozone square with slightly convex margins that gradually taper to an evenly rounded anterior margin; margins smooth or with very few blunt tubercles. Metazone with concave lateral margins without interruptions or bulges; margins with small tubercles; posterior margin with a medial emargination; the dorsal surface of the posterior third of the metazone slightly depressed. Mostly dark with pale and black marking across the surface, faint swirls present on the metazone just posterior to the supra-coxal sulcus.

*Prothoracic Legs*: Femur robust with a slightly concave dorsal margin; strongly defined pale to dark banding on posterior (external) surface; anterior (internal) surface with a black band running medially from the base to terminus that may be interrupted, various black marks present in addition to the band that correspond to banding marks on the posterior surface; the ventral surface pale. Posterior surface of femur with few tubercles. A femoral pit to accommodate terminal posteroventral tibial spine positioned medial to and exactly between the first two proximal posteroventral spines, in line with the most distal discoidal spine; pit is pigmented darkly. Posterior prothoracic femoral genicular spine smaller than posteroventral spines (highly variable), originating distal to the beginning of the genicular lobe. Prothoracic tibial posteroventral spines with the first (proximal) smallest and the third through sixth of similar length, the second slightly longer. Prothoracic coxae smooth, the anterior surface with a very small, black mark medially in the proximal half as well as a very small black spot medially towards the distal terminus.

*Meso- and Metathoracic Legs*: Femora with ventral (posterior) carina; dorsal (anterior) carina present. Mesotarsi with first segment as long or slightly longer than the remaining segments combined.

*Wings*: Forewings mottled with brown, pale and greenish coloration; the costal region with defined banding, green and brown alternating markings, the brown marks smaller; vein coloration mostly corresponding with surrounding colors; two pale spots are positioned in the proximal quarter of the discoidal region just posterior to the first radial vein; a large pale area is positioned centrally. Forewings often, but not always asymmetrically colored; one being mottled as described the other is darkened significantly with a black or rust tone, the mottled pattern still visible; extending just beyond or as long as the abdomen. Hindwings with opaque discoidal region, colored rust proximally and along the anterior margin, otherwise black; the anal region smoky black and translucent; the terminus of the discoidal region projecting beyond the distal margin of anal region, the wing appearing elongate.

*Abdomen*: Slightly widened in the middle, the fourth tergite the widest region before a gradual posterior narrowing; a smooth, brown and black colored dorsal surface. Tergites without posterolateral tergal projections. Supra-anal plate slightly transverse, a broadly rounded terminus. Subgenital plate irregularly rounded and without styli.

*Genital Complex* (Fig. 51I.1–I.4): The main body of ventral left sclerite (L4A) with rounded terminus, but with a distal process (pda) positioned just lateral to the midline that is rounded (could be short and rather blunt or more elongate and narrow), projecting at an angle, appearing like a small, well-sclerotized tooth; sometimes a depression on the opposite lateral half from the pda is present. The apofisis falloid (afa) of the main body of dorsal left sclerite (L4B) short, quickly narrowing to a sharp point, sometimes curved and sometimes with a rough surface; the apical process (paa) elongate and thin, the terminus an evenly rounded end. The right dorsal phallomere (fda) of the first sclerite of right phallomere (R1) tapers to a rounded, membranous terminus; the ventral plate (pia) long, broad proximally with strongly defined grooves; the ventral process (pva) tooth-like and curved at the proximal base, the distal tip narrowing with a rapid constriction towards the end.

###### Redescription.

**Female.** (Figs 12B, 13B, 14C) N=28: Body length 24.02–33.46 (27.24); forewing length 14.55–21.12 (16.95); hindwing length 12.02–15.61 (13.82); pronotum length 6.75–8.99 (7.46); prozone length 2.08–2.83 (2.31); pronotum width 2.74–3.55 (2.97); pronotum narrow width 1.89–2.57 (2.16); head width 5.26–6.94 (5.94); head vertex to clypeus 2.20–2.88 (2.49); frons width 2.03–2.75 (2.30); frons height 0.76–0.94 (0.85); prothoracic femur length 6.73–8.48 (7.31); mesothoracic femur length 7.64–10.25 (8.55); mesothoracic tibia length 5.74–8.19 (6.77); mesothoracic tarsus length 4.57–7.66 (5.9); metathoracic femur length 7.76–10.13 (8.53); metathoracic tibia length 8.41–11.21 (9.68); metathoracic tarsus length 7.38–11.03 (8.53); pronotal elongation measure 0.30–0.33 (0.31); pronotal shape measure 0.38–0.41 (0.40); head shape measure 0.38–0.46 (0.42); frons shape measure 0.34–0.41 (0.37); anteroventral femoral spine count 14–16 (15); anteroventral tibial spine count 7–10 (10); posteroventral tibial spine count 7.

*Head* (Fig. 42B): About as broad as long, the juxta-ocular protuberances large; the vertex higher than the dorsal margin of the eyes.

*Pronotum* (Fig. 47R): The dorsal surface of the posterior third of the metazone not depressed.

*Prothoracic Legs*: As described for males.

*Meso- and Metathoracic Legs*: Mesotarsi with first segment shorter than the remaining segments combined.

*Wings*: The costal region of forewing with less defined banding, proximal region mostly mottled with brown and pale. Forewings extending just shy of the terminus of the abdomen, the last few segments and supra-anal plate mostly visible.

*Abdomen*: Widened, the fifth tergite the widest region before a gradual posterior narrowing. Tergites without posterolateral tergal projections. Supra-anal plate about as long as wide, rounded terminus.

##### Liturgusa stiewei sp. n.

http://zoobank.org/0EB1C2D3-98D5-4315-989F-7DFB5D319504

http://species-id.net/wiki/Liturgusa_stiewei

Liturgousa mesopoda : Hebard 1919b: 134.

###### Type.

Holotype Female, pinned. Academy of Natural Sciences of Drexel University, Philadelphia, PA, USA.

###### Type locality.

Jimenez, W. Colombia, elev. 1600ft. III 1907, M.G. Palmer (Lat. 2.675931, Long. -77.148414)

###### Material examined.

*Liturgusa stiewei* sp. n.

**Table:** 

Sex	Type	Country	Label	Latitude Longitude	Code
Female	Holotype	Colombia	Jimenez, W. Colombia, elev. 1600ft. III 1907, M.G. Palmer	2.675931, -77.148414	ANSP 017
Male	Allotype	Colombia	Santander Province	6.641759, -73.653934	MSMC
Female	Paratype	Colombia	ANTIOQUIA: Maceo, to 1600 masl, ♀, 16-XII-2003, E. Henao leg (CJASE, Manizales)	6.550551, -74.778359	CJASE

###### Diagnosis.

One of the largest species of *Liturgusa*, comparable to *Liturgusa nubeculosa*, *Liturgusa stiewei* is more comparable to many large species of *Hagiomantis*. Can be distinguished from *Liturgusa nubeculosa* by its shorter, more broad pronotum and black, more opaque hindwings.

###### Description.

**Figure 15.**
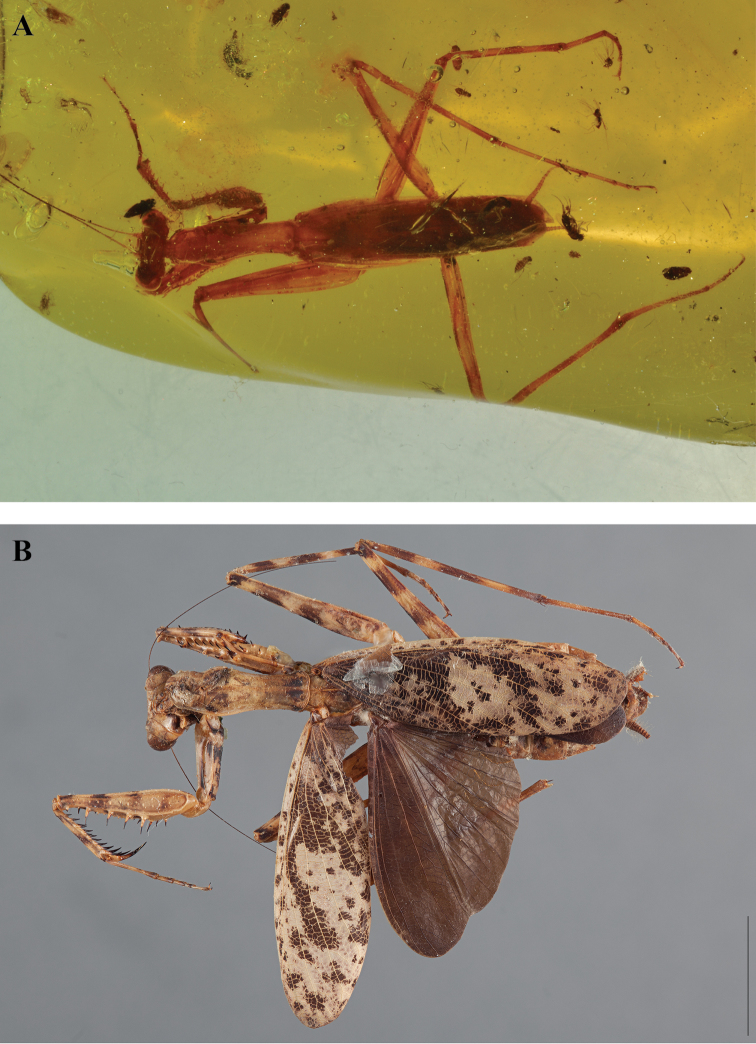
*Liturgusa stiewei* sp. n., dorsal habitus: **A** allotype male in copal (MSMC) **B** holotype female from Colombia (ANSP 017).

**Male from copal (restricted access to some characters**). (Fig. 15A) N=1: Body length 30.70; forewing length 19.10; pronotum length 9.18; prozone length 3.10; pronotum width 3.37; pronotum narrow width 2.33; head width 5.90; prothoracic femur length 7.75; mesothoracic femur length 12.14; mesothoracic tibia length 10.07; mesothoracic tarsus length 7.30; metathoracic femur length 12.96; metathoracic tibia length 13.20; metathoracic tarsus length 13.12; pronotal elongation measure 0.34; pronotal shape measure 0.37; anteroventral femoral spine count 14; anteroventral tibial spine count 10; posteroventral tibial spine count 7.

*Head*: Transverse, the juxta-ocular protuberances small, but pronounced, the apex in the middle; the vertex near straight, even with the dorsal margin of the eyes. Frontal suture with a slight medial carina forming a continuous arc. Ocelli small. Frons, clypeus and labrum not visible for description. Palpi are pale.

*Pronotum* (Fig. 48A): A little less than three times as long as wide with a moderately defined supra-coxal bulge; dorsal surface smooth, without tubercles. Prozone broader than long, with parallel margins prior to rounded anterolateral corners, the anterior margin broadly straight; margins smooth or with very few broad, blunt tubercles. Metazone with concave lateral margins with near parallel margins just posterior to the middle; margins with numerous small tubercles; posterior margin with a shallow medial emargination; the dorsal surface of the posterior third of the metazone depressed. Coloration largely unknown except for two symmetrical dark marks on the anterior corners of the metazone.

*Prothoracic Legs*: Femur robust with a slightly concave dorsal margin; pale to dark banding not well defined on posterior (external) surface; anterior (internal) surface mostly not visible, but the distal half with a very thin black band running medially; the ventral surface pale. A shallow femoral pit to accommodate terminal posteroventral tibial spine positioned medial to and between the first two proximal posteroventral spines, in line with the most distal discoidal spine; coloration of pit not visible. Posterior prothoracic femoral genicular spine much smaller than posteroventral spines, originating at the beginning of the genicular lobe. Prothoracic tibial posteroventral spines with the first (proximal) smallest and the third through sixth of similar length, the second longer. Prothoracic coxae not visible.

*Meso- and Metathoracic Legs*: Femora with ventral (posterior) carina; dorsal (anterior) carina present. Mesotarsi with first segment shorter than the remaining segments combined.

*Wings*: Forewings mottled with brown and pale coloration; the costal region with faintly defined banding; vein coloration is pale, not matching surrounding cell coloration. Hindwings are hidden in examined specimen.

*Abdomen*: Slender, the fifth tergite the widest region before a gradual posterior narrowing. Subgenital plate irregularly rounded and without styli.

*Genital Complex*: Only male in copal.

**Female.** (Fig. 15B) N=2: Body length 41.94–42.00 (41.97); forewing length 26.10–26.70 (26.40); hindwing length 21.20; pronotum length 11.14–11.15 (11.14); prozone length 2.60–3.16 (2.88); pronotum width 4.16; pronotum narrow width 2.97; head width 7.24; head vertex to clypeus 3.16; frons width 2.81; frons height 1.06; prothoracic femur length 11.86–12.10 (11.98); mesothoracic femur length 14.59; mesothoracic tibia length 9.53; metathoracic femur length 13.70–14.00 (13.85); metathoracic tibia length 15.67; metathoracic tarsus length 13.20–13.42 (13.31); pronotal elongation measure 0.23–0.28 (0.26); pronotal shape measure 0.37; head shape measure 0.44; frons shape measure 0.38; anteroventral femoral spine count 16; anteroventral tibial spine count 10; posteroventral tibial spine count 7.

*Head* (Fig. 42F): Longer than broad, the juxta-ocular protuberances very large, the apex in the middle; the vertex slightly concave or straight, above the dorsal margin of the eyes. Frontal suture without a carina, a black pigmented depression just ventral to medial region of suture, two depressed pits dorsally and symmetrically position lateral to the medial line that extend to broadly defined depressions dorsally towards the vertex. Ocelli small, all three of similar size and protruding on small cuticular mounds; the lateral ocelli oriented outward. The carina on the frons strongly pronounced, centrally elevated, the medial region just ventral to the carina strongly sloped ventrally. Clypeus transverse, the upper margin slightly straight medially and curving near dorso-lateral corners; the lower margin concave or straight; the central, transverse carina pronounced and straight. Antennae scape pale, pedicel mostly pale with faint brown marks, the flagellum fading to black in the proximal quarter. Black markings extend laterally to the margin of the eyes from ventral depression abutting frontal suture. Vertex and juxta-ocular protuberances mostly pale with brown and black speckling; region between ocelli pale except for a thin band running along medial line from the frontal suture; lower region of frons pigmented brown; the dorsolateral corners of clypeus with brown spots, the rest is pale; the mandibles and labrum with pale and brown markings; the area immediately adjacent to lateral ocelli black. Palpi are pale.

*Pronotum* (Fig. 48B): About 2.7 times long as wide with a moderately defined supra-coxal bulge; dorsal surface smooth, without tubercles. Prozone is broader than long with gradually tapering margins leading to an evenly rounded anterior margin; margins smooth. Metazone with concave lateral margins, the medial region flat or bulging outward; margins with very small tubercles; posterior margin with a very shallow medial emargination; the dorsal surface of the posterior third of the metazone depressed. Mostly pale or light brown with brown and black marking across the surface; two prominent black marks present in the posterolateral corners; two symmetrically positioned black marks on either side of midline and just anterior to the posterior margin; two symmetrically positioned lateral marks just posterior of supra-coxal sulcus.

*Prothoracic Legs*: Femur normal with a slightly concave dorsal margin; strongly defined pale to dark banding on posterior (external) surface; anterior (internal) surface with a strongly defined black band running medially from the base to terminus, some regions slightly widened, particularly near the femoral brush; the ventral surface pale. Posterior surface of femur with few tubercles. A large femoral pit to accommodate terminal posteroventral tibial spine positioned medial to and between the first two proximal posteroventral spines, in line with the most distal discoidal spine; pit is black. Posterior prothoracic femoral genicular spine tiny compared to posteroventral spines, originating at the beginning of the genicular lobe. Prothoracic tibial posteroventral spines with the first (proximal) and fourth through sixth of similar length, the second and third longer. Prothoracic coxae smooth, the anterior surface with a large medially positioned black band in the proximal half as well as a similar black band in the distal half.

*Meso- and Metathoracic Legs*: Femora with ventral (posterior) carina; dorsal (anterior) carina present. Mesotarsi with first segment shorter than the remaining segments combined.

*Wings*: Forewings mottled with brown, pale and black coloration; the costal region mostly pale with some regularly spaced black marks; vein coloration pale; discoidal area with highly contrasting regions, evenly mottled proximally with a distinct shift to mostly pale in the distal half. Forewings symmetrically colored; extending just short of the terminus of the abdomen, the supra-anal plate visible. Hindwings entirely opaque black; the terminus of the discoidal region projecting beyond the distal margin of anal region, the wing appearing slightly elongate.

*Abdomen*: Widened, the fourth tergite the widest before a gradual posterior narrowing; a smooth surface with light brown coloration. Tergites without posterolateral tergal projections, if present they are very small. Supra-anal plate transverse, an evenly rounded terminus.

###### Etymology.

A noun in the genitive case, *Liturgusa stiewei* is named for Martin Stiewe in honor of his contributions to Mantodea systematics and his collaboration in discovering this new species.

##### Liturgusa tessae sp. n.

http://zoobank.org/A7A78A8F-1162-42E3-A59D-DBC889D5C7F9

http://species-id.net/wiki/Liturgusa_tessae

###### Type.

Holotype Male, pinned. Cleveland Museum of Natural History, Cleveland, OH, USA

###### Type locality.

Bolivia: La Paz Dept., Ituralde Prov., San Miguel, 14°30.602'S, 67°29.555'W, 24-30 Sept 2007, Coll: K.B. Miller et al. (Lat. -14.510033, Long. -67.492583)

###### Material examined.

*Liturgusa tessae* sp. n.

**Table:** 

Sex	Type	Country	Label	Latitude Longitude	Code
Male	Holotype	Bolivia	La Paz Dept., Ituralde Prov., San Miguel, 14°30.602'S, 67°29.555'W, 24-30 Sept 2007, Coll: K.B. Miller et al.	-14.510033, -67.492583	GSMC000263
Female	Allotype	Peru	Smithsonian Earthwatch Sample, Location 8j, Nickle, D.		GSMC000265
Female	Paratype	Brazil	Santarem, July. 1919, S.M. Klages. Acct. 6324	-2.465914, -54.701313	ANSP 111
Female	Paratype	Brazil	Santarem, July. 1919, S.M. Klages. Acct. 6324	-2.465914, -54.701313	ANSP 108
Male	Paratype	Brazil	Santarem, July. 1919, S.M. Klages. Acct. 6324	-2.465914, -54.701313	ANSP 109
Male	Paratype	Brazil	Santarem, July. 1919, S.M. Klages. Acct. 6324	-2.465914, -54.701313	ANSP 110
Female	Paratype	Brazil	Santarem, July. 1919, S.M. Klages. Acct. 6324	-2.465914, -54.701313	ANSP 112
Female	Paratype	Brazil	Santarem, Oct. 1919, Carn. Mus. Acc. 6543	-2.465914, -54.701313	ANSP 113
Male	Paratype	Brazil	50, 2		BMNH 002
Male	Paratype	Brazil	52, 96		BMNH 005
Female	Paratype	Peru	Rio Tambopata, Explorer‘s Inn-Rio Tower, 12°50.208'S, 069°17.603'W, 10-XII-2003, Svenson	-12.836800, -69.293383	GSMC000261
Male	Paratype	Bolivia	La Paz Dept., Ituralde Prov., San Miguel, 14°30.602'S, 67°29.555'W, 24-30 Sept 2007, Coll: K.B. Miller et al.	-14.510033, -67.492583	GSMC000268
Female	Paratype	Brazil	Amazonas Reserva Biologica do Cuieiras, 50 km. N. Manaus, 15-IV-AU 15-V-1981, M. Descamps	-2.599556, -60.210631	MNHN 020
Male	Paratype	Brazil	Para, Ile de Mosqueiro, 11-9-83, male + female accouples sur trone	-1.157643, -48.465362	MNHN 086
Female	Paratype	Brazil	Para, Ile de Mosqueiro, 11-9-83, male + female accouples sur trone	-1.157643, -48.465362	MNHN 087
Male	Paratype	Brazil	Para: Rio Xingu, Camp (52°22'W, 3°39'S), ca 60 km S. Altamira, 8-12 Oct 1986, P. Spangler & O. Flint	-3.650000, -52.366667	USNM 007; USNM ENT 00873005
Male	Paratype	Brazil	Para: Rio Xingu, Camp (52°22'W, 3°39'S), ca 60 km S. Altamira, 8-12 Oct 1986, P. Spangler & O. Flint	-3.650000, -52.366667	USNM 008; USNM ENT 00873006
Male	Paratype	Brazil	Para.		ZMHB 004
Male	Paratype	Peru	Rio Tambopata Res., 30 km (air) SW Pto. Maldonado, 290 m, 12°50'S, 69°17'W; Smithsonian Institution Canopy Fogging Project, T.L. Erwin et al., colls. 07 May 89, 05/02/051	-12.833333, -69.283333	USNM 069; USNM ENT 00873048
Female	Paratype	Peru	Madre de Dios, Rio Tambopata Res., 30 km (air) SW Pto. Maldonado, 290 m, 12°50'S, 69°17'W; Smithsonian Institution Canopy Fogging Project, T.L. Erwin et al., colls. 08 Nov 83 May 89, 04/01/072	-12.833333, -69.283333	USNM 070; USNM ENT 00873049
Male	Paratype	Bolivia	La Paz Dept., Ituralde Prov., San Miguel, 14°30.602'S, 67°29.555'W, 24-30 Sept 2007, Coll: K.B. Miller et al.	-14.510033, -67.492583	GSMC000317

###### Diagnosis.

Very similar to *Liturgusa maya* in size and shape, but with an even color patterning on the forewings, lacking highly contrasting regions. The hindwings for *Liturgusa tessae* are very rusty colored in males and pale to rusty colored in females along the anterior half of the discoidal region. The male genitalia are distinct in that the ventral left sclerite (L4A) is elongate, the terminus tapers narrowly before giving rise to a short, blunt distal process (pda).

###### Description.

**Figure 16.**
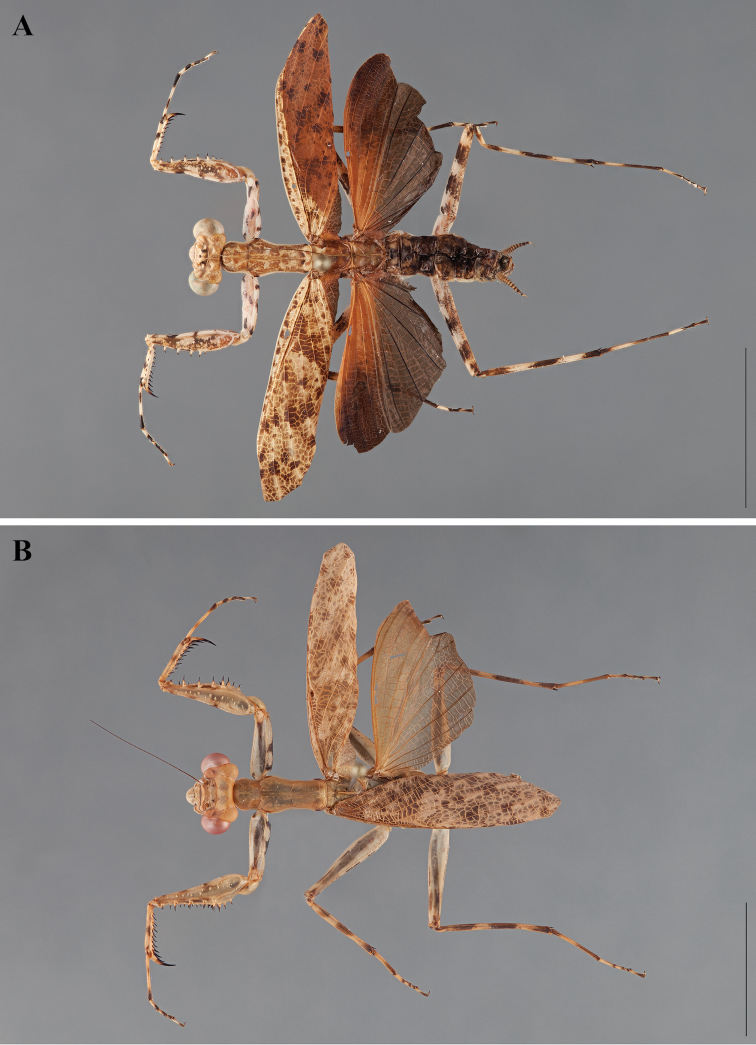
*Liturgusa tessae* sp. n., dorsal habitus: **A** holotype male from San Miguel, Bolivia (CLEV GSMC000263) **B** allotype female from Tambopata, Peru (CLEV GSMC000265).

**Male.** (Fig. 16A) N=9: Body length 19.23–23.89 (22.12); forewing length 13.88–15.21 (14.61); hindwing length 10.95–12.13 (11.47); pronotum length 5.71–6.65 (6.13); prozone length 1.68–2.00 (1.82); pronotum width 2.36–2.62 (2.48); pronotum narrow width 1.64–1.92 (1.76); head width 4.70–5.51 (5.03); head vertex to clypeus 1.90–2.16 (1.97); frons width 1.64–1.95 (1.77); frons height 0.61–0.69 (0.65); prothoracic femur length 5.73–6.64 (6.12); mesothoracic femur length 6.92–8.47 (7.67); mesothoracic tibia length 5.54–6.65 (5.97); mesothoracic tarsus length 4.84–5.70 (5.17); metathoracic femur length 7.23–8.63 (7.80); metathoracic tibia length 7.78–9.23 (8.27); metathoracic tarsus length 7.25–8.35 (7.61); pronotal elongation measure 0.29–0.31 (0.30); pronotal shape measure 0.39–0.43 (0.41); head shape measure 0.37–0.40 (0.39); frons shape measure 0.35–0.42 (0.37); anteroventral femoral spine count 14–15 (14); anteroventral tibial spine count 9–10 (10); posteroventral tibial spine count 7.

*Head* (Fig. 42C): Transverse, the juxta-ocular protuberances small, the apex in the lateral half; the vertex slightly concave, slightly above the dorsal margin of the eyes. Frontal suture with a slight medial carina forming a continuous arc. Ocelli small, the central slightly larger than lateral two, all three protruding on small cuticular mounds; the lateral ocelli oriented outward. The carina on the frons thin, the medial region just ventral to the carina depressed. Clypeus transverse, the upper margin slightly convex, the lower margin slightly concave or straight; the central, transverse carina pronounced and straight. Antennae scape and pedicel pale, the flagellum fading to black in the proximal quarter. Black band extending straight over the medial carina of the frontal suture, the carina pale; black markings extend ventrally and dorsally from black band; two prominent pale marks positioned just lateral to the lateral ocelli; two pale marks positioned on the medial region of the vertex. Lower region of frons pigmented brown; the dorsolateral corners of clypeus with brown spots, the rest is pale; the mandibles and labrum with pale and brown markings; the vertex and juxta-ocular protuberances mostly pale with brown or black speckling; the area immediately adjacent to lateral ocelli black. Palpi are pale.

*Pronotum* (Fig. 47S): About 2.5 times long as wide with a moderately defined supra-coxal bulge; dorsal surface with very small, disperse tubercles in the posterior half. Prozone a little broader than long with near parallel margins that gradually taper to an evenly rounded anterior margin; margins with few blunt tubercles. Metazone with concave lateral margins, the medial region flat or slightly bulging outward; margins with small tubercles; posterior margin with a shallow medial emargination; the dorsal surface of the posterior third of the metazone depressed. Mostly pale or light brown with brown and black markings across the surface, two prominent black marks present in the posterolateral corners.

*Prothoracic Legs*: Femur normal with a slightly concave dorsal margin; strongly defined pale to dark banding on posterior (external) surface; anterior (internal) surface with a black mark near the base and a medial black band beginning in the distal half and terminating near the end of the femur; the ventral surface pale. Posterior surface of femur with few tubercles. A shallow femoral pit to accommodate terminal posteroventral tibial spine positioned medial to and between the first two proximal posteroventral spines, in line with the most distal discoidal spine; pit is pale. Posterior prothoracic femoral genicular spine smaller than posteroventral spines (highly variable), originating distal to the beginning of the genicular lobe. Prothoracic tibial posteroventral spines with the first (proximal) smallest and the third through sixth of similar length, the second slightly longer. Prothoracic coxae smooth, the anterior surface with a black mark medially in the proximal half.

*Meso- and Metathoracic Legs*: Femora with ventral (posterior) carina; dorsal (anterior) carina present. Mesotarsi with first segment as long or slightly shorter than the remaining segments combined.

*Wings*: Forewings mottled with brown, pale and greenish coloration; the costal region without strongly defined banding, mostly pale with dispersed brown markings; vein coloration matching the corresponding surrounding cell colors; lacking noticeable central or proximal pale markings, the wing having evenly mottled coloration that is not highly contrasting. Forewings asymmetrically colored; one being mottled as described the other is darkened with a rust tone, the mottled pattern still visible; extending just beyond the abdomen. Hindwings rust colored (more pale proximally), the discoidal region more opaque, but translucent near the boundary with the anal region; the anal region translucent and rusty colored; the terminus of the discoidal region projecting beyond the distal margin of anal region, the wing appearing elongate.

*Abdomen*: Slightly widened in the middle, the fourth tergite the widest region before a gradual posterior narrowing; a smooth, light brown or rust coloration. Tergites without posterolateral tergal projections. Supra-anal plate slightly transverse, a broadly rounded terminus. Subgenital plate irregularly rounded and without styli.

*Genital Complex* (Fig. 51J.1–J.6): The main body of ventral left sclerite (L4A) elongate, the terminus tapering to a narrow point; a short, blunt distal process (pda) is centrally positioned; the right margin in the distal quarter highly sclerotized, extending to the terminus of the pda; an elliptical depression on left half is present. The apofisis falloid (afa) of the main body of dorsal left sclerite (L4B) short, quickly narrowing to a sharp point, the lower margin concave and often irregular, leading to a secondary process that is short and rounded; the apical process (paa) broad and elongate, tapering to a narrow and rounded terminus. The right dorsal phallomere (fda) of the first sclerite of right phallomere (R1) broad, tapering slightly to a rounded, blunt and membranous terminus; the ventral plate (pia) long, broad proximally with strongly defined grooves; the ventral process (pva) enlarged, c-shaped with a smooth surface, the distal tip broad and rounded.

**Female.** (Fig. 16B) N=7: Body length 23.65–29.63 (26.24); forewing length 14.71–18.43 (16.76); hindwing length 12.34–13.05 (12.7); pronotum length 6.77–7.86 (7.10); prozone length 1.99–2.27 (2.12); pronotum width 2.74–3.16 (2.93); pronotum narrow width 1.99–2.37 (2.14); head width 5.65–6.34 (5.98); head vertex to clypeus 2.35–2.64 (2.51); frons width 2.20–2.55 (2.38); frons height 0.74–0.98 (0.84); prothoracic femur length 6.68–7.83 (7.08); mesothoracic femur length 7.72–9.45 (8.33); mesothoracic tibia length 5.87–7.49 (6.57); mesothoracic tarsus length 5.23–6.39 (5.68); metathoracic femur length 7.80–9.66 (8.54); metathoracic tibia length 8.46–10.72 (9.46); metathoracic tarsus length 7.89–9.15 (8.34); pronotal elongation measure 0.29–0.31 (0.30); pronotal shape measure 0.39–0.43 (0.41); head shape measure 0.41–0.44 (0.42); frons shape measure 0.33–0.39 (0.35); anteroventral femoral spine count 14–15 (14); anteroventral tibial spine count 10; posteroventral tibial spine count 7.

*Head* (Fig. 42D): Slightly transverse, the juxta-ocular protuberances large, the apex just lateral of the middle; the vertex concave, above the dorsal margin of the eyes. Ocelli positioned on a small carina connecting all three. Antennae scape and pedicel pale, the flagellum fading to black by the middle. In addition to the black band extending straight over the medial carina, the anterior face, vertex and juxta-ocular protuberances mottled with pale, brown and black markings. Lower region of frons pigmented brown or light brown; the clypeus, mandibles, and labrum with pale and brown markings.

*Pronotum* (Fig. 47T): As described for males.

*Prothoracic Legs*: Anterior (internal) surface of femur with a black mark near the base and a medial black band that may be interrupted or degraded beginning in the distal half and terminating near the end of the femur. A shallow femoral pit that is pigmented brown or pale.

*Meso- and Metathoracic Legs*: As described for males.

*Wings*: Forewings may be asymmetrically colored like in males; extending almost to the terminus of the abdomen, but the supra-anal plate still visible. Hindwings smoky colored with a rusty base and anterior margin, opaque; the terminus of the discoidal region projecting a little beyond the distal margin of anal region, the wing appearing moderately elongate.

*Abdomen*: Moderately widened.

###### Etymology.

A noun in the genitive case, *Liturgusa tessae* is named for my daughter Tessa Eliza Svenson.

##### Liturgusa trinidadensis sp. n.

http://zoobank.org/56891F11-5672-4A7F-9D93-B6C0F4CCD515

http://species-id.net/wiki/Liturgusa_trinidadensis

Liturgousa maya (*partim*): Rehn 1935: 202.

###### Type.

Holotype Male, pinned. Academy of Natural Sciences of Drexel University, Philadelphia, PA, USA.

###### Type locality.

Trinidad, 17 I‚ 52, F. Schrader, 709 (Lat. 10.240326, Long. -61.217020).

###### Material examined.

*Liturgusa trinidadensis* sp. n.

**Table:** 

Sex	Type	Country	Label	Latitude Longitude	Code
Male	Holotype	Trinidad	17 I ‚52, F. Schrader, 709	10.240326, -61.217020	ANSP 035
Female	Allotype	Trinidad	Caparo, VIII 1913, S.M. Klages	10.449908, -61.333566	ANSP 099
Male	Paratype	Trinidad	W.I., Trinidad: Arima Vall. A.W.N.C. 7/15/78, R.A. Mendez	10.661851, -61.289723	AMNH 029
Female	Paratype	Trinidad	10 I ‚52, F. Schrader, 697	10.240326, -61.217020	ANSP 001
Male	Paratype	Trinidad	18 I ‚52, F. Schrader, 715	10.240326, -61.217020	ANSP 002
Male	Paratype	Trinidad	17 I ‚52, F. Schrader, 711	10.240326, -61.217020	ANSP 003
Male	Paratype	Trinidad	18 I ‚52, F. Schrader, 723	10.240326, -61.217020	ANSP 004
Male	Paratype	Trinidad	11 I ‚52, F. Schrader, 781	10.240326, -61.217020	ANSP 005
Female	Paratype	Trinidad	18 I ‚52, F. Schrader, 725	10.240326, -61.217020	ANSP 006
Female	Paratype	Trinidad	18 I ‚52, F. Schrader, 724	10.240326, -61.217020	ANSP 007
Male	Paratype	Trinidad	17 I ‚52, F. Schrader, 710	10.240326, -61.217020	ANSP 008
Male	Paratype	Trinidad	17 I ‚52, F. Schrader, 712	10.240326, -61.217020	ANSP 009
Male	Paratype	Trinidad	15 I ‚52, F. Schrader, 699	10.240326, -61.217020	ANSP 014
Male	Paratype	Trinidad	18 I ‚52, F. Schrader, 722	10.240326, -61.217020	ANSP 015
Male	Paratype	Trinidad	17 I ‚52, F. Schrader, 713	10.240326, -61.217020	ANSP 022
Female	Paratype	Trinidad	17 I ‚52, F. Schrader, 714	10.240326, -61.217020	ANSP 023
Female	Paratype	Trinidad	Arima Valley, B.W.I. 6-II-1952, Tropical Research Station, New York Zool Society	10.661851, -61.289723	ANSP 024
Male	Paratype	Trinidad	18 I ‚52, F. Schrader, 721	10.240326, -61.217020	ANSP 026
Female	Paratype	Trinidad	Arima Valley, B.W.I. 7-II-1952, Tropical Research Station, New York Zool Society	10.661851, -61.289723	ANSP 027
Female	Paratype	Trinidad	Arima Valley, B.W.I. 10-II-1952, Tropical Research Station, New York Zool Society	10.661851, -61.289723	ANSP 028
Male	Paratype	Trinidad	Arima Valley, B.W.I. 10-II-1952, Tropical Research Station, New York Zool Society	10.661851, -61.289723	ANSP 029
Male	Paratype	Trinidad	Arima Valley, B.W.I. 7-II-1952, Tropical Research Station, New York Zool Society	10.661851, -61.289723	ANSP 030
Female	Paratype	Trinidad	19 I ‚52, F. Schrader, 717	10.240326, -61.217020	ANSP 032
Male	Paratype	Trinidad	Nov. 1940, St. Augustine, BWI, H.S. Darling	10.659048, -61.398679	ANSP 034
Male	Paratype	Trinidad	Arima Valley, B.W.I. 7-II-1952, Tropical Research Station, New York Zool Society	10.661851, -61.289723	ANSP 036
Male	Paratype	Trinidad	Arima Valley, B.W.I. 10-II-1952, Tropical Research Station, New York Zool Society	10.661851, -61.289723	ANSP 037
Male	Paratype	Trinidad	Caparo, VI 1913, S.M. Klages	10.449908, -61.333566	ANSP 083
Male	Paratype	Trinidad	Caparo, VI 1913, S.M. Klages	10.449908, -61.333566	ANSP 084
Male	Paratype	Trinidad	Caparo, VI 1913, S.M. Klages	10.449908, -61.333566	ANSP 085
Female	Paratype	Trinidad	Caparo, VIII 1913, S.M. Klages	10.449908, -61.333566	ANSP 086
Female	Paratype	Trinidad	Caparo, VIII 1913, S.M. Klages	10.449908, -61.333566	ANSP 095
Female	Paratype	Trinidad	Caparo, VIII 1913, S.M. Klages	10.449908, -61.333566	ANSP 096
Female	Paratype	Trinidad	Caparo, VI 1913, S.M. Klages	10.449908, -61.333566	ANSP 097
Male	Paratype	Trinidad	Moruga Area, ii-iii.1986, G.B. Popov	10.089796, -61.279058	BMNH 081
Female	Paratype	Trinidad	Moruga Area, ii-iii.1986, G.B. Popov	10.089796, -61.279058	BMNH 082
Male	Paratype	Trinidad	Moruga Area, ii-iii.1986, G.B. Popov	10.089796, -61.279058	BMNH 083
Female	Paratype	Trinidad	Moruga Area, ii-iii.1986, G.B. Popov	10.089796, -61.279058	BMNH 084
Male	Paratype	Trinidad	Arima Valley, B.W.I. 20-II-1952, Tropical Research Station, New York Zool Society	10.661851, -61.289723	USNM 017; USNM ENT 00873007
Female	Paratype	Trinidad	Arima Valley, B.W.I. 8-II-1952, Tropical Research Station, New York Zool Society	10.661851, -61.289723	USNM 018; USNM ENT 00873008
Male	Paratype	Trinidad	Jun WI, Aug. Busck Collector		USNM 019; USNM ENT 00873009
Male	Paratype	Trinidad	Jun WI, Aug. Busck Collector		USNM 020; USNM ENT 00873015
Male	Paratype	Trinidad	Aug-22-1907, O.W. Barria, On Cacao, 252		USNM 052; USNM ENT 00873016

###### Diagnosis.

Most similar to *Liturgusa maya* and *Liturgusa kirtlandi*, exhibiting similar size, coloration and pronotum shape, *Liturgusa trinidadensis* is distinct from the other two by a few characteristics. First, the supra-anal plate in both males and females is nearly square with a broad and blunt terminus. Second, the central ocellus is the same size as the lateral two while *Liturgusa maya* and *Liturgusa kirtlandi* have larger central ocelli. Finally, *Liturgusa trinidadensis* has numerous tubercles in the posterolateral corners of the metazone. The species is also entirely restricted to the island of Trinidad, not extending into mainland South America.

###### Description.

**Figure 17.**
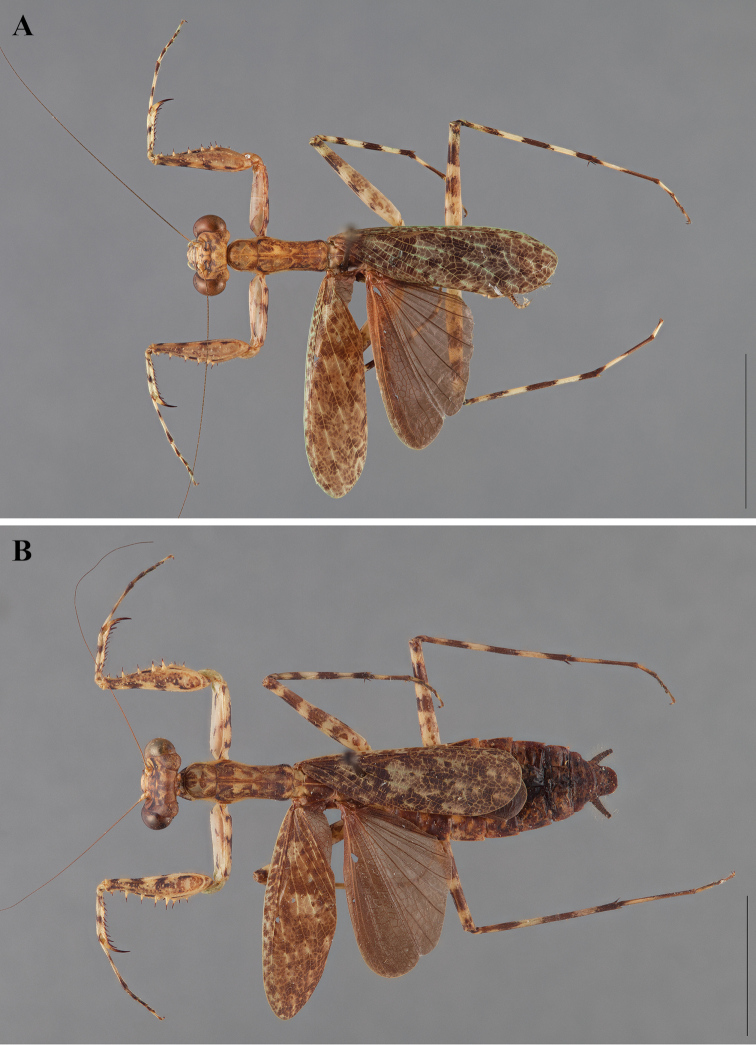
*Liturgusa trinidadensis* sp. n., dorsal habitus: **A** holotype male from Trinidad (ANSP 035) **B** allotype female from Caparo, Trinidad (ANSP 099).

**Male.** (Fig. 17A) N=15: Body length 20.29–23.06 (21.63); forewing length 13.29–15.43 (14.09); hindwing length 11.61–11.90 (11.75); pronotum length 5.89–6.85 (6.19); prozone length 1.73–2.11 (1.87); pronotum width 2.30–2.80 (2.42); pronotum narrow width 1.53–2.06 (1.74); head width 4.70–5.43 (5.01); head vertex to clypeus 1.87–2.23 (2.00); frons width 1.71–1.97 (1.82); frons height 0.59–0.71 (0.65); prothoracic femur length 5.74–6.70 (6.10); mesothoracic femur length 6.87–7.89 (7.45); mesothoracic tibia length 5.49–6.39 (5.93); mesothoracic tarsus length 4.79–5.51 (5.22); metathoracic femur length 7.05–7.82 (7.48); metathoracic tibia length 7.65–9.08 (8.40); metathoracic tarsus length 6.95–7.91 (7.42); pronotal elongation measure 0.29–0.31 (0.30); pronotal shape measure 0.38–0.41 (0.39); head shape measure 0.38–0.41 (0.40); frons shape measure 0.34–0.39 (0.36); anteroventral femoral spine count 14–16 (15); anteroventral tibial spine count 10–11 (10); posteroventral tibial spine count 7.

*Head* (Fig. 43A): Transverse, the juxta-ocular protuberances small, but pronounced, the apex just lateral to the midline; the vertex is straight, but sometimes dips just prior to the parietal sutures, even with the dorsal margin of the eyes. Frontal suture with a slight medial carina forming a continuous arc, the entire carina depressed into the head. Ocelli small, the central ocellus the same size as the lateral, all are protruding on small cuticular mounds; the lateral ocelli oriented outward. The carina on the frons not very pronounced, the medial region just ventral to the carina sloped. Clypeus transverse, the upper margin convex, the lower margin concave; the central, transverse carina pronounced and straight. Antennae mostly pale, fading to a darker brown distally. Black band extending straight over the medial carina of the frontal suture, pale medially; two black bands lateral to the lateral ocelli; two black marks just dorsal to frontal suture. Black band extending across the lower region of the frons, but separate from the ventral margin; brown markings in the dorsolateral corners for the clypeus that extend medially along the central carina; the mandibles and labrum with pale and brown markings; the vertex and juxta-ocular protuberances mostly pale with fine, disperse black speckling; the area immediately adjacent to ocelli black. Palpi are pale.

*Pronotum* (Fig. 48C): A little less than three times long as wide with a moderately defined supra-coxal bulge; some tubercles in the posterior third, but otherwise smooth. Prozone square with slightly convex margins that gradually taper to an evenly rounded anterior margin; margins smooth or with very few blunt tubercles. Metazone with concave lateral margins, the nadir of the margins in the posterior half almost parallel for a short distance before widening to the posterior margin; margins with small tubercles; posterior margin with a medial emargination; the dorsal surface of the posterior third of the metazone slightly depressed; tubercles present in the posterolateral corner. Mostly dark with pale and black marking across the surface, faint swirls present on the metazone just posterior to the supra-coxal sulcus.

*Prothoracic Legs*: Femur robust with a slightly concave dorsal margin; strongly defined pale to dark banding on posterior (external) surface; anterior (internal) surface without a black band, but three thin dashes positioned medially, one in the proximal third, one in the middle and one next to the femoral brush; the ventral surface pale. Posterior surface of femur with few tubercles. A shallow femoral pit to accommodate terminal posteroventral tibial spine positioned medial to and slightly distal to the first most proximal posteroventral spine, in line with the most distal discoidal spine; pit is pigmented pale or brown. Posterior prothoracic femoral genicular spine much smaller than posteroventral spines, originating distal to the beginning of the genicular lobe. Prothoracic tibial posteroventral spines with the first (proximal) smallest and the fourth through sixth of similar length, the second and third longer. Prothoracic coxae smooth, the anterior surface with a very small, black mark medially in the proximal half as well as a small, circular black spot medially towards the distal terminus.

*Meso- and Metathoracic Legs*: Femora with ventral (posterior) carina; dorsal (anterior) carina present. Mesotarsi with first segment as long or slightly longer than the remaining segments combined.

*Wings*: Forewings mottled with brown, pale and greenish coloration; the costal region without defined banding proximally, mostly brown and green mottling; the distal half of the costal region with regular banding; vein coloration mostly corresponding with surrounding colors; two pale spots are positioned in the proximal quarter of the discoidal region just posterior to the first radial vein; a larger pale area is positioned centrally. Forewings often, but not always asymmetrically colored; one being mottled as described the other is slightly darkened, the mottled pattern still visible; extending just beyond the abdomen. Hindwings with opaque discoidal region, colored rust proximally and along the anterior margin, otherwise black; the anal region smoky black and translucent; the terminus of the discoidal region projecting beyond the distal margin of anal region, the wing appearing elongate.

*Abdomen*: Slightly widened in the middle, the fourth tergite the widest region before a gradual posterior narrowing; a smooth, brown and black colored dorsal surface. Tergites without posterolateral tergal projections. Supra-anal plate square with rounded posterolateral corners and a blunt terminus. Subgenital plate irregularly rounded and without styli.

*Genital Complex* (Fig. 52A.1–A.5): The main body of ventral left sclerite (L4A) with rounded terminus, but with a distal process (pda) positioned just lateral to the middle that is short and tapering to a sharp point, projecting at an angle laterally, appearing like a small, well-sclerotized tooth (tooth absent in one examined specimen); sometimes a depression on the opposite lateral half from the pda is present. The apofisis falloid (afa) of the main body of dorsal left sclerite (L4B) short, barely present, quickly narrowing to a tiny, sharp point; the apical process (paa) elongate and thin, the terminus evenly rounded. The right dorsal phallomere (fda) of the first sclerite of right phallomere (R1) tapers to a rounded, membranous terminus, the end often folded; the ventral plate (pia) long, broad proximally with strongly defined grooves; the ventral process (pva) tooth-like and curved at the proximal base, the distal tip narrowing with a rapid constriction towards the end.

**Female.** (Fig. 17B) N=11: Body length 22.56–31.05 (26.47); forewing length 13.35–17.48 (15.29); hindwing length 12.61; pronotum length 6.55–7.78 (7.30); prozone length 1.91–2.37 (2.22); pronotum width 2.63–3.09 (2.91); pronotum narrow width 2.04–2.25 (2.14); head width 5.37–6.41 (6.08); head vertex to clypeus 2.27–2.77 (2.59); frons width 2.12–2.52 (2.36); frons height 0.76–0.94 (0.86); prothoracic femur length 6.34–7.65 (7.15); mesothoracic femur length 7.20–8.73 (8.14); mesothoracic tibia length 5.78–7.42 (6.75); mesothoracic tarsus length 4.95–6.42 (5.76); metathoracic femur length 7.16–8.80 (8.11); metathoracic tibia length 8.13–10.95 (9.68); metathoracic tarsus length 7.30–8.75 (8.02); pronotal elongation measure 0.29–0.32 (0.30); pronotal shape measure 0.38–0.41 (0.40); head shape measure 0.40–0.45 (0.43); frons shape measure 0.34–0.39 (0.37); anteroventral femoral spine count 14–16 (16); anteroventral tibial spine count 10; posteroventral tibial spine count 7.

*Head* (Fig. 43B): About as long as broad, the juxta-ocular protuberances large, the apex just lateral to the midline; the vertex is straight, but with two bulges just medial to the parietal sutures, slightly higher than dorsal margin of the eyes. Ocelli raised slightly on a continuous carina connecting all three. The carina on the frons pronounced, the medial region just ventral to the carina sloped. Clypeus transverse, the upper margin convex, the lower margin concave; the central, transverse carina pronounced and straight, the ventral half depressed. The vertex and juxta-ocular protuberances mostly dark brown with fine pale speckling.

*Pronotum* (Fig. 48D): Tubercles in the posterior half, but otherwise smooth; numerous tubercles present in the posterolateral corner of metazone. Two lateral bulges in the dorsal surface around the midline of the metazone.

*Prothoracic Legs*: Prothoracic tibial posteroventral spines with the first (proximal) smallest and the third through sixth of similar length, the second longer.

*Meso- and Metathoracic Legs*: As described for males.

*Wings*: The costal region of forewing without defined banding, mostly brown and green mottling. Forewings are darker brown overall than in males; far shorter than the terminus of the abdomen, often terminating around the sixth segment. Hindwings with the terminus of the discoidal region projecting slightly beyond the distal margin of anal region.

*Abdomen*: Slightly widened, the fifth tergite the widest region before a gradual posterior narrowing. Seventh tergite with small posterolateral projections. Supra-anal plate almost square, a broad, blunt terminus with a slight medial emargination.

###### Etymology.

Named for the island of Trinidad, where this species inhabits and appears to be endemic.

##### Liturgusa zoae sp. n.

http://zoobank.org/46A124DB-35CD-47FE-AB4B-708A18782B6F

http://species-id.net/wiki/Liturgusa_zoae

###### Type.

Holotype Male, pinned. National Museum of Natural History, Smithsonian Institution, Washington, DC, USA.

###### Type locality.

Guatemala, Alta V. Paz, Schwarz & Barber Coll, 2.4 Cacao, Trece Aguas. (Lat. 15.592321, Long. -90.146392).

###### Material examined.

*Liturgusa zoae* sp. n.

**Table:** 

Sex	Type	Country	Label	Latitude Longitude	Code
Male	Holotype	Guatemala	Alta V. Paz, Schwarz & Barber Coll, 2.4 Cacao, Trece Aguas.	15.592321, -90.146392	USNM 062; USNM ENT 00873990
Female	Allotype	Guatemala	Alta V. Paz, Schwarz & Barber Coll, 2.4 Cacao, Trece Aguas.	15.592321, -90.146392	USNM 045; USNM ENT 00873991
Male	Paratype	Honduras	192, Ac. 29596		AMNH 002
Female	Paratype	Panama	Puerto Armuelles	8.274772, -82.865134	AMNH 028
Nymph	nontype	Honduras	Atlantida, Massif Pico Bonito, env. El Pino 200 m, VII/1995 T. Porion A. Grange	15.716539, -86.826280	MNHN 200
Female	Paratype	Belize	Toledo Dist., Blue Creek Village, 22 June 1981, W.E. Steiner	16.195801, -89.043072	USNM 002; USNM ENT 00873017

###### Diagnosis.

A medium sized species, is distinct from all other *Liturgusa*. Could be considered most similar to *Liturgusa guyanensis* based on the pronounced pronotal shape modifications compared to all other *Liturgusa*, but *Liturgusa zoae* has a longer pronotum. The prothoracic femora lacks tubercles on the posterior (external) surface, unique to the species. The hindwings are dark black and highly opaque, appearing rounded.

###### Description.

**Figure 18.**
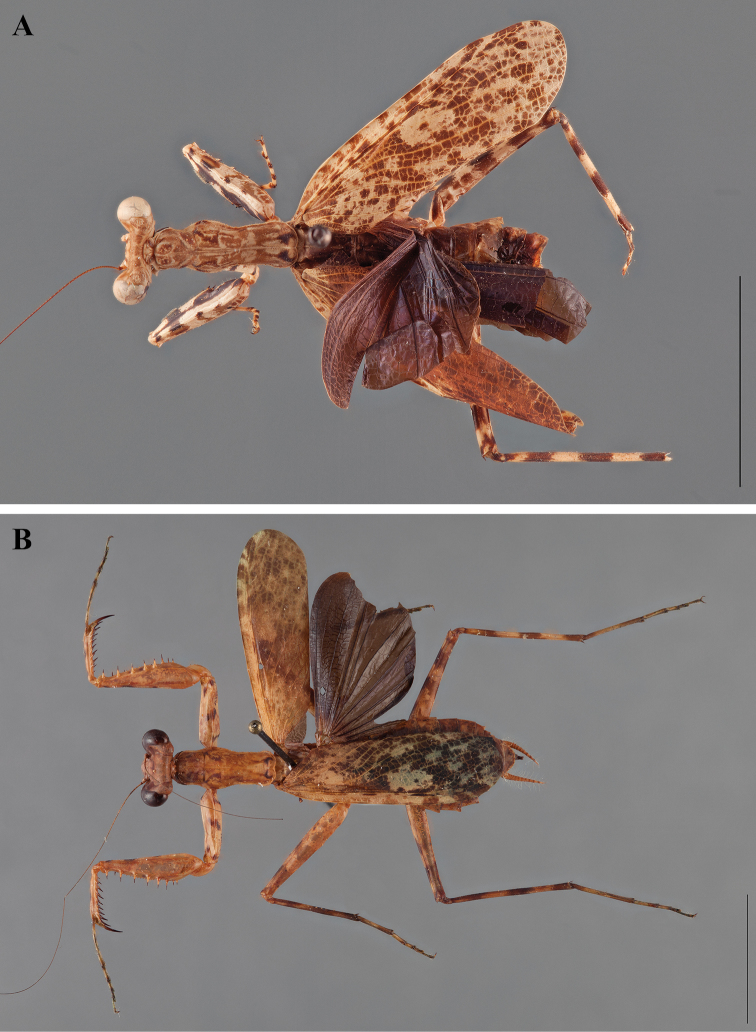
*Liturgusa zoae* sp. n., dorsal habitus: **A** holotype male from Guatemala (USNM 062) **B** allotype female from Guatemala (USNM 045).

**Male.** (Fig. 18A) N=3: Body length 23.28–25.87 (24.69); forewing length 15.09–17.00 (16.17); hindwing length 13.89; pronotum length 6.61–7.23 (6.85); prozone length 1.89–2.01 (1.96); pronotum width 2.47–3.00 (2.75); pronotum narrow width 1.89–1.98 (1.94); head width 4.95–5.52 (5.29); head vertex to clypeus 1.98–2.29 (2.18); frons width 1.66–2.00 (1.85); frons height 0.66–0.80 (0.75); prothoracic femur length 6.77–7.35 (7.01); mesothoracic femur length 8.02–9.37 (8.67); mesothoracic tibia length 6.37–7.08 (6.65); mesothoracic tarsus length 5.21–6.44 (5.82); metathoracic femur length 8.62–9.26 (8.94); metathoracic tibia length 8.84–9.81 (9.32); metathoracic tarsus length 9.47; pronotal elongation measure 0.28–0.29 (0.29); pronotal shape measure 0.37–0.41 (0.40); head shape measure 0.40–0.42 (0.41); frons shape measure 0.40–0.41 (0.40); anteroventral femoral spine count 14–15 (15); anteroventral tibial spine count 9; posteroventral tibial spine count 7.

*Head* (Fig. 43C): Transverse, the juxta-ocular protuberances prominent, the apex in the lateral half; the vertex is concave, dipping just prior to the parietal sutures, even with the dorsal margin of the eyes. Frontal suture with a medial carina forming a continuous arc, the region just ventral and dorsal to the carina depressed, the carina within a trough. Ocelli small, all the same size, protruding and laterally angled on a pronounced, curved carina that connects all three. The carina on the frons pronounced, the medial region just ventral to the carina depressed. Clypeus transverse, the upper margin convex, the lower margin slightly convex, the lateral margins adjoining lower margins with rounded corners; the central, transverse carina pronounced and slightly curved, two small grooves symmetrically located just dorsal to the carina. Antennae scape pale, pedicel dark brown, the flagellum fading to dark brown just slightly distal to the base. Vertex and juxta-ocular protuberances mottled pale and brown; cuticle just adjacent to ocelli dark brown or black; pale centrally to the three ocelli. Frons, clypeus, labrum, mandibles and palpi pale.

*Pronotum* (Fig. 48E): A little less than three times long as wide with a defined supra-coxal bulge; dorsal surface without tubercles; the lateral margins expanded and slightly lamellar. Prozone slightly broader than long with pronounced convex lateral margins; the anterior margin round; the dorsal surface spherical or bulbous, raised higher than seen in other *Liturgusa*; lateral margins smooth or with very few blunt tubercles. Metazone with concave lateral margins, tapering rapidly posterior of the supra-coxal sulcus, the medial region bulging slightly outward before widening to a rounded posterior margin; posterior margin with a small emargination; lateral margins with disperse tubercles, mostly located in the anterior third; the dorsal surface of the posterior third of the metazone very depressed; symmetrical posterior bulges pronounced. Mostly pale with black markings, two prominent black marks near the posterior margin of the metazone and laterally just posterior of the supra-coxal sulcus.

*Prothoracic Legs*: Femur robust with a slightly concave dorsal margin; strongly defined pale to dark banding on posterior (external) surface; anterior (internal) surface with a black band running medially from the base to terminus that may be thinned or interrupted medially, the band thicker near the femoral brush and connected to the dorsal margin; the ventral surface pale. Posterior surface of femur without tubercles. A femoral pit to accommodate terminal posteroventral tibial spine positioned medial to and in line with the first most proximal posteroventral spine, proximal to the most distal discoidal spine; pit is pigmented black. Posterior prothoracic femoral genicular spine smaller than posteroventral spines, originating distal to the beginning of the genicular lobe. Ventral surface with raised, blunt carina just medial to the posteroventral spines beginning at the femoral pit and fading to flat after the third most distal posteroventral spine. Prothoracic tibial posteroventral spines with the first (proximal) smallest, the fourth and fifth slightly longer and the second, third and sixth of similar length. Prothoracic coxae smooth, the anterior surface with a large black band medially in the proximal half as well as a small black spot medially towards the distal terminus.

*Meso- and Metathoracic Legs*: Femora with ventral (posterior) carina; dorsal (anterior) carina present. Mesotarsi with first segment shorter or at most as long as the remaining segments combined.

*Wings*: Forewings mottled with brown, whitish pale, and black coloration; the costal region mottled matching the discoidal region, some banding pattern present medially; the costal region widened; vein coloration across discoidal region pale or light brown; a large pale area is positioned centrally; most of the surface dark brown or black with whitish mottling across the surface, the distal tip more whitish. Forewings asymmetrically colored; one being mottled as described the other is darkened significantly with a rust tone, the mottled pattern still visible; extending just beyond the abdomen. Hindwings opaque black, the veins black; the terminus of the discoidal region projecting just beyond the distal margin of anal region, the wing appearing rounded.

*Abdomen*: Slightly widened in the middle, the fourth or fifth tergite the widest before a gradual posterior narrowing; a smooth, brown and black colored dorsal surface. Tergites with tiny triangular posterolateral tergal projections. Supra-anal plate transverse, an evenly rounded terminus. Subgenital plate irregularly rounded and without styli.

*Genital Complex* (Fig. 52B.1): The main body of ventral left sclerite (L4A) elongate, an evenly rounded terminus with the margin rolled slightly along the terminus; the left side with an elongate depression on the surface; lacking a distal process (pda). The apofisis falloid (afa) of the main body of dorsal left sclerite (L4B) forming a very large triangular projection that evenly tapers to a point; the apical process (paa) elongate and thin, the terminus rounded. The right dorsal phallomere (fda) of the first sclerite of right phallomere (R1) tapers rapidly to a narrowed and rounded, membranous terminus; the ventral plate (pia) long, curved with a medial tooth projecting towards the pva, smooth surface; the ventral process (pva) long and smooth, but with a sharply bent terminus, creating a notch.

**Female.** (Fig. 18B) N=2: Body length 27.92; forewing length 17.64; hindwing length 13.94; pronotum length 7.65–8.30 (7.97); prozone length 2.22–2.59 (2.40); pronotum width 3.23–3.26 (3.25); pronotum narrow width 2.16–2.44 (2.30); head width 6.00–6.22 (6.11); head vertex to clypeus 2.44–2.65 (2.54); frons width 2.23–2.31 (2.27); frons height 0.90–0.97 (0.94); prothoracic femur length 7.98–8.17 (8.07); mesothoracic femur length 8.83–9.54 (9.18); mesothoracic tibia length 6.94–7.09 (7.02); mesothoracic tarsus length 6.38–6.68 (6.53); metathoracic femur length 9.15; metathoracic tibia length 9.93; metathoracic tarsus length 9.93; pronotal elongation measure 0.29–0.31 (0.30); pronotal shape measure 0.39–0.43 (0.41); head shape measure 0.41–0.43 (0.42); frons shape measure 0.40–0.42 (0.41); anteroventral femoral spine count 14–15 (14); anteroventral tibial spine count 9–10 (9); posteroventral tibial spine count 7.

*Head* (Fig. 43D): About as long as broad, the juxta-ocular protuberances prominent, the apex in the middle; the vertex is slightly concave, evenly sloping to a slightly depressed center, above the dorsal margin of the eyes. Region just ventral to the frontal suture slightly depressed. Ocelli very small, all the same size, the lateral ocelli widely positioned, almost in line with the middle of the antennal insertion. The carina on the frons present, but not strongly pronounced. Antennal scape pale, pedicel with black markings, the flagellum brown proximally, fading darker and darker distally. Cuticle just adjacent to ocelli pale or light brown.

*Pronotum* (Fig. 48F): Less than three times long as wide with a defined supra-coxal bulge. Posterior margin of metazone with flat posterolateral corners and with a small medial emargination; lateral margins with numerous, pronounced tubercles.

*Prothoracic Legs*: Prothoracic tibial posteroventral spines with the first (proximal), third through fifth short, the second and sixth longer and similar length..

*Meso- and Metathoracic Legs*: Mesotarsi with first segment shorter than the remaining segments combined.

*Wings*: Forewings with the costal region mottled matching the discoidal region, some banding pattern distally; the costal region widened and extending distally; vein coloration mostly matches surrounding coloration; two pale spots are positioned in the proximal quarter of the discoidal region just posterior to the first radial vein; a large pale area is positioned centrally; black with whitish pale spots in the distal half. Forewings asymmetrically colored; one being darker and less contrasting; extending short of the terminus of the abdomen.

*Abdomen*: Slightly widened, the fourth or fifth tergite the widest region before a gradual posterior narrowing. Tergites with posterolateral projections of varying size, but shaped like expanded triangles and not posteriorly oriented teeth. Supra-anal plate transverse, an evenly rounded terminus.

###### Etymology.

A noun in the genitive case, *Liturgusa zoae* is named for my daughter Zoey Kay Svenson.

#### Cursor Group A

##### Liturgusa cursor

Rehn, 1950

http://species-id.net/wiki/Liturgusa_cursor

Liturgusa annulipes : Rehn 1935: 199, pl. 8, fig. 4.
Liturgousa cursor : Rehn 1950: 369–376, Figs 6–11; Hughes-Schrader 1950: 11–14, 27, 38, 45, Table 1, Fig. 11; Hughes-Schrader 1951: 178, 183–184, 186–187, Tables 1–2, Fig. 3; Hughes-Schrader 1953: 544–554; Henderson 1965: 215; Otte 1978: 76;
Liturgusa cursor : Terra 1995: 54; Jantsch 1999: 48; Ehrmann 2002: 207; Otte and Spearman 2005: 133; Agudelo et al. 2007: 116.

###### Type.

Holotype Male. Academy of Natural Sciences of Drexel University, Philadelphia, PA, USA, Type no. 5761.

###### Type locality.

Panama, Barro Colorado Island, Gatun Lake, Canal Zone, December 31, 1948. (Dr. Franz Schrader, no. 630.) (Lat. 9.164966, Long. -79.837098).

###### Material examined.

*Liturgusa cursor* Rehn, 1950.

**Table:** 

Sex	Type	Country	Label	Latitude Longitude	Code
Male	Holotype	Panama	Barro Colorado Island, Gatun Lake, Canal Zone, December 31, 1948. (Dr. Franz Schrader, no. 630.)	9.164966, -79.837098	ANSP
Male	Paratype	Panama	Barro Colorado C.Z., 31-XII-‘48, F. Schrader, 614	9.164966, -79.837098	ANSP 058
Male	Paratype	Panama	Barro Colorado C.Z., 31-XII-‘48, F. Schrader, 639	9.164966, -79.837098	ANSP 059
Male	Paratype	Panama	Barro Colorado C.Z., 31-XII-‘48, F. Schrader, 624	9.164966, -79.837098	ANSP 060
Male	Paratype	Panama	Barro Colorado C.Z., 31-XII-‘48, F. Schrader, 631	9.164966, -79.837098	ANSP 061
Male	Paratype	Panama	Barro Colorado C.Z., 31-XII-‘48, F. Schrader, 638	9.164966, -79.837098	ANSP 062
Male	Paratype	Panama	Barro Colorado C.Z., 31-XII-‘48, F. Schrader, 653	9.164966, -79.837098	ANSP 063
Female	Paratype	Costa Rica	Lower Rio Reventazon, Castilla Farm, VII. 23. 1936, C.W. Dodge	10.078445, -83.573754	ANSP 070
Male	Paratype	Costa Rica	Turrialba, 8 VI, 48, F. Schrader, 515	9.989971, -83.763587	ANSP 071
Male	Paratype	Costa Rica			ANSP 073
Female	Paratype	Costa Rica	Ujarass de Terraba, C.R. IX.10.07, M.A.C. Jr.	9.831438, -83.832067	ANSP 074
Female	nontype	Panama	Bugaba, 800-1,500 ft., Champion., Godman-Salvin Coll. 1894-125.	8.490081, -82.620285	BMNH 008
Female	nontype	Panama	Bocas del Toro Prov., Palo Seco NP, at ANAM station, 08°47.602'N, 082°11.330'W, 523m, 4.VI.2009, N. Lord, K. Miller, E. Nearns Colrs., General collecting	8.793367, -82.188833	GSMC000285
Female	nontype	Costa Rica	Alajuela, Rio San Juan across from Bartola, 10.974153°N, 84.343499°W, 4 November, 2010, Coll: Gavin J. Svenson	10.974153, -84.343499	GSMC002952
25 Males, 18 Females	nontype	Nicaragua	Rio San Juan, Refugio Bartola sur le Rio San Juan, 10.974309°N, 84.338318°W, 52 m, 1-5 November, 2010, Coll: Gavin J. Svenson	10.974309, -84.338318	GSMC003039, GSMC003043, GSMC003049, GSMC003055, GSMC003057, GSMC003060, GSMC003433-69
9 Males, 8 Females	nontype	Nicaragua	Rio San Juan Dpto., Refugio Bartola; KBM 18051201	10.972540, -84.338990	GSMC003598, GSMC003602, GSMC003604, GSMC003607-08, GSMC003612-13, GSMC003621-22, GSMC003624-25, GSMC003627-28, GSMC003635-36, GSMC003646-47
Female	nontype	Nicaragua	Rio San Juan, Cerro El Gigante, IX-98, col. I. Coronado	11.316590, -84.612565	MNHN 080
Female	nontype	Panama	Barro Colo Is CZ, VI-18-37, S.W. Frost colr.	9.164966, -79.837098	USNM 049; USNM ENT 00873018
Female	nontype	Costa Rica	Farm Hamburg, am Reventazon, 2.II.1932, F. Nevermann leg., Einz. Nr. 74. 1932	10.250000, -83.450000	ZMUH 003
Male	nontype	Costa Rica	Farm Hamburg, am Reventazon, 2.II.1932, F. Nevermann leg., Einz. Nr. 74. 1932	10.250000, -83.450000	ZMUH 004
Female	nontype	Costa Rica	Puntarenas Prov. Osa pen., nr. Matapalo; MV light coll. 2010 ent. Class colr. KBM 30051003	8.399580, -83.313750	GSMC003085
Female	nontype	Panama	Barro C‘do I., I-20-26: 59, C.Z.	9.164966, -79.837098	FMNH 008
Male	nontype	Panama	Barro Colorado I., Canal Zone, Panama, I-13-59, H.S. Dybas leg., at light	9.164966, -79.837098	FMNH 009

###### Taxonomic history.

Described in 1950 by James Rehn, the species was originally identified as *Liturgusa annulipes* by Rehn in his 1935 work on Orthoptera of Costa Rica. Rehn later recognized that the species was unique, recording specimens mainly from Barro Colorado in Panama, but including one specimen from Costa Rica. We found the species in southern Nicaragua as well, which is a new record for that country. Interestingly, the species was included in a number of studies focused on chromosomes headed by Sally Hughes-Schrader in the 1940’s and into the 60’s. Other than being included in species records for regional studies and taxonomic lists, the species has received no taxonomic attention since its original description.

###### Natural history.

Encountered commonly in lowland tropical forest in southern Nicaragua. Individuals were present on trees in agricultural fields, secondary forest as well as primary forest regions, but with lower densities. Usually found positioned head down on the tree trunk and varying elevations. Individuals ran rapidly up and/or to the opposite side of the trunk when approached. All trees were smooth bark. Found living sympatrically with *Liturgusa fossetti*, *Liturgusa maya*, and *Corticomantis atricoxata*.

###### Diagnosis.

A small, slender species like *Liturgusa dominica* and *Liturgusa milleri*, but restricted in distribution to Central America. The forewings of males are colored asymmetrically while *Liturgusa dominica* and *Liturgusa milleri* both have symmetrically colored forewings; the costal region in males and females with a contrasting green color in newly preserved specimens, but usually faded to pale in older specimens. The genitalia of males are unique among *Liturgusa* with an elongate and narrowed distal terminus of the right dorsal phallomere (fda) of the first sclerite of right phallomere (R1).

A thorough description of the species was provided by Rehn (1950) and can be referred to in addition to the standardized description provided herein.

###### Redescription.

**Figure 19.**
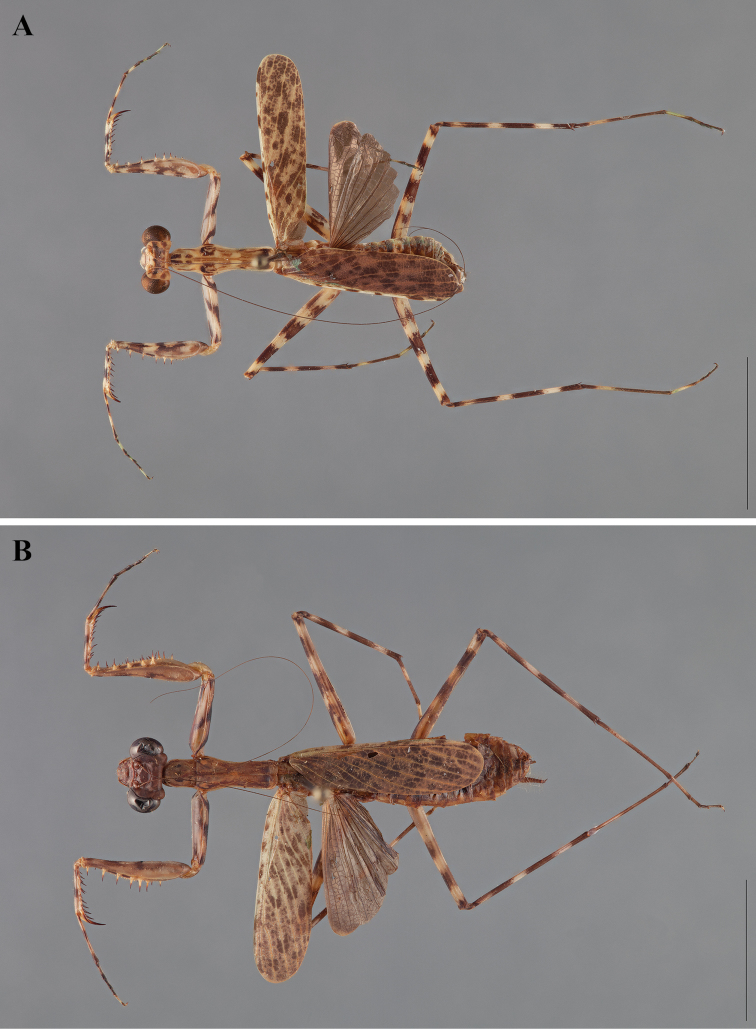
*Liturgusa cursor* Rehn, 1950, dorsal habitus: **A** paratype male from Barro Colorado Island, Panama (ANSP 061) **B** paratype female from Barro Colorado Island, Panama (ANSP 070).

**Male.** (Fig. 19A) N=11: Body length 19.23–22.61 (20.94); forewing length 12.00–13.18 (12.57); hindwing length 9.34–9.84 (9.52); pronotum length 6.27–7.01 (6.66); prozone length 1.82–2.07 (1.99); pronotum width 1.99–2.17 (2.09); pronotum narrow width 1.32–1.54 (1.39); head width 4.37–4.74 (4.51); head vertex to clypeus 1.69–1.85 (1.76); frons width 1.39–1.56 (1.47); frons height 0.53–0.74 (0.60); prothoracic femur length 6.03–6.70 (6.33); mesothoracic femur length 8.41–9.37 (8.71); mesothoracic tibia length 6.42–7.28 (6.67); mesothoracic tarsus length 5.81–6.78 (6.31); metathoracic femur length 8.18–9.23 (8.56); metathoracic tibia length 8.68–9.83 (8.93); metathoracic tarsus length 9.72–11.46 (10.23); pronotal elongation measure 0.29–0.30 (0.30); pronotal shape measure 0.30–0.33 (0.32); head shape measure 0.38–0.40 (0.39); frons shape measure 0.38–0.47 (0.41); anteroventral femoral spine count 14–15 (15); anteroventral tibial spine count 9; posteroventral tibial spine count 7.

*Head* (Fig. 43E): Slightly transverse, the juxta-ocular protuberances small, the apex in the lateral half; the vertex between the parietal sutures with two convex regions, the medial line being slightly depressed; vertex slightly lower than the dorsal margin of the eyes. Frontal suture with a medial carina forming a continuous arc. Ocelli small and positioned on the edge of a blunt, curved carina that connects all three ocelli, continuing slightly beyond the lateral ocelli. Frons transverse, the lower portion narrowed under the antennal insertions. Lateral ocelli oriented outward. Upper margin of clypeus convex, lower margin straight; a medial transverse ridge moderately pronounced, rounded. Antennae pale basally, fading almost immediately to black. Two black spots lateral to the frontal suture at the base of each parietal suture; lower region of frons with a broad black band; the clypeus mostly pale with two brown spots in the upper corners; the mandibles pale with lateral darkening; the labrum with a pale upper region and a brown to black lower region; the vertex and juxta-ocular protuberances with brown and black markings; thin, black margins surround the ocelli. Palpi are pale.

*Pronotum* (Fig. 48G): Elongate with a defined supra-coxal bulge; dorsal surface smooth. Prozone elongate with gradually widening margins before tapering anteriorly to a rounded anterior margin; the lateral margins smooth. Metazone with concave lateral margins, narrowing quickly posterior to the supra-coxal bulge to the midpoint, then widening gradually until reaching the rounded posterior terminus; margins with few small tubercles; posterior margin barely medially emarginate; the dorsal surface of the posterior third of the metazone barely depressed. Pale coloration dominant with strong black marks near the anterior and posterior margins and symmetrical black swirls on the supra-coxal bulge.

*Prothoracic Legs*: Femur elongate with a slightly concave dorsal margin; strongly defined pale to dark banding on posterior (external) surface; anterior (internal) surface with a black band running medially from the base to terminus, often interrupted medially; a broad black mark at the base and just distal to the tibial spur groove; the ventral surface pale. Posterior surface of femur with few tubercles. A well developed femoral pit to accommodate terminal posteroventral tibial spine positioned medial to the most proximal posteroventral spine; pit is pale. Posterior prothoracic femoral genicular spine slightly smaller than posteroventral spines, originating distal to the beginning of the genicular lobe. Prothoracic tibial posteroventral spines with the first (proximal) smallest and the fourth through sixth of similar length, the second and third are longer (the second being the longest). Prothoracic coxae smooth; the anterior surface with a small, black band medially in the proximal half as well as a black spot medially towards the distal terminus.

*Meso- and Metathoracic Legs*: Femora with a faint ventral (posterior) carina; dorsal (anterior) carina faint. Mesotarsi with first segment as long or longer than remaining segments combined.

*Wings*: Forewings mottled with pale, brown, and green coloration; the costal region with pale/green and dark near regular banding; the discoidal region evenly mottled with large brown and pale color markings. Forewings often colored asymmetrically, one being mottled as described above while the other is rust colored with the mottled pattern still visible; extending to around the terminus of the abdomen. Hindwings smoky black, but translucent, the discoidal region darker and more opaque; the discoidal region narrowed; the costal region dark brown proximally; the terminus of the discoidal region projecting slightly beyond the distal margin of anal region.

*Abdomen*: Elongate, tubular with slight widening before posterior narrowing; a smooth brown and black colored dorsal surface. Supra-anal plate highly transverse, tapering quickly to a broad, blunt and wide terminus. Subgenital plate irregularly rounded and without styli.

*Genital Complex* (Fig. 52C.1–C.3): The main body of ventral left sclerite (L4A) with a narrowed, but rounded terminus, lacking a distal process (pda); an elliptical depression on the lateral half is present. The apofisis falloid (afa) of the main body of dorsal left sclerite (L4B) elongate and finger-like, margins narrowing before reaching an expanded and bulbous terminal end, either straight or curved; the apical process (paa) short, curved and with a rounded end. The right dorsal phallomere (fda) of the first sclerite of right phallomere (R1) tapers dramatically to a narrowed and membranous terminus, almost pointed, but the tip is blunt; the ventral plate (pia) short and tooth-like; the ventral process (pva) c-shaped and smooth.

###### Redescription.

**Female.** (Figs 1A, 19B) N=11: Body length 24.15–29.52 (26.39); forewing length 13.83–16.18 (14.73); hindwing length 10.73–12.71 (11.46); pronotum length 7.37–8.32 (7.88); prozone length 2.20–2.49 (2.35); pronotum width 2.38–2.78 (2.58); pronotum narrow width 1.55–2.02 (1.74); head width 5.23–5.48 (5.35); head vertex to clypeus 2.19–2.38 (2.28); frons width 1.85–1.97 (1.90); frons height 0.71–0.86 (0.79); prothoracic femur length 7.19–7.97 (7.64); mesothoracic femur length 9.28–10.45 (9.84); mesothoracic tibia length 7.25–8.09 (7.73); mesothoracic tarsus length 6.73–7.43 (7.10); metathoracic femur length 9.25–10.53 (9.80); metathoracic tibia length 9.88–10.99 (10.33); metathoracic tarsus length 10.70–11.61 (11.11); pronotal elongation measure 0.29–0.31 (0.30); pronotal shape measure 0.31–0.35 (0.33); head shape measure 0.41–0.46 (0.43); frons shape measure 0.38–0.44 (0.42); anteroventral femoral spine count 14–15 (15); anteroventral tibial spine count 9–10 (9); posteroventral tibial spine count 7.

*Head* (Fig. 43F): As broad is high, the juxta-ocular protuberances large, the apex in the lateral half; the vertex between the parietal sutures concave; vertex slightly higher than the dorsal margin of the eyes. Frontal suture with a medial carina forming a high and continuous arc. Lateral ocelli oriented anterolaterally. Clypeus slightly transverse, upper margin convex, lower margin straight. Antennae pale basally, fading almost immediately to black. Black markings surround frontal suture, are present on the vertex and juxta-ocular protuberances. Lower region of frons with a broad black band; the mandibles pale with distal darkening; the labrum mostly pale with a brown medial strip.

*Pronotum* (Fig. 48H): Margins of prozone with few small tubercles.

*Prothoracic Legs*: As described for males.

*Meso- and Metathoracic Legs*: As described for males.

*Wings*: Forewings colored symmetrically; shorter than the abdomen, reaching the fifth or sixth tergite. Hindwings with the costal region light brown proximally; this distal margin of the discoidal region pale or light brown.

*Abdomen*: Widening from first segment until the beginning of the distal half (segments 4–5) when the lateral margins narrow to the terminus, the middle being the broadest region. Tergites without posterolateral tergal projections. Supra-anal plate highly transverse, evenly rounded.

##### Liturgusa dominica sp. n.

http://zoobank.org/99CF12A4-0033-420A-AB6F-5E7BA848A1D2

http://species-id.net/wiki/Liturgusa_dominica

###### Type.

Holotype Male, pinned. National Museum of Natural History, Smithsonian Institution, Washington, DC, USA.

###### Type locality.

Dominica, Grand Bay, IV-13-1964, O.S. Flint, Jr. (Lat. 15.239545, Long. -61.320099)

###### Material examined.

*Liturgusa dominica* sp. n.

**Table:** 

Sex	Type	Country	Label	Latitude Longitude	Code
Male	Holotype	Dominica	Grand Bay, IV-13-1964, O.S. Flint, Jr., Bredin-Archbold-Smithsonian Bio.Surv.Dominica	15.239545, -61.320099	USNM 036: USNM ENT 00873995
Female	Allotype	Dominica	Grand Bay, IV-13-1964, O.S. Flint, Jr., Bredin-Archbold-Smithsonian Bio.Surv.Dominica	15.239545, -61.320099	USNM 034: USNM ENT 00873996
Female	Paratype	Dominica	B.W.I., La Haut Estate, 18-I-1979, A.T. Finnamore		Lyman Museum
Nymph	Paratype	Dominica	Grand Bay, IV-13-1964, O.S. Flint, Jr., Bredin-Archbold-Smithsonian Bio.Surv.Dominica	15.239545, -61.320099	USNM 039; USNM ENT 00873019
Female	Paratype	Dominica	Fond Figues, III-16-1964, Dale F. Bray, Bredin-Archbold-Smithsonian Bio.Surv.Dominica		USNM 040; USNM ENT 00873020
Male	Paratype	Dominica	Grand Bay, IV-13-1964, O.S. Flint, Jr., Bredin-Archbold-Smithsonian Bio.Surv.Dominica	15.239545, -61.320099	USNM 041; USNM ENT 00873021
Male	Paratype	Dominica	Grand Bay, IV-13-1964, O.S. Flint, Jr., Bredin-Archbold-Smithsonian Bio.Surv.Dominica	15.239545, -61.320099	USNM 042; USNM ENT 00873022
Male	Paratype	Dominica	Grand Bay, IV-13-1964, O.S. Flint, Jr., Bredin-Archbold-Smithsonian Bio.Surv.Dominica	15.239545, -61.320099	USNM 043; USNM ENT 00873023
Nymph	Paratype	Dominica	Clarke Hall, 22-31-X-1966, A.B. Gurney		USNM 021; USNM ENT 00873024

###### Diagnosis.

This small species can be identified based on its slender size and its geographic restriction to the Caribbean island of Dominica. Much like *Liturgusa milleri*, the forewings also have white or grey spots in proximal quarter just posterior to the radial vein. The forewings are also net-like in appearance with pale veins and black pigmented cells. The anterior surface of the prothoracic coxae has no pigmentation, while *Liturgusa milleri* and *Liturgusa cursor* both have black markings. In addition, the juxta-ocular protuberances are very small and the entire vertex is concave, falling well below the dorsal margin of the eyes in both sexes. The other two species that resemble *Liturgusa dominica* are restricted to French Guiana and Central America (*Liturgusa milleri* and *Liturgusa cursor*, respectively).

###### Description.

**Figure 20.**
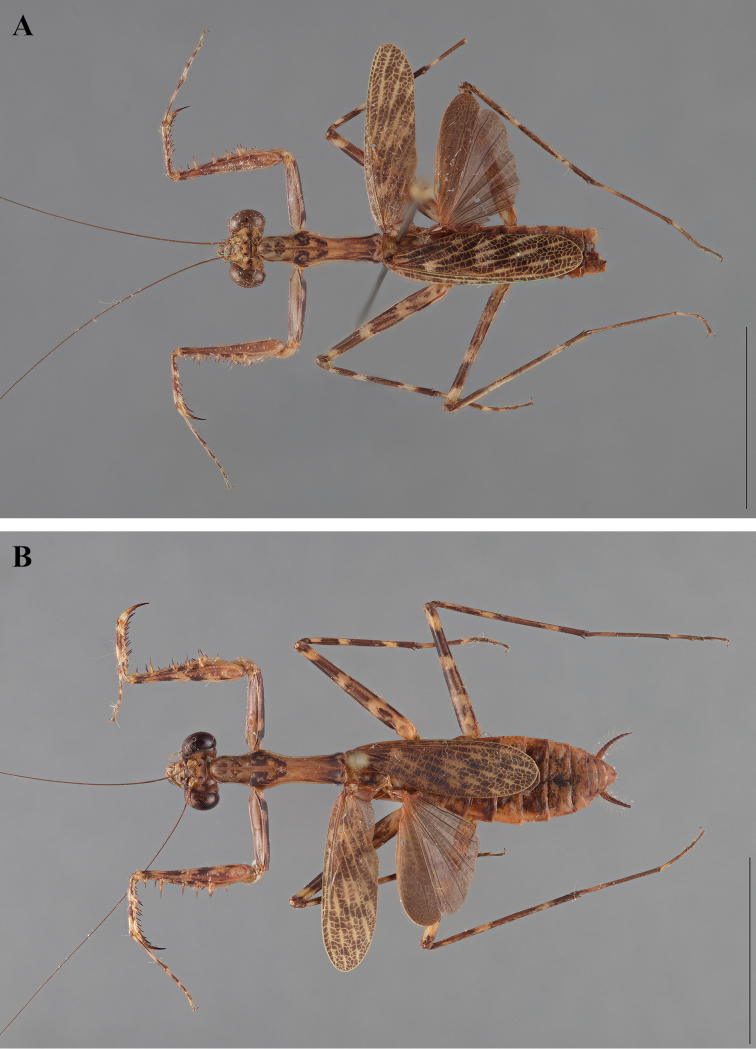
*Liturgusa dominica* sp. n., dorsal habitus: **A** holotype male from Dominica (USNM 036) **B** allotype female from Dominica (USNM 034).

**Male.** (Fig. 20A) N=4: Body length 21.12–21.82 (21.38); forewing length 10.74–11.71 (11.29); hindwing length 8.12; pronotum length 6.91–7.28 (7.11); prozone length 2.02–2.11 (2.06); pronotum width 2.01–2.15 (2.09); pronotum narrow width 1.33–1.45 (1.40); head width 4.36–4.56 (4.47); head vertex to clypeus 1.55–1.67 (1.62); frons width 1.47–1.58 (1.53); frons height 0.56–0.60 (0.57); prothoracic femur length 6.02–6.47 (6.28); mesothoracic femur length 8.42–8.91 (8.66); mesothoracic tibia length 6.21–6.60 (6.44); mesothoracic tarsus length 5.79–6.19 (5.97); metathoracic femur length 8.58–9.05 (8.82); metathoracic tibia length 8.70–9.18 (8.93); metathoracic tarsus length 8.57–9.08 (8.80); pronotal elongation measure 0.29; pronotal shape measure 0.29–0.30 (0.29); head shape measure 0.36–0.38 (0.36); frons shape measure 0.36–0.40 (0.37); anteroventral femoral spine count 14–16 (15); anteroventral tibial spine count 10; posteroventral tibial spine count 7 (one male with 6 on left tibia).

*Head* (Fig. 43G): Transverse, the juxta-ocular protuberances barely present, reduced to a slight bulge, the apex of which is in the lateral half; the vertex is concave overall; the medial region well below the dorsal margin of the eyes. Frontal suture with a medial carina forming a continuous arc, but depressed into the anterior surface of the head, more so ventral to the suture. Ocelli small and protruding on small cuticular mounds. Lateral ocelli oriented outward, a few degrees off perpendicular. Clypeus transverse, the upper margin convex, the lower margin straight. Antennae pale at the base, the flagellum fading to brown near the base. Thin black band extending straight over the medial carina of the frontal suture, the medial portion of the carina pale; lower region of frons with a black band; the clypeus is pale; the mandibles and labrum mostly pale, but with some brown marks; the vertex and juxta-ocular protuberances pale, but with four brown and black marks positioned on each juxta-ocular protuberance and between the parietal sutures; the area immediately adjacent to lateral ocelli black. Palpi are pale.

*Pronotum* (Fig. 48I): Highly elongate with a defined supra-coxal bulge; dorsal surface entirely smooth. Prozone elongate with slightly convex lateral margins that taper anteriorly; the margins smooth. Metazone with strongly concave lateral margins, a slight bulge in the posterior half; margins with small tubercles; posterior margin with a medial emargination; the dorsal surface of the posterior third of the metazone barely depressed. Pale with strong black marks across the surface, swirls present at the supra-coxal bulge.

*Prothoracic Legs*: Femur elongate with a slightly concave dorsal margin; tubular in overall shape with the dorsal margin less defined, the anterior and posterior surfaces almost continuous; strongly defined pale to dark banding on posterior (external) surface; anterior (internal) surface with a black band running medially from the base to terminus; the ventral surface pale. Posterior surface of femur with few tubercles. A well developed femoral pit to accommodate terminal posteroventral tibial spine positioned medial to and exactly between the first two proximal posteroventral spines, but slightly proximal to the most distal discoidal spine; pit is pigmented darkly. Posterior prothoracic femoral genicular spine much smaller than posteroventral spines, originating just distal to the beginning of the genicular lobe. Prothoracic tibial posteroventral spines with the first (proximal) smallest and the fourth through sixth of similar length, the second and third are much longer (about half the length of the terminal spine). Prothoracic coxae smooth, the anterior surface pale.

*Meso- and Metathoracic Legs*: Femora with ventral (posterior) carina; dorsal (anterior) carina faint. Mesotarsi with first segment as long or shorter than remaining segments combined.

*Wings*: Forewings mottled with black, light brown, and greenish coloration; the costal region without strong banding, pale and black proximally and mostly black distally; veins are pale and cells are black across the discoidal and distal portion of the costal region, giving a contrasting net-like appearance; two pale spots are positioned in the proximal quarter of the discoidal region just posterior to the first radial vein. Forewings colored symmetrically; extending just beyond the terminus of the abdomen. Hindwings opaque brown, the discoidal region more pale proximally; the costal region light brown proximally and narrowing to the anterior margin distally; the terminus of the discoidal region barely projecting beyond the distal margin of anal region, the distal margin strongly emarginate between the anal and discoidal region.

*Abdomen*: Elongate, tubular with slight widening before posterior narrowing; a smooth, brown and black colored dorsal surface. Supra-anal plate transverse, tapering gradually to a rounded terminus; the terminus with a slight emargination. Subgenital plate irregularly rounded and without styli.

*Genital Complex* (Fig. 52D.1): The main body of ventral left sclerite (L4A) with rounded terminus, but often with a slight bulge just lateral to the medial line, lacking a distal process (pda); sometimes a depression on the lateral half is present. The apofisis falloid (afa) of the main body of dorsal left sclerite (L4B) elongate and robust, tapering to a strong point and angled off the central axis of the L4B; the apical process (paa) broad, shortened, cylindrical and curved, the terminus a rounded end. The right dorsal phallomere (fda) of the first sclerite of right phallomere (R1) tapers to a rounded, membranous terminus; the ventral plate (pia) long and irregular, with grooves; the ventral process (pva) smooth and tapering to a point distally, one edge straight and the other convex, tooth-like in appearance.

**Female.** (Fig. 20B) N=2: Body length 26.05–27.47 (26.76); forewing length 12.52–12.79 (12.65); hindwing length 9.29; pronotum length 8.67–9.04 (8.85); prozone length 2.50–2.66 (2.58); pronotum width 2.57–2.65 (2.61); pronotum narrow width 1.66–1.72 (1.69); head width 5.22–5.29 (5.26); head vertex to clypeus 2.09; frons width 1.87–2.10 (1.98); frons height 0.63–0.78 (0.71); prothoracic femur length 7.69–7.75 (7.72); mesothoracic femur length 9.92–10.23 (10.08); mesothoracic tibia length 7.69–7.84 (7.77); mesothoracic tarsus length 6.73–6.78 (6.76); metathoracic femur length 10.00; metathoracic tibia length 10.57–10.94 (10.75); metathoracic tarsus length 9.78–9.97 (9.88); pronotal elongation measure 0.29; pronotal shape measure 0.29–0.30 (0.29); head shape measure 0.40; frons shape measure 0.30–0.42 (0.36); anteroventral femoral spine count 14–16 (16); anteroventral tibial spine count 10; posteroventral tibial spine count 7.

*Head* (Fig. 43H): Head is transverse, the juxta-ocular protuberances very small, reduced to a bulge, the apex of which is in the lateral half; the vertex is concave overall, the medial region below the dorsal margin of the eyes. Clypeus transverse, the upper margin convex, the lower margin concave. Antennae pale at the insertion, fading to a brown black at the first antennomere. The clypeus is pale with two dorsolateral dark spots; the mandibles and labrum mostly pale, but with some brown marks; the vertex and juxta-ocular protuberances pale, but with disperse brown markings; the area immediately adjacent to lateral ocelli black. Palpi are pale.

*Pronotum* (Fig. 48J): As described for males.

*Prothoracic Legs*: As described for males.

*Meso- and Metathoracic Legs*: As described for males.

*Wings*: Forewings shorter than the abdomen, often terminating at the fifth tergite. Hindwings with the terminus of the discoidal region barely projecting beyond the distal margin of anal region, the distal margin strongly emarginate between the anal and discoidal region.

*Abdomen*: Widening from first segment until the beginning of the distal half (segments 4–5) when the lateral margins narrow to the terminus, the middle being the broadest region. Tergites without posterolateral tergal projections. Supra-anal plate slightly transverse, rounded.

###### Ootheca

**Figure 21.**
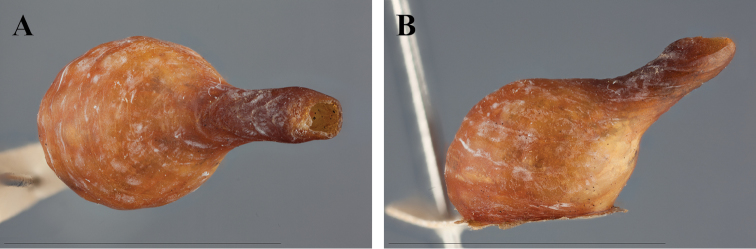
*Liturgusa dominica* sp. n., ootheca: **A** dorsal **B** lateral.

(Fig. 21A–B). Like all other known oothecae of *Liturgusa*, *Liturgusa dominica* produces a broad based case with a narrowing tube extending dorsolaterally away from the substrate. The eggs are contained within the bulbous base that is almost spherical excluding the flattened margin where the case is attached to the bark of a tree or branch. The tube narrows quickly and is short, about half the diameter of the main body. The tube is hollow and the entry is flat, but at an angle to the long axis of the tube, the upper margin extending further than the lower margin forming an elliptical opening that is parallel to the substrate (may reduce water intrusion).

###### Etymology.

A noun in apposition, *Liturgusa dominica* is named for the island of Dominica, where this species inhabits and appears to be endemic.

##### Liturgusa milleri sp. n.

http://zoobank.org/CDF3E3B4-0076-471F-9B3A-158270CE8E29

http://species-id.net/wiki/Liturgusa_milleri

###### Type.

Holotype Male, pinned. Cleveland Museum of Natural History, Cleveland, OH, USA.

###### Type locality.

French Guiana: Kaw Mountain Res., Amazonas Lodge, 8-19 Feb 2005, Coll: K.B. Miller (Lat. 4.549389, Long. -52.213806).

###### Material examined.

*Liturgusa milleri* sp. n.

**Table:** 

Sex	Type	Country	Label	Latitude Longitude	Code
Male	Holotype	French Guiana	Kaw Mountain Res., Amazonas Lodge, 4°32'57.8"N, 52°12'49.7"W, 8-19 Feb 2005, Coll: K.B. Miller	4.549389, -52.213806	GSMC000264
Female	Allotype	French Guiana	Kaw Mountain Res., Amazonas Lodge, 4°32'57.8"N, 52°12'49.7"W, 8-19 Feb 2005, Coll: K.B. Miller	4.549389, -52.213806	GSMC000260
3 Females	Paratypes	French Guiana	Kaw Mountain Res., Amazonas Lodge, 4°32'57.8"N, 52°12'49.7"W, 8-19 Feb 2005, Coll: K.B. Miller	4.549389, -52.213806	GSMC000266, GSMC000314, GSMC003470
Male	Paratype	French Guiana	Kaw Mountain Reserve, vicinity of Patawa, 30.IV/1.V.2011, 4.56888N, 52.21388W, elev. 282 m (at light), J. Rivera leg.	4.568880, -52.213880	GSMC003471
Female	Paratype	French Guiana	St-Laurent du Maroni, Collection Le Moult, Coll. L. Chopard, 1919	5.487038, -54.008462	MNHN 017
Female	Paratype	French Guiana	Arataye Affl Approuagues, Skm NE pied Saut Parare, 1-VI-1988, Chasse de nuit, L. Desutter & P. Grandcolas rec.	4.046724, -52.698087	MNHN 021
Female	Paratype	French Guiana	Crique Venus, IX-1992, P. Peters	5.181326, -52.926674	MNHN 023
Female	Paratype	French Guiana	Sinnamary, 3-6-VII-1977, Guyane, M. Descamps rec.	5.370512, -52.960320	MNHN 025
Male	Paratype	French Guiana	St-Jean du Maroni, R. Benoist, 1914	5.487038, -54.008462	MNHN 035
Female	Paratype	French Guiana	St-Laurent du Maroni, Collection Le Moult, Coll. L. Chopard, 1919, Juliet	5.487038, -54.008462	MNHN 052
Female	Paratype	French Guiana	St-Jean du Maroni, R. Benoist, 1914, Mars	5.487038, -54.008462	MNHN 053
Female	Paratype	French Guiana	Nouveau Chantier, collection Le Moult, Coll. L. Chopard, 1919, Mai		MNHN 054
Male	Paratype	French Guiana	Camopi, F. Geay - 1900	3.167700, -52.339455	MNHN 072
Male	Paratype	French Guiana	Massikiri-Oyapock, 16-Nov-1969, Balachowski-Gruner, Oct.Nov. 1969		MNHN 073
Male	Paratype	French Guiana	S. Boucher, 1984-85		MNHN 076
Female	Paratype	French Guiana	NE, Route de Kaw, Caiman Camp env. 7.XII.2006, Snizek		MSMC 003
Female	Paratype	French Guiana	St-Laurent du Maroni	5.487038, -54.008462	OUMNH 009
Male	Paratype	French Guiana			GSMC003015

###### Diagnosis.

This small species can be identified based on its slender size, its geographic restriction to the Guianas as well as the two symmetrically located white spots in the proximal quarter of their forewings. The other two species that resemble *Liturgusa milleri* are restricted to the island of Dominica and Central America (*Liturgusa dominica* and *Liturgusa cursor*, respectively).

###### Description.

**Figure 22.**
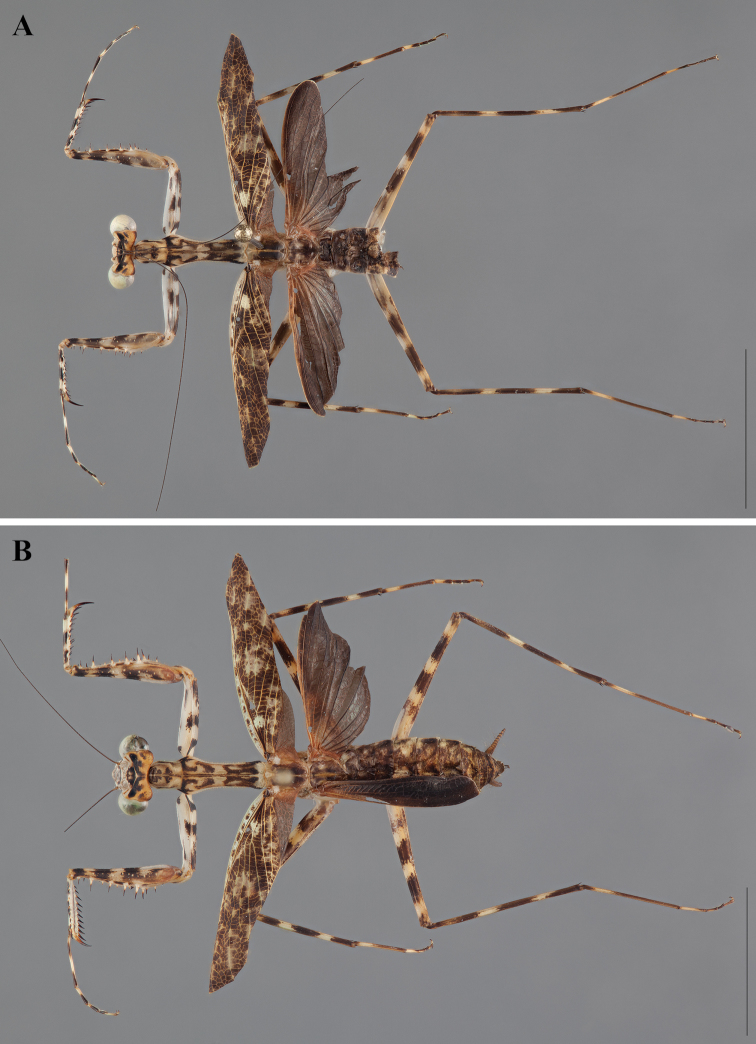
*Liturgusa milleri* sp. n., dorsal habitus: **A** holotype male from Kaw Mountain, French Guiana (CLEV GSMC000264) **B** allotype female from Kaw Mountain, French Guiana (CLEV GSMC000260).

**Male.** (Fig. 22A) N=6: Body length 19.54–19.91 (19.67); forewing length 11.59–12.72 (12.07); hindwing length 9.26–10.19 (9.72); pronotum length 6.32–7.16 (6.69); prozone length 1.84–2.12 (1.95); pronotum width 1.90–2.10 (2.02); pronotum narrow width 1.27–1.39 (1.32); head width 4.42–4.74 (4.53); head vertex to clypeus 1.68–1.79 (1.72); frons width 1.46–1.63 (1.53); frons height 0.57–0.62 (0.59); prothoracic femur length 5.78–6.30 (5.92); mesothoracic femur length 7.96–8.70 (8.26); mesothoracic tibia length 6.11–7.09 (6.54); mesothoracic tarsus length 4.64–6.06 (5.58); metathoracic femur length 7.73–9.03 (8.46); metathoracic tibia length 8.22–9.55 (8.76); metathoracic tarsus length 8.80–9.35 (9.15); pronotal elongation measure 0.27–0.31 (0.29); pronotal shape measure 0.28–0.33 (0.30); head shape measure 0.38; frons shape measure 0.37–0.41 (0.39); anteroventral femoral spine count 13–15 (15); anteroventral tibial spine count 10; posteroventral tibial spine count 7.

*Head* (Fig. 43I): Transverse, the juxta-ocular protuberances small, the apex in the lateral half; the vertex between the parietal sutures is slightly concave, uneven; vertex lower than the dorsal margin of the eyes. Frontal suture with a medial carina forming a continuous arc, but depressed into the anterior surface of the head. Ocelli small and protruding on small cuticular mounds. Lateral ocelli oriented outward, a few degrees off perpendicular. Clypeus highly transverse, the upper margin convex, the lateral and lower margin forming a continuous rounded margin. Antennae pale at the base, the scapes with two dark marks, the flagellum fading to black within a few antennomeres from the base. Broad black band extending straight over the medial carina of the frontal suture, the carina remaining white; two white spots between the lateral ocelli; lower region of frons with a black band; the clypeus with two black spots in the upper lateral corners; the mandibles and labrum pale; the vertex and juxta-ocular protuberances pale, but with four black marks, two positioned on each side of the parietal sutures; the area adjacent to lateral ocelli black. Palpi are pale.

*Pronotum* (Fig. 48K): Highly elongate with a defined supra-coxal bulge; dorsal surface entirely smooth. Prozone elongate with slightly convex lateral margins that taper anteriorly; the margins smooth. Metazone with strongly concave lateral margins, a slight bulge in the posterior half; margins with numerous small tubercles; posterior margin with a strong medial emargination; the dorsal surface of the posterior third of the metazone barely depressed. Pale with strong black marks across the surface, swirls present at the supra-coxal bulge.

*Prothoracic Legs*: Femur elongate with a slightly concave dorsal margin; tubular in overall shape with the dorsal margin less defined, the anterior and posterior surfaces almost continuous; strongly defined pale to dark banding on posterior (external) surface; anterior (internal) surface with a black band running medially from the base to terminus; the ventral surface pale. Posterior surface of femur with few tubercles. A well developed femoral pit to accommodate terminal posteroventral tibial spine positioned medial and just distal to the first most proximal posteroventral spine and in line with the distal most discoidal spine; pit is black. Posterior prothoracic femoral genicular spine smaller than all other posteroventral spines, originating just proximal to the beginning of the genicular lobe. Prothoracic tibial posteroventral spines with the first (proximal) smallest and the fourth through sixth of similar length, the second and third are longer. Prothoracic coxae smooth; the anterior surface with a broad, black band medially in the proximal half as well as a black spot medially towards the distal terminus.

*Meso- and Metathoracic Legs*: Femora with ventral (posterior) carina; dorsal (anterior) carina faint. Mesotarsi with first segment longer than remaining segments combined.

*Wings*: Forewings mottled with pale, black, and brown coloration; the costal region with pale or whitish and dark irregular banding; two bright pale spots are positioned in the proximal quarter of the discoidal region just posterior to the first radial vein. Forewings colored symmetrically; extending just beyond the terminus of the abdomen. Hindwings smoky black, but translucent, the discoidal region darker and more opaque; the costal region pale proximally and narrowing to the anterior margin distally, the pale margin continuing across the terminal margin of the discoidal region; the margin of the anal region black; the terminus of the discoidal region barely projecting beyond the distal margin of anal region, the distal margin strongly emarginate between the anal and discoidal region.

*Abdomen*: Elongate, tubular with slight widening before posterior narrowing; a smooth and black colored dorsal surface. Supra-anal plate transverse, tapering quickly to a blunt and wide terminus. Subgenital plate irregularly rounded and without styli.

*Genital Complex* (Fig. 52E.1): The main body of ventral left sclerite (L4A) with a slight medial bulge, but mostly a rounded terminus, lacking a distal process (pda); sometimes a depression on the lateral half is present. The apofisis falloid (afa) of the main body of dorsal left sclerite (L4B) elongate and robust, tapering to a strong point, one margin being concave and the other being convex; the apical process (paa) broad, shortened, cylindrical and curved, the terminus with a rounded end. The right dorsal phallomere (fda) of the first sclerite of right phallomere (R1) tapers to a rounded terminus, both margins sclerotized with a broad central membranous gap; the ventral plate (pia) smooth and broad; the ventral process (pva) smooth and tapering to a point distally, one edge straight and the other convex.

**Female.** (Fig. 22B) N=9: Body length 22.70–26.63 (25.22); forewing length 13.52–14.75 (14.15); hindwing length 9.65–11.33 (10.58); pronotum length 7.64–8.32 (7.97); prozone length 2.18–2.41 (2.32); pronotum width 2.36–2.60 (2.48); pronotum narrow width 1.43–1.75 (1.57); head width 5.29–5.66 (5.48); head vertex to clypeus 2.09–2.33 (2.18); frons width 1.94–2.12 (2.03); frons height 0.68–0.90 (0.77); prothoracic femur length 6.90–7.62 (7.28); mesothoracic femur length 9.07–9.82 (9.47); mesothoracic tibia length 7.24–8.13 (7.61); mesothoracic tarsus length 6.31–7.11 (6.75); metathoracic femur length 9.25–9.96 (9.67); metathoracic tibia length 9.89–10.75 (10.17); metathoracic tarsus length 9.46–10.65 (10.32); pronotal elongation measure 0.28–0.30 (0.29); pronotal shape measure 0.29–0.34 (0.31); head shape measure 0.38–0.41 (0.39); frons shape measure 0.34–0.42 (0.38); anteroventral femoral spine count 14–15 (15); anteroventral tibial spine count 10; posteroventral tibial spine count 7.

*Head* (Fig. 43J): Juxta-ocular protuberances large, the apex in the middle; the vertex between the parietal sutures is concave; vertex even or just higher than the dorsal margin of the eyes. Lower region of frons with black marks laterally and a central black mark, two pale gaps on each side of the central black mark; the mandibles pale with brown markings distally.

*Pronotum* (Fig. 48L): As described for males.

*Prothoracic Legs*: Prothoracic coxae smooth; the anterior surface with a broad, black band medially in the proximal half as well as a black spot medially in the distal half.

*Meso- and Metathoracic Legs*: Mesotarsi with first segment as long or slightly longer than remaining segments combined.

*Wings*: Forewings shorter than abdomen, often terminating prior to the narrowing of the abdomen.

*Abdomen*: Widening from first segment until the beginning of the distal half (segments 4–5) when the lateral margins narrow to the terminus, the middle being the broadest region. Tergites without posterolateral tergal projections. Supra-anal plate slightly transverse, rounded.

###### Etymology.

A noun in the genitive case, *Liturgusa milleri* is named for Kelly B. Miller for his contributions to Mantodea sampling and his valuable collaboration.

#### Cursor Group B

##### Liturgusa actuosa

Rehn, 1950

http://species-id.net/wiki/Liturgusa_actuosa

Liturgousa actuosa : Rehn 1950: 377–382, Figs 12–17; Hughes-Schrader 1951: 178, 183–184, 186–187, Tables 1–2, Fig. 2; Hughes-Schrader 1953: 544–554; Callan and Jacobs 1957: 201; Otte 1978: 76;
Liturgousa arcuosa : Hughes-Schrader 1950: 11–14, 38–39, Table 1, Fig. 10; Henderson 1965: 206, 215.
Liturgusa actuosa : Terra 1995: 53; Jantsch 1999: 47; Ehrmann 2002: 206; Otte and Spearman 2005: 132; Agudelo et al. 2007: 116.

###### Type.

Holotype Male. Academy of Natural Sciences of Drexel University, Philadelphia, PA, USA, Type no. 5760.

###### Type locality.

Panama, Barro Colorado Island, Gatun Lake, Canal Zone, January 1, 1948. (Dr. Franz Schrader, no. 637.) (Lat. 9.164966, Long. -79.837098).

###### Material examined.

*Liturgusa actuosa* Rehn, 1950.

**Table:** 

Sex	Type	Country	Label	Latitude Longitude	Code
Male	Holotype	Panama	Barro Colorado Island, Gatun Lake, Canal Zone, January 1, 1948. (Dr. Franz Schrader, no. 637.)	9.164966, -79.837098	ANSP
Male	Paratype	Panama	Barro Colorado, C.Z., 4 I, 49, F. Schrader, Male, 657	9.164966, -79.837098	ANSP 040
Male	Paratype	Panama	Barro Colorado Is. C.Z., F.-S.H. Schrader	9.164966, -79.837098	ANSP 041
Male	Paratype	Panama	Barro Colorado, C.Z., 4 I, 49, F. Schrader, Male, 652	9.164966, -79.837098	ANSP 042
Female	Paratype	Panama	Barro Colorado Is. C.Z., F.-S.H. Schrader	9.164966, -79.837098	ANSP 043
Male	Paratype	Panama	Barro Colorado Is. C.Z., F.-S.H. Schrader	9.164966, -79.837098	ANSP 044
Male	nontype	Panama	Barro Colorado I. C.Z. XI-22-44. Pres. by K.E. Frick Collector	9.164966, -79.837098	CAS 006
Female	nontype	Panama	Barro Colorado I., C.Z., XI-22-44, Pres. by K.E. Frick Collector	9.164966, -79.837098	CAS 011
Male	nontype	Panama	Barro Colorado I. C.Z. XI-22-44. Pres. by K.E. Frick Collector	9.164966, -79.837098	CAS 014
Male	nontype	Panama	C.Z., Barro Colorado Is., 09°10'N, 79°50'W, 27-30 June 1973, Erwin & Hevel Central America Expedition, 1973	9.164966, -79.837098	USNM 012; USNM ENT 00873025
Male	nontype	Panama	Barro Colorado I., Canal Zone, Panama, Jan. 1 1959, CNHM Panama, Zool. Exped. (1959) H.S. Dybas leg.	9.164966, -79.837098	FMNH 012
Nymph	nontype	Panama	Barro Colorado, C.Z. Nov. 6 1930, H.T. Schwarz Coll.	9.164966, -79.837098	AMNH 008

###### Taxonomic history.

Described in 1950 by James Rehn, the species was only known from Barro Colorado Island in the Republic of Panama. At the time, it was considered most similar to *Liturgusa annulipes* rather than the sympatric *Liturgusa cursor*, but this study establishes that *Liturgusa annulipes* is not distributed in Central America and *Liturgusa actuosa* was being compared with an unknown taxon considered as *Liturgusa annulipes* at the time. Interestingly, the species was included in a number of studies focused on chromosomes headed by Sally Hughes-Schrader in the 1940’s and into the 60’s. Other than being included in species records for regional studies and taxonomic lists, the species has received no taxonomic attention since its original description.

###### Diagnosis.

The species is most similar in appearance to *Liturgusa cameroni* and *Liturgusa nubeculosa*, but is much smaller and restricted entirely to Central America. The pronotum is moderately elongate and the coloration of forewing is more evenly brown rather than exhibiting the highly contrasting mottled color patterns as seen in *Liturgusa nubeculosa*. The costal region of the forewing is more pale or green with black markings that are not consistent with regular banding. In addition, the discoidal region of the hindwing projects well beyond the terminal margin of the anal region while the hindwing of *Liturgusa cameroni* and *Liturgusa nubeculosa* are more truncate. The wings extend just shy of the tip of the abdomen in females, but extend slightly beyond in males.

A thorough description of the male and female was provided by Rehn (1950) and can be referred to in addition to the standardized description provided herein.

###### Redescription.

**Figure 23.**
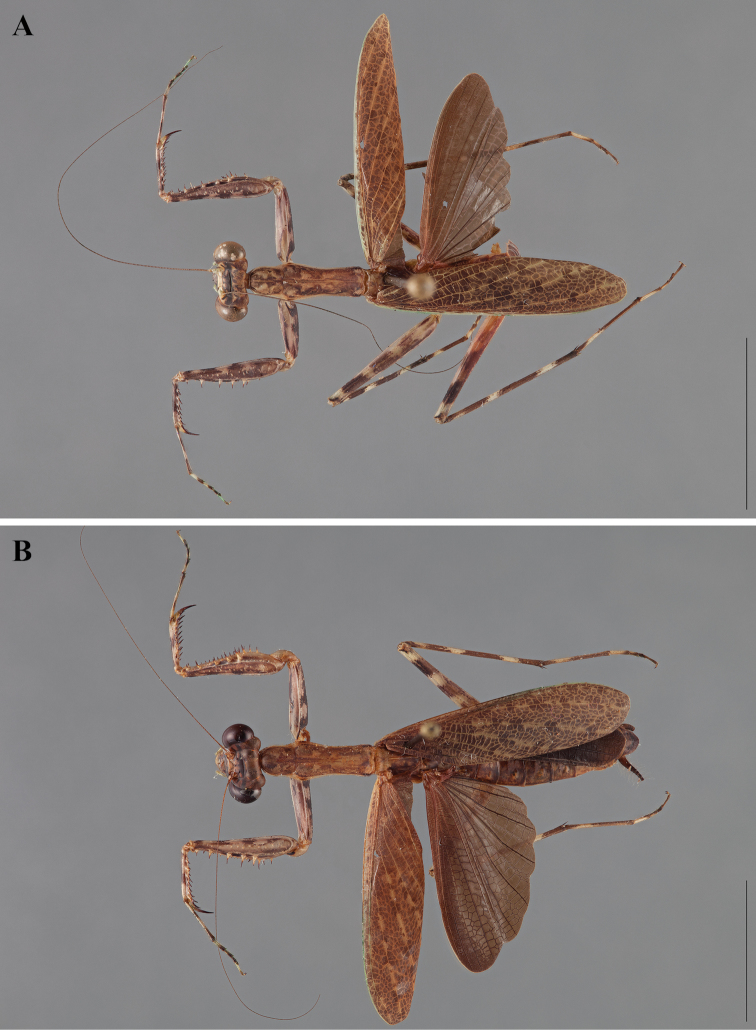
*Liturgusa actuosa* Rehn, 1950, dorsal habitus: **A** male from Barro Colorado Island, Panama (USNM 012) **B** female from Barro Colorado Island, Panama (CAS 011).

**Male.** (Fig. 23A) N=7: Body length 22.22–24.52 (23.50); forewing length 14.46–16.13 (15.44); hindwing length 11.63; pronotum length 6.59–7.08 (6.78); prozone length 1.92–2.12 (2.03); pronotum width 2.19–2.40 (2.28); pronotum narrow width 1.60–1.72 (1.64); head width 4.78–4.95 (4.88); head vertex to clypeus 1.87–2.00 (1.93); frons width 1.66–1.74 (1.70); frons height 0.61–0.73 (0.68); prothoracic femur length 6.10–6.57 (6.32); mesothoracic femur length 7.73–8.49 (8.02); mesothoracic tibia length 6.16–6.48 (6.32); mesothoracic tarsus length 5.44–5.54 (5.50); metathoracic femur length 7.78–8.93 (8.15); metathoracic tibia length 7.11–9.12 (8.59); metathoracic tarsus length 6.41–8.28 (7.71); pronotal elongation measure 0.29–0.31 (0.30); pronotal shape measure 0.33–0.35 (0.34); head shape measure 0.38–0.41 (0.40); frons shape measure 0.36–0.44 (0.40); anteroventral femoral spine count 13–15 (15); anteroventral tibial spine count 9–10 (10); posteroventral tibial spine count 7.

*Head* (Fig. 44A): Juxta-ocular protuberances small, the apex in the middle third; the vertex between the parietal sutures is straight; vertex even with the dorsal margin of the eyes. Frontal suture with a medial carina forming a continuous arc. Ocelli small and protruding on small cuticular mounds. Lateral ocelli oriented outward, a few degrees off perpendicular. Upper margin of clypeus convex, lower margin barely concave. Antennae pale basally fading gradually to dark brown or black near the middle. Broad black band extending straight over the medial carina of the frontal suture; lower region of frons with a broad black band; the clypeus mostly black with pale lateral and lower margins; the mandibles pale with lateral darkening; the labrum mostly dark; the vertex pale with black splotches and juxta-ocular protuberances mostly black anteriorly; the area around the ocelli mostly black. Palpi are pale.

*Pronotum* (Fig. 49A): Moderately elongate with a slightly defined supra-coxal bulge; dorsal surface with very few, very small tubercles that are mostly in the posterior half of the metazone. Prozone with lateral margins that are gently convex, tapering anteriorly; the margins smooth or at most with one or two very small tubercles. Metazone with concave lateral margins; margins with numerous small tubercles; posterior margin medially emarginate; the dorsal surface of the posterior third of the metazone not depressed. Brown coloration dominant, but a few strong black marks.

*Prothoracic Legs*: Femur elongate with a slightly concave dorsal margin; strongly defined pale to dark banding on posterior (external) surface, but dark areas are dominant; area between the posteroventral spines black; anterior (internal) surface with a thin black band running medially from the base to terminus; the ventral surface pale. Posterior surface of femur with few tubercles. A well developed femoral pit to accommodate terminal posteroventral tibial spine positioned medially to the proximal two posteroventral spines and in line with the distal most discoidal spine; pit is entirely black. Posterior prothoracic femoral genicular spine much smaller than posteroventral spines, originating distal to the beginning of the genicular lobe. Prothoracic tibial posteroventral spines with the first (proximal) and fourth through sixth approximately the same size, the second and third being much longer. Prothoracic coxae smooth; the anterior surface with a small black mark in the proximal half positioned medially in both orientations.

*Meso- and Metathoracic Legs*: Femora with ventral (posterior) carina; dorsal (anterior) carina obvious. Mesotarsi with first segment at most equal to remaining segments combined.

*Wings*: Forewings mostly brown with darker splotching, the anterior margin of the costal region green or pale, the veins are mostly pale; lacking highly contrasting coloration and more evenly dark brown; the costal region with dark marks, but not regularly banded. Forewings colored symmetrically. Hindwings smoky black with the anterior and basal region of the discoidal region with faded dark brown coloration; the anal region smoky and translucent; the terminus of the discoidal region projecting well beyond the distal margin of anal region, the wing elongate in appearance.

*Abdomen*: Elongate, tubular with slight widening before posterior narrowing; smooth, a brown and black colored dorsal surface. Supra-anal plate transverse, tapering to a broadly rounded terminus. Subgenital plate irregularly rounded and without styli.

*Genital Complex* (Fig. 52F.1–F.3): The main body of ventral left sclerite (L4A) with a small, dull, cone-shaped distal process (pda) positioned laterally on the terminus and oriented 45 degrees from the central axis of the L4A. The apofisis falloid (afa) of the main body of dorsal left sclerite (L4B) straight and tapering into a short, sharp point; the apical process (paa) cylindrical and curved, the terminus evenly rounded. The right dorsal phallomere (fda) of the first sclerite of right phallomere (R1) tapers to a rounded, membranous terminus; the ventral plate (pia) strongly sclerotized and narrow, with a few strongly defined and curved grooves; the ventral process (pva) small and irregularly shaped, tapering to a point with a rough surface oriented towards the pia.

###### Redescription.

**Female.** (Fig. 23B) N=2: Body length 26.90–28.73 (27.81); forewing length 16.84–17.63 (17.23); hindwing length 14.55; pronotum length 7.79–7.97 (7.88); prozone length 2.37–2.46 (2.42); pronotum width 2.74–2.78 (2.76); pronotum narrow width 1.93–1.94 (1.94); head width 5.68–5.72 (5.70); head vertex to clypeus 2.46–2.51 (2.48); frons width 2.15; frons height 0.82–0.83 (0.82); prothoracic femur length 7.43–7.66 (7.54); mesothoracic femur length 8.43; mesothoracic tibia length 6.84; mesothoracic tarsus length 5.98; metathoracic femur length 8.43–8.62 (8.52); metathoracic tibia length 9.71–9.77 (9.74); metathoracic tarsus length 8.73; pronotal elongation measure 0.30–0.31 (0.31); pronotal shape measure 0.35; head shape measure 0.43–0.44 (0.43); frons shape measure 0.38; anteroventral femoral spine count 14–15 (14); anteroventral tibial spine count 10; posteroventral tibial spine count 7.

*Head* (Fig. 44B): Juxta-ocular protuberances moderately pronounced, the apex in the middle third; the vertex between the parietal sutures is slightly concave; vertex well above the dorsal margin of the eyes. Ocelli small and laying nearly flat. Antennae pale brown basally, fading gradually to dark brown. Clypeus mostly pale with lateral and lower margins that are dark brown, but pale on the very edge; the labrum mostly pale; the vertex and juxta-ocular protuberances brown with disperse black splotches. Palpi are pale.

*Pronotum* (Fig. 49B): Dorsal surface with few, very small tubercles.

*Prothoracic Legs*: Femur moderately elongate with a slightly concave dorsal margin.

*Meso- and Metathoracic Legs*: As described for males.

*Wings*: As described for males.

*Abdomen*: Widening from first segment until the beginning of the distal half (segments 4–5) when the lateral margins narrow to the terminus, the middle being the broadest region. Tergites without posterolateral tergal projections. Supra-anal plate almost as long as wide, evenly rounded.

##### Liturgusa algorei sp. n.

http://zoobank.org/110C8CAF-E80A-4A75-BCC4-C88321A26E76

http://species-id.net/wiki/Liturgusa_algorei

###### Type.

Holotype Male, pinned. Cleveland Museum of Natural History, Cleveland, OH, USA.

###### Type locality.

Peru: Loreto Province, Madre Selva Biological Research Station, -3.62096, -72.24744, 10-17 February 2013, Coll: G.J. Svenson, Tissue 011, GSMC004007. (Lat. -3.62096, Long. -72.24744).

###### Material examined.

*Liturgusa algorei* sp. n.

**Table:** 

Sex	Type	Country	Label	Latitude Longitude	Code
Male	Holotype	Peru	Loreto Province, Madre Selva Biological Research Station - Tissue 022	-3.620960, -72.247440	GSMC004007
Female	Allotype	Peru	Loreto Province, Puerto Almendra	-3.830525, -73.374000	GSMC004011
Male	Paratype	Peru	Loreto Province, Madre Selva Biological Research Station - Tissue 011	-3.620960, -72.247440	GSMC004000
Female	Paratype	Peru	Loreto Province, Madre Selva Biological Research Station - Tissue 029	-3.620960, -72.247440	GSMC004021
Female	Paratype	Peru	Loreto Province, Madre Selva Biological Research Station - Tissue 023	-3.620960, -72.247440	GSMC004023
Male	Paratype	Peru	Loreto Province, Madre Selva Biological Research Station - Tissue 001	-3.620960, -72.247440	GSMC004026
Male	Paratype	Peru	Loreto Province, Puerto Almendra - Tissue 042	-3.830525, -73.374000	GSMC004029
Male	Paratype	Peru	Loreto Province, Madre Selva Biological Research Station	-3.620960, -72.247440	GSMC004033
Male	Paratype	Equador	Napo Rio Aguarico, San Pablo Tumba Emilio, VI-1985, K. Riede Rec.		MNHN 037
Male	Paratype	Peru	Loreto, Pebas, River Amazonas, -3.329066°S, -71.854168°E, 28 Feb 2010, Coll: J.J. Ramirez	-3.329066, -71.854168	MNHN 095
Female	Paratype	Ecuador	Napo: Yasuri Natl. Pk. 0°40'S, 76°20'W., Tiputini Biodiversity Station, 14-19 FEB 1998. UofT Field Course, per DC Darling. ROM 980000	-0.637742, -76.150216	ROM 001

###### Natural history.

Males and females found in local abundance at the Madre Selva Biological Research Station in the Loreto Province, Peru. The species was living in sympatry with *Liturgusa krattorum* on the same smooth bark, medium diameter trees. The species was easily collected during the day and often found at reachable heights in lower sections of the tree.

###### Diagnosis.

Extremely similar in coloration, size, and distribution to *Liturgusa krattorum*. Posterior prothoracic femoral genicular spine of female tiny, much smaller than seen in any other species. Overall color mottling darker with greater contrast, the hindwings being darkly smoke colored or nearly black and never rusty as in *Liturgusa krattorum*.

###### Description.

**Figure 24.**
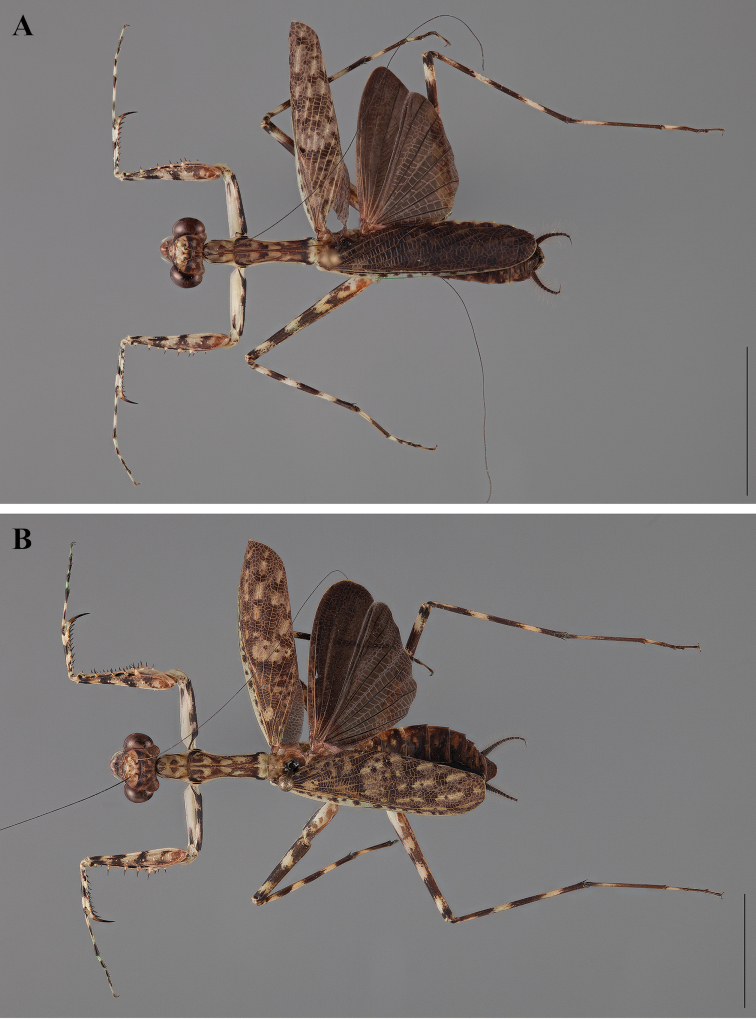
*Liturgusa algorei* sp. n., dorsal habitus: **A** holotype male from Loreto, Peru (CLEV GSMC004007) **B** allotype female from Loreto, Peru (CLEV GSMC004011).

**Male.** (Fig. 24A) N=7: Body length 22.00–24.36 (23.42); forewing length 13.17–14.98 (14.31); hindwing length 10.89–11.64 (11.28); pronotum length 7.11–7.79 (7.52); prozone length 1.94–2.18 (2.08); pronotum width 2.15–2.33 (2.23); pronotum narrow width 1.52–1.68 (1.57); head width 4.51–5.10 (4.83); head vertex to clypeus 1.89–2.03 (1.97); frons width 1.69–1.77 (1.72); frons height 0.63–0.75 (0.69); prothoracic femur length 6.20–7.00 (6.70); mesothoracic femur length 7.07–10.52 (9.43); mesothoracic tibia length 5.67–8.22 (7.37); mesothoracic tarsus length 5.36–6.94 (6.36); metathoracic femur length 8.97–10.71 (9.91); metathoracic tibia length 9.06–10.85 (9.88); metathoracic tarsus length 9.77–11.08 (10.44); pronotal elongation measure 0.27–0.28 (0.28); pronotal shape measure 0.28–0.31 (0.30); head shape measure 0.39–0.44 (0.41); frons shape measure 0.36–0.44 (0.40); anteroventral femoral spine count 15–16 (15); anteroventral tibial spine count 10–11 (10); posteroventral tibial spine count 7.

*Head* (Fig. 44C): Juxta-ocular protuberances large, the apex in the lateral third; the vertex between the parietal sutures is straight; vertex just below the dorsal margin of the eyes. Frontal suture with a medial carina forming a continuous arc. Ocelli small and protruding on small cuticular mounds. Lateral ocelli oriented outward, a few degrees off perpendicular. Upper margin of clypeus convex, lower margin slightly concave with a medial bulge. Antennae pale basally fading quickly to black. Broad black band extending from eye to eye over the medial carina of the frontal suture; lower region of frons with a transverse black band; vertex and juxta-ocular protuberances speckled with brown and black marks. Palpi are pale.

*Pronotum* (Fig. 49C): Elongate with a moderately defined supra-coxal bulge; dorsal surface entirely smooth. Prozone with lateral margins that are parallel, tapering anteriorly; the margins smooth. Metazone with concave lateral margins; margins smooth or at most with very small and blunt tubercles; posterior margin medially emarginate; the dorsal surface of the posterior half slightly depressed, evenly concave.

*Prothoracic Legs*: Femur elongate with a slightly concave dorsal margin; strongly defined pale to dark banding on posterior (external) surface; anterior (internal) surface with a black band running medially from the base to terminus; the ventral surface pale. Posterior surface of femur with few tubercles. A well developed femoral pit to accommodate terminal posteroventral tibial spine positioned medially to the proximal two posteroventral spines and in line with the distal most discoidal spine; pit is colored black. Posterior prothoracic femoral genicular spine much smaller than posteroventral spines, originating at the beginning of the genicular lobe. Prothoracic tibial posteroventral spines with the first (proximal) smallest and the second through sixth of similar length, the second and third may be slightly longer. Prothoracic coxae smooth; the anterior surface with a medial black band centrally located in the central 75% of the surface; a very small black spot on the distal lobe.

*Meso- and Metathoracic Legs*: Femora with faint ventral (posterior) carina; dorsal (anterior) carina faint. Mesotarsi with first segment equal to remaining segments combined.

*Wings*: Forewings mottled with brown, black, and pale coloration; the costal region with light to dark irregular banding. Forewings colored asymmetrically, one being mottled the other is blackened with the mottled pattern still slightly visible. Hindwings smoky black with rusty coloration at the very base of the discoidal region; the terminus of the discoidal region projecting well beyond the distal margin of anal region.

*Abdomen*: Elongate, tubular with slight widening before posterior narrowing; smooth surface. Supra-anal plate transverse, tapering to a broadly rounded terminus. Subgenital plate irregularly rounded and without styli.

*Genital Complex* (Fig. 52G.1–G.2): The main body of ventral left sclerite (L4A) with a prominent, distal process (pda) positioned medially and tapering to a sclerotized point that is oriented in line with the central axis of L4A; the margins of the pda sclerotized and either straight or convex. The apofisis falloid (afa) of the main body of dorsal left sclerite (L4B) tapering to an elongate, sharp point that is well sclerotized and oriented approximately 35 degrees from the central axis; the apical process (paa) cylindrical and gently curved, the terminus being an expanded, blunt knob. The right dorsal phallomere (fda) of the first sclerite of right phallomere (R1) tapers to a rounded terminus, the lateral margins being sclerotized and robust with a medial, membranous gap at the terminus; the ventral plate (pia) strongly sclerotized, broadened proximally with large curved grooves; the ventral process (pva) c-shaped, broad, and rounded distally, the surface slightly rough.

**Female.** (Fig. 24B) N=4: Body length 31.26–32.15 (31.68); forewing length 17.99–19.15 (18.61); hindwing length 14.78–15.39 (15.08); pronotum length 9.11–9.99 (9.74); prozone length 2.70–2.88 (2.81); pronotum width 2.94–3.14 (3.07); pronotum narrow width 1.84–2.08 (1.97); head width 5.75–6.28 (6.12); head vertex to clypeus 2.60–2.76 (2.69); frons width 2.31–2.54 (2.43); frons height 0.89–0.96 (0.92); prothoracic femur length 8.75–9.23 (8.99); mesothoracic femur length 11.24–12.09 (11.81); mesothoracic tibia length 8.58–9.39 (9.13); mesothoracic tarsus length 7.73–8.22 (7.95); metathoracic femur length 11.34–12.45 (12.01); metathoracic tibia length 11.62–12.49 (12.20); metathoracic tarsus length 10.81–12.5 (11.95); pronotal elongation measure 0.28–0.30 (0.29); pronotal shape measure 0.29–0.34 (0.32); head shape measure 0.42–0.47 (0.44); frons shape measure 0.37–0.39 (0.38); anteroventral femoral spine count 14–15 (15); anteroventral tibial spine count 10; posteroventral tibial spine count 7.

*Head* (Fig. 44D): The vertex between the parietal sutures is slightly concave; vertex just above the dorsal margin of the eyes. Ocelli small and laying flatly on the surface. Lower margin of clypeus straight with a slight medial bulge. Broad black band extending from eye to eye over the medial carina of the frontal suture, but with medial gap; lower region of frons with lateral black markings, a pale medial region. Palpi darkened terminally.

*Pronotum* (Fig. 49D): Margins of metazone with numerous small, sharp tubercles.

*Prothoracic Legs*: Posterior prothoracic femoral genicular spine tiny, barely present, originating at the beginning of the genicular lobe. Prothoracic tibial posteroventral spines with the first (proximal) smallest and the fourth through sixth of similar length, the second and third longer. Anterior surface of prothoracic coxae with a black medial mark in the proximal half.

*Meso- and Metathoracic Legs*: Femora with ventral (posterior) and dorsal (anterior) carina present.

*Wings*: Forewings colored symmetrically. Hindwings smoky black with rusty coloration at the very base of the discoidal region, sometimes extending distally along anterior margin; the terminus of the discoidal region projecting slightly beyond the distal margin of anal region.

*Abdomen*: Widening from first segment until the beginning of the distal half (segments 5–6) when the lateral margins narrow to the terminus, the middle being the broadest region. Tergites with slight posterolateral tergal projections in the distal half of the abdomen. Supra-anal plate slightly transverse, tapering to a rounded terminus.

###### Etymology.

A noun in the genitive case, *Liturgusa algorei* is named for Albert Arnold 'Al' Gore, Jr., former Vice President of the United States of America, for his environmental activism including his efforts to raise public awareness of global climate change.

##### Liturgusa cameroni sp. n.

http://zoobank.org/89C447CA-6F11-4E4A-AACE-DF83195907F1

http://species-id.net/wiki/Liturgusa_cameroni

###### Type.

Holotype Male, pinned. American Museum of Natural History, New York, NY, USA.

###### Type locality.

Venezuela: Rancho Grande, nr. Maracay, Ven. 17-VI-1948 (Lat. 10.350000, Long. -67.683330).

###### Material examined.

*Liturgusa cameroni* sp. n.

**Table:** 

Sex	Type	Country	Label	Latitude Longitude	Code
Male	Holotype	Guyana	Kartabo, Bartica District, 1921	6.242050, -59.306552	ANSP 047
Female	Allotype	Venezuela	Estado de Aragua, Rancho Grande, N. of Maracay, 1300 m, 20.II.1987, Edward S. Ross	10.350000, -67.683330	CAS 003
Male	Paratype	Venezuela	Rancho Grande, nr. Maracay, 17-VI-1948	10.350000, -67.683330	AMNH 016
Male	Paratype	Venezuela	Rancho Grande, nr. Maracay, 23-VI-1946	10.350000, -67.683330	AMNH 017
Female	Paratype	Venezuela	San Esteban, X-XI 1910, M.A. Carriker Jr.	10.422557, -68.012585	ANSP 019
Male	Paratype	Guyana	Kartabo, Bartica District, 1921	6.242050, -59.306552	ANSP 045
Female	Paratype	Venezuela	San Rafael, Cumanacoa, Sucre, G. Netting, Nov 29 1929	10.227884, -63.947462	ANSP 114
Female	Paratype	Venezuela	La Guaira, Robinson, S.H. Scudder Collection	10.596235, -66.934786	ANSP 117
Female	Paratype	Venezuela	La Guaira, Robinson, S.H. Scudder Collection	10.596235, -66.934786	ANSP 118
Male	Paratype	Venezuela	Petare, 880 mtrs., 21.V.1926, H.E. Box Collector	10.500706, -66.799504	BMNH 075
Female	Paratype	Venezuela	Petare, 880 mtrs., 21.V.1926, H.E. Box Collector	10.500706, -66.799504	BMNH 078
Female	Paratype	Venezuela	Petare, 880 mtrs., 21.V.1926, H.E. Box Collector	10.500706, -66.799504	BMNH 079
Female	Paratype	Venezuela	Petare, 880 mtrs., 21.V.1926, H.E. Box Collector	10.500706, -66.799504	BMNH 080
Male	Paratype	Venezuela	Petare, 880 mtrs., 21.V.1926, H.E. Box Collector	10.500706, -66.799504	BMNH 085
Nymph	nontype	Venezuela	Petare, 880 mtrs., 21.V.1926, H.E. Box Collector	10.500706, -66.799504	BMNH 077

###### Diagnosis.

Nearly identical to *Liturgusa nubeculosa*, the primary distinguishing characteristics being male genital features, distribution and the hindwing. It is one of the largest *Liturgusa* species with robust legs, highly contrasting mottled forewings and strong banding. A feature easily seen on most specimens that is capable of distinguishing the species from *Liturgusa nubeculosa* is that the discoidal region of the hindwing does not extend much beyond the distal margin of the anal region, which gives the wing a more truncate appearance compared to the hindwing of *Liturgusa nubeculosa*.

###### Description.

**Figure 25.**
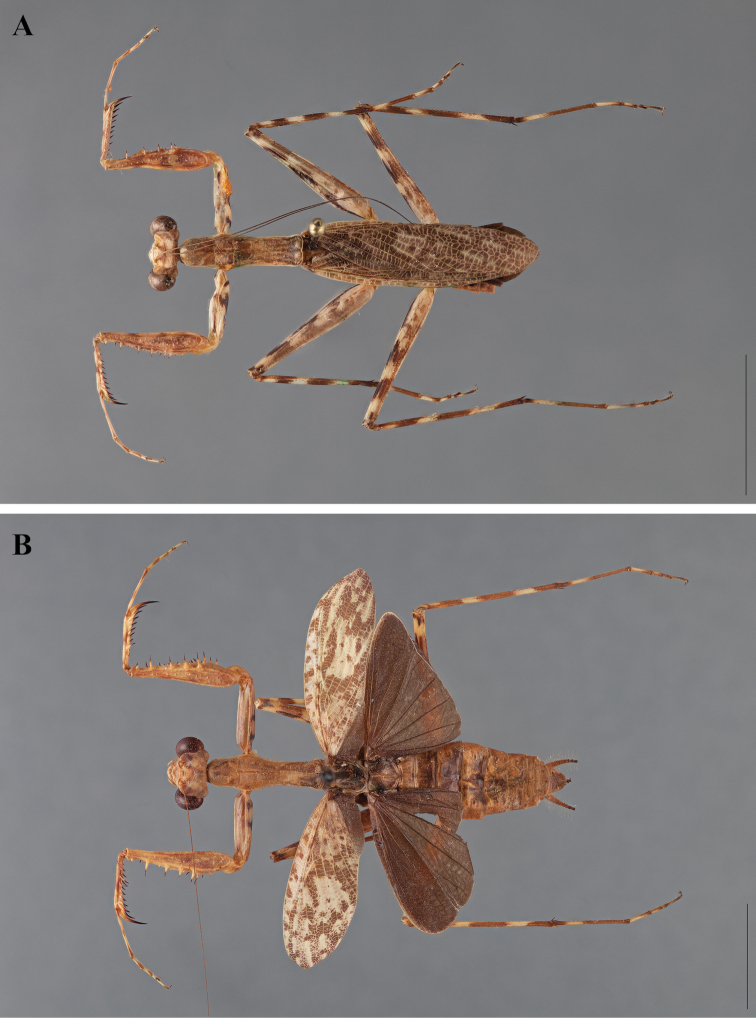
*Liturgusa cameroni* sp. n., dorsal habitus: **A** holotype male from Guyana (ANSP 047) **B** allotype female from Maracay, Venezuela (CAS 003).

**Male.** (Fig. 25A) N=5: Body length 21.88–26.52 (24.66); forewing length 13.40–16.34 (15.19); pronotum length 7.21–8.74 (8.09); prozone length 2.08–2.49 (2.31); pronotum width 2.23–2.68 (2.48); pronotum narrow width 1.73–2.01 (1.88); head width 4.87–5.54 (5.23); head vertex to clypeus 1.80–2.12 (1.97); frons width 1.76–2.00 (1.89); frons height 0.55–0.73 (0.65); prothoracic femur length 6.44–8.10 (7.29); mesothoracic femur length 8.54–10.93 (9.89); mesothoracic tibia length 6.66–8.49 (7.96); mesothoracic tarsus length 5.57–7.51 (6.76); metathoracic femur length 7.27–11.64 (9.78); metathoracic tibia length 8.97–11.76 (10.55); metathoracic tarsus length 10.66; pronotal elongation measure 0.28–0.29 (0.29); pronotal shape measure 0.30–0.32 (0.31); head shape measure 0.36–0.39 (0.38); frons shape measure 0.30–0.38 (0.34); anteroventral femoral spine count 15–16 (16); anteroventral tibial spine count 10; posteroventral tibial spine count 7.

*Head* (Fig. 44E): Transverse, juxta-ocular protuberances small, the apex in the lateral third; the vertex between the parietal sutures is straight and broad; vertex even or slightly below the dorsal margin of the eyes. Frontal suture with a medial carina forming an arc the middle with an obtuse angle. Ocelli small and protruding on small cuticular mounds. Lateral ocelli oriented outward. Frons highly transverse, the region below the antennal insertions very narrow; lower medial region with black marking. Clypeus transverse; upper margin straight, lower margin concave. Antennae pale basally, gradually fading to black. Upper region of clypeus pale, lower half brown; mandibles pale or brown; the vertex and juxta-ocular protuberances splotched with brown; the area adjacent to lateral ocelli black. Palpi are pale.

*Pronotum* (Fig. 49E): Elongate, but robust with a slightly defined supra-coxal bulge; dorsal surface with numerous small tubercles. Prozone elongate with lateral margins that are near parallel, tapering anteriorly; the margins smooth. Metazone with concave lateral margins, a slight bulge in the posterior half; margins with few small, blunt tubercles; posterior margin slightly emarginate medially; the anterior half of dorsal surface raised, the dorsal surface of the posterior half depressed. Mostly brown with black marks.

*Prothoracic Legs*: Femur elongate with a slightly concave dorsal margin; strongly defined pale to dark banding on posterior (external) surface; anterior (internal) surface with a thin, black band running medially from the base to terminus; the ventral surface pale. Posterior surface of femur with few tubercles. A well developed femoral pit to accommodate terminal posteroventral tibial spine positioned just distal and medial to the most proximal posteroventral spine; pit is dark. Posterior prothoracic femoral genicular spine much smaller than posteroventral spines, originating distal to the beginning of the genicular lobe. Prothoracic tibial posteroventral spines with the first (proximal) smallest and the third through sixth of similar length, the second longer. Prothoracic coxae smooth; the anterior surface with a small, black band medially in the proximal half as well as a small black spot medially towards the distal terminus.

*Meso- and Metathoracic Legs*: Femora with strongly pronounced ventral (posterior) and dorsal (anterior) carina; posterior surface with medial carina in the distal two thirds. Mesotarsi with first segment at most as long as the remaining segments combined.

*Wings*: Forewings mottled with brown, green, and pale coloration; the costal region with pale and dark irregular banding. Forewings sometimes colored asymmetrically, one being mottled the other is rust colored with the mottled pattern still visible. Hindwings smoky black and brown, opaque; the terminus of the discoidal region not projecting much beyond the distal margin of anal region, giving the wing a truncate appearance.

*Abdomen*: Elongate, tubular with slight widening before posterior narrowing; smooth. Tergites with small posterolateral tergal projections. Supra-anal plate transverse, tapering to a broadly rounded terminus. Subgenital plate irregularly rounded and without styli.

*Genital Complex* (Fig. 52H.1): The main body of ventral left sclerite (L4A) with a sharply pointed distal process (pda) resembling a curved tooth positioned laterally, the terminal margin of the L4A centrally rounded before a depression that leads to the pda. The apofisis falloid (afa) of the main body of dorsal left sclerite (L4B) broad and tapering quickly to a point; the apical process (paa) broad, cylindrical and curved, the terminus evenly rounded. The right dorsal phallomere (fda) of the first sclerite of right phallomere (R1) tapers to a rounded, membranous terminus with fine setae; the ventral plate (pia) strongly sclerotized, broadening proximally with strong curved grooves; the ventral process (pva) c-shaped, tapering to a point distally.

**Female.** (Fig. 25B) N=6: Body length 27.44–35.27 (31.21); forewing length 15.35–19.92 (17.30); hindwing length 14.68; pronotum length 9.07–11.12 (10.01); prozone length 2.59–3.17 (2.83); pronotum width 3.00–3.47 (3.23); pronotum narrow width 2.19–2.43 (2.27); head width 6.10–6.86 (6.48); head vertex to clypeus 2.48–2.78 (2.63); frons width 2.36–2.61 (2.46); frons height 0.78–0.97 (0.86); prothoracic femur length 8.02–10.43 (9.04); mesothoracic femur length 9.81–12.73 (11.14); mesothoracic tibia length 7.96–10.25 (8.94); mesothoracic tarsus length 6.96–8.69 (7.63); metathoracic femur length 9.70–12.13 (10.83); metathoracic tibia length 10.54–14.05 (12.09); metathoracic tarsus length 9.90–12.31 (10.99); pronotal elongation measure 0.28–0.29 (0.28); pronotal shape measure 0.31–0.34 (0.32); head shape measure 0.40–0.42 (0.41); frons shape measure 0.33–0.39 (0.35); anteroventral femoral spine count 16–17 (16); anteroventral tibial spine count 10; posteroventral tibial spine count 7.

*Head* (Fig. 44F): Slightly transverse, juxta-ocular protuberances medium, the apex in the middle third; the vertex between the parietal sutures is slightly concave and broad; vertex well above the dorsal margin of the eyes. Frontal suture with a medial carina forming a continuous arc.

*Pronotum* (Fig. 49F): Elongate, but robust with a moderately defined supra-coxal bulge. Metazone with concave lateral margins, a slight bulge in the posterior half; margins with small, blunt tubercles; posterior margin slightly emarginate medially.

*Prothoracic Legs*: Femur with very long posteroventral spines. A well developed femoral pit to accommodate terminal posteroventral tibial spine positioned medially to the proximal two posteroventral spines and in line with the distal most discoidal spine; pit is very deep, but pigmented pale. Prothoracic tibial posteroventral spines with the first (proximal) very small and the third through sixth of similar length, the second very long, nearly the same length as the terminal spine.

*Meso- and Metathoracic Legs*: Mesotarsi with first segment shorter than the remaining segments combined.

*Wings*: Forewings broadened with a widened costal region, extending at most to the tip of the abdomen, but usually shorter; mottled with highly contrasting brown and pale coloration; the costal region with pale and dark irregular banding. Forewings colored symmetrically. Hindwings smoky black and brown, opaque, the anterior margin more pale.

*Abdomen*: Broad, widening from first segment until the beginning of the distal half (segment 4) when the lateral margins narrow gradually to the terminus, the middle being the broadest region. Tergites in the posterior half with small posterolateral tergal projections. Supra-anal plate as long as wide, evenly rounded.

###### Etymology.

A noun in the genitive case, *Liturgusa cameroni* is named for Stephen L. Cameron for his contributions to Mantodea sampling and his valuable collaboration.

##### Liturgusa krattorum sp. n.

http://zoobank.org/0DB60DC2-3747-4610-869C-E2D4703011B1

http://species-id.net/wiki/Liturgusa_krattorum

###### Type.

Holotype Male, pinned. Cleveland Museum of Natural History, Cleveland, OH, USA.

###### Type locality.

Peru: Loreto Province, Madre Selva Biological Research Station, -3.62096, -72.24744, 10-17 February 2013, Coll: G.J. Svenson, Tissue 005, GSMC004004. (Lat. -3.62096, Long. -72.24744).

###### Material examined.

*Liturgusa krattorum* sp. n.

**Table:** 

Sex	Type	Country	Label	Latitude Longitude	Code
Male	Holotype	Peru	Loreto Province, Madre Selva Biological Research Station, -3.62096, -72.24744, 10–17 February 2013, Coll: G.J. Svenson - Tissue 005	-3.620960, -72.247440	GSMC004004
Female	Allotype	Peru	Loreto Province, Puerto Almendra - Tissue 040	-3.830525, -73.374000	GSMC004032
Female	Paratype	Peru	Rio Maranon, XII-30, F 6079, H. Bassler Collection	-4.832821, -76.639033	AMNH 003
Male	Paratype	Peru	Monson Valley, Tingo Maria, X-19-1954, E.I. Schlinger & E.S. Ross collectors	-9.314153, -76.006745	CAS 008
Female	Paratype	Peru	20 mi. W. of Pucalipa, X-3-1954, E.I. Schlinger & E.S. Ross collectors	-8.354749, -74.552113	CAS 019
Male	Paratype	Peru	Loreto Province, Madre Selva Biological Research Station, -3.62096, -72.24744, 10-17 February 2013, Coll: G.J. Svenson - Tissue 026	-3.620960, -72.247440	GSMC004001
Male	Paratype	Peru	Loreto Province, Puerto Almendra - Tissue 033	-3.830525, -73.374000	GSMC004018
Male	Paratype	Peru	Loreto Province, Madre Selva Biological Research Station, -3.62096, -72.24744, 10-17 February 2013, Coll: G.J. Svenson - Tissue 002	-3.620960, -72.247440	GSMC004034
Male	Paratype	Peru	Loreto Province, Madre Selva Biological Research Station, -3.62096, -72.24744, 10-17 February 2013, Coll: G.J. Svenson - Tissue 009	-3.620960, -72.247440	GSMC004035
Male	Paratype	Peru	Dept. Loreto Rio Yubineto, 1-VII - 1.VIII - 1978, M. Descamps rec.	-0.999780, -74.253238	MNHN 024
Female	Paratype	Colombia	Dept. Amazonas, 30 KM. Aval de la Chorrera, Rio Igara - Parana, 1.2.II.1974, J. Desplats Rec.	-0.754819, -73.007213	MNHN 082
Female	Paratype	Peru	Dept. Loreto Rio Yubineto, 1-VII - 1.VIII - 1978, M. Descamps rec.	-0.999780, -74.253238	MNHN 084
Male	Paratype	Peru	Loreto, Pebas, River Amazonas, -3.329066°S, -71.854168°E, 28 Feb 2010, Coll: J.J. Ramirez	-3.329066, -71.854168	MNHN 093
Female	Paratype	Peru	Loreto, Iquitos, Nov. 2010, Collection Stiewe	-3.741872, -73.272190	MSMC 002
Male	Paratype	Ecuador	Pastaza; Ashuara, Rio Macuma, 10km from Rio Morona, 300m, VII:7-16:1971, le. B. Malkin	-2.753512, -77.444899	FMNH 004
Female	Paratype	Ecuador	Pastaza; Ashuara, Rio Macuma, 10km from Rio Morona, 300m, VII:7-16:1971, le. B. Malkin	-2.753512, -77.444899	FMNH 011

###### Natural history.

Males and female found in local abundance at the Madre Selva Biological Research Station in the Loreto Province, Peru. The species was living in sympatry with *Liturgusa algorei* on the same smooth bark, medium diameter trees. The species was easily collected during the day and often found at reachable heights in lower sections of the tree.

###### Diagnosis.

Most similar to *Liturgusa purus* and *Liturgusa algorei* in body shape and slenderness, but far more green in coloration across head, pronotum, and prothoracic legs with highly contrasting banding across the body. In addition, the head is mostly pale, but the lower portion of the frons, the clypeus, mandibles and labrum are darkly colored. Forewings are browner than other two species with a pronounced green and dark banded costal region. Hindwings are darker than *Liturgusa purus*, but more rusty than *Liturgusa algorei*. In addition, the prothoracic femoral posterior genicular spine in females is elongate, but shorter than posteroventral spines, but relatively much longer than that seen in *Liturgusa algorei*.

###### Description.

**Figure 26.**
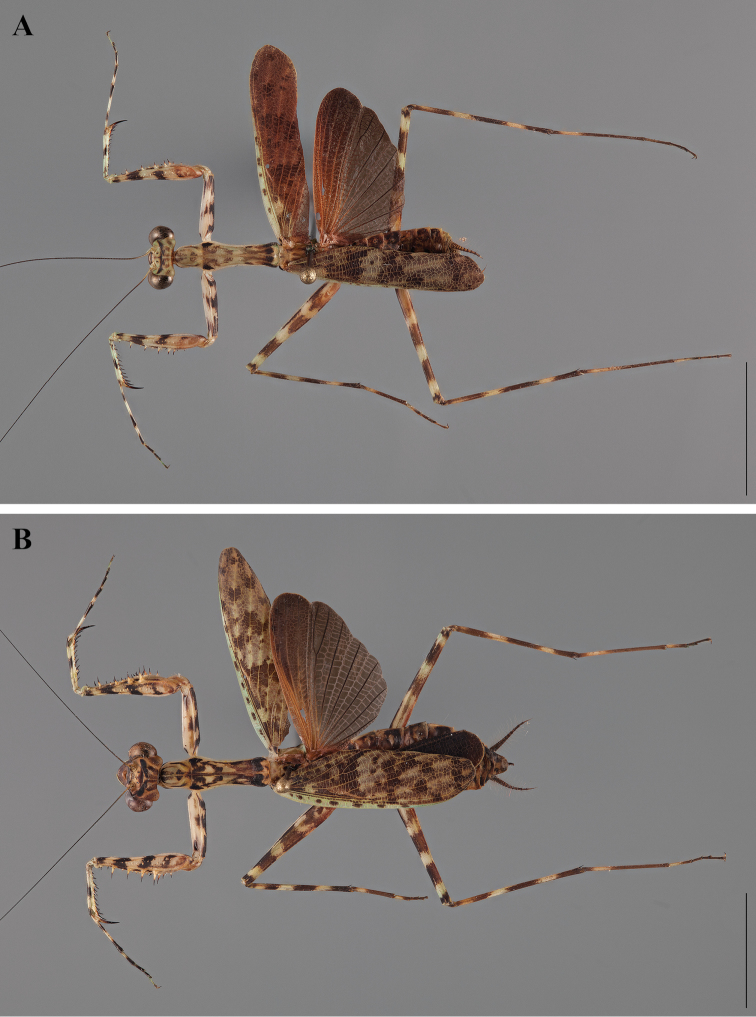
*Liturgusa krattorum* sp. n., dorsal habitus: **A** holotype male from Loreto, Peru (CLEV GSMC004004) **B** allotype female from Loreto, Peru (CLEV GSMC004032).

**Male.** (Fig. 26A) N=8: Body length 23.24–24.68 (23.94); forewing length 14.39–15.50 (14.91); hindwing length 11.33–12.48 (11.81); pronotum length 7.27–7.85 (7.62); prozone length 2.06–2.24 (2.13); pronotum width 2.21–2.41 (2.33); pronotum narrow width 1.51–1.71 (1.59); head width 4.70–4.92 (4.85); head vertex to clypeus 1.88–2.03 (1.96); frons width 1.64–1.77 (1.70); frons height 0.63–0.73 (0.67); prothoracic femur length 6.62–7.09 (6.90); mesothoracic femur length 9.40–10.17 (9.79); mesothoracic tibia length 7.46–8.28 (7.95); mesothoracic tarsus length 6.69–7.45 (7.06); metathoracic femur length 9.95–10.80 (10.28); metathoracic tibia length 9.93–10.86 (10.50); metathoracic tarsus length 10.12–11.52 (11.00); pronotal elongation measure 0.27–0.29 (0.28); pronotal shape measure 0.29–0.32 (0.30); head shape measure 0.40–0.42 (0.40); frons shape measure 0.36–0.42 (0.40); anteroventral femoral spine count 15–17 (16); anteroventral tibial spine count 10–11 (10); posteroventral tibial spine count 7.

*Head* (Fig. 44G): Transverse, juxta-ocular protuberances medium, the apex in the lateral third; the vertex between the parietal sutures is straight; vertex even with the dorsal margin of the eyes. Frontal suture with a medial carina forming a continuous arc. Ocelli small and protruding on a small cuticular mound, but almost flat. Lateral ocelli oriented outward, a few degrees off perpendicular. Upper margin of clypeus barely convex, lower margin straight. Antennae pale basally fading quickly to black. Broad black band extending straight over the medial carina of the frontal suture; lower region of frons with two black marks separated by a pale gap centrally; the clypeus brown on lateral and lower region; the mandibles and labrum dark brown; the vertex and juxta-ocular protuberances splotched with brown and green; the area adjacent to lateral ocelli black. Palpi are pale.

*Pronotum* (Fig. 49G): Elongate with a moderately defined supra-coxal bulge; dorsal surface entirely smooth. Prozone with lateral margins that are near parallel, tapering anteriorly; the margins smooth. Metazone with concave lateral margins, a slight bulge in the posterior half; margins with numerous small tubercles; posterior margin medially emarginate; the dorsal surface of the posterior half moderately depressed. Green coloration dominant, but a few strong black marks.

*Prothoracic Legs*: Femur elongate with a slightly concave dorsal margin; strongly defined pale to dark banding on posterior (external) surface; anterior (internal) surface with a black band running medially from the base to terminus; the ventral surface pale. Posterior surface of femur with few tubercles. A well developed femoral pit to accommodate terminal posteroventral tibial spine positioned medially to the proximal two posteroventral spines and in line with the distal most discoidal spine; pit is pale. Posterior prothoracic femoral genicular spine at most a quarter the length of the posteroventral spines, originating just distal to the beginning of the genicular lobe. Prothoracic tibial posteroventral spines with the first (proximal) smallest and the second through sixth of similar length, the second and third are slightly longer than adjacent spines. Prothoracic coxae smooth; the anterior surface with a broad, black band medially in the proximal half as well as a black spot medially towards the distal terminus.

*Meso- and Metathoracic Legs*: Femora with ventral (posterior) carina; dorsal (anterior) carina faint. Mesotarsi with first segment approximately equal to remaining segments combined.

*Wings*: Forewings mottled with brown, green, and pale coloration; the costal region with green and dark irregular banding. Forewings colored asymmetrically, one being mottled the other is rust colored with the mottled pattern still visible. Hindwings with a red or rusty color in the discoidal region, darkening distally to black; the anal region rusty basally and fading to a smoky grey and translucent medially before becoming black along the distal margin; the terminus of the discoidal region projecting well beyond the distal margin of anal region, the wings appearing elongate.

*Abdomen*: Elongate, tubular with slight widening before posterior narrowing; smooth, black and green colored dorsal surface. Supra-anal plate transverse, tapering to a broadly rounded terminus. Subgenital plate irregularly rounded and without styli.

*Genital Complex* (Fig. 52I.1): The main body of ventral left sclerite (L4A) with a dull or sharply pointed, sickle shaped distal process (pda) positioned laterally, the terminal margin of the L4A centrally rounded before a depression that leads to the pda. The apofisis falloid (afa) of the main body of dorsal left sclerite (L4B) straight, tapering into a needle-like process; the apical process (paa) broad, cylindrical and curved, the terminus a blunt and rounded end. The right dorsal phallomere (fda) of the first sclerite of right phallomere (R1) tapers to a rounded terminus, both margins sclerotized with a broad central membranous gap; the ventral plate (pia) strongly sclerotized and broad, with strong and numerous, curved grooves; the ventral process (pva) c-shaped, tapering to a point distally.

**Female.** (Fig. 26B) N=6: Body length 30.58–37.69 (33.14); forewing length 17.74–19.93 (18.91); hindwing length 15.02–15.97 (15.35); pronotum length 9.32–10.93 (9.86); prozone length 2.66–3.00 (2.78); pronotum width 2.94–3.40 (3.10); pronotum narrow width 2.08–2.24 (2.15); head width 5.90– 6.84 (6.22); head vertex to clypeus 2.57–2.91 (2.72); frons width 2.32–2.61 (2.42); frons height 0.86–0.97 (0.92); prothoracic femur length 8.37–9.90 (8.94); mesothoracic femur length 11.25–12.67 (11.80); mesothoracic tibia length 9.08–10.45 (9.65); mesothoracic tarsus length 7.59–8.47 (7.95); metathoracic femur length 11.44–13.44 (12.20); metathoracic tibia length 11.53–14.03 (12.91); metathoracic tarsus length 11.71–14.80 (13.13); pronotal elongation measure 0.28–0.29 (0.28); pronotal shape measure 0.30–0.33 (0.32); head shape measure 0.42–0.45 (0.44); frons shape measure 0.35–0.40 (0.38); anteroventral femoral spine count 14–16 (16); anteroventral tibial spine count 10; posteroventral tibial spine count 7.

*Head* (Fig. 44H): Juxta-ocular protuberances large, the apex in the medial third; vertex well above the dorsal margin of the eyes. Upper margin of clypeus barely convex, lower margin straight with medial bulge.

*Pronotum* (Fig. 49H): Prozone with lateral margins that are parallel, tapering anteriorly; the margins smooth. Metazone with concave lateral margins, a barely visible bulge in the posterior half; margins with small tubercles medially. Highly contrasting green and black coloration.

*Prothoracic Legs*: Femoral pit is black. Posterior prothoracic femoral genicular spine smaller than posteroventral spines, originating distal to the beginning of the genicular lobe. Prothoracic tibial posteroventral spines with the first (proximal) smallest and the fourth through sixth of similar length, the second and third are much longer.

*Meso- and Metathoracic Legs*: Femora with ventral (posterior) carina; dorsal (anterior) carina obvious.

*Wings*: Forewings colored symmetrically or asymmetrically, one being rust colored. The anal region of hindwings rusty basally and fading to a smoky grey and translucent medially before becoming black along the distal margin; the terminus of the discoidal region projecting beyond the distal margin of anal region.

*Abdomen*: Widening from first segment until the beginning of the distal half (segments 5–6) when the lateral margins narrow to the terminus, the middle being the broadest region. Tergites without posterolateral tergal projections. Supra-anal plate as long as wide, evenly rounded.

###### Etymology.

A noun in the genitive case, *Liturgusa krattorum* is named for Chris and Martin Kratt, hosts and creators of Kratts’ Creatures and Wild Kratts, among other television shows, which provide children with entertaining programming focused on animal biology presented with accurate information.

##### Liturgusa maroni sp. n.

http://zoobank.org/3406E298-A77C-4C78-A89B-039B6A80C540

http://species-id.net/wiki/Liturgusa_maroni

###### Type.

Holotype Female, pinned. Muséum national d’Histoire naturelle, Paris, France.

###### Type locality.

French Guiana: Guyane Franc, St-Laurent du Maroni, Collection Le Moult, Coll. L. Chopard, 1919, Octobre (Lat. 5.487038, Long. -54.008462).

###### Material examined.

*Liturgusa maroni* sp. n.

**Table:** 

Sex	Type	Country	Label	Latitude Longitude	Code
Female	Holotype	French Guiana	St-Laurent du Maroni, Collection Le Moult, Coll. L. Chopard, 1919, Octobre	5.487038, -54.008462	MNHN 019
Female	Paratype	French Guiana	Petit Saut, 8-II-1994, P.E. Rouland	5.069416, -53.047566	MNHN 061

###### Diagnosis.

A medium size species known only from female specimens from French Guiana. Most similar to *Liturgusa nubeculosa*, but much smaller and with blunt and shortened hindwings. The pronotum is moderately elongate and females have a broad abdomen. Forewings slightly shortened and obviously rounded, extending barely to the tip of the abdomen.

###### Description.

**Female.** (Fig. 7B) N=2: Body length 27.81–28.75 (28.28); forewing length 15.94–16.24 (16.09); hindwing length 12.69–12.78 (12.73); pronotum length 8.98–9.19 (9.09); prozone length 2.54–2.63 (2.58); pronotum width 3.00–3.04 (3.02); pronotum narrow width 2.01–2.21 (2.11); head width 6.18–6.29 (6.23); head vertex to clypeus 2.49–2.66 (2.58); frons width 2.27–2.30 (2.28); frons height 0.78; prothoracic femur length 8.49–8.88 (8.68); mesothoracic femur length 11.09–11.74 (11.41); mesothoracic tibia length 8.95–9.36 (9.15); mesothoracic tarsus length 7.78–8.05 (7.92); metathoracic femur length 11.25–11.77 (11.51); metathoracic tibia length 12.39–12.82 (12.60); metathoracic tarsus length 12.11–12.37 (12.24); pronotal elongation measure 0.28–0.29 (0.28); pronotal shape measure 0.33; head shape measure 0.40–0.43 (0.41); frons shape measure 0.34; anteroventral femoral spine count 16; anteroventral tibial spine count 10; posteroventral tibial spine count 7.

*Head* (Fig. 45B): Slightly transverse, juxta-ocular protuberances large, the apex in the middle third; the vertex between the parietal sutures is slightly concave; vertex above dorsal margin of the eyes. Frontal suture with a medial carina forming a continuous arc. Ocelli small and protruding on small cuticular mounds. Lateral ocelli oriented outward. Upper margin of clypeus barely convex, lower margin concave. Black markings surrounding frontal suture, the lower forming a point medially; lateral margins of frons with two, small black marks; the clypeus pale; the mandibles dark brown; the labrum mostly pale; the vertex and juxta-ocular protuberances splotched with dark brown marks; the area adjacent to lateral ocelli black. Palpi are pale.

*Pronotum* (Fig. 49I): Elongate with a moderately defined supra-coxal bulge; dorsal surface entirely smooth. Prozone with lateral margins that broaden slightly anterior to the supra-coxal sulcus before tapering anteriorly to a rounded terminus. Metazone with concave lateral margins, becoming nearly parallel in the posterior half with a slight bulge prior to narrowing towards the rounded posterior terminus; margins with small tubercles; posterior margin medially emarginate; the dorsal surface of the posterior half moderately depressed. The two posterior bulges on each side of the medial line are pronounced. Mostly brown with few black markings.

*Prothoracic Legs*: Femur elongate with a slightly concave dorsal margin; strongly defined pale to dark banding on posterior (external) surface; anterior (internal) surface with a black band running medially from the base to terminus; the ventral surface pale. Posterior surface of femur with few tubercles. A well developed femoral pit to accommodate terminal posteroventral tibial spine positioned medially to the proximal two posteroventral spines and in line with the distal most discoidal spine; pit is pale. Posterior prothoracic femoral genicular spine smaller than posteroventral spines, originating distal to the beginning of the genicular lobe. Prothoracic tibial posteroventral spines with the first (proximal) and fourth through sixth approximately the same size, the third being longer and the second very long, being nearly as long as the terminal spine. Prothoracic coxae smooth; the anterior surface with a broad, black band medially in the proximal half as well as a black spot medially towards the distal terminus.

*Meso- and Metathoracic Legs*: Femora with ventral (posterior) carina; dorsal (anterior) carina faint. Mesotarsi with first segment slightly shorter or the same length as the remaining segments combined.

*Wings*: Forewings mottled with brown, black, and pale coloration that is highly contrasting with large regions of lighter colored splotches; the costal region widened slightly with irregularly spaced black and pale banding; the terminus rounded and blunt, the overall shape appearing like a paddle, not extending to the tip of the abdomen. Forewings colored symmetrically. Hindwings smoky grey and translucent, the costal region more pale; the terminus of the discoidal region not projecting beyond the distal margin of anal region, the wing appearing truncate.

*Abdomen*: Broad, widening from first segment until the beginning of the distal half (segment 4) when the lateral margins narrow gradually to the terminus, the middle being the broadest region. Tergites in the posterior half with small posterolateral tergal projections. Supra-anal plate triangular with rounded margins and a rounded point.

###### Etymology.

A noun in apposition, *Liturgusa maroni* is named for the Maroni River near the type locality of Saint Laurent du Maroni on the border of French Guiana and Suriname.

##### Liturgusa nubeculosa

Gerstaecker, 1889

http://species-id.net/wiki/Liturgusa_nubeculosa

Liturgusa nubeculosa : Gerstaecker 1889: 54–56; Bertkau 1889: 87; Giglio-Tos 1927: 294; Beier 1935: 11; Terra 1995: 54; Jantsch 1999: 48; Lombardo and Agabiti 2001: 90, 97; Ehrmann 2002: 207; Otte and Spearman 2005: 133; Agudelo et al. 2007: 116, 142.
Liturgousa nubeculosa : Westwood 1889: 5, 51; Rehn 1935: 199; Rehn 1954: 177, pl. 1, fig. 2; Cerdá 1996: 76, Fig. 1.
Hagiomantis nubeculosa : Kirby 1904: 271.
Liturgusa peruviana : Giglio-Tos 1914: 77; Giglio-Tos 1927: 293; Beier 1935: 11; Rehn 1954: 177; Marshall 1975: 322; Terra 1995: 54; Ehrmann 2002: 207; Otte and Spearman 2005: 133. syn. n. (Rehn 1935)

###### Type.

Holotype Female. Ernst-Moritz-Arndt-Universität Greifswald, Germany.

###### Type locality.

Brazil: Ega, Fonteboa (Amazonas) (Lat. -2.522585, Long. -66.097224).

###### Material examined.

*Liturgusa nubeculosa* Gerstaecker, 1889.

**Table:** 

Sex	Type	Country	Label	Latitude Longitude	Code
Female	Holotype	Brazil	Ega, Fonteboa (Amazonas)	-2.522585, -66.097224	EMAU
Female	Holotype (peruviana)	Peru	Palcaru, 1904-65		BMNH
Female	nontype	Peru	Middle Rio Ucayali, II -27, F 6085, H. Bassler Collection Acc. 33591	-9.823201, -73.960930	AMNH 001
Female	nontype	Venezuela	Amazonas: Rio Mavaca Cp. 65°06'W, 2°2'N, 150m. III-16/27-1989, David Grimaldi, Exp. Phipps-Fudeci	2.033333, -65.100000	AMNH 025
Female	nontype	Ecuador	Pastaza: Cuisimi, on Rio Cuisimi 150km. SE Puyo, 350 m. June 1–5, 1971, B. Malkin	-2.404277, -77.040975	AMNH 026
Female	nontype	Brazil	Obidos, Para, Brazil, VIII 23 1919 (H.S. Parish)	-1.899285, -55.528371	ANSP 018
Female	nontype	Peru	Leonpampa 110 k. E. Huanaco, Huanaco Prov. Peru, Tropical Jungle, December 1937, Felix Woytkowski		ANSP 039
Male	nontype	Brazil	Manacapuru, S.M. Klages, March 1926	-3.286094, -60.640173	ANSP 106
Female	nontype	Brazil	Nova Oiinda, Rio Purus, S.M. Klages, May 1922, Carn. Mus. 6962		ANSP 107
Male	nontype	Brazil	Para		BMNH 004
Female	nontype	Peru	Yuras, 67 mi. E. of Tingo Maria. 350 m. XII-11-54. E.I. Schlinger & E.S. Ross collectors	-9.231743, -76.321433	CAS 016
Female	nontype	Peru	Monzon Valley, Tingo Maria, IX-23-1954, E.I. Schlinger & E.S. Ross collectors	-9.314153, -76.006745	CAS 018
Female	nontype	Bolivia	Dpto. Santa Cruz, Reserva Natural Potrerillo del Guenda, 17°40.281'S, 063°27.451'W, 400 m, 3-9.XI.2009, at MV.UV lights & gen. coll., Coll: G.J. Svenson	-17.671350, -63.457517	GSMC000269
Female	nontype	Bolivia	Dpto. Santa Cruz, Reserva Natural Potrerillo del Guenda, 17°40.281'S, 063°27.451'W, 400 m, 3–9.XI.2009, at MV.UV lights & gen. coll., Coll: G.J. Svenson	-17.671350, -63.457517	GSMC000270
Female	nontype	Bolivia	Dpto. Santa Cruz, Reserva Natural Potrerillo del Guenda, 17°40.281'S, 063°27.451'W, 400 m, 3–9.XI.2009, at MV.UV lights & gen. coll., Coll: G.J. Svenson	-17.671350, -63.457517	GSMC000273
Female	nontype	Bolivia	Dpto. Santa Cruz, Reserva Natural Potrerillo del Guenda, 17°40.281'S, 063°27.451'W, 400 m, 3-9.XI.2009, at MV.UV lights & gen. coll., Coll: G.J. Svenson	-17.671350, -63.457517	GSMC000308
Female	nontype	Peru	Loreto, Pacaya Samiria, Cocha Shinguito; TLE & MGP; TSnl Insecticidal fog of Fog Tree 2		GSMC003072
Male	nontype	Peru	Loreto Province, Madre Selva Biological Research Station, -3.62096, -72.24744, 10-17 February 2013, Coll: G.J. Svenson	-3.620960, -72.247440	GSMC004006
Male	nontype	Peru	Loreto Province, Madre Selva Biological Research Station - Tissue 024	-3.620960, -72.247440	GSMC004006
Female	nontype	Peru	Loreto Province, Puerto Almendra, -3.830525, -73.374, 108 m, 19-21 February 2013, Coll: G.J. Svenson, Tissue 032	-3.830525, -73.374000	GSMC004010
Female	nontype	Peru	Loreto Province, Puerto Almendra - Tissue 035	-3.830525, -73.374000	GSMC004010
Male	nontype	Peru	Loreto Province, Puerto Almendra, -3.830525, -73.374, 108 m, 19-21 February 2013, Coll: G.J. Svenson, Tissue 032	-3.830525, -73.374000	GSMC004012
Male	nontype	Peru	Loreto Province, Puerto Almendra - Tissue 044	-3.830525, -73.374000	GSMC004012
Male	nontype	Peru	Loreto Province, Puerto Almendra, -3.830525, -73.374, 108 m, 19-21 February 2013, Coll: G.J. Svenson, Tissue 032	-3.830525, -73.374000	GSMC004013
Male	nontype	Peru	Loreto Province, Puerto Almendra	-3.830525, -73.374000	GSMC004013
Male	nontype	Peru	Loreto Province, Puerto Almendra, -3.830525, -73.374, 108 m, 19-21 February 2013, Coll: G.J. Svenson, Tissue 032	-3.830525, -73.374000	GSMC004014
Male	nontype	Peru	Loreto Province, Puerto Almendra - Tissue 043	-3.830525, -73.374000	GSMC004014
Female	nontype	Peru	Loretro Province, Madre Selva Biological Research Station, -3.62096, -72.24744, 10-17 February 2013, Coll: G.J. Svenson	-3.620960, -72.247440	GSMC004022
Female	nontype	Peru	Loreto Province, Madre Selva Biological Research Station - Tissue 021	-3.620960, -72.247440	GSMC004022
Nymph	nontype	Peru	Loreto Province, Puerto Almendra, -3.830525, -73.374, 108 m, 19-21 February 2013, Coll: G.J. Svenson, Tissue 032	-3.830525, -73.374000	GSMC004030
Female	nontype	Peru	Loreto Province, Puerto Almendra, -3.830525, -73.374, 108 m, 19-21 February 2013, Coll: G.J. Svenson, Tissue 032	-3.830525, -73.374000	GSMC004031
Female	nontype	Peru	Loreto Province, Puerto Almendra - Tissue 039	-3.830525, -73.374000	GSMC004031
Nymph	nontype	Peru	Loreto Province, Puerto Almendra, -3.830525, -73.374, 108 m, 19-21 February 2013, Coll: G.J. Svenson, Tissue 032	-3.830525, -73.374000	GSMC004040
Nymph	nontype	Peru	Loreto Province, Puerto Almendra, -3.830525, -73.374, 108 m, 19-21 February 2013, Coll: G.J. Svenson, Tissue 032	-3.830525, -73.374000	GSMC004045
Male	nontype	Peru	Madre de Dios, Rio Tambopata Res. 30km (air) sw Pto. Maldonato, 290m, 12°50'S, 069°20'W, Smithsonian Institution Canopy Fogging Project, T.L. Erwin et al. colls. 30 Apr84, 03/O&S (01), Fogging 00017348	-12.833333, -69.333333	GSMC004055
Male	nontype	Colombia	Dept. Amazonas, Rio Igara Parana, 30 km aval La Chorrera, VI-VIII 1974, M. Descamps rec.	-1.197385, -72.937475	MNHN 009
Female	nontype	Ecuador	Limoncocha, 25-VII-1983, K. Riede rec.	-0.400848, -76.618481	MNHN 056
Female	nontype	Peru	Dept. Loreto Colonia, Amont Conflt. Rios Zumun & Yahuasyacu, 20-V-20-VI-1978, M. Descamps rec.		MNHN 057
Female	nontype	Brazil	Benjamin Constant, IX 1979, AM, B. Silva rec., C. Seabia leg.	-4.383010, -70.042251	MNHN 058
Female	nontype	Peru	Loreto, Maynas, Picuroyacu, apres Sta Clotilde, N. Iquitos, 130 mts., 10.II.2010, S03.37.04 - W 73.15.44, M. Dottax leg.	-3.617778, -73.262222	MNHN 089
Female	nontype	Peru	Loreto, Picuroyacu, Jan 2010, Coll: J.J. Ramirez	-3.617778, -73.262222	MNHN 094
Male	nontype	Peru	Loreto, Maynas, Picuroyacu, apres Sta Clotilde, N. Iquitos, 130 mts., 10.II.2010, S03.37.04 - W 73.15.44, M. Dottax leg.	-3.617778, -73.262222	MNHN 098
Male	nontype	Peru	Loreto, Maynas, Picuroyacu, apres Sta Clotilde, N. Iquitos, 130 mts., 10.II.2010, S03.37.04 - W 73.15.44, M. Dottax leg.	-3.617778, -73.262222	MNHN 099
Female	nontype	Ecuador	Pr. Napo, Rio Tiputini, Pindo, Mandaripanga, 76°44'W, 0°43'S, 30 IX / 15 × 1997, Amedegnato / Poulain rec.	-0.716667, -76.733333	MNHN 204
Female	nontype	Ecuador	Pr. Napo, Rio Tiputini, Pindo, Mandaripanga, 76°44'W, 0°43'S, 30 IX / 15 × 1997, Amedegnato / Poulain rec.	-0.716667, -76.733333	MNHN 205
Female	nontype	Bolivia	Dpto. Santa Cruz, Prov. Andres Ibanez, Loc. Espejillos, 30-VIII-1990, col. Ma. Estker Moutano	-17.718841, -63.438001	MNKM 001
Female	nontype	Peru	Loreto, Iquitos, Nov. 2010, Collection Stiewe	-3.741872, -73.272190	MSMC 001
Female	nontype	Peru	Chamicuros, Bartlett		OUMNH 007
Female	nontype	Brazil	Para		OUMNH 017
Male	nontype	Brazil	Amazonas, Rio Janauaca, 40 km SW Manaus, 10 Mar 1979, 03°20'S, 060°17'W, White water innundation forest canopy fogged with Pyrethrin Sample #57, Montgomery, Erwin, Schimmel, Krischik, Date, Bacon colls.	-3.333333, -60.283333	USNM 005; USNM ENT 00873027
Male	nontype	Brazil	Amazonas, Rio Janauaca, 40 km SW Manaus, 10 Mar 1979, 03°20'S, 060°17'W, White water innundation forest canopy fogged with Pyrethrin Sample #52, Montgomery, Erwin, Schimmel, Krischik, Date, Bacon colls.	-3.333333, -60.283333	USNM 009; USNM ENT 00873026
Female	nontype	Ecuador	N.P., Sant Cecilia, III-25-31-1969, P. & P. Spangler		USNM 031; USNM ENT 00873028
Female	nontype	Colombia	Amazonas, Rio Cotuhe, St. Lucia + Tarapaca, 4 Feb. Megers	-2.889475, -69.745012	USNM 032; USNM ENT 00873029
Male	nontype	Ecuador	Road between El Puyo & Puerto Napo, VII-1964, Dr. Ch. Gregoire	-1.229112, -77.879766	USNM 068; USNM ENT 00873030
Female	nontype	Peru	Nord-Peru, Mishuyacu (Maranon-Gebiet)	-5.910456, -76.103566	ZMHB 006
Female	nontype	Ecuador	Canálos, S.V. Feyer	-1.589524, -77.746701	ZMHB 008
Female	nontype	Brazil	Obidos, Amazonas, I. Michaelis leg., vend. 13.IX 1900	-1.899285, -55.528371	ZMUH 001
Male	nontype	Ecuador	Pastaza; 300m. Rio Macuma, 10km from Rio Marona, VII:5-7:1971, leg. B. Malkin	-2.753512, -77.444899	FMNH 003
Male	nontype	Ecuador	Pastaza; Cusuimi, Rio Cusuimi, 150km SE of Puyo, V:15-31:1971, leg. B. Malkin	-2.391736, -77.047683	FMNH 006

###### Taxonomic history.

The species was described early relative to others in the genus, but was only included in taxon lists without receiving revisionary attention. In 1904 Kirby moved the species to *Hagiomantis*, but this action was ignored by other taxonomists since Giglio-Tos included the species within *Liturgusa* along with all subsequent works.

In 1954, James Rehn synonymized *Liturgusa peruviana* Giglio-Tos, 1914, with its senior synonym, *Liturgusa nubeculosa* Gerstaecker, 1889, but his action appears to have been overlooked. Here again these two species are synonymized, but credit for first recognizing this synonymy goes to Rehn.

###### Natural history.

*Liturgusa nubeculosa* is one of the largest species of Neotropical bark mantis. Males and females are extremely difficult to catch during the day and have been primarily found on large diameter, smooth bark trees. Once they spot a threat, they run rapidly up the tree and out of reach, often circling to the back side, which is typical of the genus. Males were collected far more often than females on a recent trip to the Loreto province in northern Peru, but the opposite was true at a location in Bolivia (see material examined). It is not clear whether there are distinct sex ratios in certain locations or this sex bias was coincidence of habitat utilization.

###### Diagnosis.

Nearly identical to *Liturgusa cameroni*, the primary distinguishing characteristics being male genital features, distribution and the hindwing. It is one of the largest *Liturgusa* species with robust legs, highly contrasting mottled forewings and strong banding. A feature easily seen on most specimens that is capable of distinguishing the species from *Liturgusa cameroni* is that the discoidal region of the hindwing extends beyond the distal margin of the anal region, which gives the wing a slightly more elongate appearance compared to the hindwing of *Liturgusa cameroni*.

###### Description.

**Figure 27.**
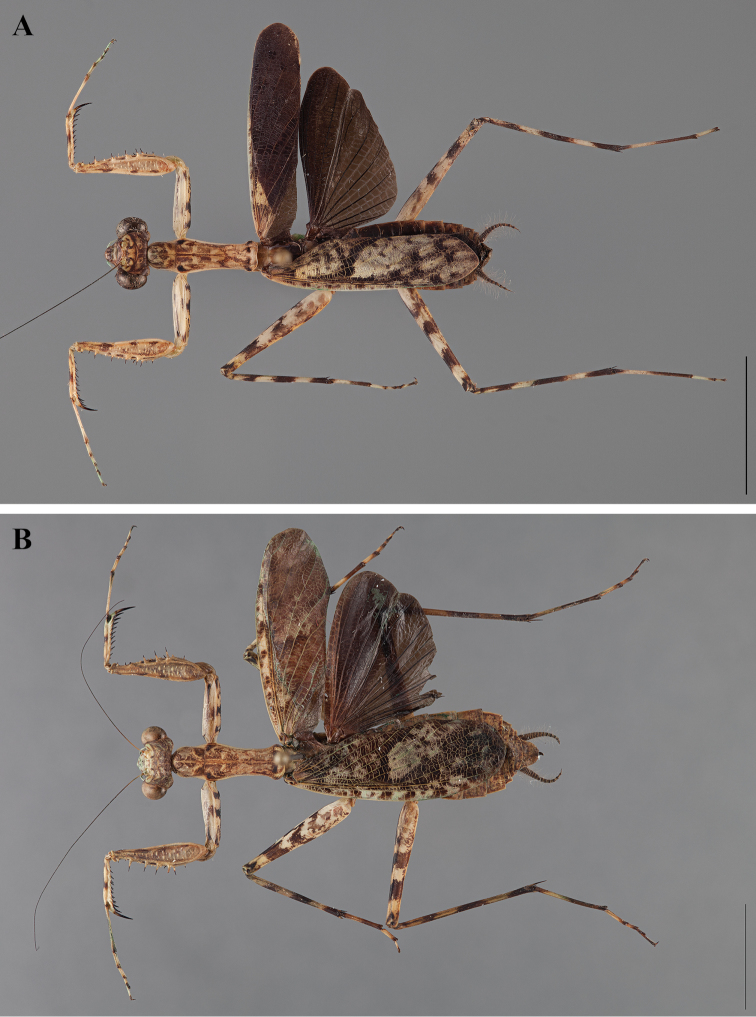
*Liturgusa nubeculosa* Gerstaecker, 1889, dorsal habitus: **A** male from Loreto, Peru (CLEV GSMC004012) **B** female from Santa Cruz, Bolivia (CLEV GSMC000263).

**Male.** (Fig. 27A) N=11: Body length 24.64–27.05 (25.62); forewing length 15.12–16.26 (15.73); hindwing length 11.94–12.67 (12.37); pronotum length 7.39–8.84 (8.06); prozone length 2.03–2.34 (2.19); pronotum width 2.42–2.68 (2.56); pronotum narrow width 1.74–2.06 (1.86); head width 4.79–5.47 (5.27); head vertex to clypeus 1.90–2.15 (2.07); frons width 1.77–2.02 (1.91); frons height 0.61–0.79 (0.70); prothoracic femur length 6.63–7.51 (7.14); mesothoracic femur length 8.93–10.67 (9.70); mesothoracic tibia length 7.03–8.34 (7.59); mesothoracic tarsus length 6.30–7.43 (6.64); metathoracic femur length 9.01–10.30 (9.69); metathoracic tibia length 9.60–10.89 (10.34); metathoracic tarsus length 9.06–10.84 (9.80); pronotal elongation measure 0.26–0.28 (0.27); pronotal shape measure 0.30–0.33 (0.32); head shape measure 0.38–0.41 (0.39); frons shape measure 0.34–0.40 (0.37); anteroventral femoral spine count 15–16 (16); anteroventral tibial spine count 10; posteroventral tibial spine count 7.

*Head* (Fig. 45C): Transverse, juxta-ocular protuberances small, the apex in the lateral third; the vertex between the parietal sutures is straight; vertex even or slightly below the dorsal margin of the eyes. Frontal suture with a medial carina forming a continuous arc. Ocelli small and protruding on small cuticular mounds. Lateral ocelli oriented outward. The frons transverse; lower region entirely black. Clypeus transverse; upper margin convex, lower margin concave. Antennae pale basally, fading to black within the proximal ten percent. A moderate black band that fades medially extending across the frontal suture from eye to eye; clypeus mostly pale with brown markings in the upper lateral corners; mandibles pale medially and brown laterally; the vertex and juxta-ocular protuberances speckled with dense black markings; the area adjacent to lateral ocelli black. Palpi are pale.

*Pronotum* (Fig. 49K): Elongate, but robust with a slightly defined supra-coxal bulge; dorsal surface with numerous small tubercles. Prozone elongate with slightly widening lateral margins before tapering anteriorly; the margins smooth. Metazone with concave lateral margins, a slight bulge in the posterior half; margins with small, blunt tubercles; posterior margin slightly emarginate medially; the anterior half of dorsal surface raised; the dorsal surface of the posterior half depressed. Mostly brown with black marks.

*Prothoracic Legs*: Femur elongate, but robust (thick) with a slightly concave dorsal margin; strongly defined pale to dark banding on posterior (external) surface; anterior (internal) surface with a black band running medially from the base to terminus; the ventral surface pale. Posterior surface of femur with numerous tubercles. A well developed femoral pit to accommodate terminal posteroventral tibial spine positioned medially to the proximal two posteroventral spines and in line with the distal most discoidal spine; pit is dark. Posterior prothoracic femoral genicular spine much smaller than posteroventral spines, originating distal to the beginning of the genicular lobe. Prothoracic tibial posteroventral spines with the first (proximal) smallest and the third through sixth of similar length, the second longer. Prothoracic coxae smooth; the anterior surface with a small, black band medially in the proximal half as well as a small black spot medially towards the distal terminus.

*Meso- and Metathoracic Legs*: Femora with strongly pronounced ventral (posterior) and dorsal (anterior) carina. Mesotarsi with first segment shorter than the remaining segments combined.

*Wings*: Forewings mottled with black, pale, brown and grey coloration that is highly contrasting, exhibiting large grey regions surrounded by black markings; the costal region widened with pale and dark irregular banding. Forewings colored asymmetrically, one being mottled as described the other is darkened significantly with a black or rust tone, the mottled pattern still visible. Hindwings smoky black, mostly translucent; the discoidal region with black, opaque pigmentation basally and in the anterior two thirds extending from the base to the distal terminus, continuing into the costal region; the terminus of the discoidal region projecting beyond the distal margin of anal region, giving the wing an elongate appearance.

*Abdomen*: Elongate, tubular with slight widening before the posterior narrowing; smooth and black coloration dorsally. Tergites without posterolateral projections. Supra-anal plate transverse, half as long as wide, tapering to a rounded terminus. Subgenital plate irregularly rounded and without styli.

*Genital Complex* (Fig. 52J.1–J.2): The main body of ventral left sclerite (L4A) with a short, triangular distal process (pda) positioned lateral to the central axis, the outer margin straight, heavily sclerotized and serrated from the terminus of the pda extending proximally one quarter the length of L4A. The apofisis falloid (afa) of the main body of dorsal left sclerite (L4B) large and broad, but short, forming a sharply pointed process with a convex medial margin (closest to the paa) leading to the terminus and a concave outer margin (opposite the paa), the entire structure resembling a broad sickle; the apical process (paa) broad basally, quickly narrowing to a heavily sclerotized, rounded terminus. The right dorsal phallomere (fda) of the first sclerite of right phallomere (R1) tapers to a broadly rounded, membranous terminus, the outer margin sclerotized; the ventral plate (pia) strongly sclerotized, broad proximally with strong curved grooves; the ventral process (pva) enlarged and c-shaped.

###### Redescription.

**Figure 28.**
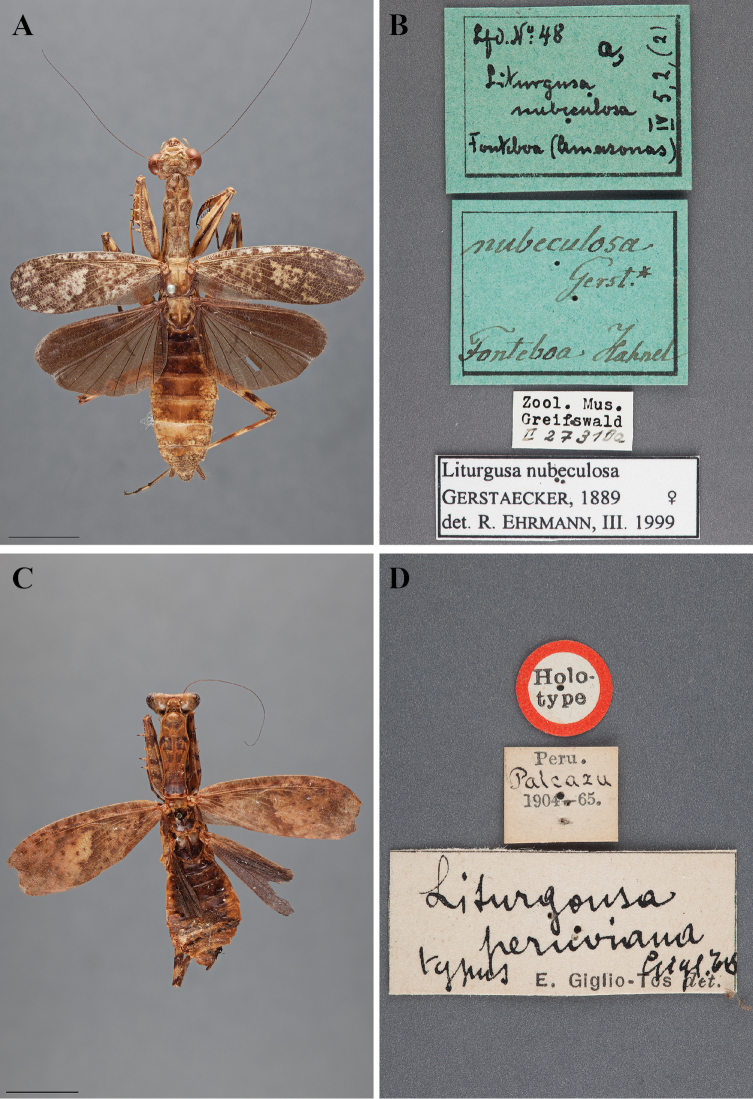
*Liturgusa nubeculosa* Gerstaecker, 1889, and *Liturgusa peruviana* Giglio-Tos, 1914 syn. n., dorsal habitus and labels. *Liturgusa nubeculosa*: **A** holotype female (EMAU) **B** labels. *Liturgusa peruviana*: **C** holotype female (BMNH) **D** labels.

**Female.** (Figs 1C, 27B, 28A, 28C) N=23: Body length 32.17–52.03 (37.48); forewing length 19.32–26.96 (21.76); hindwing length 15.98–19.15 (17.44); pronotum length 9.77–13.12 (10.85); prozone length 2.76–3.69 (3.04); pronotum width 3.16–4.49 (3.62); pronotum narrow width 2.09–3.07 (2.54); head width 6.37–7.91 (6.86); head vertex to clypeus 2.58–3.38 (2.90); frons width 2.46–3.23 (2.70); frons height 0.89–1.23 (1.00); prothoracic femur length 8.54–12.24 (9.88); mesothoracic femur length 10.74–14.62 (12.30); mesothoracic tibia length 8.84–12.35 (9.94); mesothoracic tarsus length 7.71–10.74 (8.66); metathoracic femur length 10.88–14.87 (12.26); metathoracic tibia length 12.22–17.08 (13.55); metathoracic tarsus length 10.68–13.38 (12.34); pronotal elongation measure 0.27–0.29 (0.28); pronotal shape measure 0.32–0.35 (0.33); head shape measure 0.40–0.44 (0.42); frons shape measure 0.34–0.42 (0.37); anteroventral femoral spine count 15–17 (16); anteroventral tibial spine count 10; posteroventral tibial spine count 7 (one female with 8 on left tibia).

*Head* (Fig. 45D): As broad as long, juxta-ocular protuberances very large, the apex in the middle third; the vertex between the parietal sutures is slightly concave; vertex well above the dorsal margin of the eyes. Frontal suture with a medial carina forming a continuous arc, more angular medially than in male. Ocelli small and protruding on a contiguous carina connecting all three ocelli; lateral ocelli oriented outward, the central ocelli almost vertical. The frons transverse, lower region below the carina black. Clypeus slightly transverse. Frontal suture without a defined black band as described in males; clypeus pale; mandibles pale; the vertex and juxta-ocular protuberances speckled with dense black markings; the area adjacent to lateral ocelli black. Palpi are pale.

*Pronotum* (Fig. 49L): Prozone elongate with near parallel lateral margins before tapering anteriorly; the margins with few small tubercles.

*Prothoracic Legs*: Prothoracic tibial posteroventral spines with the first (proximal) smallest and the third through sixth of similar length (the third slightly longer than the other three), the second much longer. Posterior prothoracic femoral genicular spine tiny compared to posteroventral spines, originating distal to the beginning of the genicular lobe. Prothoracic coxae smooth; the anterior surface with a small, black band medially in the proximal half, but no black mark in near the distal terminus.

*Meso- and Metathoracic Legs*: As described for males.

*Wings*: Forewings colored symmetrically. The terminus of the discoidal region of the hindwing projecting slightly beyond the distal margin of anal region, giving the wing a moderately elongate appearance.

*Abdomen*: Broad, widening from first segment until the beginning of the distal half (segment 4) when the lateral margins narrow gradually to the terminus, the middle being the broadest region. Tergites with or without small posterolateral tergal projections in the posterior half of the abdomen. Supra-anal plate slightly transverse, rounded.

##### Liturgusa purus sp. n.

http://zoobank.org/01D25B69-2391-4AFA-81CF-FCD28AC0F274

http://species-id.net/wiki/Liturgusa_purus

###### Type.

Holotype Male, pinned. Academy of Natural Sciences of Drexel University, Philadelphia, PA, USA.

###### Type locality.

Brazil, Hyutanahan, Rio Purus, March 1922, S.M. Klages (Lat. -5.602502, Long. -63.221263).

###### Material examined.

*Liturgusa purus* sp. n.

**Table:** 

Sex	Type	Country	Label	Latitude Longitude	Code
Male	Holotype	Brazil	Hyutanahan, Rio Purus, Klages, S.M., March 1922	-5.602502, -63.221263	ANSP 101
Female	Allotype	Brazil	Hyutanahan, Rio Purus, Klages, S.M., March 1922	-5.602502, -63.221263	ANSP 103

###### Diagnosis.

Brown and light green with a slender appearance. Forewings asymmetrically colored and hindwings with the discoidal region mostly orange or rust colored. Most similar to *Liturgusa krattorum* and *Liturgusa algorei*, but with a paler head and a less contrasting banding pattern. The prothoracic femora are more brown across the posterior surface compared to the other two species. In addition, *Liturgusa purus* has a posterior prothoracic femoral genicular spine that originates well proximal to the beginning of the genicular lobe, which is unique among *Liturgusa krattorum*, *Liturgusa algorei*, and other Cursor Group species.

###### Description.

**Figure 29.**
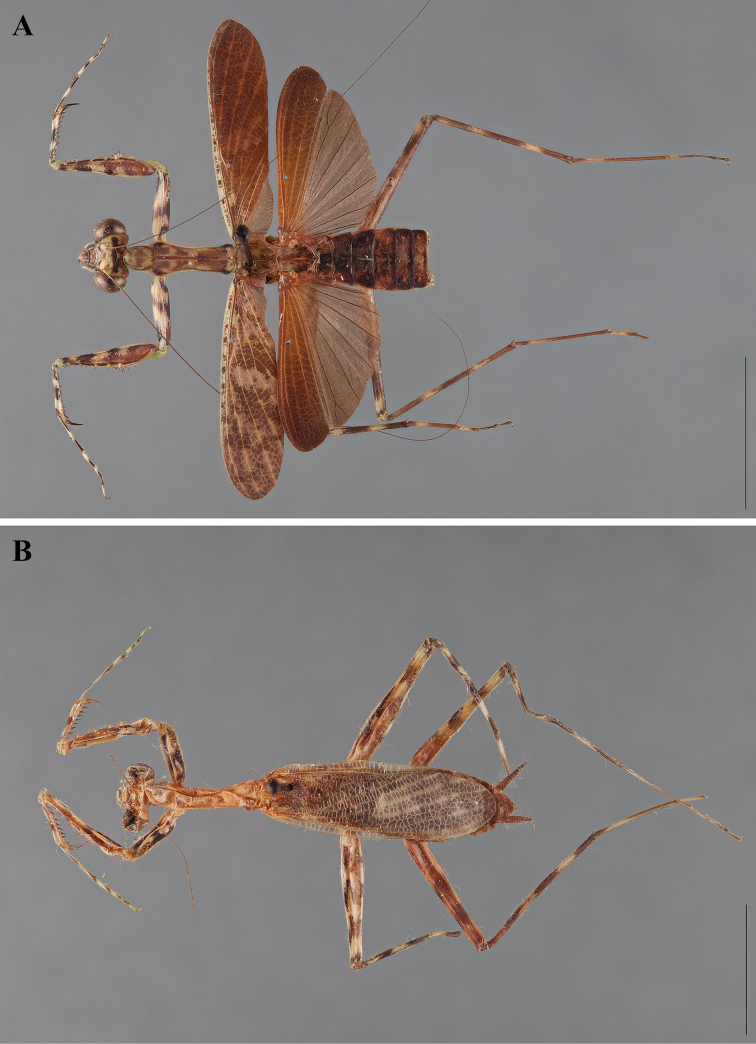
*Liturgusa purus* sp. n., dorsal habitus: **A** holotype male from Rio Purus, Brazil (ANSP 101) **B** allotype female from Rio Purus (ANSP 103).

**Male.** (Fig. 29A) N=2: Body length 29.16; forewing length 15.12–17.65 (16.39); hindwing length 11.86; pronotum length 7.42; prozone length 2.00; pronotum width 2.36; pronotum narrow width 1.61; head width 4.90; head vertex to clypeus 2.03; frons width 1.71; frons height 0.68; prothoracic femur length 6.64; mesothoracic femur length 9.40–11.15 (10.28); mesothoracic tibia length 7.44–8.83 (8.14); mesothoracic tarsus length 6.89; metathoracic femur length 9.67–11.48 (10.58); metathoracic tibia length 9.99–12.00 (10.99); metathoracic tarsus length 10.48–11.13 (10.80); pronotal elongation measure 0.27; pronotal shape measure 0.32; head shape measure 0.41; frons shape measure 0.40; anteroventral femoral spine count 15–16 (16); anteroventral tibial spine count 10; posteroventral tibial spine count 7.

*Head* (Fig. 45A): Transverse, juxta-ocular protuberances large, the apex in the lateral third; the vertex between the parietal sutures is straight; vertex even with the dorsal margin of the eyes. Frontal suture with a medial carina forming a continuous arc. Ocelli small and protruding on a small cuticular mound, but almost flat. Lateral ocelli oriented outward, a few degrees off perpendicular. Upper margin of clypeus convex, lower margin straight. Antennae pale basally fading quickly to brown. Broad black band extending straight over the medial carina of the frontal suture; lower region of frons dark brown with two pale lateral spots; vertex and juxta-ocular protuberances mostly brown; the area around ocelli pale, the lower region of clypeus, the mandibles, and the labrum are mostly brown. Palpi are pale.

*Pronotum* (Fig. 49J): Elongate with a moderately defined supra-coxal bulge; dorsal surface entirely smooth. Prozone with lateral margins that are near parallel, tapering anteriorly; the margins smooth. Metazone with concave lateral margins; margins with numerous small tubercles; posterior margin medially emarginate; the dorsal surface of the posterior half slightly depressed.

*Prothoracic Legs*: Femur elongate with a nearly straight dorsal margin; less defined pale to dark banding on posterior (external) surface, more brown on the ventral margin with strongly brown regions between posteroventral spines; anterior (internal) surface with a black band running medially from the base to terminus; the ventral surface pale. Posterior surface of femur with few tubercles. A well developed femoral pit to accommodate terminal posteroventral tibial spine positioned medially to the proximal two posteroventral spines and in line with the distal most discoidal spine; pit is pale. Posterior prothoracic femoral genicular spine half the length as the posteroventral spines, originating well proximal to the beginning of the genicular lobe. Prothoracic tibial posteroventral spines with the first (proximal) smallest and the second through sixth of similar length, the second and third are slightly longer. Prothoracic coxae smooth; the anterior surface with a broad, diagonal black band in the proximal half.

*Meso- and Metathoracic Legs*: Femora with faint ventral (posterior) carina; dorsal (anterior) carina faint. Mesotarsi with first segment equal to remaining segments combined.

*Wings*: Forewings mottled with brown, green, and pale coloration; the costal region with light to dark irregular banding. Forewings colored asymmetrically, one being mottled, the other is rust colored with the mottled pattern still visible. Hindwings with a red or rusty color in the discoidal region, darkening distally; the anal region a smoky grey and translucent; the terminus of the discoidal region projecting well beyond the distal margin of anal region, the wings appearing elongate.

*Abdomen*: Elongate, tubular with slight widening before posterior narrowing; smooth, reddish dorsal surface. Supra-anal plate transverse, tapering to a broadly rounded terminus. Subgenital plate irregularly rounded and without styli.

*Genital Complex* (Fig. 52K.1): The main body of ventral left sclerite (L4A) with a bulging distal process (pda) positioned laterally (45 degrees from central axis) and projecting like a blunt knob. The apofisis falloid (afa) of the main body of dorsal left sclerite (L4B) broadly tapering to a short, sharp point that is well sclerotized and oriented approximately 25 degrees from the central axis; the apical process (paa) broad, cylindrical and curved, the terminus a tapered and rounded end. The right dorsal phallomere (fda) of the first sclerite of right phallomere (R1) tapers to a rounded terminus; the ventral plate (pia) strongly sclerotized, slightly widened proximally with curved grooves; the ventral process (pva) c-shaped, tapering to a point.

**Female.** (Fig. 29B) A single specimen is known, but has degraded due to poor preservation. Measurements are impossible, but some aspects of the description are possible and those included are presented in full as to not confuse character states that may or may not match with males.

*Head*: Frontal suture with a medial carina forming a continuous arc. Ocelli small and almost flat on the surface. Upper margin of clypeus convex, lower margin straight. Antennae pale basally fading quickly to brown. Palpi are pale.

*Pronotum*: Elongate with a moderately defined supra-coxal bulge. Metazone with concave lateral margins; margins with numerous small tubercles; posterior margin medially emarginate.

*Prothoracic Legs*: Femur with less defined pale to dark banding on posterior (external) surface, more brown on the ventral margin with strongly brown regions between posteroventral spines; anterior (internal) surface with a black band running medially from the base to terminus, a broad mark medially; the ventral surface pale. A well developed femoral pit to accommodate terminal posteroventral tibial spine positioned medially to the proximal two posteroventral spines and in line with the distal most discoidal spine; pit is pale. Prothoracic tibial posteroventral spines with the first (proximal) smallest and the second through sixth of similar length, the second and third are slightly longer. Prothoracic coxae smooth; the anterior surface with a broad, diagonal black band in the proximal half.

*Meso- and Metathoracic Legs*: Mesotarsi absent, metatarsi with first segment much longer than remaining segments combined.

*Wings*: Forewings mottled with brown, green, and pale coloration.

*Abdomen*: Widening from first segment until the beginning of the distal half (segments 5–6) when the lateral margins narrow to the terminus, the middle being the broadest region. Tergites with slight posterolateral projections in the distal half of the abdomen. Supra-anal plate slightly transverse, tapering to a rounded, emarginate terminus.

###### Etymology.

A noun in apposition, *Liturgusa purus* is named for Rio Purus, Brazil.

#### Fuga gen. n.

http://zoobank.org/43C4414B-EAF2-473B-BD34-8DAB040D3699

http://species-id.net/wiki/Fuga

Mantis (*partim*): Audinet Serville 1838: 199.
Oxypilus (*partim*): De Haan 1842: 84.
Liturgousa (*partim*): Saussure 1869: 62; Brauer 1870: 92; Saussure 1871b: 102 (♀ only; *partim*); Westwood 1889: 4, 49, pl. 2, fig. 3; Kirby 1904: 271; Werner 1906: 372; Chopard 1911: 323; Chopard 1916: 164; Rehn 1935: 199, pl. 8, fig. 4; Hughes-Schrader 1943: 266, 280, Table 1, Figs 19–28; Hughes-Schrader 1948: 267; Hughes-Schrader 1950: 11, Table 1; Cerdá 1996: 75.
Liturgusa (*partim*): Hebard 1922: 337; Giglio-Tos 1927: 294; Beier 1935: 11; Terra 1995: 53, Figs 85–87; Jantsch 1999: 48; Ehrmann 2002: 206; Otte and Spearman 2005: 132; Agudelo et al. 2007: 116, 141.
Hagiomantis (*partim*): Piza 1965: 130; Piza 1966: 8; Terra 1995: 54; Ehrmann 2002: 163–164; Otte and Spearman 2005: 129–130.

##### Type species.

*Mantis annulipes* Audinet Serville, 1838

##### Description.

*Body*: The overall coloration of all *Fuga* species varies with a mottled or camouflage pattern that incorporates black, brown, pale tan, white or grey, and sometimes shades of green. The mottled patterns can be diffuse or highly contrasting with whitish regions abutting black spots or splotches. All species are dorsoventrally flattened with disproportionately long legs in comparison to body length.

*Measurement ranges*: **Male.** Body length 18.84–22.11; forewing length 12.76–16.47; hindwing length 10.23–11.89; pronotum length 4.75–6.53; prozone length 1.54–2.12; pronotum width 2.02–2.42; pronotum narrow width 1.66–1.87; head width 4.33–4.88; head vertex to clypeus 1.59–1.86; frons width 1.49–1.63; frons height 0.52–0.65; prothoracic femur length 5.09–6.39; mesothoracic femur length 6.58–8.67; mesothoracic tibia length 5.20–7.00; mesothoracic tarsus length 4.79–6.03; metathoracic femur length 6.33–7.39; metathoracic tibia length 6.86–8.35; metathoracic tarsus length 6.98–9.11; pronotal elongation measure 0.32–0.33; pronotal shape measure 0.37–0.44; head shape measure 0.34–0.38; frons shape measure 0.34–0.40. **Female.** Body length 21.70–29.89; forewing length 14.89–20.82; hindwing length 12.08–16.07; pronotum length 5.75–7.85; prozone length 1.93–2.62; pronotum width 2.55–3.04; pronotum narrow width 1.98–2.34; head width 5.30–6.14; head vertex to clypeus 2.08–2.39; frons width 1.89–2.32; frons height 0.67–0.88; prothoracic femur length 6.26–8.17; mesothoracic femur length 7.22–9.92; mesothoracic tibia length 5.83–8.28; mesothoracic tarsus length 5.26–7.53; metathoracic femur length 7.24–10.01; metathoracic tibia length 8.07–11.50; metathoracic tarsus length 7.81–11.55; pronotal elongation measure 0.32–0.34; pronotal shape measure 0.36–0.49; head shape measure 0.37–0.41; frons shape measure 0.33–0.40.

*Head*: Transverse with large, rounded eyes projecting outside the profile of the head both laterally and anteriorly (the anterior margin of the eyes anterior to the central surface of the head). Juxta-ocular protuberances present to varying degrees within males, but always well developed in females. The vertex between the parietal sutures is either straight or concave. Frontal suture with a faint medial carina. Ocelli small in males protruding slightly on a cuticular mound; reduced in females and laying more flat on the surface. Central ocellus oriented anteriorly and lateral ocelli oriented outward, perpendicular to the central axis of the head or at most a few degrees off perpendicular. Frons narrowed between the antennal insertion sites and depressed below the central ocellus; a transverse carina present below the central ocellus, running from lateral margins under the antennal insertion sites medially in a dorsally oriented curve. Upper margin of clypeus convex, lower margin straight; a transverse ridge medially; lateral margins tapering, widest at the upper margin. Labrum with minimal sculpting and a rounded terminus. Antennae filiform and with rare setae, pale or dark or a combination of both, never banded. Varying levels of black markings across the anterior surface of head that can include a transverse band or spots on the lower part of the frons, markings around the ocelli and the vertex, and markings on the clypeus, labrum and mandibles. Palpi are pale or with a darkened terminus.

*Pronotum*: Slightly elongate (*pronotum shape measure* 0.36) to squat (*pronotum shape measure* 0.49) with a moderately defined supra-coxal bulge; dorsal surface smooth or at most with dispersed, fine tubercles. Prozone with lateral margins that are parallel before tapering anteriorly. Metazone with concave lateral margins, always with a middle region that is narrower than the supra-coxal bulge and the posterior end. Coloration highly variable with pale and black markings. Supra-coxal sulcus strongly defined; posterior margin straight or barely medially emarginate.

*Prothoracic legs*: Femoral spine count of male and female: anteroventral 14–17, posteroventral 4, discoidal 4. Femur robust with a straight or slightly concave dorsal margin; anteroventral and posteroventral (internal and external, respectively) spines well developed; line of small tubercles running medially of the posteroventral spines. A continuous carina running from distal terminus of femur along dorsal margin to the base, circling the posterior surface of the proximal end and running along the ventral margin at the base of the posteroventral spines. Pale to dark banding on posterior (external) surface of femur; anterior (internal) surface entirely black or pale with varying patterns of black markings. Posterior (external) surface of femur smooth or with few tubercles. Well developed femoral pit on the ventral surface to accommodate terminal posteroventral tibial spine positioned between the most proximal posteroventral spine and the most distal discoidal spine; pit is colored black or pale. Prothoracic tibial spine count of male and female: anteroventral 9–10, posteroventral 8. Prothoracic tibial spines robust; the posteroventral spines with the first and second most proximal and fifth through seventh shorter than the longer proximal third, fourth, and terminal spines; the anteroventral spines longest at distal end and shortening proximally, but the sixth and seventh spines from the distal terminal spine longer than adjacent spines. Tarsi banded with pale and dark coloration. Prothoracic coxae smooth with no or a few very minor tubercles or setae along dorsal margin; black markings vary across species.

*Meso- and metathoracic legs*: Long and slender with pale to dark banding on the femur and tibia; posterior (upper) surface of femora smooth. Femora with ventral (posterior) carina, some species being more pronounced than others; dorsal (anterior) carina less pronounced, but visible. Tibia long and rounded with well developed terminal spurs. Mesotarsi with first segment as long or shorted than the remaining segments combined. Metatarsi with first segment equal to or longer than remaining segments combined.

*Wings*: Developed in males and females. Forewings mottled with contrasting regions of brown, green, and pale tan, and sometimes dark black; the costal region wide relative to the wing length, the width between 4–8% the total wing length, always with light - dark regular banding. The forewings may be colored asymmetrically, one being mottled as described above while the other is either dark rust or blackened with the mottled pattern still slightly visible (darker wing typically folded under the mottled wing). Hindwings opaque and smoky; the distal terminus of the discoidal region darker than the rest; the costal region can be much darker or paler than the discoidal region depending on species; the terminus of the discoidal region projecting well beyond the distal margin of anal region, making the wing appear pointed or elongate.

*Abdomen*: Males and females with gradually widening abdomen from first segment until the beginning of the distal half (segments 5–7) when the lateral margins narrow to the terminus, the middle being the broadest region. Posterolateral corners of tergites simple, without projections. Cerci cylindrical, long and setose, tapering to a point. Supra-anal plate long (females) or transverse (males), with a rounded or more pointed terminus. Subgenital plate of male with rounded, slightly irregular terminus; without styli.

*Male genital complex*: The main body of ventral left sclerite (L4A) with a prominent and curved distal process (pda). The apofisis falloid (afa) of the main body of dorsal left sclerite (L4B) well sclerotized with a blunt, rounded terminus; the apical process (paa) cylindrical and curved, terminating with a rounded end; with or without a large membranous lobe originating between the apofisis falloid (afa) and the apical process (paa), if present then with or without robust setae. The right dorsal phallomere (fda) of the first sclerite of right phallomere (R1) tapers to a rounded terminus and is mostly membranous with setae; the ventral plate (pia) strongly sclerotized and short, but with a smooth surface; the ventral process (pva) strongly sclerotized and curved.

##### Ootheca.

Unknown for the genus.

##### Etymology.

A noun in apposition, the name is derived from the Latin noun "fuga" meaning flight or escape, and the corresponding verb "fugere" meaning to evade or escape. Many that have collected or attempted to collect these mantises will recognize their ability to disappear on the tree, never to be seen again.

##### Key to species

**Table:** 

1	Anterior (inner) surface of prothoracic femora of males pale with few black markings. Length of forewing of male 13 mm or less (observed range 12.76–13 mm). In females, the terminus of the discoidal region of the hindwing not projecting beyond the anal region, forming a blunt and rounded outer margin	*Fuga annulipes* (Audinet Serville, 1838)
–	Anterior (inner) surface of the prothoracic femora of males entirely black. Length of forewing of male 14 mm or greater (observed range 14.12–16.47 mm). In females, the terminus of the discoidal region of the hindwing projects beyond the outer profile of the anal region, forming a distinct separation from the discoidal and anal regions	2
2	Length of forewing of female 17.5 mm or greater (observed range 17.65–20.82 mm). Length of pronotum of male 5.8 mm or greater (observed range 5.87–6.53 mm). The main body of dorsal left sclerite (L4B) of the male genital complex with a membranous lobe positioned between the apofisis falloid (afa) and the apical process (paa), projecting well beyond the length of the pseudophallus; the lobe with robust, straight setae	*Fuga fluminensis* (Piza, 1965)
–	Length of forewing of female 17.1 mm or less (observed range 15.73–17.04 mm). Length of pronotum of male 5.7 mm or less (observed range 5.33–5.61 mm). The main body of dorsal left sclerite (L4B) of the male genital complex with a membranous bifurcate lobe positioned between the apofisis falloid (afa) and the apical process (paa), projecting well beyond the length of the apofisis falloid (afa); the bifurcate lobe with robust, straight setae positioned at each terminus and in a line along the mid-section	*Fuga grimaldii* sp. n.

##### Clave Para las Especies

**Table:** 

1	Cara interna de los fémurs anteriores del macho con unas cuantas marcas negras. Longitud de las alas anteriores del macho igual o menor a 13 mm (rango observado 12.76–13 mm). En las hembras, el ápice de la region discoidal de las alas posteriores no proyectandose más allá de la región anal	*Fuga annulipes* (Audinet Serville, 1838)
–	Cara interna de los fémurs anteriores del macho enteramente negros. Longitud de las alas anteriores del macho igual o mayor a 14 mm (rango observado 14.12–16.47 mm). En las hembras, el terminus de la region discoidal de las alas posteriores se proyecta más allá del margen de la region anal, creándose así una division clara entre ambas regiones	2
2	Longitud de las alas anteriores de la hembra mayor a 17.5 mm (rango observado 17.65–20.82 mm). Longitud del pronotum del macho igual o mayor de 5.8 mm (rango 5.87–6.53 mm). Esclerito dorsal izquierdo (L4B) de la genitalia del macho con un lobulo membranáceo ubicado entre el apofisis falloid (afa) y el apical process (paa), proyectandose mas allá de la longitud del apofisis falloid (afa); el lóbulo membranáceo tiene setas robustas y rectas	*Fuga fluminensis* (Piza, 1965)
–	Longitud de las alas anteriores de la hembra igual o menor a 17.1 mm (rango observado 15.73–17.04 mm). Longitud del pronotum del macho igual o menor a 5.7 mm (rango 5.33–5.61 mm). Esclerito dorsal izquierdo (L4B) de la genitalia del macho con un lobulo membranáceo bifurcado ubicado entre el apofisis falloid (afa) y el apical process (paa), proyectandose mas allá de la longitud del apofisis falloid (afa); el lóbulo bifurcado es robusto, con setas robustas y rectas en ambos extremos	*Fuga grimaldii* sp. n.

#### Fuga annulipes

(Audinet Serville, 1838)

http://species-id.net/wiki/Fuga_annulipes

Mantis annulipes : Audinet Serville 1838: 199.
Mantis (Oxypilus) annulipes : De Haan 1842: 84.
Liturgousa annulipes : Saussure 1869: 62; Brauer 1870: 92; Saussure 1871b: 102 (female only; *partim*); Westwood 1889: 4, 49, pl. 2, fig. 3; Kirby 1904: 271; Werner 1906: 372; Chopard 1911: 323; Chopard 1916: 164; Rehn 1935: 199, pl. 8, fig. 4; Hughes-Schrader 1943: 266, 280, Table 1, Figs 19–28; Hughes-Schrader 1948: 267; Hughes-Schrader 1950: 11, Table 1; Cerdá 1996: 75.
Liturgusa annulipes : Hebard 1922: 337; Giglio-Tos 1927: 294; Beier 1935: 11; Terra 1995: 53, Figs 85–87; Jantsch 1999: 48; Ehrmann 2002: 206; Otte and Spearman 2005: 132; Agudelo et al. 2007: 116, 141.
Hagiomantis parva : Piza 1966: 8; Terra 1995: 54; Ehrmann 2002: 164; Otte and Spearman 2005: 130. syn. n.
Liturgusa sinvalnetoi : Piza 1982: 94; Terra 1995: 54; Jantsch 1999: 48; Ehrmann 2002: 207; Otte and Spearman 2005: 133; Agudelo et al. 2007: 116. syn. n.
Liturgusa parva : Giglio-Tos 1914: 77–78; Giglio-Tos 1927: 295; Beier 1935: 11; Terra 1995: 54; Jantsch 1999: 48; Ehrmann 2002: 207; Otte and Spearman 2005: 133; Agudelo et al. 2007: 116. syn. n.

##### Lectotype.

Female. Ernst-Moritz-Arndt-Universität Greifswald, Germany.

##### Type locality.

Bahia, Brasilla, Fruhstorfer.

##### Material examined.

*Fuga annulipes* (Audinet Serville, 1838).

**Table:** 

Sex	Type	Country	Label	Latitude Longitude	Code
Female	Lectotype	Brazil	Bahia, Brasilia, Fruhstorfer		EMAU
Female	Paralectotype	Brazil	a. Mant. annuli-, pede Charn. tl., 28 Differt cotta alarum fusca		EMAU
Female	Holotype (*Liturgusa sinvalnetoi*)	Brazil			DZES
Female	Holotype (*Liturgusa parva*)	Brazil			ZMHB
Female	Holotype (*Hagiomantis parva*)	Brazil			DZES
Female	nontype	Brazil	Rio de Jan., Acc.No.2966, Oct.	-22.941750, -43.221293	ANSP 104
Female	nontype	Brazil	Rio de Jan., IV 02, Acc.No.2066	-22.941750, -43.221293	ANSP 105
Female	nontype	Brazil			BMNH 003
Female	nontype	Brazil			BMNH 006
Male	nontype	Brazil			OUMHN 006
Female	nontype	Brazil			OUMNH 016
Female	nontype	Brazil	29-9-29, S. Paulo, Est. S. Paulo, Luderwald coll.	-23.629236, -46.393254	SDEI 003
Female	nontype	Brazil	Bahia, 1930, on tree trunk	-12.410253, -39.903680	USNM 015; USNM ENT 00873031
Male	nontype	Brazil	Espirito Santo, J. Michaelis vend. 22.IV.1898	-19.959049, -40.646506	ZMUH 006
Male	nontype	Brazil	Espirito Santo, J. Michaelis vend. 22.IV.1898	-19.959049, -40.646506	ZMUH 007
Female	nontype	Brazil	(Prov. Rio de Jan.), Coll. v. Bonninghausen (20.X.1906)	-22.604492, -43.222469	ZMUH 009
Female	nontype	Brazil	Bahia, Fruhstorfer, Bahia (Brasilien) H. Fruhstorfer, vend. 1.IX.1896	-12.410253, -39.903680	ZMUH 017

##### Taxonomic history.

The oldest described species of Neotropical Liturgusini, its proper identification is rarely achieved historically. It appears to be a default identification for *Liturgusa* specimens, giving a highly inaccurate account of the species’ distribution and morphological characteristics. When the types were compared to other specimens in this study, it was immediately clear that this species was not a *Liturgusa* at all, but a different and geographically isolated species unlike all other *Liturgusa* species.

When describing *Mantis annulipes* in 1838 Audinet Serville provided a relatively lengthy description, but was ambiguous when referencing the locality of the specimen or specimens by only listing “Of Brazil and of Cayenne”. He did reference that the description was based on the female form, but it is unknown if there were one or multiple specimens examined; the distribution information presented suggests multiples. The specimens are presumed to come from the collection of MM. Dejean et Viard, but the current location of Audinet Serville’s specimens is not known. The catalog of Reinhard Ehrmann listed the holotype female as deposited in the Muséum national d’Histoire naturelle, Paris, and paratypes deposited in the Ernst-Moritz-Arndt-Universität Greifswald, Germany. The specimens from EMAU were located and examined, but the holotype in MNHN was not located. A search did not reveal its presence in the collection historically. It is not truly known whether the two EMAU specimens are those examined by Audinet Serville, but they date from the correct period and are from locations in Brazil. However, since a holotype was never designated by Audinet Serville and no subsequent fixation was uncovered, the specimens are treated as syntypes. Therefore, to increase taxonomic stability within the species, under Article 74.1 of the International Code of Zoological Nomenclature a the female from Bahia, Brazil deposited in EMAU has been selected to become the unique bearer of the name of the nominal species-group *Fuga annulipes* (lectotype). The additional specimen also deposited in EMAU is selected as a paralectotype under Article 74.1.3 of The Code.

The types of three species, *Hagiomantis parva* Piza, 1966, *Liturgusa sinvalnetoi* Piza, 1982, and *Liturgusa parva* Giglio-Tos, 1914, were examined and compared with the original description of *Mantis annulipes* and the two specimens from EMAU and were found to be conspecific. These three new synonymies are likely the result of the historical confusion surrounding the distribution and characteristics of *Fuga annulipes*.

##### Diagnosis.

A small species, males being darkly colored with highly contrasting grey and black on forewings. Females also with a darker coloration, but most noticeable is the less elongate pronotum compared to the other two species in the genus. Their squat appearance should be obvious when observed in the field as they are the only species known in eastern Brazil with such short pronota.

##### Description.

**Figure 30.**
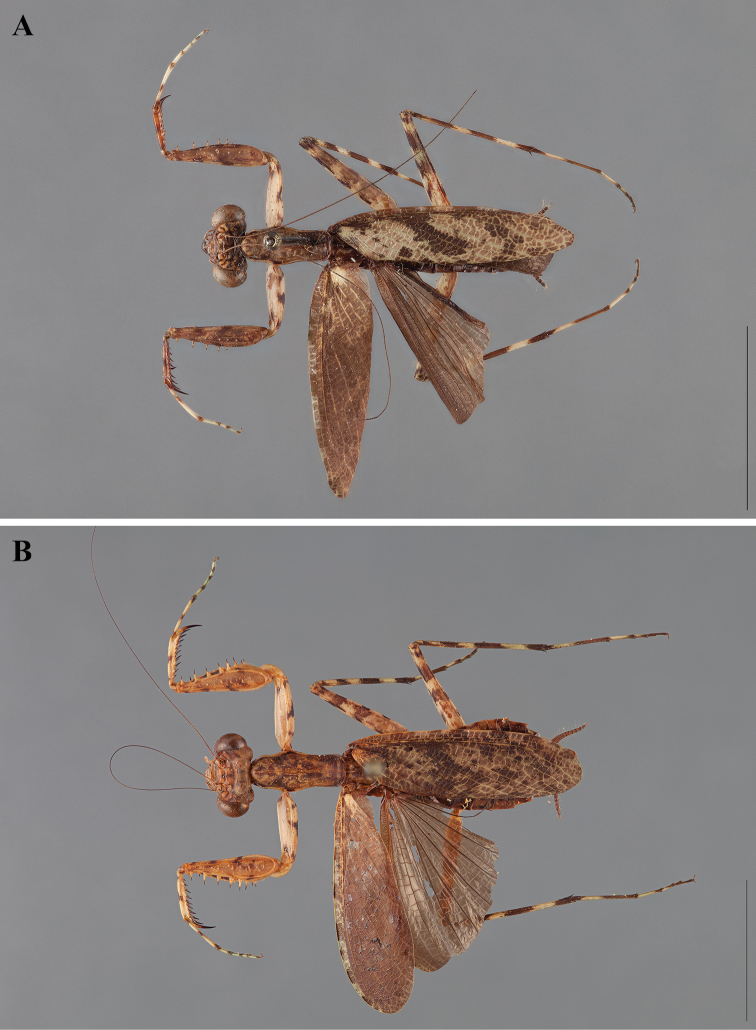
*Fuga annulipes* (Audinet Serville, 1838), dorsal habitus: **A** male from Espirito Santo, Brazil (ZMUH 006) **B** female from Rio de Janeiro, Brazil (ANSP 105).

**Male.** (Fig. 30A) N=3: Body length 18.84–19.03 (18.93); forewing length 12.76–13.00 (12.89); hindwing length 10.23–10.58 (10.40); pronotum length 4.75–4.96 (4.86); prozone length 1.54–1.64 (1.60); pronotum width 2.02–2.20 (2.10); pronotum narrow width 1.66–1.75 (1.69); head width 4.33–4.63 (4.51); head vertex to clypeus 1.60–1.64 (1.62); frons width 1.54–1.59 (1.57); frons height 0.52–0.59 (0.55); prothoracic femur length 5.09–5.38 (5.23); mesothoracic femur length 6.58–6.72 (6.65); mesothoracic tibia length 5.20–5.27 (5.23); mesothoracic tarsus length 4.79–5.16 (4.97); metathoracic femur length 6.33–6.89 (6.64); metathoracic tibia length 6.86–7.44 (7.21); metathoracic tarsus length 6.98–7.88 (7.43); pronotal elongation measure 0.32–0.33 (0.33); pronotal shape measure 0.43–0.44 (0.43); head shape measure 0.34–0.38 (0.36); frons shape measure 0.34–0.37 (0.35); anteroventral femoral spine count 15; anteroventral tibial spine count 9; posteroventral tibial spine count 8.

*Head* (Fig. 45E): Juxta-ocular protuberances small, the middle being the most pronounced; the vertex between the parietal sutures is slightly concave; vertex lower than the dorsal margin of the eyes. Frontal suture with a curved carina forming a continuous arc. Ocelli small with the central ocellus oriented anteriorly while the lateral are oriented slightly off 90 degrees from the central axis of the head; protruding slightly on cuticular mounds. Frons narrowed between the antennal insertion sites and depressed below the central ocellus; a transverse carina present below the central ocellus, running from lateral margins under the antennal insertion sites medially in a dorsally oriented curve. Upper margin of clypeus slightly convex, lower margin slightly concave; a central protruding carina; the lateral margins tapering, widest at the upper margin. Antennae pale proximally and fading to brown distally. Varying levels of black markings across the anterior surface of head; the vertex, juxta-ocular protuberances, frons, mandibles, and labrum with black and pale mottling, but giving a darker appearance. Palpi are darkened terminally.

*Pronotum* (Fig. 50A): Shortened, but not squat with a slightly defined supra-coxal bulge; dorsal surface mostly smooth with a few tubercles present; prozone with parallel lateral margins prior to a rounded anterior margin; metazone with concave lateral margins, smooth or at most with tiny tubercles associated with setae; posterior margin of the metazone rounded with a slight medial emargination; the dorsal surface of the posterior half of the metazone slightly depressed. Supra-coxal sulcus strongly defined. The lateral margins of the pronotum slightly expanded to form a small ledge. Colored with black and pale markings that vary across specimens.

*Prothoracic Legs*: Femur robust with a straight dorsal margin. Brown to dark banding on posterior (outer) surface of femur with less than 10 tubercles; anterior (internal) surface amber or pale with one black spot in the groove for the tibial spur; ventral surface amber or pale. Well developed femoral pit on the ventral surface to accommodate terminal posteroventral tibial spine positioned medial to the most proximal posteroventral spine; pit is black. Posterior prothoracic femoral genicular spine slightly smaller than posteroventral spines, originating distal to the beginning of the genicular lobe. Prothoracic tibial posteroventral spines with the first and second (proximal) short with the fourth through seventh of similar length, the third and terminal spines much longer; the anteroventral spines longest at distal end and shortening proximally. Posterior surface of the prothoracic tibiae smooth and banded, but dark brown; anterior surface amber, the ventral surface amber. Prothoracic coxae smooth with varying black markings on the posterior and ventral surface, the anterior surface with a proximal (near center) and a distal black marking.

*Meso- and Metathoracic Legs*: Femora with a pronounced dorsal and ventral carina; posterior (upper) surface with two carinae, one running nearly the entire length, fading proximally and positioned ventrally to the dorsal carina, the other in the distal third and positioned parallel and between the previous and the ventral carina. Mesotarsi with first segment as long as the remaining segments combined. Metatarsi with first segment slightly longer than remaining segments combined.

*Wings*: The same length or slightly longer than the abdomen. Forewings mottled with contrasting regions of black and pale white or grey; the costal region alternating from pale to dark its entire length, mostly brown proximally; the costal region slightly widened. The forewings may be colored asymmetrically, one being mottled as described above while the other is much darker and usually folded under the other. Hindwings opaque and smoky with near black veins; the terminus of the discoidal region truncate and not projecting beyond the distal margin of anal region, giving a stubby appearance.

*Abdomen*: Elongate, tubular, and smooth. Tergites without posterolateral tergal projections. Cerci cylindrical, long and setose, tapering to a point. Supra-anal plate transverse, evenly rounded. Subgenital plate rounded and without styli.

*Genital Complex* (Fig. 53A.1–A.2): The main body of ventral left sclerite (L4A) with a prominent, thin, curved distal process (pda), the entire structure curving laterally and back onto the L4A. The apofisis falloid (afa) of the main body of dorsal left sclerite (L4B) well sclerotized and roughly textured with a short, broad terminus, the tip broader than medial region; the apical process (paa) cylindrical and gently curved, terminating with a rounded terminus; numerous, very long, slightly curved setae emerging from a central point between the apofisis falloid (afa) and the apical process (paa) that extend distally as long as the apical process. The right dorsal phallomere (fda) of the first sclerite of right phallomere (R1) tapers to a broadly, rounded and well sclerotized terminus and has few fine setae; the ventral plate (pia) strongly sclerotized, long and thin, but without a knob or tooth proximally, the surface smooth; the ventral process (pva) strongly sclerotized, large and curved, tapering to a point, each edge serrated with small, pointed teeth.

##### Redescription.

**Figure 31.**
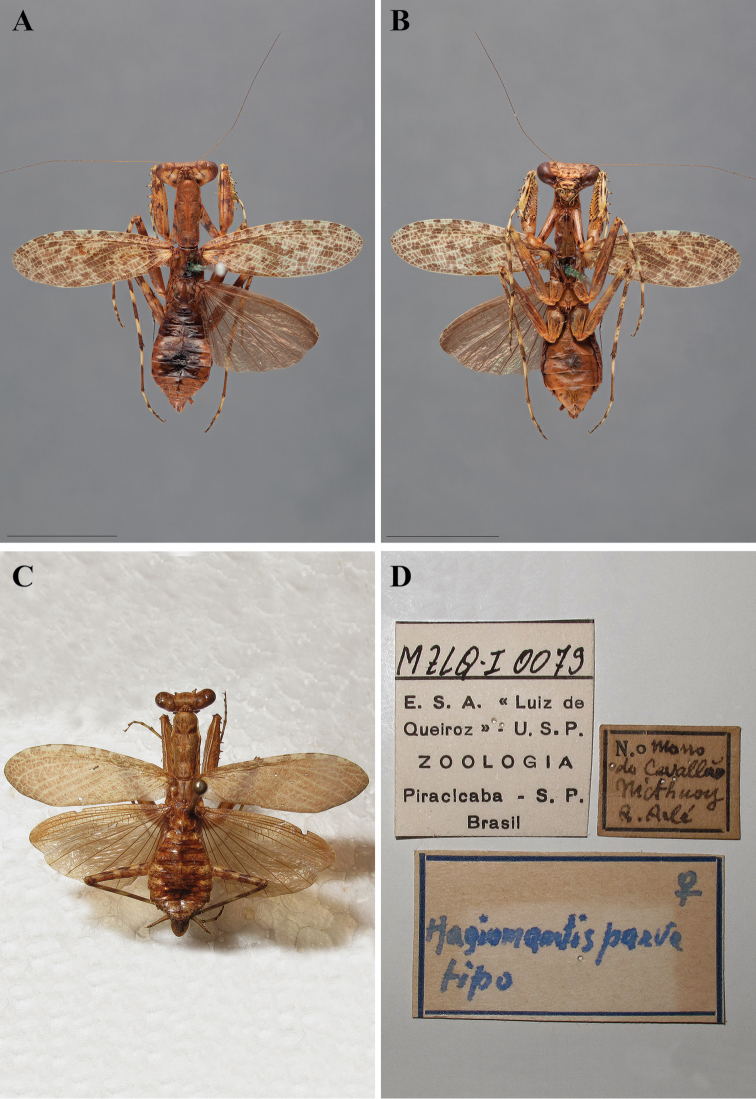
*Fuga annulipes* (Audinet Serville, 1838), types and labels. *Mantis annulipes* Audinet Serville, 1838, lectotype female (EMAU): **A** dorsal habitus **B** ventral habitus. *Hagiomantis parva* Piza, 1966 syn. n. (DZES), holotype female: **C** dorsal habitus **B** labels.

**Figure 32.**
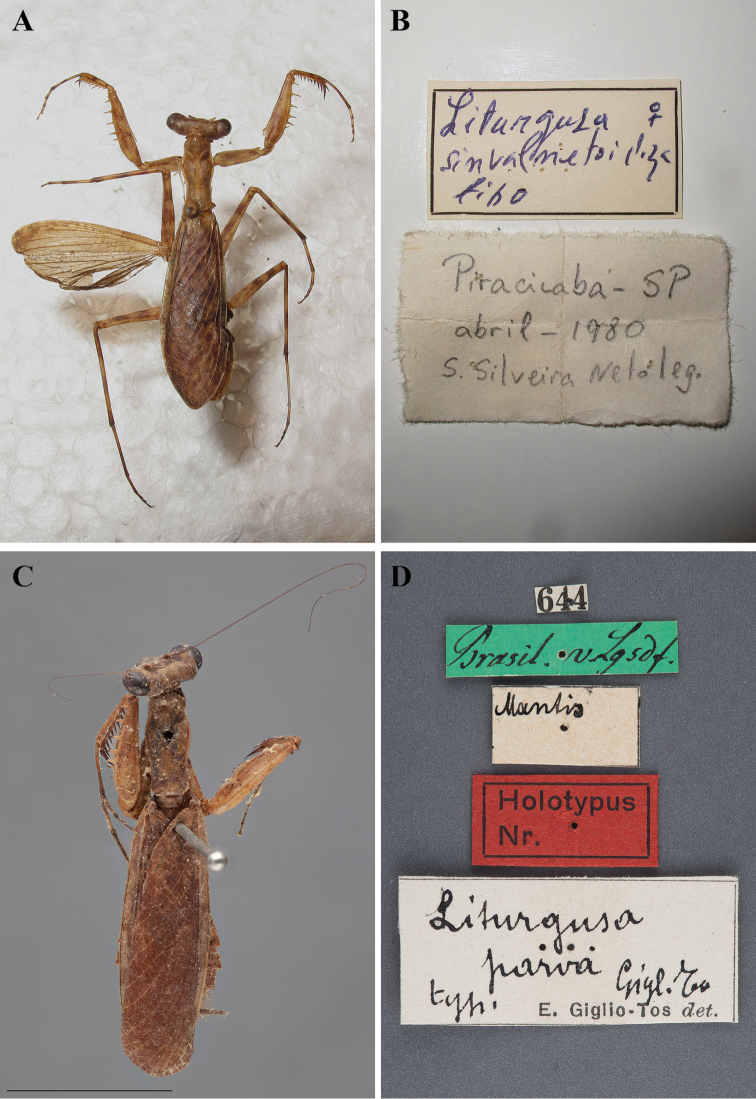
*Fuga annulipes* (Audinet Serville, 1838), types and labels. *Liturgusa sinvalnetoi* Piza, 1982 syn. n., holotype female (DZES): **A** dorsal habitus **B** labels. *Liturgusa parva* Giglio-Tos, 1914 syn. n., holotype female (ZMHB): **C** dorsal habitus **D** labels.

**Female.** (Figs 30B, 31A–C, 32A, 32C) N=10: Body length 21.70–26.74 (24.25); forewing length 14.89–18.41 (16.26); hindwing length 12.32–13.77 (12.92); pronotum length 5.75–6.74 (6.20); prozone length 1.93–2.25 (2.06); pronotum width 2.55–2.93 (2.70); pronotum narrow width 1.98–2.30 (2.13); head width 5.30–6.05 (5.65); head vertex to clypeus 2.08–2.35 (2.21); frons width 2.00–2.32 (2.16); frons height 0.67–0.88 (0.76); prothoracic femur length 6.26–7.18 (6.65); mesothoracic femur length 7.22–8.24 (7.63); mesothoracic tibia length 5.83–6.73 (6.22); mesothoracic tarsus length 5.26–6.13 (5.63); metathoracic femur length 7.24–8.23 (7.70); metathoracic tibia length 8.07–9.84 (8.75); metathoracic tarsus length 7.81–9.76 (8.61); pronotal elongation measure 0.32–0.34 (0.33); pronotal shape measure 0.41–0.49 (0.44); head shape measure 0.38–0.40 (0.39); frons shape measure 0.33–0.38 (0.35); anteroventral femoral spine count 14–16 (15); anteroventral tibial spine count 9; posteroventral tibial spine count 8.

*Head* (Fig. 45F): Juxta-ocular protuberances moderate size, the middle being the most pronounced; the vertex between the parietal sutures is slightly concave; vertex higher than the dorsal margin of the eyes. Ocelli protruding slightly on a carina connecting all three and extending laterally. Varying levels of black markings across the anterior surface of head; the vertex, juxta-ocular protuberances, frons, mandibles, and labrum with fine black speckling over a largely pale coloration, giving a lighter appearance than males. Palpi are darkened brown terminally.

*Pronotum* (Fig. 50B): Shortened, but not squat with a slightly defined supra-coxal bulge; dorsal surface mostly smooth, but some tubercles are present; prozone with parallel lateral margins prior to a rounded anterior margin; metazone with concave lateral margins, with small tubercles in the posterior two thirds; posterior margin of the metazone rounded with no medial emargination.

*Prothoracic Legs*: Femur robust with a nearly straight dorsal margin; anteroventral spines black, posteroventral spines pale basally with a black terminus. Prothoracic tibial posteroventral spines with the first and second most proximal and fifth through seventh shorter than the proximal third, fourth and terminal spines; the anteroventral spines longest at distal end and shortening proximally, but the sixth and seventh from the distal end longer than adjacent spines. Posterior surface of the prothoracic tibiae smooth and banded; anterior surface pale, the ventral surface pale. Prothoracic coxae smooth with varying black markings on the posterior and ventral surface, the anterior surface with a small proximal (near center) and a small distal black marking.

*Meso- and Metathoracic Legs*: Femora with a pronounced dorsal and ventral (posterior) carina. Mesotarsi with first segment as long as or shorter than the remaining segments combined. Metatarsi with first segment slightly longer than remaining segments combined.

*Wings*: Costal region of hindwings and the anterior margin of the discoidal region more pale than the rest.

*Abdomen*: Broad and smooth, widening from first segment until the beginning of the distal half (segments 4–5) when the lateral margins narrow gradually to the terminus, the middle being the broadest region. Tergites with small posterolateral projections on the sixth and seventh segments. Supra-anal plate longer than wide, broadly rounded.

#### Fuga fluminensis

(Piza, 1965)

http://species-id.net/wiki/Fuga_fluminensis

Hagiomantis fluminensis : Piza 1965: 130; Terra 1995: 54; Ehrmann 2002: 163; Otte and Spearman 2005: 129.

##### Type.

Holotype Male. Universidade de Sao Paulo, Piracicaba, Brazil.

##### Type locality.

Brazil: Itaipu, E. da Guanabara, Col: D. Lacombe - 5-2-61 (Lat. -22.950912, Long. -43.036068).

##### Material examined.

*Fuga fluminensis* (Piza, 1965).

**Table:** 

Sex	Type	Country	Label	Latitude Longitude	Code
Male	Holotype	Brazil	Patria: Itaipu, E. da Guanabara, Col: D. Lacombe - 5-2-61.	-22.950912, -43.036068	DZES
Male	nontype	Brazil			BMNH 010
Female	nontype	Brazil			OUMNH 004
Female	nontype	Brazil			OUMNH 005
Female	nontype	Brazil			OUMNH 014
Female	nontype	Brazil			OUMNH 018
Female	nontype	Brazil			OUMNH 019
Female	nontype	Brazil	Para, Sieber	-25.668076, -48.750323	ZMHB 003
Female	nontype	Brazil	(Prov. Rio de Jan.), Coll. v. Bonninghausen (20.X.1906)	-22.604492, -43.222469	ZMUH 010
Female	nontype	Brazil	Espirito Santo, J. Michaelis vend. 22.IV.1898	-19.959049, -40.646506	ZMUH 011
Female	nontype	Brazil	R.d. Janeiro, Petropolis, F. Ohs, 2.2.99, Dr. Fr. Ohaus, vend. 20.VI.1911	-22.503159, -43.183477	ZMUH 012
Female	nontype	Brazil	Prv. Rio de Janeiro, Grz. m. Minas Geraes, Fr. Wiengreen leg., ded. 1. XI. 1894	-22.034612, -43.228938	ZMUH 014
Female	nontype	Brazil	Santos, J. Metz, leg., ded. 30. V. 1894.	-23.944261, -46.343691	ZMUH 016
Male	nontype	Brazil			OUMNH 002

##### Taxonomic history.

Described in 1965 by Piza as a species of *Hagiomantis*, the species has since received no attention taxonomically.

##### Diagnosis.

Medium size species, mottling pattern highly contrasting with light and dark browns, no white, black, or green included. Costal region of the forewing one of the widest in the genus relative to the wing length, banded with regular alternating patterning between pale and dark brown.

##### Redescription.

**Figure 33.**
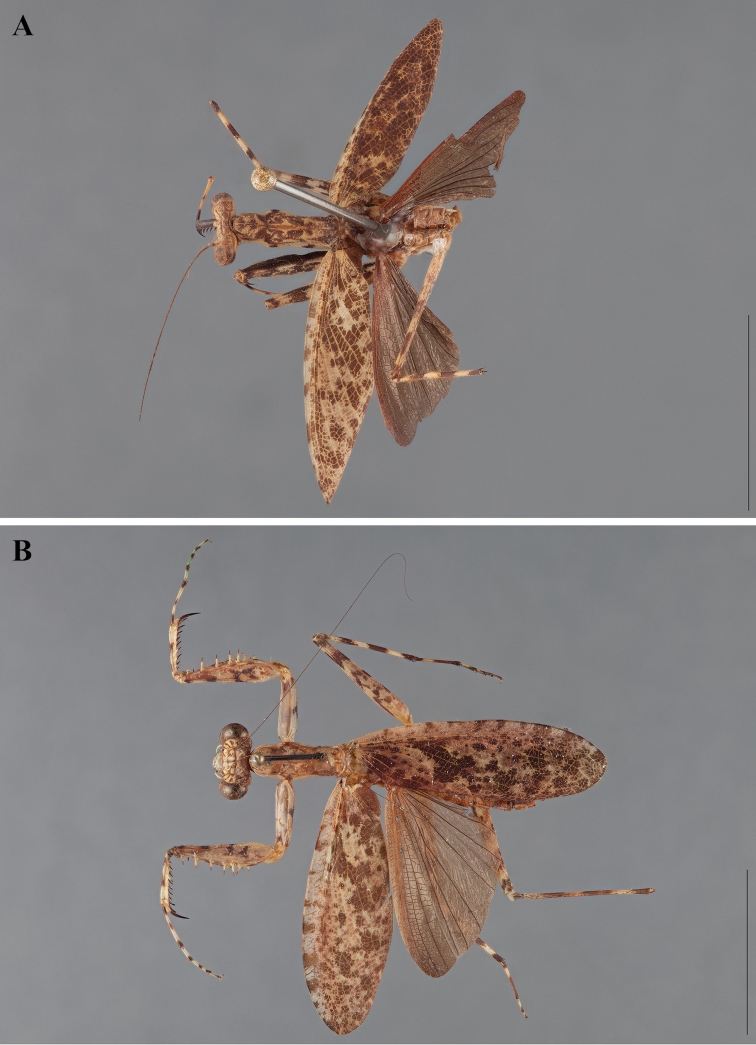
*Fuga fluminensis* (Piza, 1965), dorsal habitus: **A** male (BMNH 010) **B** female from Espirito Santo, Brazil (ZMUH 011).

**Male.** (Figs 33A, 37A) N=2: Body length measurement not possible; forewing length 14.57–16.47 (15.53); hindwing length 11.34–11.89 (11.61); pronotum length 5.87–6.53 (6.20); prozone length 1.92–2.12 (2.02); pronotum width 2.21–2.42 (2.32); pronotum narrow width 1.72–1.77 (1.75); head width 4.54–4.88 (4.71); head vertex to clypeus 1.59–1.86 (1.73); frons width 1.49–1.63 (1.56); frons height 0.58–0.65 (0.62); prothoracic femur length 6.04–6.39 (6.21); mesothoracic femur length 8.67; mesothoracic tibia length 7.00; pronotal elongation measure 0.33; pronotal shape measure 0.37–0.38 (0.37); head shape measure 0.35–0.38 (0.37); frons shape measure 0.39–0.40 (0.40); anteroventral femoral spine count 15–17 (17); anteroventral tibial spine count 9; posteroventral tibial spine count 8.

*Head* (Fig. 45G): Juxta-ocular protuberances small, the middle being the most pronounced; the vertex between the parietal sutures is slightly concave; vertex slightly lower than the dorsal margin of the eyes. Frontal suture with a curved carina medially with an obvious angle. Ocelli small with the central ocellus oriented anteriorly while the lateral are oriented slightly off 90 degrees from the central axis of the head; protruding slightly on cuticular mounds. Frons narrowed between the antennal insertion sites and depressed below the central ocellus; a transverse carina present below the central ocellus, running from lateral margins under the antennal insertion sites medially in a dorsally oriented curve. Upper margin of clypeus slightly convex, lower margin slightly concave; a central protruding ridge strongly defined; the lateral margins tapering, widest at the upper margin. Antennae pale proximally and fading to black distally. Varying levels of black markings across the anterior surface of head, variable within the species; the vertex and juxta-ocular protuberances dark brown; two black spots medial to the parietal sutures. Palpi are pale.

*Pronotum* (Fig. 50C): Slightly elongate with a slightly defined supra-coxal bulge; dorsal surface mostly smooth, but with a few very small tubercles; prozone with parallel lateral margins prior to a rounded anterior margin. Metazone with concave lateral margins with a slight medial bulge, exhibiting small denticles in the posterior two thirds; posterior margin of the metazone with angled margins leading to a straight or slightly concave posterior margin, no noticeable medial emargination; the dorsal surface of the posterior half of the metazone depressed. Supra-coxal sulcus strongly defined. The lateral margins of the pronotum slightly expanded to form a small ledge. Colored with black and pale markings that vary across specimens.

*Prothoracic Legs*: Femur robust with a straight or slightly concave dorsal margin; anteroventral spines black, posteroventral spines pale basally with black terminus. Black and pale mottling on posterior (external) surface of femur with less than 10 tubercles; anterior (internal) surface entirely black; ventral surface entirely black. Well developed femoral pit on the ventral surface to accommodate terminal posteroventral tibial spine positioned medial to and slightly proximal to the most proximal posteroventral spine; pit is black. Posterior prothoracic femoral genicular spine smaller than posteroventral spines, originating at the beginning of the genicular lobe. Prothoracic posteroventral tibial spines with the first and second most proximal and fourth through seventh shorter than the proximal third and terminal spines; the anteroventral spines longest at distal end and shortening proximally. Posterior surface of the prothoracic tibiae smooth and dark; anterior surface black, the ventral surface brown. Prothoracic coxae smooth with varying black markings on the posterior and ventral surface, the anterior surface with a medial black stripe.

*Meso- and Metathoracic Legs*: Femora with pronounced dorsal and ventral carinae; posterior (upper) surface smooth. Tarsi missing.

*Wings*: Longer than the abdomen. Forewings mottled with contrasting regions of light and dark brown; the costal region alternating regularly from pale to dark its entire length; the costal region slightly widened. The forewings may be colored asymmetrically, one being mottled as described above while the other is much darker. Hindwings opaque and smoky with near black veins; the costal region and the anterior margin of the discoidal region pigmented with a darker reddish or rust coloration; the terminus of the discoidal region projecting well beyond the distal margin of anal region, giving an elongate appearance.

*Abdomen*: Elongate, tubular, and smooth. Tergites without posterolateral projections. Supra-anal plate transverse, evenly rounded. Subgenital plate rounded and without styli.

*Genital Complex* (Fig. 53B.1–B.2): The main body of ventral left sclerite (L4A) with a prominent, robust, curved distal process (pda) that tapers quickly to a point, the entire structure curving laterally and back onto the L4A. The apofisis falloid (afa) of the main body of dorsal left sclerite (L4B) small and curved, terminating with a blunt end; the apical process (paa) cylindrical and curved, terminating in a rounded end, slightly bulbous; a large, long membranous lobe originating between the apofisis falloid (afa) and the apical process (paa). Right phallomere (R1) too damaged for accurate description.

##### Description.

**Female.** (Fig. 33B) N=8: Body length 25.26–29.89 (27.22); forewing length 17.65–20.82 (19.07); hindwing length 14.11–16.07 (14.77); pronotum length 6.69–7.85 (7.38); prozone length 2.17–2.62 (2.45); pronotum width 2.73–3.04 (2.92); pronotum narrow width 2.14–2.34 (2.25); head width 5.48–6.14 (5.92); head vertex to clypeus 2.12–2.39 (2.29); frons width 2.02–2.18 (2.10); frons height 0.73–0.88 (0.79); prothoracic femur length 6.97–8.17 (7.73); mesothoracic femur length 7.92–9.92 (9.15); mesothoracic tibia length 6.16–8.28 (7.38); mesothoracic tarsus length 6.16–7.53 (6.92); metathoracic femur length 8.38–10.01 (9.26); metathoracic tibia length 9.35–11.50 (10.46); metathoracic tarsus length 9.90–10.94 (10.42); pronotal elongation measure 0.32–0.34 (0.33); pronotal shape measure 0.37–0.42 (0.40); head shape measure 0.37–0.40 (0.39); frons shape measure 0.35–0.40 (0.38); anteroventral femoral spine count 14–16 (15); anteroventral tibial spine count 9–10 (9); posteroventral tibial spine count 8.

*Head* (Fig. 45H): Juxta-ocular protuberances pronounced, the apex in the middle; the vertex between the parietal sutures is straight or slightly concave; vertex higher than the dorsal margin of the eyes. Frontal suture with a curved carina forming a continuous arc. Antennae pale proximally and fading to black or dark brown distally. Varying levels of black markings across the anterior surface of head, variable within the species; the vertex and juxta-ocular protuberances dark brown; two black spots medial to the parietal sutures. Terminus of palpi are darkened.

*Pronotum* (Fig. 50D): Slightly elongate with a somewhat defined supra-coxal bulge; dorsal surface mostly smooth, but a few very small tubercles are present; prozone with parallel or divergent lateral margins prior to a rounded anterior margin; metazone with concave lateral margins with a medial bulge, exhibiting sparse small denticles in the posterior two thirds; posterior margin of the metazone rounded irregularly with a slight medial emargination. Supra-coxal sulcus strongly defined. The lateral margins of the pronotum slightly expanded to form a small ledge. Colored with black and pale markings that vary across specimens.

*Prothoracic Legs*: Black and pale banding on posterior surface of femur with less than 10 tubercles; anterior (inner) surface with a proximal black stripe as well as a distal black stripe medially; ventral surface with a black mark distally and a black mark just medial to second most proximal posteroventral spine. Posterior prothoracic femoral genicular spine slightly smaller than posteroventral spines, originating just distal to the beginning of the genicular lobe. Prothoracic posteroventral tibial spines with the first and second most proximal and fifth through seventh shorter than the proximal third, fourth and terminal spines; the anteroventral spines longest at distal end and shortening proximally. Posterior surface of the prothoracic tibiae smooth with few black marks; anterior surface pale with black marks on dorsal margin, the ventral surface pale. Prothoracic coxae smooth with varying black markings on the posterior and ventral surface, the anterior surface with two black markings of varying size, one in the proximal half and one in the distal half.

*Meso- and Metathoracic Legs*: Mesotarsi with first segment as long as the remaining segments combined. Metatarsi with first segment slightly longer than remaining segments combined.

*Wings*: Fully developed, longer than the abdomen. Forewings mottled with contrasting regions of light and dark brown; the costal region alternating regularly from pale to dark its entire length; the costal region widened, the widest part being in the middle of the wing. The forewings have not been observed to be asymmetrically colored.

*Abdomen*: Broad and smooth, widening from first segment until the beginning of the distal half (segments 4–5) when the lateral margins narrow gradually to the terminus, the middle being the broadest region. Tergites without posterolateral tergal projections. Cerci cylindrical, long and setose, tapering to a point. Supra-anal plate as long as wide, rounded.

#### Fuga grimaldii sp. n.

http://zoobank.org/A60690AD-6F8A-48F1-8E8B-8624F32821C9

http://species-id.net/wiki/Fuga_grimaldii

##### Type.

Holotype Male, pinned. American Museum of Natural History, New York, NY, USA.

##### Type locality.

Brazil: Corupa S. Cath. (Hansa Humbolt), Feb. 1949, A. Maller Coll., Frank Johnson Donor (Lat. -26.425027, Long. -49.247112).

##### Material examined.

*Fuga grimaldii* sp. n.

**Table:** 

Sex	Type	Country	Label	Latitude Longitude	Code
Male	Holotype	Brazil	Corupa S. Cath. (Hansa Humbolt), Feb. 1949, A. Maller Coll., Frank Johnson Donor	-26.425027, -49.247112	AMNH 019
Female	Allotype	Brazil	Etat de Espirito Santo, L. Desutter-Grandcolas rec., Linhares, Reserve forestiere, Compagnie Vale do Rio Doce, 2-XI-1992, Nuit 35_Trone	-19.415407, -39.964626	MNHN 048
Male	Paratype	Brazil	Corupa S. Cath. (Hansa Humbolt), Jan. 1948, A. Maller Coll., Frank Johnson Donor	-26.425027, -49.247112	AMNH 021
Female	Paratype	Brazil	Espirito Santo, Res. de Sooretama, IBAMA, S 19°3'20.7"W, 40°8'49", 11-X-1999, 44km NNE Linhares, Foret semi-decidue, P. Grandcolas & R. Peliens rec.	-19.055750, -40.146944	MNHN 026
Male	Paratype	Brazil	(Prov. Rio de Jan.), Coll. v. Bonninghausen (20.X.1906)	-22.604492, -43.222469	ZMUH 008
Female	Paratype	Brazil	Minas Geraes, Brasil, 1897, ex coll. Fruhstorfer, H. Fruhstorfer, vend. 15.IV.1898	-16.768176, -43.809992	ZMUH 013

##### Diagnosis.

Small species with a strongly defined darkened band running diagonally across the medial section of the forewing; forewings can be asymmetrically colored, one being darker than the other.

##### Description.

**Figure 34.**
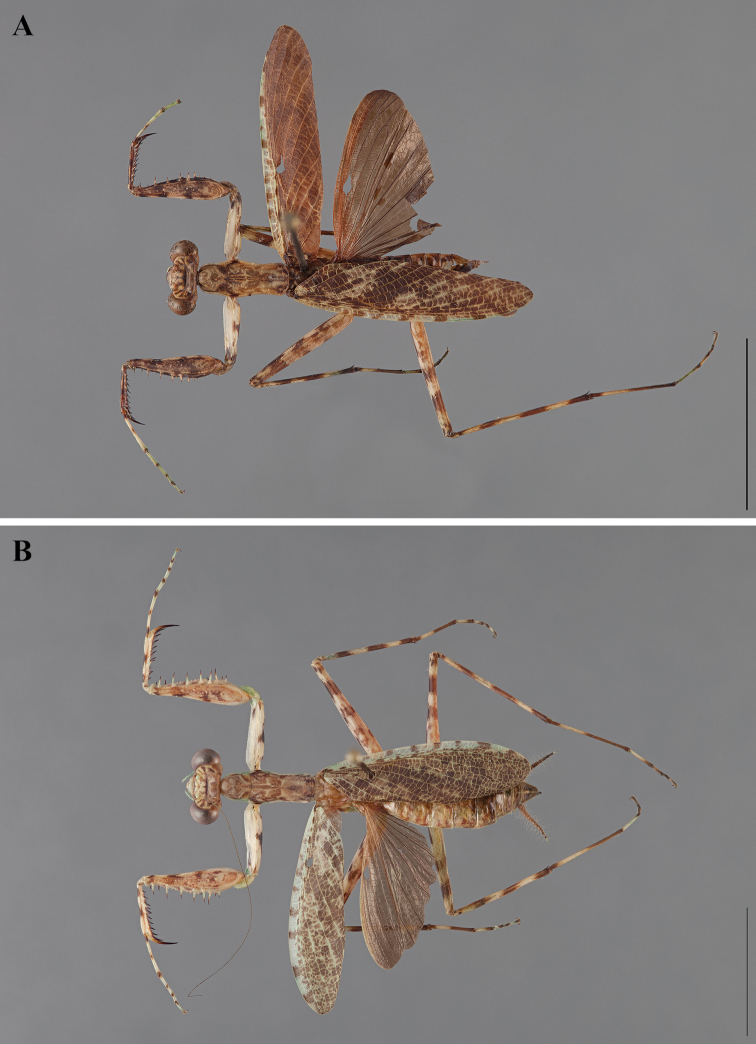
*Fuga grimaldii* sp. n., dorsal habitus: **A** holotype male from Brazil (AMNH 019) **B** allotype female from Brazil (MNHN 048).

**Male.** (Fig. 34A) N=2: Body length 20.42–22.11 (21.26); forewing length 14.12–15.41 (14.77); hindwing length 10.58; pronotum length 5.33–5.61 (5.47); prozone length 1.76–1.79 (1.77); pronotum width 2.14–2.20 (2.17); pronotum narrow width 1.75–1.87 (1.81); head width 4.36–4.62 (4.49); head vertex to clypeus 1.67–1.68 (1.68); frons width 1.50–1.56 (1.53); frons height 0.54–0.56 (0.55); prothoracic femur length 5.51–5.90 (5.70); mesothoracic femur length 7.26–7.31 (7.29); mesothoracic tibia length 5.89–6.22 (6.05); mesothoracic tarsus length 5.90–6.03 (5.97); metathoracic femur length 7.39; metathoracic tibia length 8.35; metathoracic tarsus length 9.11; pronotal elongation measure 0.32–0.33 (0.33); pronotal shape measure 0.39–0.40 (0.40); head shape measure 0.36–0.38 (0.37); frons shape measure 0.35–0.37 (0.36); anteroventral femoral spine count 15–16 (16); anteroventral tibial spine count 9; posteroventral tibial spine count 8.

*Head* (Fig. 46A): Juxta-ocular protuberances pronounced, the apex in the middle; the vertex between the parietal sutures is straight; vertex slightly lower than the dorsal margin of the eyes. Frontal suture with a curved carina forming a continuous arc. Ocelli small with the central ocellus oriented anteriorly while the lateral are oriented slightly off 90 degrees from the central axis of the head; protruding slightly on cuticular mounds. Frons narrowed between the antennal insertion sites and depressed below the central ocellus; a transverse carina present below the central ocellus, running from lateral margins under the antennal insertion sites medially in a dorsally oriented curve. Upper margin of clypeus slightly convex, lower margin slightly concave; a central protruding carina strongly defined; the lateral margins tapering, widest at the upper margin. Antennae pale proximally and fading to black distally. Varying levels of black markings across the anterior surface of head, variable within the species; the vertex and juxta-ocular protuberances dark brown. Palpi are pale.

*Pronotum* (Fig. 50E): Slightly elongate with a slightly defined supra-coxal bulge; dorsal surface smooth, but a few tubercles are present; prozone with parallel lateral margins prior to a rounded anterior margin; metazone with concave lateral margins, exhibiting small denticles in the posterior half; posterior margin of the metazone rounded and without a medial emargination; the dorsal surface of the posterior third of the metazone slightly depressed. Supra-coxal sulcus strongly defined. The lateral margins of the pronotum slightly expanded to form a small ledge. Colored with black and pale markings that vary across specimens.

*Prothoracic Legs*: Femur robust with a straight dorsal margin; anteroventral spines black, posteroventral spines pale basally with black terminus. Pale to dark banding on posterior (outer) surface of femur with less than 10 tubercles; anterior (inner) surface black; ventral surface black. Well developed femoral pit on the ventral surface to accommodate terminal posteroventral tibial spine positioned medial to and slightly proximal to the most proximal posteroventral spine; pit is black. Posterior prothoracic femoral genicular spine slightly smaller than posteroventral spines, originating distal to the genicular lobe. Prothoracic posteroventral tibial spines with the first and second most proximal and fifth through seventh shorter than the proximal third, fourth and terminal spines; the anteroventral spines longest at distal end and shortening proximally, but the sixth and seventh from the distal end longer than adjacent spines. Posterior surface of the prothoracic tibiae smooth and banded, but dark brown; anterior surface black; the ventral surface amber. Prothoracic coxae smooth with varying black markings on the posterior and ventral surface, the anterior surface with a proximal and a distal black marking.

*Meso- and Metathoracic Legs*: Femora with pronounced dorsal and ventral carinae. Mesotarsi with first segment as long as the remaining segments combined. Metatarsi with first segment longer than remaining segments combined.

*Wings*: Fully developed, the same length or slightly longer than the abdomen. Forewings mottled with contrasting regions of brown, white, and black; the costal region alternating regularly from pale to dark its entire length; the costal region slightly widened. The forewings may be colored asymmetrically, one being mottled as described above while the other is much darker and usually folded under the other. Hindwings opaque and smoky with near black veins; the costal region and the anterior margin of the discoidal region pigmented with a reddish or rust coloration; the terminus of the discoidal region projecting well beyond the distal margin of anal region, giving an elongate appearance.

*Abdomen*: Elongate, tubular, and smooth. Tergites with very small posterolateral projections in the distal half of the abdomen. Supra-anal plate transverse, evenly rounded. Subgenital plate rounded and without styli.

*Genital Complex* (Fig. 53C.1): The main body of ventral left sclerite (L4A) with a prominent, robust, curved distal process (pda) that tapers to a point, the entire structure curving laterally and back onto the L4A. The apofisis falloid (afa) of the main body of dorsal left sclerite (L4B) well sclerotized with a blunt, rounded terminus, the tip broader than medial region; the apical process (paa) cylindrical and curved, terminating in a rounded end; a large, bifid and membranous lobe originating between the apofisis falloid (afa) and the apical process (paa), with robust setae along a lateral margin as well as emerging from the terminus of each sub-lobe. The right dorsal phallomere (fda) of the first sclerite of right phallomere (R1) tapers to a rounded terminus and is mostly membranous with setae; the ventral plate (pia) strongly sclerotized and short, but with a smooth surface; the ventral process (pva) strongly sclerotized and curved, terminating with a rounded, blunt end.

**Female.** (Fig. 34B) N=4: Body length 21.94–26.39 (24.88); forewing length 15.73–17.04 (16.55); hindwing length 12.08–13.56 (13.00); pronotum length 7.02–7.36 (7.19); prozone length 2.32–2.40 (2.36); pronotum width 2.64–2.73 (2.68); pronotum narrow width 2.07–2.15 (2.11); head width 5.47–5.93 (5.75); head vertex to clypeus 2.14–2.26 (2.20); frons width 1.89–2.04 (1.98); frons height 0.71–0.80 (0.74); prothoracic femur length 7.47–7.79 (7.64); mesothoracic femur length 8.46–9.60 (9.17); mesothoracic tibia length 6.97–7.69 (7.40); mesothoracic tarsus length 6.88–7.26 (7.07); metathoracic femur length 8.76–9.47 (9.05); metathoracic tibia length 9.76–10.90 (10.31); metathoracic tarsus length 10.77–11.55 (11.16); pronotal elongation measure 0.32–0.34 (0.33); pronotal shape measure 0.36–0.38 (0.37); head shape measure 0.37–0.41 (0.38); frons shape measure 0.35–0.40 (0.37); anteroventral femoral spine count 14–16 (16); anteroventral tibial spine count 9; posteroventral tibial spine count 8.

*Head* (Fig. 46B): Vertex slightly above the dorsal margin of the eyes.

*Pronotum* (Fig. 50F): Metazone with concave lateral margins, exhibiting small denticles in the posterior two thirds; the dorsal surface of the posterior half of the metazone depressed.

*Prothoracic Legs*: Femur robust with a slightly concave dorsal margin distally, convex proximally; line of larger, pointed tubercles running medially of the posteroventral spines. Anterior (inner) surface of femur pale with proximal and dorso-distal black markings; ventral surface pale; femoral pit pale. Posterior prothoracic femoral genicular spine slightly smaller than posteroventral spines, originating just proximal to the genicular lobe. Posterior surface of the prothoracic tibiae smooth and banded; anterior surface mostly pale, the ventral surface pale. Prothoracic coxae smooth with varying black markings on the posterior and ventral surface, the anterior surface with a nearly continuous medial black marking running the entire length.

*Meso- and Metathoracic Legs*: Tibiae with a prominent ventral carina.

*Wings*: The forewings rarely colored asymmetrically, if so then one being mottled while the other is much darker. Hindwings opaque and smoky with near black veins; the costal region and the anterior margin of the discoidal region more pale than the rest; the terminus of the discoidal region projecting well beyond the distal margin of anal region, giving an elongate appearance.

*Abdomen*: Broad and smooth, widening from first segment until the beginning of the distal half (segments 4–5) when the lateral margins narrow gradually to the terminus, the middle being the broadest region. Tergites without posterolateral projections. Supra-anal plate as wide as long, tapering to a rounded point.

##### Etymology.

A noun in the genitive case, *Liturgusa grimaldii* is named for David Grimaldi, whose work on fossil Mantodea added considerable knowledge to our understanding of the origins and evolution of the group.

#### Velox gen. n.

http://zoobank.org/5698BB08-7A26-4A94-BC12-374349F85956

http://species-id.net/wiki/Velox

##### Type species.

*Velox wielandi* sp. n.

##### Description.

*Habitus*: One of the larger Neotropical Liturgusini, *Velox* is long and slender with tapered forewings, an elongate pronotum and long, slender meso and metathoracic legs. Coloration is heavily mottled and most observed males and the single female have asymmetrical wing coloration, one wing being much darker and folded under the other.

*Measurement Ranges*: Monotypic genus, see *Velox wielandi* for measurements.

*Head*: Transverse with large, rounded eyes projecting outside the profile of the head both laterally and anteriorly (the anterior margin of the eyes anterior to the central surface of the head). Juxta-ocular protuberances present, the lateral third being the most pronounced. The vertex between the parietal sutures is straight or barely concave. Frontal suture with a faint curved carina forming a continuous arc. Ocelli small with the central ocellus oriented anteriorly while the lateral are oriented anterolaterally at a 45 degree angle from the central axis of the head; protruding slightly on cuticular mounds. Frons narrowed between the antennal insertion sites and depressed below the central ocellus; a transverse carina present below the central ocellus, running from lateral margins under the antennal insertion sites medially in a dorsally oriented curve, the middle forming an angle. Upper margin of clypeus straight, lower margin concave or straight; a central protruding ridge strongly defined; the lateral margins tapering, widest at the upper margin. Labrum with minimal sculpting and a rounded terminus; lateral margins widening ventrally. Antennae filiform and with rare setae, pale proximally and fading distally to light brown in males and dark brown in females. Varying levels of black markings across the anterior surface of head, variable within the species. Palpi are pale.

*Pronotum*: Elongate with a slightly defined supra-coxal bulge; dorsal surface with tubercles; prozone long with parallel lateral margins prior to a rounded anterior margin; metazone long with sweeping concave lateral margins in males and concave, but near parallel margins medially; margins exhibit small, occasional denticles; posterior margin of the metazone straight or broadly rounded, with a slight medial emargination. Supra-coxal sulcus strongly defined. The lateral margins of the pronotum slightly expanded to form a small ledge. Colored with black and pale markings that vary across specimens.

*Prothoracic Legs*: Femur robust with a straight dorsal margin distally, the proximal third slightly convex; anteroventral and posteroventral (internal and external, respectively) spines well developed; line of small, pointed tubercles running medially of the posteroventral spines; anteroventral spines black, posteroventral spines pale basally with black terminus. A strongly pronounced continuous carina running from distal terminus of femur along dorsal margin to the base, circling the external surface of the proximal end and running along the ventral margin at the base of the posteroventral spines. Pale to dark banding on posterior surface of femur with numerous tubercles; anterior (inner) surface amber colored or pale ventrally, a black strip medially, and pale along the dorsal margin; ventral surface between the anteroventral and posteroventral spines amber or pale colored. Well developed femoral pit on the ventral surface to accommodate terminal posteroventral tibial spine positioned on the lateral margin, pushing the margin outward between the proximal two posteroventral spines; pit is pale. Prothoracic tibial spines robust; the posteroventral spines with the first and second most proximal and fourth through seventh shorter than the proximal third and terminal spines; the anteroventral spines longest at distal end and shortening proximally. Posterior surface of the prothoracic tibiae smooth and banded with pale and dark coloration; anterior and ventral surface amber colored. Tarsi banded with pale and dark coloration. Prothoracic coxae smooth with varying black markings on the posterior and ventral surface, the anterior surface mostly black, but with varying pale marks.

*Meso- and Metathoracic Legs*: Long and slender with pale to dark banding on the femur and tibia. Femora with pronounced dorsal and ventral carinae; posterior (upper) surface smooth. Tibiae banded with alternating pale and dark regions with a pronounced ventral carina. Mesotarsi with first segment as long or shorter than the remaining segments combined. Metatarsi with first segment longer than remaining segments combined.

*Wings*: Fully developed, the same length or slightly longer than the abdomen. Forewings mottled with contrasting regions of brown, white, and black; the costal region alternating irregularly from pale to dark its entire length; the costal region widened. The forewings may be colored asymmetrically, one being mottled as described above while the other is much darker and usually folded under the other. Hindwings opaque and smoky with near black veins; the costal region and the distal tip of the discoidal region more opaque with black pigment; the terminus of the discoidal region projecting well beyond the distal margin of anal region giving the appearance of an elongate wing.

*Abdomen*: Elongate for males and broad for females. Tergites without posterolateral projections. Cerci cylindrical, long and setose, tapering to a point. Supra-anal plate highly to slightly transverse between the sexes. Subgenital plate of male elongate, tapering to the terminus with two prominent styli.

*Genital Complex*: The main body of ventral left sclerite (L4A) with a smooth, rounded terminus, the left side with a broad indentation. The apofisis falloid (afa) of the main body of dorsal left sclerite (L4B) well sclerotized forming a smooth, rounded terminus in the shape of a broad, dull ninety degree hook; the apical process (paa) heavily sclerotized and curved, tapering to a smooth, rounded terminus that is heavily sclerotized. The right dorsal phallomere (fda) of the first sclerite of right phallomere (R1) tapers to a narrow, rounded terminus with short, dispersed setae; the ventral plate (pia) strongly sclerotized proximally, the surface rough and with broad curved grooves; the ventral process (pva) c-shaped, the distal end with rough, micro-toothed surface on the inside edge of the inward curve.

##### Ootheca.

Unknown for the genus.

##### Etymology.

A substantivated adjective, the name is derived from the Latin noun "velox" meaning swift or rapid, an apt name for their amazing speed when running across tree trunks.

#### Velox wielandi sp. n.

http://zoobank.org/2D67D4B9-682C-4A10-A7D6-870398FE9670

http://species-id.net/wiki/Velox_wielandi

##### Type.

Holotype Male, pinned. Biozentrum Grindel und Zoologisches Museum, Universität Hamburg, Germany.

##### Type locality.

Brazil: Espirito Santo (Brasil.), J. Michaelis vend., 22.IV.1898 (Lat. -19.995135, Long. -40.496412).

##### Material examined.

*Velox wielandi* sp. n.

**Table:** 

Sex	Type	Country	Label	Latitude Longitude	Code
Male	Holotype	Brazil	Espirito Santo, J. Michaelis vend., 22.IV.1898.	-19.995135, -40.496412	ZMUH 015
Female	Allotype	Brazil	Espirito Santo, J. Michaelis vend., 22.IV.1898.	-19.995135, -40.496412	ZMUH 002
Female	Paratype	Brazil			BMNH 011
Female	Paratype	Brazil			OUMNH 011
Female	Paratype	Brazil			OUMNH 012
Female	Paratype	Brazil	Bahia	-11.440584, -41.301279	ZMHB 007

##### Diagnosis.

Large species with an elongate habitus. Distal terminus of the forewing tapered, giving a sharp appearance; often asymmetrically colored with one being much darker. Hindwings are elongate with the distal margin of the discoidal region projecting well beyond the anal region. Tubercles present across the pronotum, but meso and metafemora smooth on the posterior (upper) surface. The posteroventral spines of the prothoracic femora very long, the distal the largest of the four. Species similar in general appearance to *Hagiomantis mesopoda*, but smaller and distributed in eastern Brazil.

##### Description.

**Figure 35.**
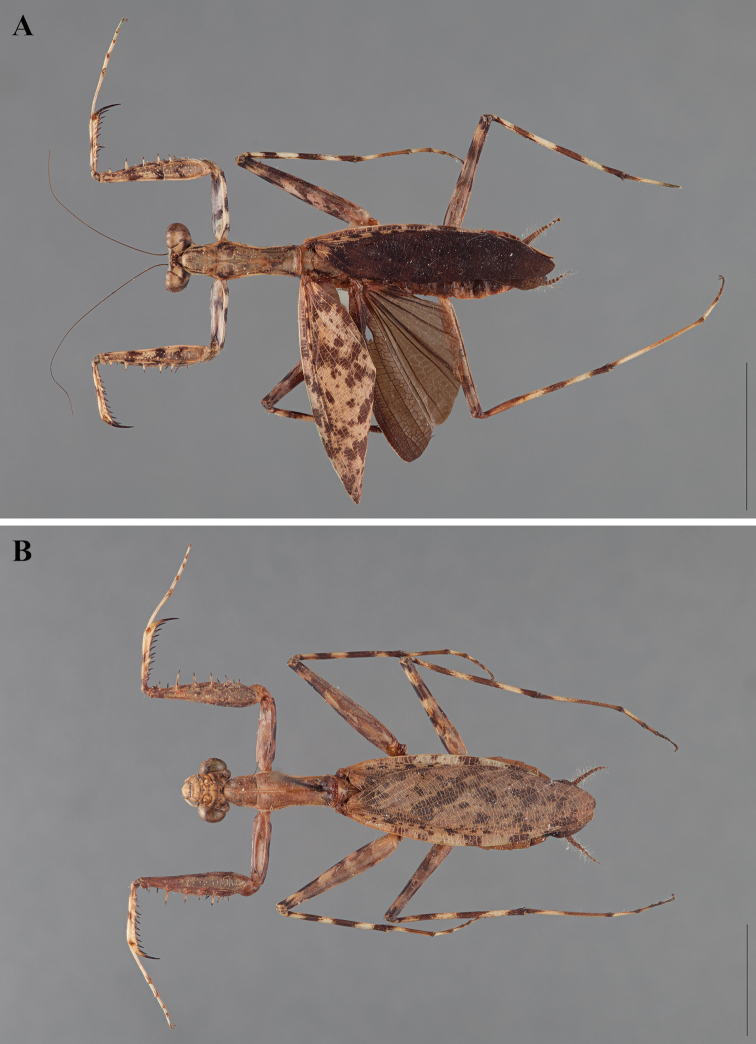
*Velox wielandi* sp. n., dorsal habitus: **A** holotype male from Espirito Santo, Brazil (ZMUH 015) **B** allotype female from Espirito Santo, Brazil (ZMUH 002).

**Male.** (Fig. 35A) N=1: Body length 26.25; forewing length 17.01; hindwing length 12.46; pronotum length 8.10; prozone length 2.37; pronotum width 2.67; pronotum narrow width 1.87; head width 4.80; head vertex to clypeus 1.91; frons width 1.62; frons height 0.64; prothoracic femur length 7.55; mesothoracic femur length 9.98; mesothoracic tibia length 7.65; mesothoracic tarsus length 7.86; metathoracic femur length 9.00; metathoracic tibia length 9.45; metathoracic tarsus length 10.65; pronotal elongation measure 0.29; pronotal shape measure 0.33; head shape measure 0.40; frons shape measure 0.40; anteroventral femoral spine count 16; anteroventral tibial spine count 10; posteroventral tibial spine count 8.

*Head* (Fig. 46C): Juxta-ocular protuberances present, the lateral third being the most pronounced; the vertex between the parietal sutures is straight or barely concave; vertex just lower than the dorsal margin of the eyes. Frontal suture with a faint curved carina forming a continuous arc. Ocelli small with the central ocellus oriented anteriorly while the lateral are oriented anterolaterally at the 45 degree angle from the central axis of the head; protruding slightly on cuticular mounds. Frons narrowed between the antennal insertion sites and depressed below the central ocellus; a transverse carina present below the central ocellus, running from lateral margins under the antennal insertion sites medially in a dorsally oriented curve, the middle forming an angle. Upper margin of clypeus straight, lower margin concave; a central protruding ridge strongly defined; the lateral margins tapering, widest at the upper margin. Antennae pale proximally and fading to light brown distally. Varying levels of black markings across the anterior surface of head. Palpi are pale.

*Pronotum* (Fig. 50G): Elongate with a slightly defined supra-coxal bulge; dorsal surface with tubercles; prozone long with parallel lateral margins prior to a rounded anterior margin; metazone long with sweeping concave lateral margins that exhibit small, occasional denticles; posterior margin of the metazone straight with a slight medial emargination; the dorsal surface of the posterior half of the metazone depressed. Colored with black and pale markings that vary across specimens.

*Prothoracic Legs*: Femur robust with a straight dorsal margin distally, the proximal third slightly convex. Pale to dark banding on posterior surface of femur with numerous tubercles; anterior (inner) surface amber colored ventrally, a black strip medially, and pale along the dorsal margin; ventral surface amber colored. Well developed femoral pit on the ventral surface to accommodate terminal posteroventral tibial spine positioned on the lateral margin, pushing the margin outward between the proximal two posteroventral spines; pit is pale. Posterior prothoracic femoral genicular spine at most half the length of the posteroventral spines, originating proximal to the beginning of the genicular lobe. Prothoracic posteroventral tibial spines with the first and second most proximal and fourth through seventh shorter than the proximal third and terminal spines; the anteroventral spines longest at distal end and shortening proximally. Posterior surface of the prothoracic tibiae smooth and banded with pale and dark coloration; anterior and ventral surface amber colored. Prothoracic coxae smooth with varying black markings on the posterior and ventral surface, the anterior surface mostly black with the ventral margin pale.

*Meso- and Metathoracic Legs*: Femora with pronounced dorsal and ventral carinae; posterior (upper) surface smooth. Tibiae with a pronounced ventral carina. Mesotarsi with first segment as long as the remaining segments combined. Metatarsi with first segment longer than remaining segments combined.

*Wings*: Fully developed, the same length or slightly longer than the abdomen. Forewings mottled with contrasting regions of brown, white, and black; the costal region alternating irregularly from pale to dark its entire length; the costal region widened. The forewings may be colored asymmetrically, one being mottled as described above while the other is much darker. Hindwings opaque and smoky with near black veins; the costal region and the distal tip of the discoidal region more opaque with black pigment; the terminus of the discoidal region projecting well beyond the distal margin of anal region, the wing appearing elongate.

*Abdomen*: Elongate, tubular, and smooth. Tergites without posterolateral projections. Supra-anal plate highly transverse with a blunt, straight terminus. Subgenital plate elongate, tapering to the terminus with two prominent styli.

*Genital Complex* (Fig. 53D.1): The main body of ventral left sclerite (L4A) with a smooth, rounded terminus, the left side with a broad indentation. The apofisis falloid (afa) of the main body of dorsal left sclerite (L4B) well sclerotized forming a smooth, rounded terminus in the shape of a broad, dull ninety degree hook; the apical process (paa) heavily sclerotized and curved, tapering to a smooth, rounded terminus. The right dorsal phallomere (fda) of the first sclerite of right phallomere (R1) tapers to a narrow, rounded terminus with short, dispersed setae; the ventral plate (pia) strongly sclerotized proximally, the surface rough and with broad curved grooves; the ventral process (pva) c-shaped, the distal end with rough, micro-toothed surface on the inside edge of the inward curve.

**Female.** (Fig. 35B) N=4: Body length 29.56–34.07 (32.27); forewing length 18.93–23.00 (21.07); hindwing length 14.81–18.00 (16.06); pronotum length 7.59–10.19 (9.28); prozone length 2.36–2.95 (2.73); pronotum width 2.80–3.56 (3.25); pronotum narrow width 2.06–2.50 (2.27); head width 5.55–6.09 (5.79); head vertex to clypeus 2.16–2.78 (2.47); frons width 1.86–2.30 (2.11); frons height 0.68–0.95 (0.85); prothoracic femur length 7.88–9.77 (9.08); mesothoracic femur length 11.67–12.16 (11.91); mesothoracic tibia length 8.89–9.49 (9.19); mesothoracic tarsus length 8.30–8.97 (8.64); metathoracic femur length 10.19–10.75 (10.47); metathoracic tibia length 11.43–12.00 (11.71); metathoracic tarsus length 11.11–13.96 (12.53); pronotal elongation measure 0.28–0.31 (0.29); pronotal shape measure 0.33–0.37 (0.35); head shape measure 0.39–0.46 (0.42); frons shape measure 0.36–0.42 (0.40); anteroventral femoral spine count 16; anteroventral tibial spine count 10; posteroventral tibial spine count 8.

*Head* (Fig. 46D): Vertex slightly higher than the dorsal margin of the eyes. Upper margin of clypeus straight, lower margin straight. Antennae pale proximally and fading to dark brown distally.

*Pronotum* (Fig. 50H): Elongate with a slightly defined supra-coxal bulge; dorsal surface with tubercles; prozone long with parallel lateral margins prior to a rounded anterior margin; metazone long with slightly concave lateral margins, nearly straight medially; lateral margin with very small, occasional denticles; posterior margin of the metazone broadly rounded with a slight medial emargination.

*Prothoracic Legs*: Anterior (inner) surface of femur pale ventrally, a black strip medially, and pale along the dorsal margin; ventral surface pale. Prothoracic coxae smooth with varying black markings on the posterior and ventral surface, the anterior surface with a medial black marking in the proximal half.

*Meso- and Metathoracic Legs*: Mesotarsi with first segment shorter than the remaining segments combined. Metatarsi with first segment longer than remaining segments combined.

*Wings*: As described for males.

*Abdomen*: Broad, widening from first segment until the beginning of the distal half (segments 4–5) when the lateral margins narrow gradually to the terminus, the middle being the broadest region. Supra-anal plate slightly transverse with a dull, pointed terminus, the margins tapering distally.

##### Etymology.

A noun in the genitive case, *Velox wielandi* is named for Frank Wieland, whose contributions to Mantodea systematics, phylogeny and morphology have advanced the field considerably.

#### Corticomantis gen. n.

http://zoobank.org/7C91CFD5-5826-4E17-9224-B9E6B16CC970

http://species-id.net/wiki/Corticomantis

Liturgusa (*partim*): Beier 1931: 14–15; Beier 1935: 11; La Greca 1939: 5; Weidner 1964: 143; Terra 1995: 53; Jantsch 1999: 48; Ehrmann 2002: 206; Otte and Spearman 2005: 132; Agudelo et al. 2007: 116.
Liturgousa (*partim*): Rehn 1935: 204.

##### Type species.

*Liturgusa atricoxata* Beier, 1931

##### Description.

*Habitus*: Small and squat, wide body, the genus *Corticomantis* exhibits striking, contrasting coloration including dark brown, black and varying shades of green that resembles a bark-lichen surface. Dorso-ventrally flattened with moderately long legs.

*Measurement Ranges*: Monotypic genus, see *Corticomantis atricoxata* for measurement ranges.

*Head*: Transverse with large, rounded eyes projecting outside the profile of the head both laterally and anteriorly (the anterior margin of the eyes anterior to the central surface of the head). Juxta-ocular protuberances present, small in males and well developed in females. The vertex between the parietal sutures is straight. Frontal suture with a faint curved carina. Ocelli small in males and females with the central ocellus oriented anteriorly while the lateral are oriented anteriorly at a 45 degree angle from the central axis of the head; protruding on a cuticular mounds or on a prominent continuous curved carina. Frons narrowed between the antennal insertion sites and depressed below the central ocellus; a transverse carina present below the central ocellus, running from lateral margins under the antennal insertion sites medially in a dorsally oriented curve. Upper margin of clypeus convex, lower margin straight; a central protruding carina; the lateral margins tapering, widest at the upper margin. Labrum with minimal sculpting and a rounded terminus. Antennae filiform and with rare setae, pale proximally and fading to black distally. Varying levels of black markings across the anterior surface of head, the vertex and juxta-ocular protuberances speckled. Palpi are pale.

*Pronotum*: Short and broad with a less defined supra-coxal bulge, the metazone being wide and the lateral margins of the metazone nearly parallel before tapering posteriorly; dorsal surface with tubercles of varying size and density, but obviously prominent. Broad prozone with lateral margins that taper anteriorly, the anterior margin rounded; a central depression medially on the dorsal surface. Metazone with lateral margins that are nearly parallel anteriorly, but taper dramatically to the narrowest point (the midpoint of the metazone) before becoming parallel anterior to the posterior terminus; the corners of the posterior margin rounded with the medial region straight; posterior region with two blunt protrusions on each side of the medial line. Supra-coxal sulcus strongly defined. Colored with black and pale speckling. The lateral margins of the pronotum slightly expanded to form a ledge.

*Prothoracic Legs*: Femoral spine count of males and females: anteroventral 15–16, posteroventral 4, discoidal 4. Femur robust with a slightly concave dorsal margin distally, the proximal half convex, larger in females; anteroventral and posteroventral (internal and external, respectively) spines well developed; line of small tubercles running medially of the posteroventral spines. A continuous carina running from distal terminus of femur along dorsal margin to the base, circling the posterior (external) surface of the proximal end and running along the ventral margin at the base of the posteroventral spines. Pale to dark banding on posterior surface of femur, sometimes not well defined and degenerating into pale and dark speckling ventrally; internal surface mostly black, but with pale regions dorsally; ventral surface black and pale. Posterior surface of femur smooth or with few tubercles. Well developed femoral pit on the ventral surface to accommodate terminal posteroventral tibial spine positioned between the most proximal posteroventral spine of femur and the most distal discoidal spine, pit is colored black. Prothoracic tibial spine count of males and females: anteroventral 9–10, posteroventral 8. Prothoracic tibial spines robust; the posteroventral spines with the first and second most proximal and fifth through seventh shorter than the much longer proximal third, fourth, and terminal spines; the anteroventral spines longest at distal end and shortening proximally. Prothoracic tibiae with a smooth posterior surface. Tarsi banded with pale and dark coloration. Prothoracic coxae smooth with no or a few very minor tubercles or setae along anterior margin; black markings vary.

*Meso- and Metathoracic Legs*: Long and slender with pale to dark banding on the femur and tibia. Femora with a pronounced dorsal and ventral (posterior) carina; posterior (upper) surface with two carina. Tibia widening from the proximal terminus and with multiple, faint carina. Mesotarsi with first segment shorter than remaining segments combined. Metatarsi with first segment the same length as the remaining segments combined.

*Wings*: Fully developed in males and females. Forewings mottled with contrasting regions of brown, white, green, and black; the proximal quarter dark, then fading dramatically to a mottled white with a darkened spot on and around the pterostigma; the distal quarter of the wing mottled irregularly with half greenish white and half brownish black; the costal vein alternating from pale to dark its entire length while the costal region is mostly pale with banding in the distal quarter, but can be banded throughout its length; the costal region wide relative to the wing length. The forewings may be colored asymmetrically, one being mottled as described above while the other is blackened. Hindwings opaque and smoky; the terminus of the discoidal region projecting to beyond the distal margin of anal region.

*Abdomen*: Males and females with widening abdomen from first segment until the beginning of the distal half (segments 5–6) when the lateral margins narrow to the terminus, the middle being the broadest region. Tergites with pointed posterolateral projections in the distal half of the abdomen of females. Cerci cylindrical, long and setose, tapering to a point. Supra-anal plate of female as broad as wide with a blunt terminus, large in size; of male transverse with blunt terminus. Subgenital plate of male with rounded, slightly irregular terminus; without styli.

*Male Genital Complex*: The only known species for the genus exhibits dextral genitalia (‘reversed’ genitalia in Balderson 1978 and Holwell and Herberstein (2010); defined as a genital complex in which the apical process (paa) of L4B is directed to the right) while most Mantodea exhibit sinistral oriented genitalia (e.g. all species of *Liturgusa* and *Fuga*). Although *Corticomantis atricoxata* exhibits this orientation, any future new species could exhibit sinistral orientation and the genus should not be defined by dextral genital orientation. Holwell and Herberstein (2010) demonstrated that some species of *Ciulfina* exhibit both orientations, thus could be variable within *Corticomantis* species as well. The main body of ventral left sclerite (L4A) with a prominent, heavily sclerotized distal process (pda). The apofisis falloid (afa) of the main body of dorsal left sclerite (L4B) well sclerotized with a blunt, rounded terminus, and long setae emerging laterally; the apical process (paa) heavily sclerotized and curved, ending with a rounded terminus. The right dorsal phallomere (fda) of the first sclerite of right phallomere (R1) tapers to a rounded terminus; the ventral plate (pia) strongly sclerotized; the ventral process (pva) strongly sclerotized.

##### Ootheca.

Unknown for the genus.

##### Etymology.

A compound word formed from two components, “corticis” and “mantis”. In the feminine, *Corticomantis* translates to “bark mantis”.

#### Corticomantis atricoxata

(Beier, 1931)

http://species-id.net/wiki/Corticomantis_atricoxata

Liturgusa atricoxata : Beier 1931: 14–15; Beier 1935: 11; La Greca 1939: 5; Weidner 1964: 143; Terra 1995: 53; Jantsch 1999: 48; Ehrmann 2002: 206; Otte and Spearman 2005: 132; Agudelo et al. 2007: 116.
Liturgousa atricoxata : Rehn 1935: 204.

##### Type.

Holotype Female. Biozentrum Grindel und Zoologisches Museum, Universität Hamburg, Germany.

##### Type locality.

Costa Rica. Limon Plain at Las Mercedes, Hamburg Farm on the Reventazon, 10-30 meters above the sea, 12-30 kilometers from the Atlantic, August 15, 1923, F. Nevermann collector. (Lat. 10.250000, Long. -83.450000).

##### Material examined.

*Corticomantis atricoxata* (Beier, 1931).

**Table:** 

Sex	Type	Country	Label	Latitude Longitude	Code
Female	Holotype	Costa Rica	Limon Plain at Las Mercedes, Hamburg Farm on the Reventazon, 10-30 meters above the sea, 12-30 kilometers from the Atlantic, August 15, 1923, F. Nevermann collector.	10.250000, -83.450000	ZMUH
Female	nontype				OUMNH 015
Female	nontype	Costa Rica	Puerto Viejo, Meredia Prov., VI.25.1965, Collected by D. H. Janzen	10.458492, -84.006706	USNM 003; USNM ENT 00873032
Female	nontype	Costa Rica	Turrialba, 5 June 1951, OL Cartwright	9.904458, -83.688949	USNM 025; USNM ENT 00873033
Male	nontype	Costa Rica	Turrialba, 5 June 1951, OL Cartwright	9.904458, -83.688949	USNM 026; USNM ENT 00873034
Female	nontype	Costa Rica	Turrialba, 4 June 1951, OL Cartwright	9.904458, -83.688949	USNM 027; USNM ENT 00873035
Male	nontype	Costa Rica	Turrialba, 5 June 1951, OL Cartwright	9.904458, -83.688949	USNM 028; USNM ENT 00873036
Female	nontype	Costa Rica	Turrialba, 22 June 1951, OL Cartwright	9.904458, -83.688949	USNM 029; USNM ENT 00873037
Male	nontype	Costa Rica	Turrialba, 13 June 1951, OL Cartwright	9.904458, -83.688949	USNM 030; USNM ENT 00873038

##### Taxonomic history.

First described by Max Beier, the species was referenced only a few times over the next 80 years and never treated in a comprehensive taxonomic study. However, Rehn (1935) suggested that the species did not really belong within *Liturgusa* based on its considerable differences in general appearance. Examining the male genitalia and redescribing the genus allowed description of numerous characters that distinguishes the species from all other Neotropical Liturgusini. More than one species likely exists, but only *Corticomantis atricoxata* is known; two larger females from Colombia have been examined but are not distinct other than size, but the poor condition of the specimens limits their evaluation. No males are known from Colombia, which makes species assessment with genitalia impossible.

##### Diagnosis.

A darkened, mottled appearance. Overall a squat species with robust legs and a short, broad pronotum and abdomen. The wings are marked with highly contrasting white or greenish and nearly black or dark brown markings. The lateral margins of the abdomen visible in females when the wings are folded over the abdomen.

##### Description.

**Figure 36.**
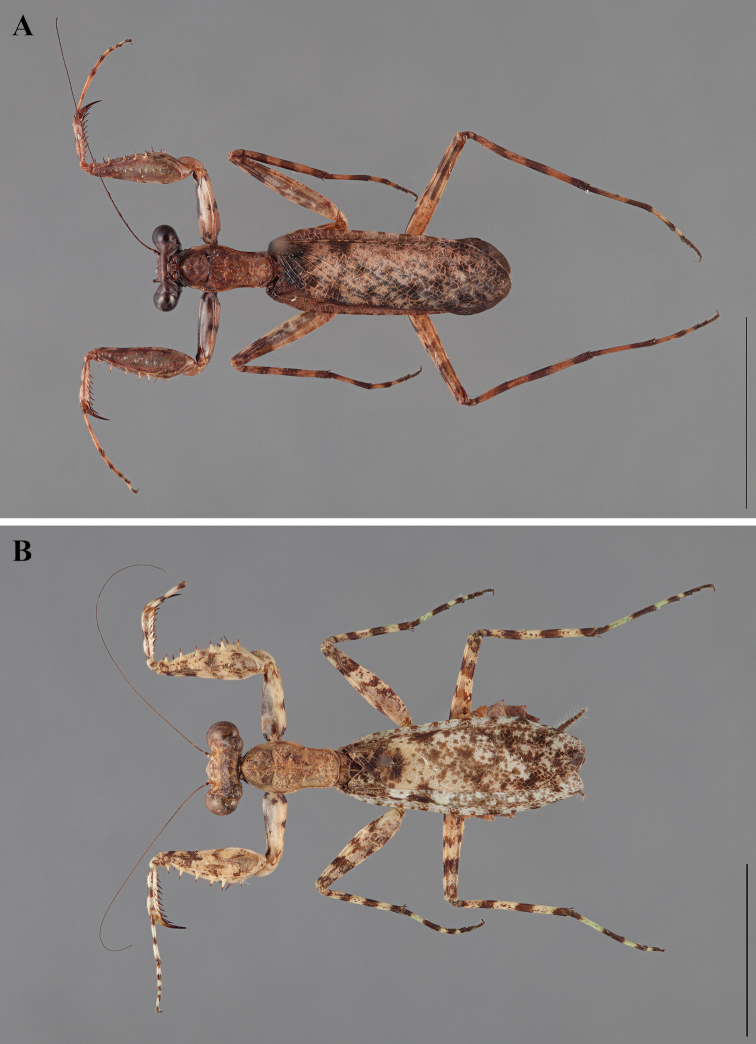
*Corticomantis atricoxata* (Beier, 1931), dorsal habitus: **A** male from Costa Rica (USNM 026) **B** female from Costa Rica (USNM 003).

**Male.** (Fig. 36A) N=3: Body length 18.68–19.64 (19.19); forewing length 13.04–13.42 (13.23); pronotum length 4.78–5.05 (4.91); prozone length 1.49–1.60 (1.56); pronotum width 2.50–2.56 (2.53); pronotum narrow width 1.88–1.90 (1.89); head width 4.60–4.70 (4.65); head vertex to clypeus 1.75–1.84 (1.78); frons width 1.60–1.65 (1.61); frons height 0.56–0.64 (0.60); prothoracic femur length 5.43–5.49 (5.46); mesothoracic femur length 6.56–6.63 (6.60); mesothoracic tibia length 4.92–5.05 (5.00); mesothoracic tarsus length 5.24–5.52 (5.38); metathoracic femur length 6.32–6.96 (6.71); metathoracic tibia length 6.94–7.06 (6.99); metathoracic tarsus length 7.35–7.65 (7.50); pronotal elongation measure 0.31–0.33 (0.32); pronotal shape measure 0.51–0.53 (0.52); head shape measure 0.38–0.39 (0.38); frons shape measure 0.35–0.39 (0.38); anteroventral femoral spine count 16; anteroventral tibial spine count 9; posteroventral tibial spine count 8.

*Head* (Fig. 46E): Juxta-ocular protuberances small; the vertex between the parietal sutures is straight; vertex lower than the dorsal margin of the eyes. Frontal suture with a faint curved carina. Ocelli small on protruding cuticular mounds. Head is dark brown with fine black speckling across the vertex and juxta-ocular protuberances, the frons, and parts of the clypeus.

*Pronotum* (Fig. 50I): Short and broad with a less defined supra-coxal bulge; dorsal surface with tubercles of varying size and density. Broad prozone with lateral margins that taper anteriorly, the anterior margin rounded; a central depression medially on the dorsal surface. Metazone with lateral margins that are nearly parallel anteriorly, but taper dramatically to the narrowest point (the midpoint of the metazone) before becoming parallel anterior to the posterior terminus; the posterior margin rounded overall with the medial region being straight; posterior region with two blunt protrusions on each side of the medial line; the dorsal surface of the posterior half of the metazone depressed. Mostly pale in coloration with black speckling; two dark marks laterally on the posterior surface of the metazone.

*Prothoracic Legs*: Femur robust with a slightly concave dorsal margin distally, the proximal half slightly convex. Pale to dark banding on posterior (external) surface of femur, sometimes not well defined and degenerating into pale and dark speckling ventrally; anterior (internal) surface mostly black, but with pale regions dorsally; ventral surface mostly black with a pale margin just inside the posteroventral spines. Posterior prothoracic femoral genicular spine nearly the same length as the posteroventral spines, originating distal to the beginning of the genicular lobe. Prothoracic tibia with pale and dark banding on the anterior and posterior surface, the ventral surface mostly pale. Prothoracic coxae black anteriorly (internal side) with a small pale marking in the proximal half along the dorsal margin.

*Meso- and Metathoracic Legs*: Femora with a pronounced dorsal and ventral (posterior) carina; posterior (upper) surface with two carinae, one running the nearly entire length, fading proximally and positioned ventrally to the dorsal carina, the other in the distal third and positioned parallel and between the previous and the ventral carina. Mesotarsi with first segment shorter than remaining segments combined. Metatarsi with first segment the same length as the remaining segments combined.

*Wings*: Forewings mottled with contrasting regions of brown, white, green, and black; the proximal quarter dark, then fading dramatically to a mottled white with a darkened spot on and around the pterostigma; the distal quarter of the wing mottled irregularly with half greenish white and half brownish black; the costal vein alternating from pale to dark its entire length while the costal region is mostly pale with banding in the distal quarter, but can be banded throughout its length; the costal region wide relative to the wing length, the width between 6.5–7.5% the total wing length. The forewings may be colored asymmetrically, one being mottled as described above while the other is blackened. Hindwings opaque and smoky; the terminus of the discoidal region projecting slightly beyond the distal margin of anal region, almost fitting within the outer margin of the wing.

*Abdomen*: Widening from first segment until the beginning of the distal half (segments 5–6) when the lateral margins narrow to the terminus, the middle being the broadest region. Tergites without pointed posterolateral projections. Supra-anal plate transverse with a blunt terminus. Subgenital plate of male with a rounded, slightly irregular terminus; without styli.

*Genital Complex* (Fig. 53E.1–E.3): Dextral genitalia with the main body of ventral left sclerite (L4A) with a prominent, heavily sclerotized distal process (pda) that forms a half circle, the terminus with a posterior orientation (Fig. 53, E.1 folded out and incorrectly position due to slide mounting). The apofisis falloid (afa) of the main body of dorsal left sclerite (L4B) well sclerotized with a smooth, blunt, rounded terminus, and long, robust setae of varying length emerging from the lateral margin of the afa; the apical process (paa) heavily sclerotized and curved, including a medial bump before ending with a smooth and rounded terminus. The right dorsal phallomere (fda) of the first sclerite of right phallomere (R1) tapers to a rounded terminus and is mostly membranous with disperse fine setae; the ventral plate (pia) strongly sclerotized and small with a blunt knob nearest to the ventral process (pva); the ventral process (pva) strongly sclerotized and small with an irregular shape, almost resembling a tooth.

##### Redescription.

**Figure 37.**
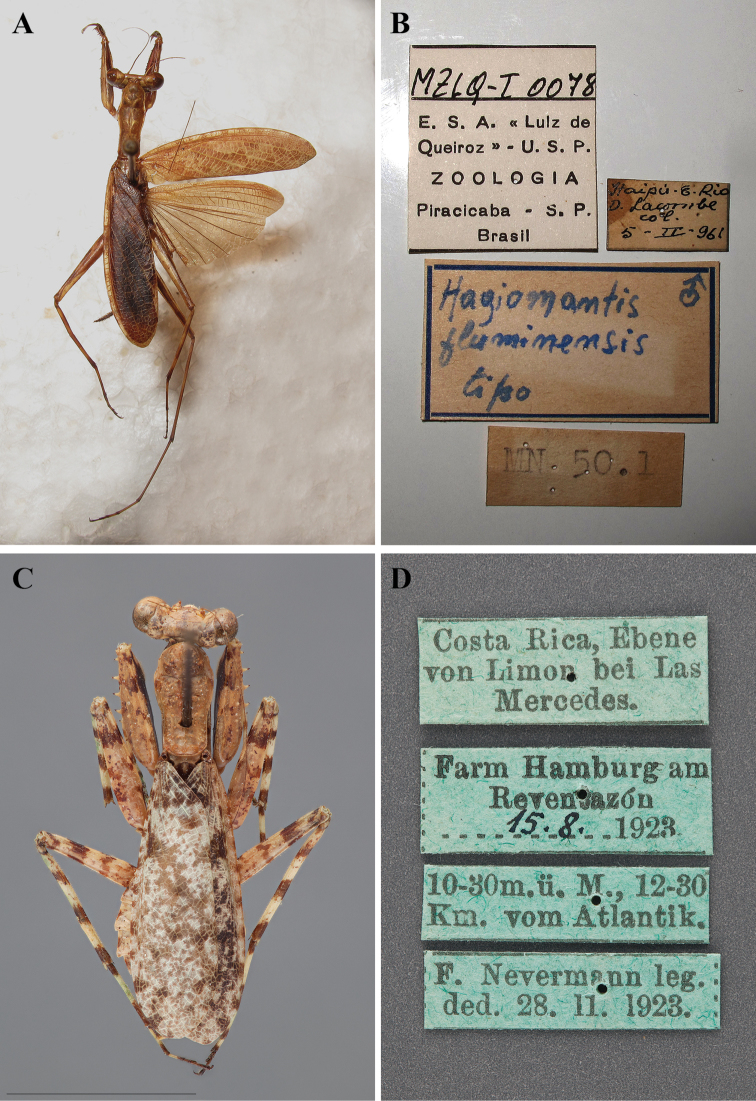
*Fuga fluminensis* (Piza, 1965), and *Corticomantis atricoxata* (Beier, 1931), dorsal habitus of types and labels. *Fuga fluminensis* (Piza, 1965): **A** holotype female (DZES) **B** labels. *Corticomantis atricoxata* (Beier, 1931): **C** holotype female from Costa Rica (ZMUH) **D** labels.

**Female.** (Figs 36B, 37C) N=6: Body length 21.41–24.72 (22.95); forewing length 14.02–18.38 (15.63); hindwing length 12.41–15.10 (13.95); pronotum length 5.69–6.55 (6.05); prozone length 1.85–2.15 (1.96); pronotum width 3.09–3.34 (3.20); pronotum narrow width 2.29–2.44 (2.36); head width 5.52–5.91 (5.70); head vertex to clypeus 2.24–2.36 (2.30); frons width 1.98–2.24 (2.12); frons height 0.67–0.84 (0.78); prothoracic femur length 6.32–7.86 (6.95); mesothoracic femur length 6.51–7.40 (7.11); mesothoracic tibia length 5.25–5.69 (5.53); mesothoracic tarsus length 5.26–5.85 (5.62); metathoracic femur length 6.77–8.28 (7.55); metathoracic tibia length 7.23–8.85 (7.92); metathoracic tarsus length 7.42–8.90 (8.19); pronotal elongation measure 0.31–0.35 (0.32); pronotal shape measure 0.51–0.55 (0.53); head shape measure 0.39–0.41 (0.40); frons shape measure 0.30–0.42 (0.37); anteroventral femoral spine count 15–16 (16); anteroventral tibial spine count 9–10 (9); posteroventral tibial spine count 8.

*Head* (Fig. 46F): Juxta-ocular protuberances large and pronounced, the vertex between the parietal sutures is straight; vertex higher than the dorsal margin of the eyes. Frontal suture with a pronounced curved carina. Ocelli small and positioned laterally on a prominent, continuous curved carina. Head is brown with fine black speckling across the vertex and more dense on the juxta-ocular protuberances; frons and parts of the clypeus exhibit small black markings as well.

*Pronotum* (Fig. 50J): As described for males.

*Prothoracic Legs*: Posterior prothoracic femoral genicular spine slightly smaller than the posteroventral spines, originating distal to the genicular lobe.

*Meso- and Metathoracic Legs*: As described for males.

*Wings*: As described for males.

*Abdomen*: As described for males.

#### Hagiomantis mesopoda

(Westwood, 1889)

http://species-id.net/wiki/Hagiomantis_mesopoda

Liturgousa mesopoda : Westwood 1889: 5, 30, 51, pl. 13, fig. 10; Kirby 1904: 271; Chopard 1911: 323; Hebard 1919b: 134.
Liturgusa mesopoda : Apolinar M. 1924: 47; Giglio-Tos 1927: 294; Beier 1935: 11, pl. 3, fig. 7; Apolinar M. 1937: 226; Terra 1995: 54; Jantsch 1999: 48; Salazar E. 1999: 10; Salazar E. 2002: 124; Ehrmann 2002: 207; Otte and Spearman 2005: 133; Agudelo et al. 2007: 116, 142.
Liturgusa mesapoda : Agudelo 2004: 55, Table 3.1; Agudelo 2005: 3.

##### Type.

Holotype Female. Oxford University Museum of Natural History, Oxford, United Kingdom.

##### Type locality.

French Guiana: Guiana Francesca, St. Laurent de Maroni (Depuiset) (Lat. 5.487038, Long. -54.008462).

##### Material examined.

*Hagiomantis mesopoda* (Westwood, 1889).

**Table:** 

Sex	Type	Country	Label	Latitude Longitude	Code
Female	Holotype	French Guiana	St. Laurent du Maroni (Depuiset)	5.487038, -54.008462	OUMNH
Male	nontype	French Guiana	Saut Aiuara, 8-VIII-1994, P. Peters		MNHN 029
Female	nontype	French Guiana	St-Laurent du Maroni, Collection Le Moult	5.487038, -54.008462	MNHN 030
Female	nontype	French Guiana	Pied Saut Parare, IX-1977, M. Descamps Rec.	4.046724, -52.698087	MNHN 031
Female	nontype	French Guiana	Nouveau Chantier, collection Le Moult, Coll. L. Chopard, 1919, Novembre		MNHN 032

##### Taxonomic history.

One of the earlier specimens described (Westwood 1889), *Hagiomantis mesopoda* has always been included within *Liturgusa*. However, the examination of the holotype while treating the broader diversity of *Liturgusa* has revealed the species actually fits within the closely related genus *Hagiomantis* Saussure & Zehntner, 1894. Although the species is moved into *Hagiomantis*, the broader genus is not treated within this study, but *Hagiomantis mesopoda* is redescribed herein. Two type specimens labeled as *Liturgusa mesopoda* from the Oxford University Museum of Natural History (OUMNH Type 452 2/3 and 3/3) were also examined and found to be *Liturgusa cayennensis*. The history of these two specimens is not known, but they should not be considered as types of *Hagiomantis mesopoda* and may have been mislabeled at some point in the past. Further, Westwood (1889: 30) references only the holotype from St. Laurent de Maroni, French Guiana, and not any additional type material.

##### Diagnosis.

Large species with a dark dorsal habitus, the coloration with highly contrasting light and dark mottling on the forewings. Numerous tubercles present on the posterior (external) surface of the prothoracic femora, the mesofemora, and the metafemora.

##### Description.

**Figure 38.**
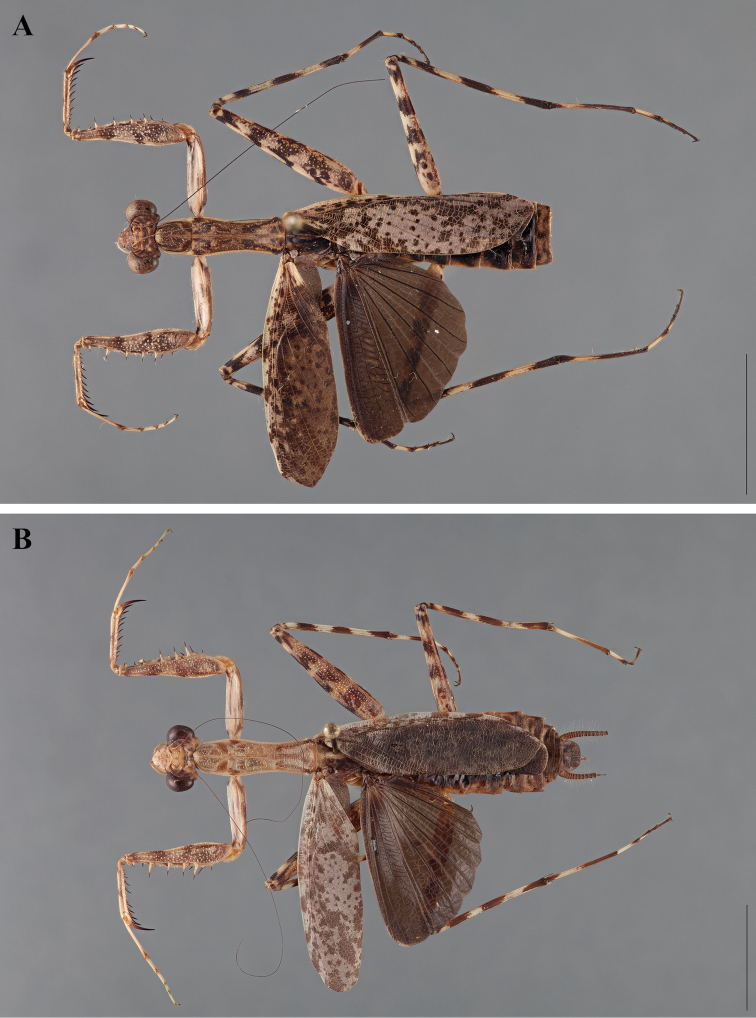
*Hagiomantis mesopoda* (Westwood, 1889), dorsal habitus: **A** male from French Guiana (MNHN 029) **B** female from French Guiana (MNHN 031).

**Male.** (Fig. 38A) N=1: Body length 31.77; forewing length 17.12; hindwing length 13.71; pronotum length 9.15; prozone length 2.60; pronotum width 2.76; pronotum narrow width 2.07; head width 5.33; head vertex to clypeus 2.03; frons width 1.80; frons height 0.74; prothoracic femur length 8.30; mesothoracic femur length 11.63; mesothoracic tibia length 9.67; mesothoracic tarsus length 7.86; metathoracic femur length 10.18; metathoracic tibia length 11.26; metathoracic tarsus length 11.21; pronotal elongation measure 0.28; pronotal shape measure 0.30; head shape measure 0.38; frons shape measure 0.41; anteroventral femoral spine count 15; anteroventral tibial spine count 10; posteroventral tibial spine count 8.

*Head* (Fig. 46G): Transverse with large, rounded eyes projecting outside the profile of the head both laterally and anteriorly (the anterior margin of the eyes anterior to the central surface of the head). Juxta-ocular protuberances present, the lateral third being the most pronounced; the vertex between the parietal sutures is slightly concave; vertex lower than the dorsal margin of the eyes. Frontal suture with a faint curved carina, forming sharp angle medially. Two slightly protruding mounds are located symmetrically between the lateral sections of the frontal suture and the parietal sutures. Ocelli small with the central ocellus oriented anteriorly while the lateral are oriented anterolaterally at the 45 degree angle from the central axis of the head; protruding slightly on cuticular mounds. Frons narrowed between the antennal insertion sites and depressed below the central ocellus; a transverse carina present below the central ocellus, running from lateral margins under the antennal insertion sites medially in a dorsally oriented curve. Upper margin of clypeus convex, lower margin slightly concave; a central protruding ridge; the lateral margins tapering, widest at the upper margin. Labrum with minimal sculpting and a rounded terminus; lateral margins widening ventrally and concave. Antennae filiform and with rare setae, pale proximally and fading to black distally. Varying levels of black markings across the anterior surface of head. Palpi are pale.

*Pronotum* (Fig. 50K): Elongate with a less defined supra-coxal bulge; dorsal surface with numerous tubercles; prozone long with parallel lateral margins prior to a rounded anterior margin; metazone long with sweeping concave lateral margins that exhibit small denticles; posterior margin of the metazone straight; the dorsal surface of the posterior half of the metazone depressed. Supra-coxal sulcus strongly defined. The lateral margins of the pronotum slightly expanded to form a small ledge. Colored with black and pale markings that vary across specimens.

*Prothoracic Legs*: Femur robust with a slightly concave dorsal margin distally, the proximal third convex; anteroventral and posteroventral spines well developed; line of small, pointed tubercles running medially of the posteroventral spines; anteroventral spines black, posteroventral spines pale basally with black terminus. A strongly pronounced continuous carina running from distal terminus of femur along dorsal margin to the base, circling the posterior (external) surface of the proximal end and running along the ventral margin at the base of the posteroventral spines. Pale to dark banding on posterior (external) surface of femur with numerous tubercles; anterior (inner) surface pale with femoral brush colored black; ventral surface pale. Well developed femoral pit on the ventral surface to accommodate terminal posteroventral tibial spine positioned on the lateral margin between the proximal two posteroventral spines, pushing the margin outward; pit is pale. Posterior prothoracic femoral genicular spine much smaller than posteroventral spines, originating distal to the beginning of the genicular lobe. Prothoracic tibial spines robust; the posteroventral spines with the first and second most proximal and fifth through seventh shorter than the slightly longer proximal third, fourth, and terminal spines; the anteroventral spines longest at distal end and shortening proximally. Prothoracic tibiae with a line of pronounced tubercles on the posterior surface, extending almost the entire length. Tarsi banded with pale and dark coloration. Prothoracic coxae smooth with varying black markings on the posterior and ventral surface, the anterior surface entirely pale.

*Meso- and Metathoracic Legs*: Long and slender with pale to dark banding on the femur and tibia. Femora with a pronounced ventral carina and a faint dorsal carina; posterior (upper) surface with pronounced tubercles. Tibiae banded with alternating pale and dark regions. Mesotarsi with first segment shorter than remaining segments combined. Metatarsi with first segment longer than remaining segments combined.

*Wings*: Fully developed, but shorter than the abdomen. Forewings mottled with contrasting regions of brown, white, and black; the costal region with alternating irregular banding from pale to dark its entire length; the costal region not expanded. The forewings may be colored asymmetrically, one being mottled as described above while the other is much darker. Hindwings opaque and smoky with near black veins; the terminus of the discoidal region blunt and not projecting beyond the distal margin of anal region, the wing appearing truncate.

*Abdomen*: Elongate, tubular, and smooth. Tergites with small, pointed posterolateral projections. Cerci cylindrical, long and setose, tapering to a point. Supra-anal plate broad with a blunt, straight terminus. Subgenital plate rounded, but with a broad medial depression, the bottom of which has a straight margin; without styli.

*Genital Complex* (Fig. 53F.1): The main body of ventral left sclerite (L4A) with a terminus that exhibits a heavily sclerotized region (75% percent of terminus) that ends abruptly on the left side with a angled, blunt nub. The apofisis falloid (afa) of the main body of dorsal left sclerite (L4B) well sclerotized with a very small, sharp terminus; the apical process (paa) heavily sclerotized and curved, ending with a smooth, rounded terminus that is heavily sclerotized. The right dorsal phallomere (fda) of the first sclerite of right phallomere (R1) tapers to a rounded terminus and is slightly sclerotized, without many setae; the ventral plate (pia) long and strongly sclerotized, extending nearly a third the entire length of R1, terminating with a rounded process oriented towards the ventral process (pva); the ventral process (pva) strongly sclerotized and small, tapering to a point.

##### Redescription.

**Figure 39.**
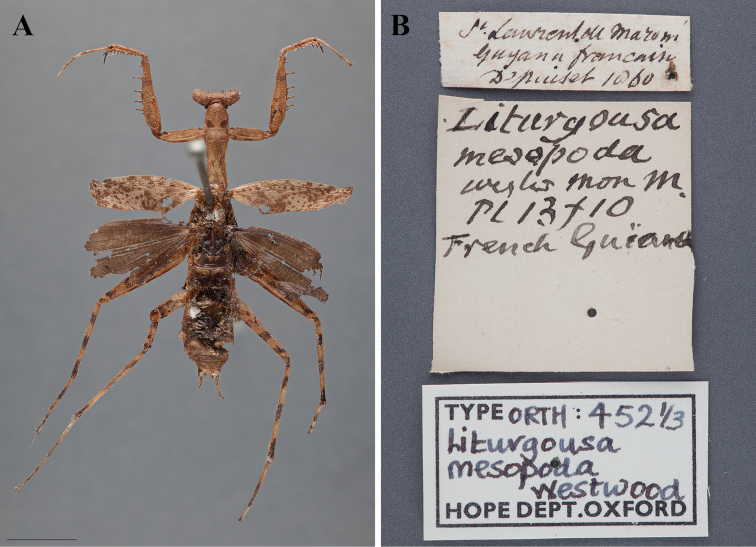
*Hagiomantis mesopoda* (Westwood, 1889) holotype female (OUMNH): **A** dorsal habitus **B** labels.

**Female.** (Figs 38B, 39A) N=2: Body length 37.52–38.07 (37.79); forewing length 19.89–21.19 (20.54); hindwing length 16.84; pronotum length 11.53–11.54 (11.54); prozone length 3.24–3.34 (3.29); pronotum width 3.39–3.50 (3.45); pronotum narrow width 2.46–2.65 (2.55); head width 6.26–6.53 (6.39); head vertex to clypeus 2.54–2.70 (2.63); frons width 2.26–2.32 (2.29); frons height 0.93–0.96 (0.94); prothoracic femur length 10.09–10.43 (10.26); mesothoracic femur length 13.51–13.69 (13.60); mesothoracic tibia length 11.03–11.08 (11.05); mesothoracic tarsus length 9.78–9.94 (9.86); metathoracic femur length 11.82–11.96 (11.89); metathoracic tibia length 12.52–13.44 (12.98); metathoracic tarsus length 13.41–13.56 (13.49); pronotal elongation measure 0.28–0.29 (0.28); pronotal shape measure 0.29–0.30 (0.30); head shape measure 0.41; frons shape measure 0.41; anteroventral femoral spine count 15; anteroventral tibial spine count 10; posteroventral tibial spine count 8.

*Head* (Fig. 46H): Juxta-ocular protuberances present, the middle third being the most pronounced; the vertex between the parietal sutures is slightly concave; vertex just lower than the dorsal margin of the eyes. Two protruding mounds are located symmetrically between the lateral region of the frontal suture and the parietal suture; region above the frontal suture between the two mounds depressed. A carina connecting all three ocelli that continues laterally half the distance to the margin of the eye. Palpi pale with a dark terminus.

*Pronotum* (Fig. 50L): Metazone with sharply rounded posterior corners caused by straightening of the lateral margins in the posterior terminus connecting to a straight posterior margin.

*Prothoracic Legs*: As described for males.

*Meso- and Metathoracic Legs*: As described for males.

*Wings*: As described for males.

*Abdomen*: Elongate, gradually widening until the third to last tergite where it narrows dramatically, that last three segments being compressed. Tergites with small, pointed posterolateral projections. Supra-anal plate nearly square with a blunt, straight terminus.

**Figure 40.**
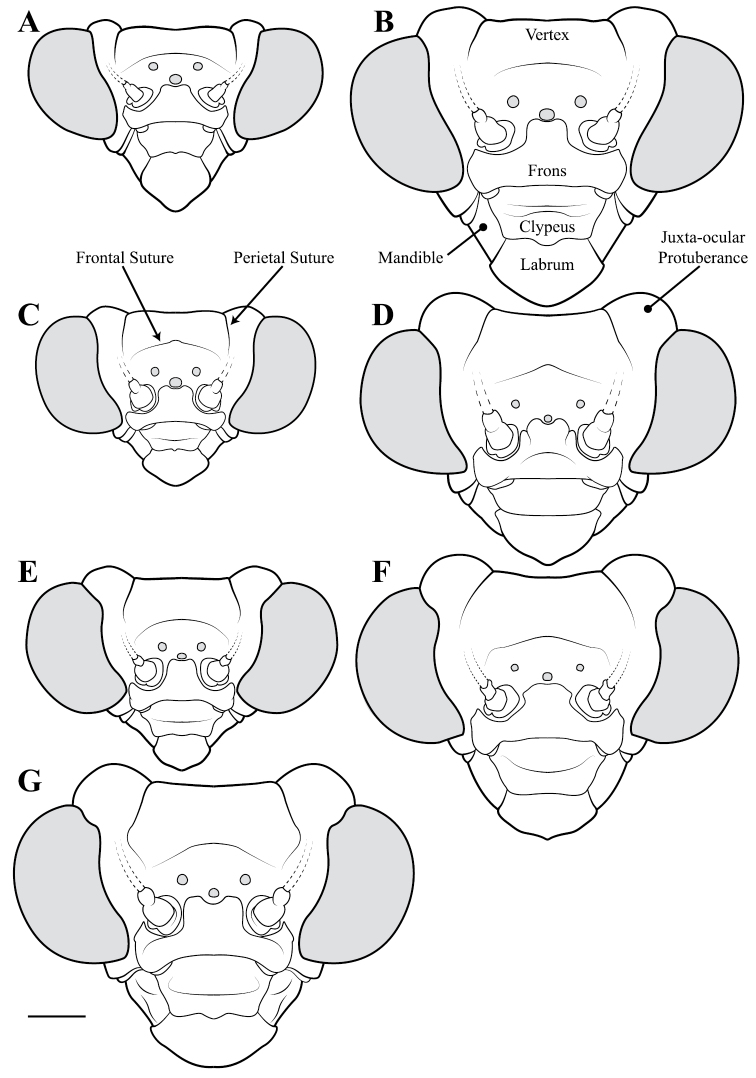
*Liturgusa*, head from the anterior perspective to scale (scale bar = 1 mm). *Liturgusa cayennensis* Saussure, 1869: **A** male **B** female. *Liturgusa lichenalis* Gerstaecker, 1889: **C** male **D** female. *Liturgusa guyanensis* La Greca, 1939: **E** male **F** female. *Liturgusa neblina* sp. n.: **G** female.

**Figure 41.**
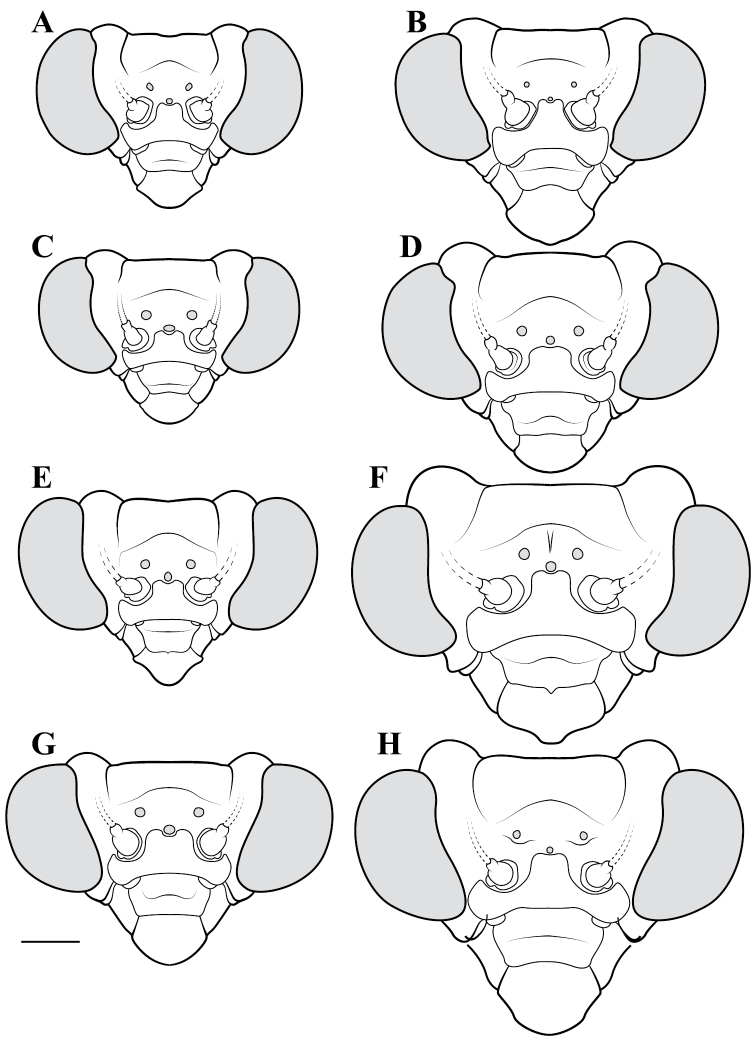
*Liturgusa*, head from the anterior perspective to scale (scale bar = 1 mm). *Liturgusa bororum* sp. n.: **A** male **B** female. *Liturgusa cura* sp. n.: **C** male **D** female. *Liturgusa fossetti* sp. n.: **E** male **F** female. *Liturgusa kirtlandi* sp. n.: **G** male **H** female.

**Figure 42.**
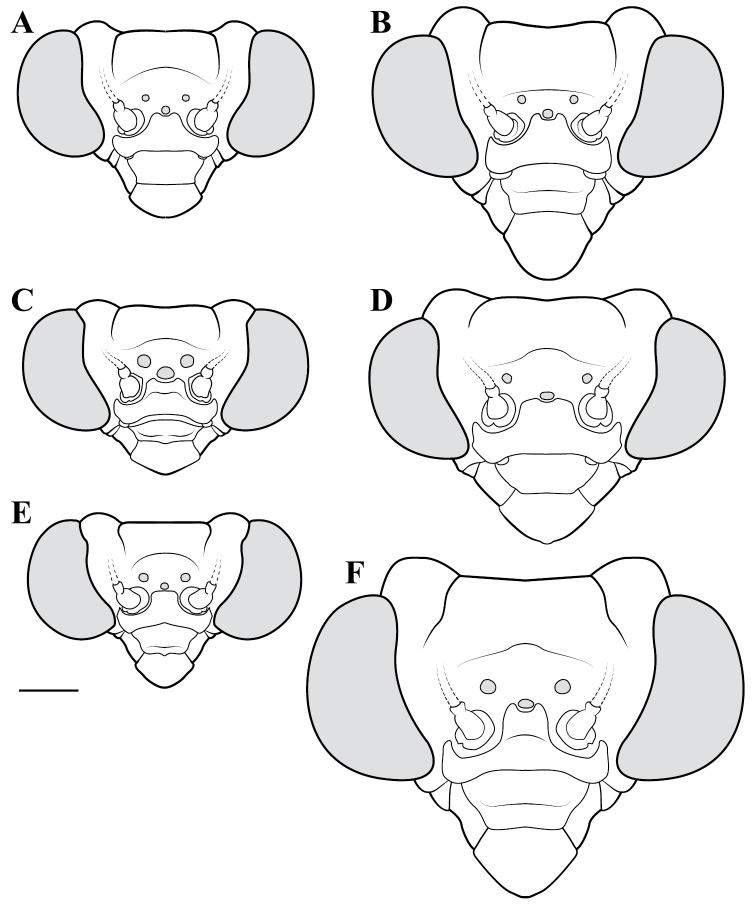
*Liturgusa*, head from the anterior perspective to scale (scale bar = 1 mm). *Liturgusa maya* Saussure & Zehntner, 1894: **A** male **B** female. *Liturgusa tessae* sp. n.: **C** male **D** female. *Liturgusa manausensis* sp. n.: **E** male. *Liturgusa stiewei* sp. n.: **F** female.

**Figure 43.**
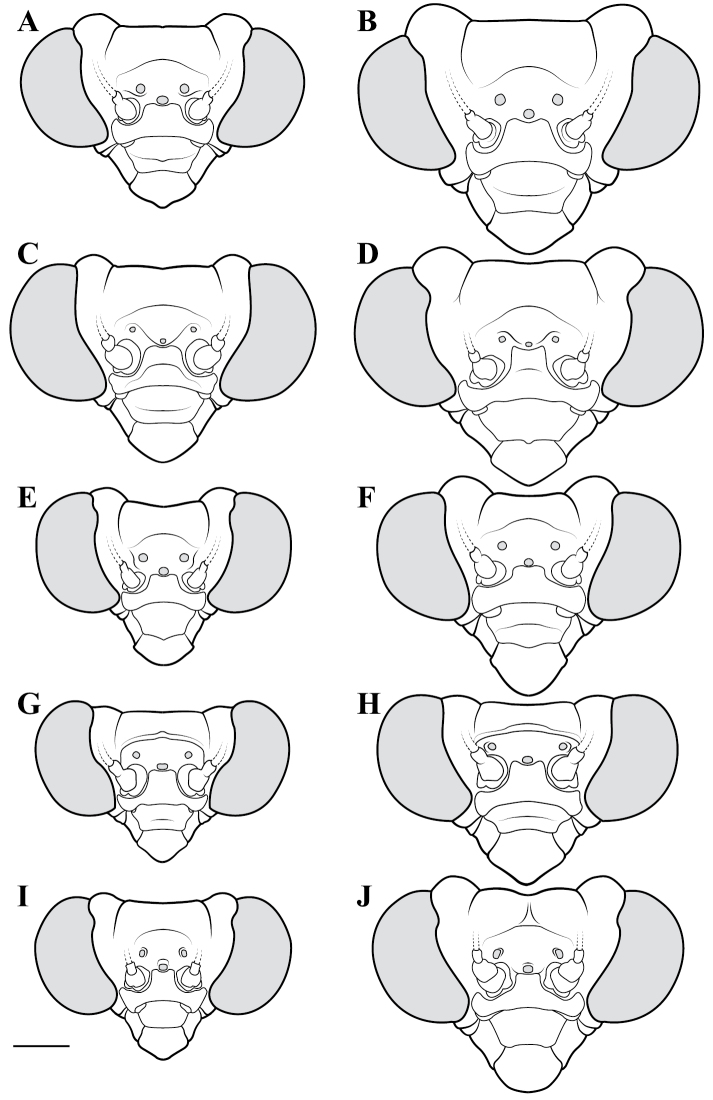
*Liturgusa*, head from the anterior perspective to scale (scale bar = 1 mm). *Liturgusa trinidadensis* sp. n.: **A** male **B** female. *Liturgusa zoae* sp. n.: **C** male **D** female. *Liturgusa cursor* Rehn, 1950: **E** male **F** female. *Liturgusa dominica* sp. n.: **G** male **H** female. *Liturgusa milleri* sp. n.: **I** male **J** female.

**Figure 44.**
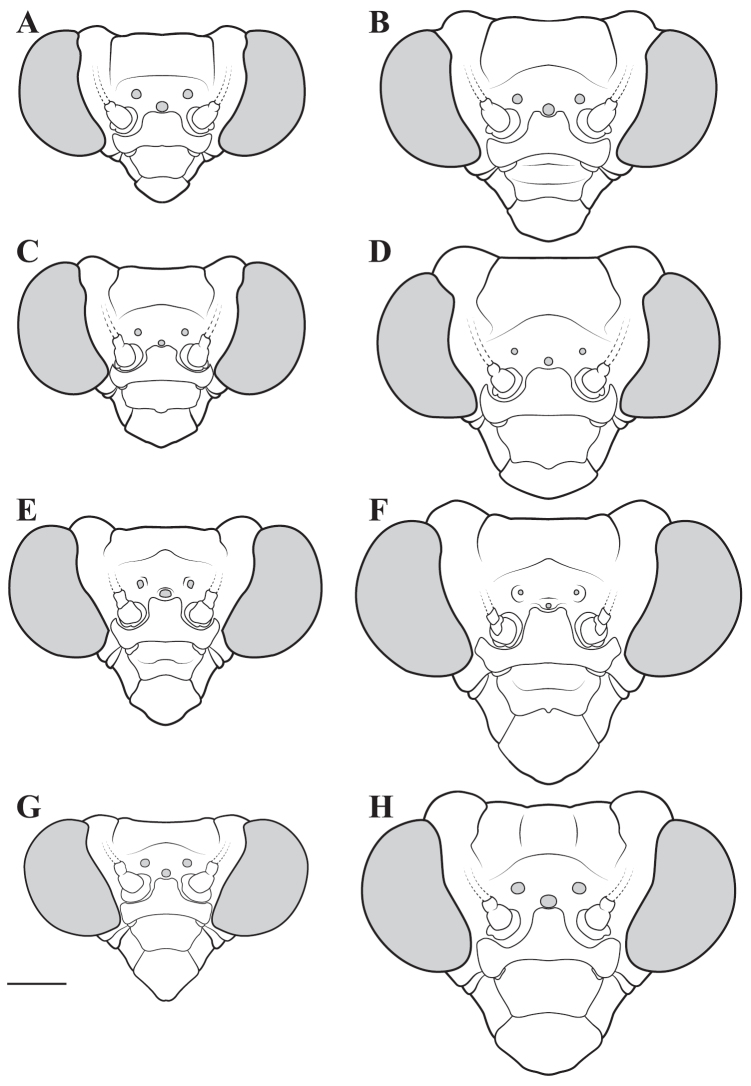
*Liturgusa*, head from the anterior perspective to scale (scale bar = 1 mm). *Liturgusa actuosa* Rehn, 1950: **A** male **B** female. *Liturgusa algorei* sp. n.: **C** male **D** female. *Liturgusa cameroni* sp. n.: **E** male **F** female. *Liturgusa krattorum* sp. n.: **G** male **H** female.

**Figure 45.**
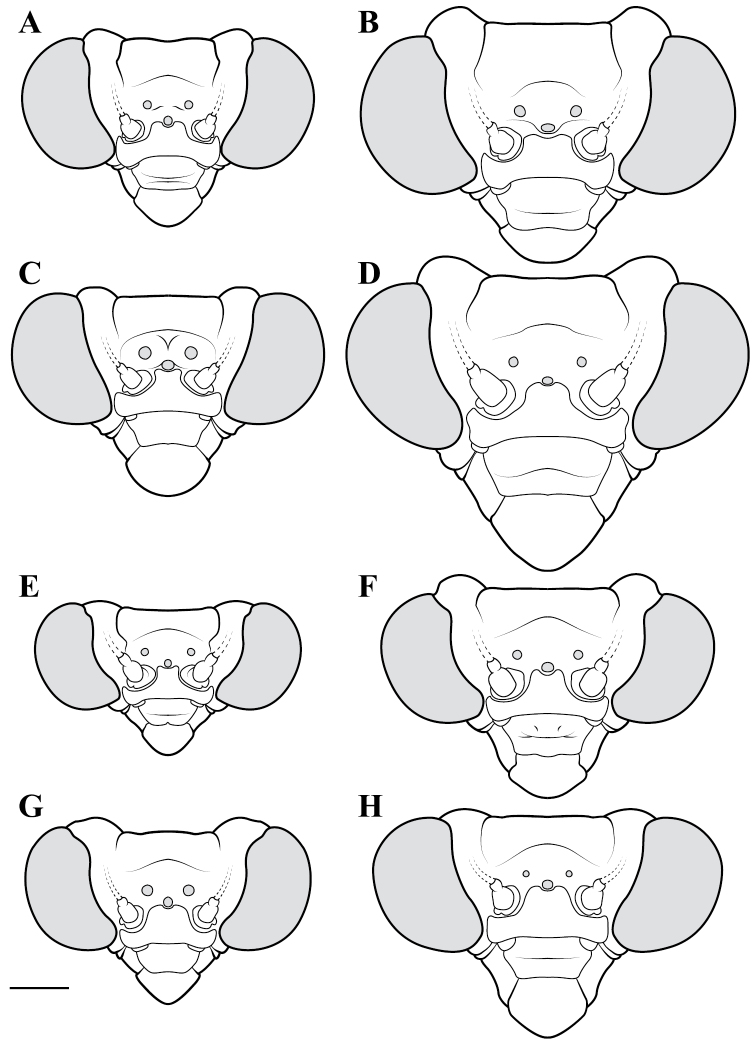
*Liturgusa* and *Fuga*, head from the anterior perspective to scale (scale bar = 1 mm). *Liturgusa purus* sp. n.: **A** male. *Liturgusa maroni* sp. n.: **B** female. *Liturgusa nubeculosa* Gerstaecker, 1889: **C** male **D** female. *Fuga annulipes* (Audinet Serville, 1839): **E** male **F** female. *Fuga fluminensis* (Piza, 1965): **G** male **H** female.

**Figure 46.**
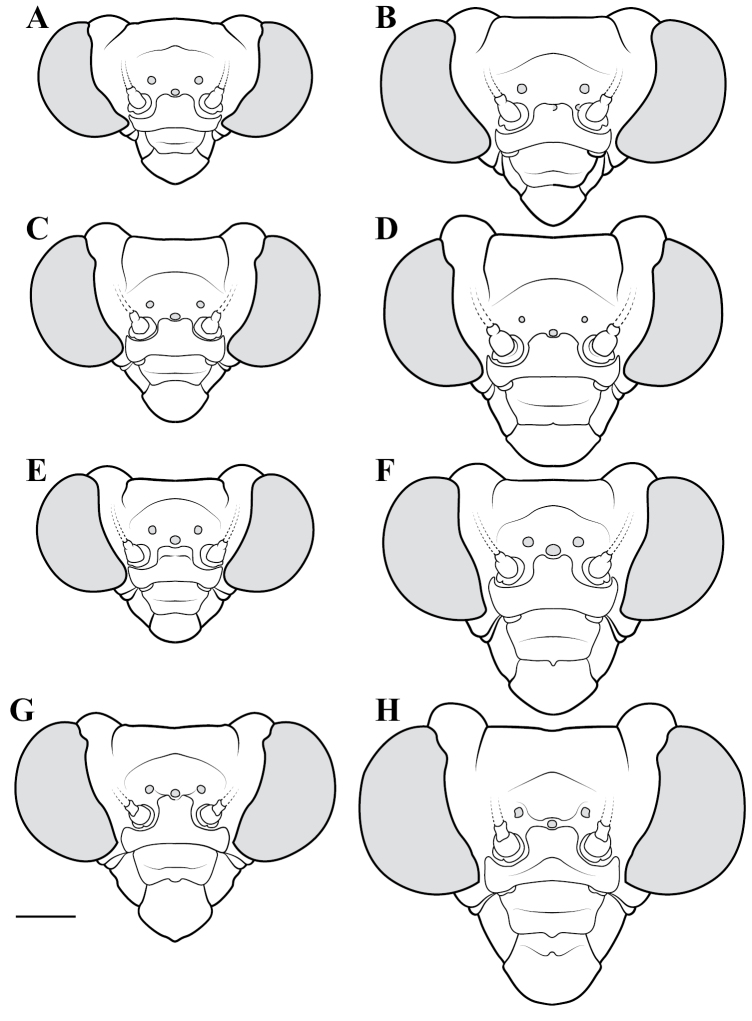
*Fuga*, *Velox*, *Corticomantis*, and *Hagiomantis*, head from the anterior perspective to scale (scale bar = 1 mm). *Fuga grimaldii* sp. n.: **A** male **B** female. *Velox wielandi* sp. n.: **C** male **D** female. *Corticomantis atricoxata* (Beier, 1931): **E** male **F** female. *Hagiomantis mesopoda* (Westwood, 1889): **G** male **H** female.

**Figure 47.**
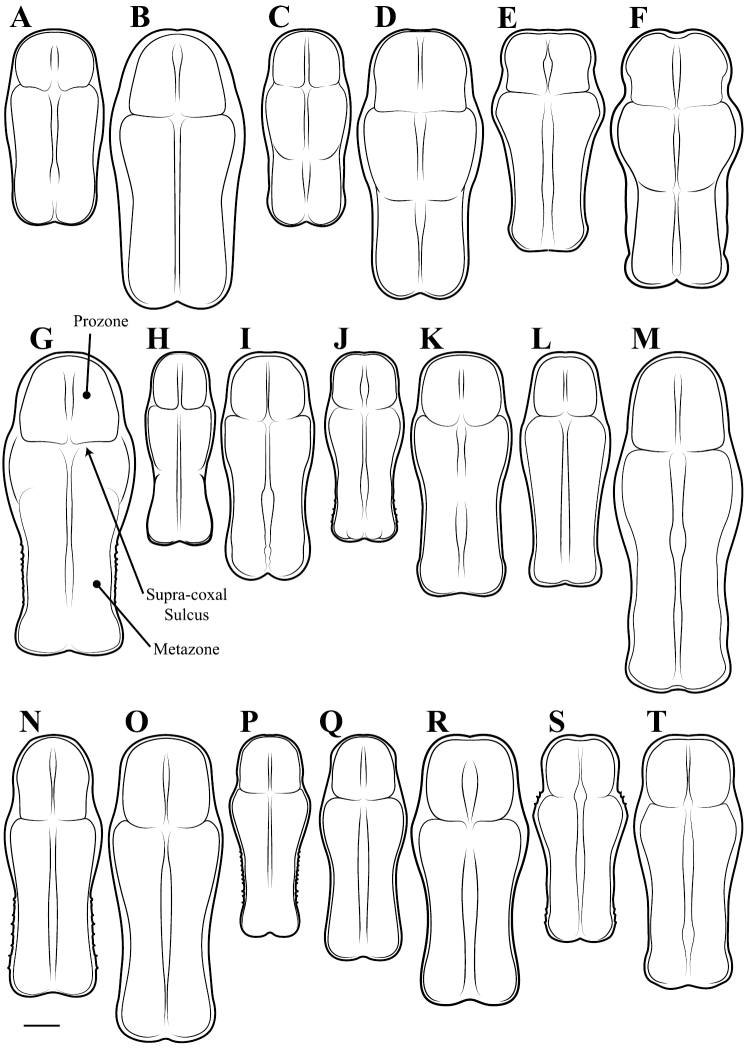
*Liturgusa*, dorsal perspective of the pronotum (scale bar = 1 mm). *Liturgusa cayennensis* Saussure, 1869: **A** male **B** female. *Liturgusa lichenalis* Gerstaecker, 1889: **C** male **D** female. *Liturgusa guyanensis* La Greca, 1939: **E** male **F** female. *Liturgusa neblina* sp. n.: **G** female. *Liturgusa bororum* sp. n.: **H** male **I** female. *Liturgusa cura* sp. n.: **J** male **K** female. *Liturgusa fossetti* sp. n.: **L** male **M** female. *Liturgusa kirtlandi* sp. n.: **N** male **O** female. *Liturgusa manausensis* sp. n.: **P** male. *Liturgusa maya* Saussure & Zehntner, 1894: **Q** male **R** female. *Liturgusa tessae* sp. n.: **S** male **T** female.

**Figure 48.**
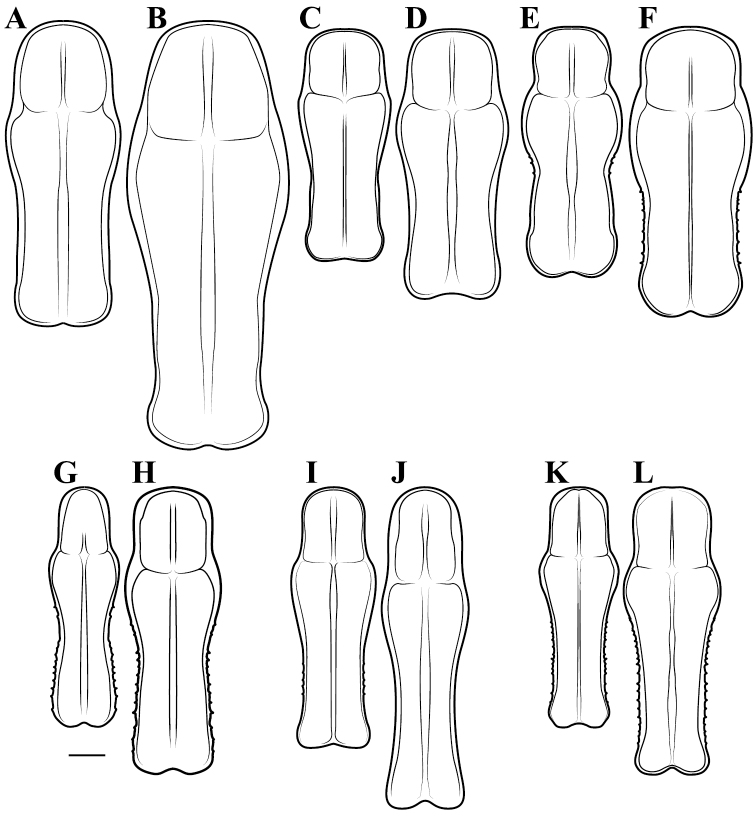
*Liturgusa*, dorsal perspective of the pronotum (scale bar = 1 mm). *Liturgusa stiewei* sp. n.: **A** male **B** female. *Liturgusa trinidadensis* sp. n.: **C** male **D** female. *Liturgusa zoae* sp. n.: **E** male **F** female. *Liturgusa cursor* Rehn, 1950: **G** male **H** female. *Liturgusa dominica* sp. n.: **I** male **J** female. *Liturgusa milleri* sp. n.: **K** male **L** female.

**Figure 49.**
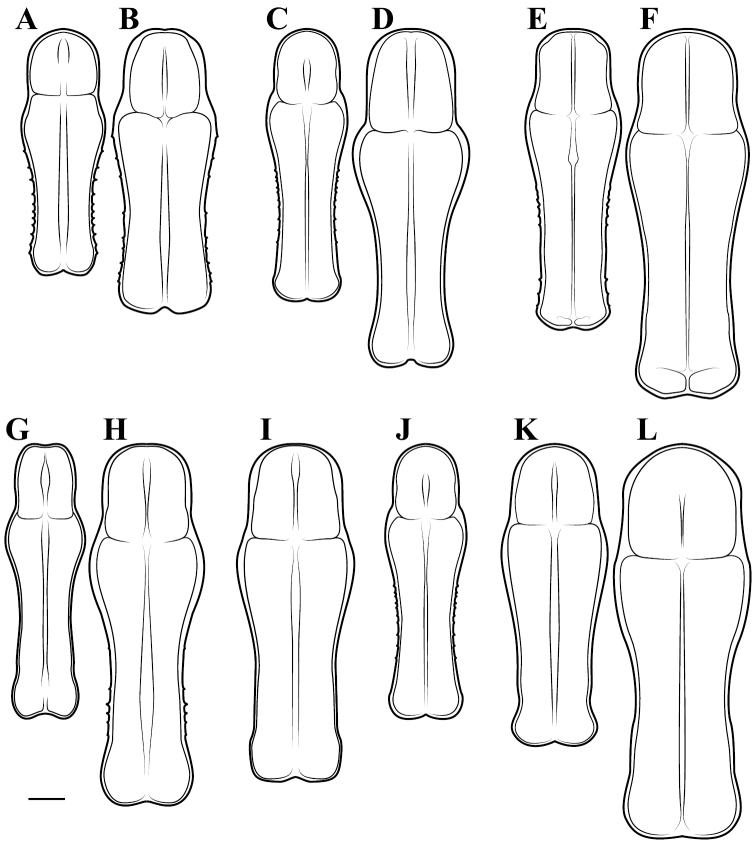
*Liturgusa*, dorsal perspective of the pronotum (scale bar = 1 mm). *Liturgusa actuosa* Rehn, 1950: **A** male **B** female. *Liturgusa algorei* sp. n.: **C** male **D** female. *Liturgusa cameroni* sp. n.: **E** male **F** female. *Liturgusa krattorum* sp. n.: **G** male **H** female. *Liturgusa maroni* sp. n.: **I** female. *Liturgusa purus* sp. n.: **J** male. *Liturgusa nubeculosa* Gerstaecker, 1889: **K** male **L** female.

**Figure 50.**
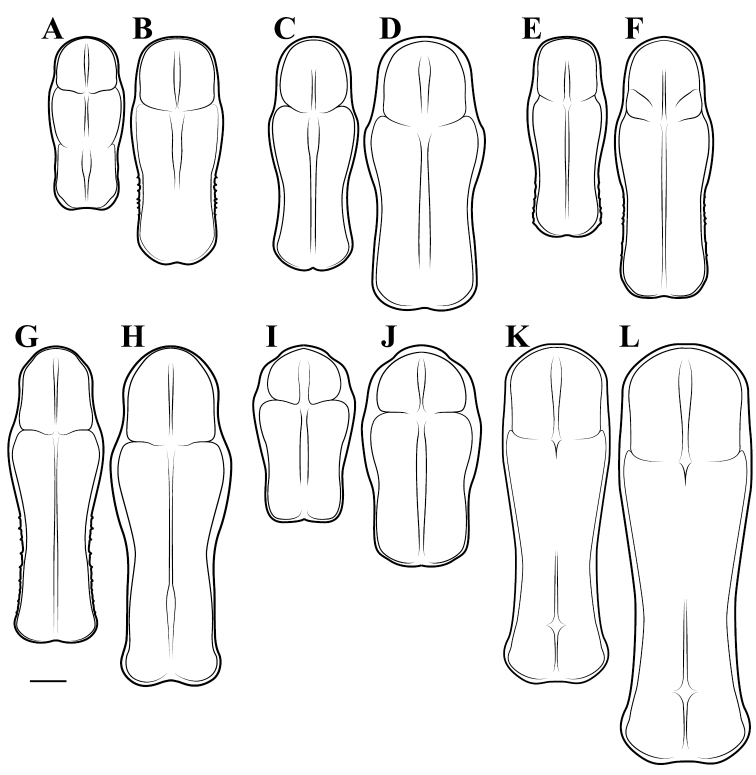
*Fuga*, *Velox*, *Corticomantis*, and *Hagiomantis*, dorsal perspective of the pronotum (scale bar = 1 mm). *Fuga annulipes* (Audinet Serville, 1838): **A** male **B** female. *Fuga fluminensis* (Piza, 1965): **C** male **D** female. *Fuga grimaldii* sp. n.: **E** male **F** female. *Velox wielandi* sp. n.: **G** male **H** female. *Corticomantis atricoxata* (Beier, 1931): **I** male **J** female. *Hagiomantis mesopoda* (Westwood, 1889): **K** male **L** female.

**Figure 51.**
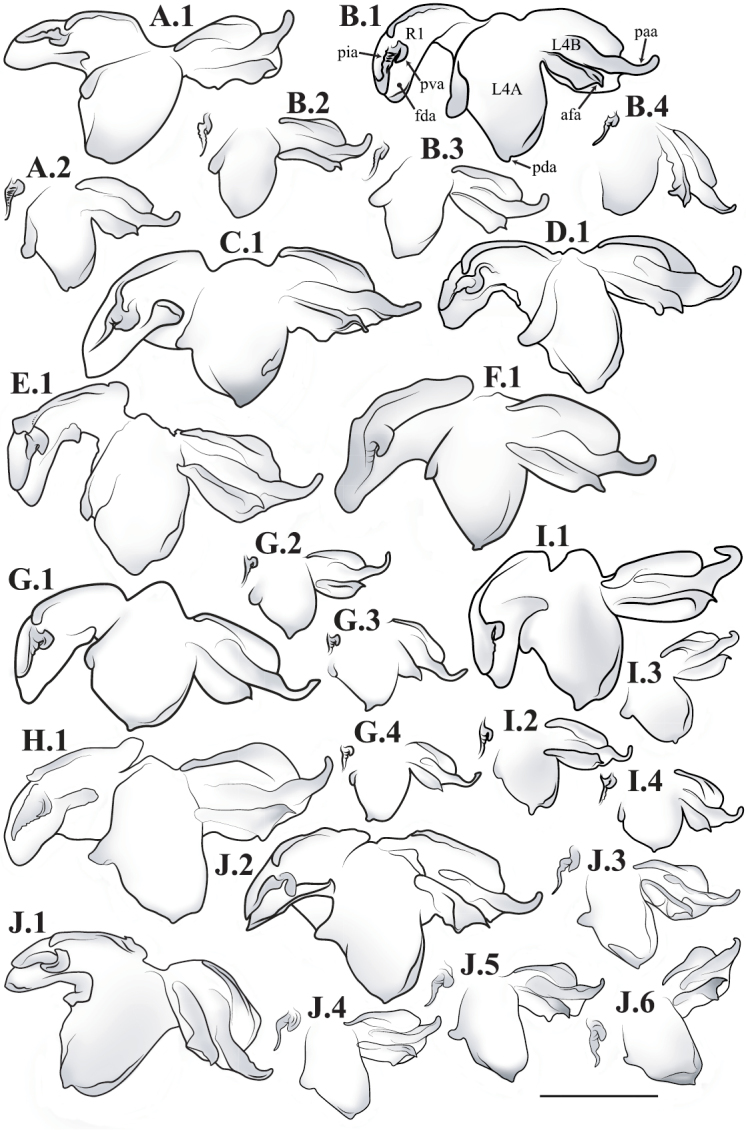
*Liturgusa*, male genital complex from the ventral perspective (scale bar = 1 mm, but all secondary illustrations 60% of scale (e.g. B.2 and B.3). *Liturgusa cayennensis* Saussure, 1869: **A.1** French Guiana (ANSP 055) **A.2** French Guiana (MNHN 074). *Liturgusa lichenalis* Gerstaecker, 1889: **B.1** Peru (CAS 005) **B.2** Peru (CLEV GSMC000259) **B.3** Peru (CAS 010) **B.4** Peru (MEKRB 010). *Liturgusa guyanensis* La Greca, 1939: **C.1** Guyana (CAS 017). *Liturgusa bororum* sp. n.: **D.1** Holotype from Peru (MNHN 038). *Liturgusa cura* sp. n.: **E.1** Holotype from Venezuela (ANSP 082). *Liturgusa fossetti* sp. n.: **F.1** Paratype from Panama (CAS 020). *Liturgusa kirtlandi* sp. n.: **G.1** Paratype from Bolivia (CLEV GSMC000276) **G.2** Paratype from Bolivia (CLEV GSMC000279) **G.3** Paratype from Bolivia (CLEV GSMC000283) **G.4** Paratype from Bolivia (CLEV GSMC000282). *Liturgusa manausensis* sp. n.: **H.1** Holotype from Brazil (USNM 001). *Liturgusa maya* Saussure & Zehntner, 1894: **I.1** Colombia (AMNH 011) **I.2** Peru (ANSP 038) **I.3** Venezuela (AMNH 023) **I.4** Guatemala (USNM 014). *Liturgusa tessae* sp. n.: **J.1** Paratype from Bolivia (CLEV GSMC000268) **J.2** Paratype from Brazil (ZMHB 004) **J.3** Paratype from Brazil (USNM 007) **J.4** Paratype from Brazil (MNHN 086) **J.5** Paratype from Brazil (ANSP 110); **J.6** Paratype from Brazil (ANSP 109).

**Figure 52.**
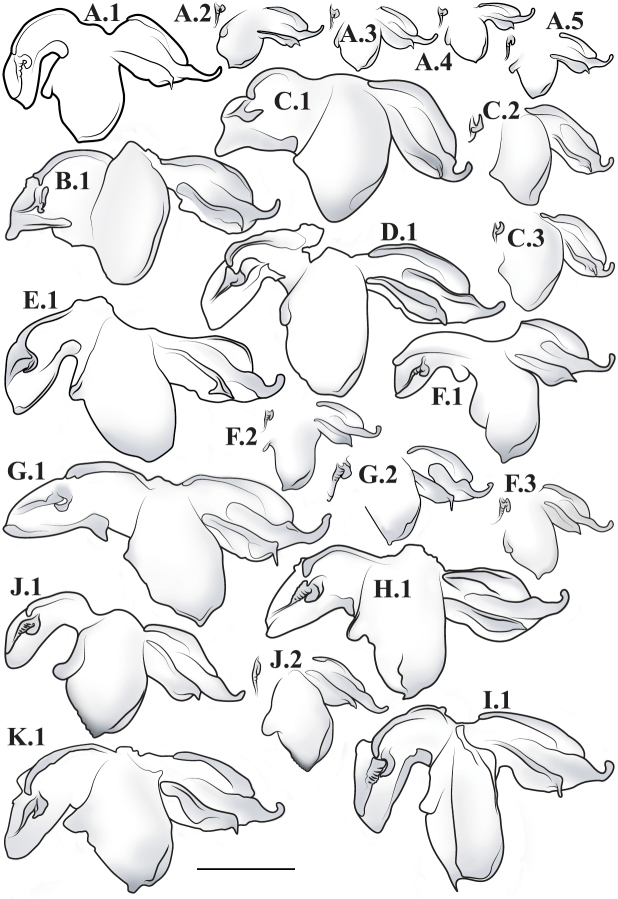
*Liturgusa*, male genital complex from the ventral perspective (scale bar = 1 mm, but all secondary illustrations 60% of scale (e.g. **A.2** and **A.3**). *Liturgusa trinidadensis* sp. n.: **A.1** Paratype from Trinidad (ANSP 002) **A.2** Paratype from Trinidad (ANSP 036) **A.3** Paratype from Trinidad (ANSP 085) **A.4** Paratype from Trinidad (ANSP 037) **A.5** Paratype from Trinidad (USNM 019). *Liturgusa zoae* sp. n.: **B.1** Holotype from Guatemala (USNM 062). *Liturgusa cursor* Rehn, 1935: **C.1** Panama (ANSP 060) **C.2** Panama (ANSP 062) **C.3** Panama (ANSP 063). *Liturgusa dominica* sp. n.: **D.1** Paratype from Dominica (USNM 042). *Liturgusa milleri* sp. n.: **E.1** Paratype from French Guiana (MNHN 035). *Liturgusa actuosa* Rehn, 1950: **F.1** Panama (ANSP 044) **F.2** Panama (USNM 012) **F.3** Panama (CAS 006). *Liturgusa algorei* sp. n.: **G.1** Paratype from Ecuador (MNHN 037) **G.2** Holotype from Peru (MNHN 095). *Liturgusa cameroni* sp. n.: **H.1** Paratype from Venezuela (AMNH 017). *Liturgusa krattorum* sp. n.: **I.1** Paratype from Peru (MNHN 024). *Liturgusa nubeculosa* Gerstaecker, 1889: **J.1** Colombia (USNM 009) **J.2** Ecuador (USNM 068). *Liturgusa purus* sp. n.: **K.1** Holotype from Brazil (ANSP 101).

**Figure 53.**
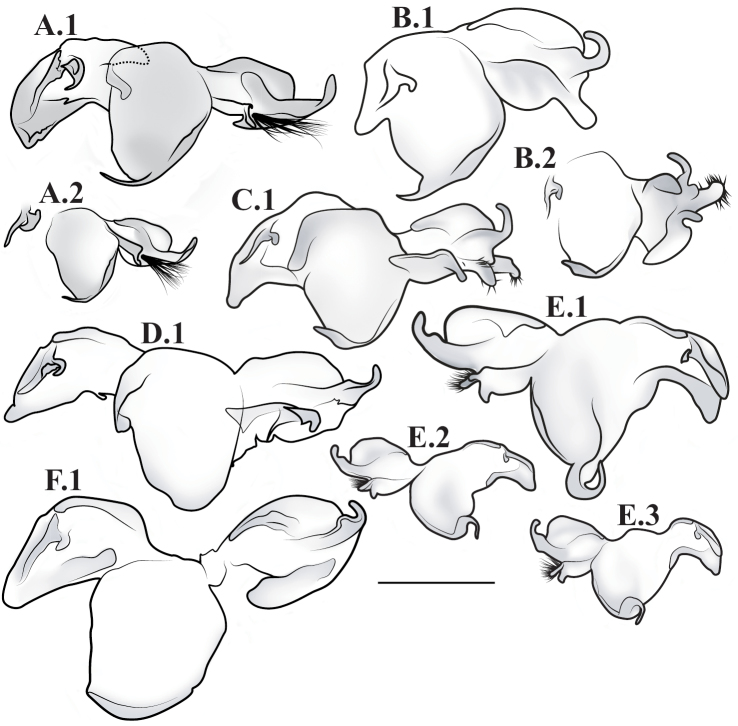
*Fuga*, *Velox*, *Corticomantis*, and *Hagiomantis*, male genital complex from the ventral perspective (scale bar = 1 mm, but all secondary illustrations 60% of scale (e.g. C.2). *Fuga annulipes* (Audinet Serville, 1838): **A.1** Brazil (ZMUH 007) **A.2** Brazil (ZMUH 006). *Fuga fluminensis* (Piza, 1965): **B.1** damaged specimen from Brazil (BMNH 010) **B.2** Brazil (OUMNH 002). *Fuga grimaldii* sp. n.: **C.1** Holotype from Brazil (AMNH 019). *Velox wielandi* sp. n.: **D.1** Holotype from Brazil (ZMUH 015). *Corticomantis atricoxata* (Beier, 1931): **E.1** Costa Rica (USNM 026) **E.2** Costa Rica (USNM 028) **E.3** Costa Rica (USNM 030). *Hagiomantis mesopoda* (Westwood, 1889): **F.1** French Guiana (MNHN 029).

### Summary of taxonomic organization

When considering the phylogenetic results recovered by Svenson and Whiting (2009) the family-group, Liturgusidae, can not be considered a natural lineage. The name, stemming from the genus *Liturgusa*, could still apply to the Neotropical genera, but non-Neotropical genera would be taxonomically orphaned. The complexity of the polyphyly seen in Liturgusidae prevents simple taxonomic action since the higher-level relationships are still not robustly resolved to safely place non-Neotropical genera within established taxa. Therefore, these actions are avoided here as to not further destabilize the taxonomic organization. Listed here is the current taxonomic organization of the Neotropical Liturgusidae as well as a list of non-Neotropical genera that will eventually require placement within other family-groups. Interestingly, recent phylogenetic results stemming from new work on Neotropical mantises indicate that *Gonatista* Saussure, 1869 is not recovered with the Neotropical Liturgusidae, its fait currently unknown for higher-level placement (Rivera and Svenson, unpublished data).

Neotropical Liturgusidae
  *Liturgusa* Saussure, 1869
    Cayennensis Group
      *Liturgusa cayennensis* Saussure, 1869
      *Liturgusa lichenalis* Gerstaecker, 1889
      *Liturgusa guyanensis* La Greca, 1939
      *Liturgusa neblina* **sp. n.**
    Maya Group
      *Liturgusa bororum* **sp. n.**
      *Liturgusa cura* **sp. n.**
      *Liturgusa fossetti* **sp. n.**
      *Liturgusa kirtlandi* **sp. n.**
      *Liturgusa manausensis* **sp. n.**
      *Liturgusa maya* Saussure & Zehntner, 1894
        = *Liturgusa charpentieri* Giglio-Tos, 1927 **syn. n.**
      *Liturgusa stiewei* **sp. n.**
      *Liturgusa tessae* **sp. n.**
      *Liturgusa trinidadensis* **sp. n.**
      *Liturgusa zoae* **sp. n.**
    Cursor Group A
      *Liturgusa cursor* Rehn, 1950
      *Liturgusa dominica* **sp. n.**
      *Liturgusa milleri* **sp. n.**
    Cursor Group B
      *Liturgusa actuosa* Rehn, 1950
      *Liturgusa algorei* **sp. n.**
      *Liturgusa cameroni* **sp. n.**
      *Liturgusa krattorum* **sp. n.**
      *Liturgusa maroni* **sp. n.**
      *Liturgusa nubeculosa* Gerstaecker, 1889
        = *Liturgusa peruviana* Giglio-Tos, 1914 **syn. n.**
      *Liturgusa purus* **sp. n.**
  *Fuga* **gen. n.**
    *Fuga annulipes* (Audinet Serville, 1838)
      = *Hagiomantis parva* Piza, 1966 **syn. n.**
      = *Liturgusa sinvalnetoi* Piza, 1982 **syn. n.**
      = *Liturgusa parva* Giglio-Tos, 1914 **syn. n.**
    *Fuga fluminensis* (Piza, 1965)
    *Fuga grimaldii* **sp. n.**
  *Velox* **gen. n.**
    *Velox wielandi* **sp. n.**
  *Corticomantis* **gen. n.**
    *Corticomantis atricoxata* (Beier, 1931)
  *Hagiomantis* Audinet Serville, 1838
    *Hagiomantis mesopoda* (Westwood, 1889)
    *Hagiomantis ornata* (Stoll, 1813)
      = *Hagiomantis lutescens* Guerin-Men. & Percheron, 1835
    *Hagiomantis pallida* Beier, 1942
    *Hagiomantis superba* (Gerstaecker, 1889)
    *Hagiomantis surinamensis* (Saussure, 1872)
Phylogenetically placed outside Neotropical Liturgusidae
  *Gonatista* Saussure, 1869
Non-Neotropical Genera classified within Liturgusidae
  *Ciulfina* Giglio-Tos, 1915 (Australasia)
  *Stenomantis* Saussure, 1871 (Australasia)
  *Gonatistella* Giglio-Tos, 1915 (Australasia)
  *Mellierella* Giglio-Tos, 1915 (Australasia)
  *Scolodera* Milledge, 1989 (Australasia)
  *Dactylopteryx* Karsch, 1892 (Afrotropical)
  *Theopompella* Giglio-Tos, 1917 (Afrotropical)
  *Zouza* Rehn, 1911 (Afrotropical)
  *Liturgusella* Giglio-Tos, 1915 (Malagasy)
  *Majanga* Wood-Mason, 1891 (Malagasy)
  *Humbertiella* Saussure, 1869 (Indomalayan)
  *Pseudogousa* Tinkham, 1937 (Indomalayan)
  *Theopompa* Stal, 1877 (Indomalayan)

## Supplementary Material

XML Treatment for Liturgusa

XML Treatment for Liturgusa cayennensis

XML Treatment for Liturgusa lichenalis

XML Treatment for Liturgusa guyanensis

XML Treatment for Liturgusa neblina

XML Treatment for Liturgusa bororum

XML Treatment for Liturgusa cura

XML Treatment for Liturgusa fossetti

XML Treatment for Liturgusa kirtlandi

XML Treatment for Liturgusa manausensis

XML Treatment for Liturgusa maya

XML Treatment for Liturgusa stiewei

XML Treatment for Liturgusa tessae

XML Treatment for Liturgusa trinidadensis

XML Treatment for Liturgusa zoae

XML Treatment for Liturgusa cursor

XML Treatment for Liturgusa dominica

XML Treatment for Liturgusa milleri

XML Treatment for Liturgusa actuosa

XML Treatment for Liturgusa algorei

XML Treatment for Liturgusa cameroni

XML Treatment for Liturgusa krattorum

XML Treatment for Liturgusa maroni

XML Treatment for Liturgusa nubeculosa

XML Treatment for Liturgusa purus

XML Treatment for Fuga

XML Treatment for Fuga annulipes

XML Treatment for Fuga fluminensis

XML Treatment for Fuga grimaldii

XML Treatment for Velox

XML Treatment for Velox wielandi

XML Treatment for Corticomantis

XML Treatment for Corticomantis atricoxata

XML Treatment for Hagiomantis mesopoda

## Supplementary materials

Supplementary material 1 Material examined 10.3897/zookeys.390.6661.app1 Data type: collection data Explanation note: All specimens examined within this study are included to improve digital access to locality data, specimen identifications, and georeference information. This dataset is made available under the Open Database License (http://opendatacommons.org/licenses/odbl/1.0/). The Open Database License (ODbL) is a license agreement intended to allow users to freely share, modify, and use this Dataset while maintaining this same freedom for others, provided that the original source and author(s) are credited.Gavin J. Svenson

Supplementary material 2 Specimen locality map 10.3897/zookeys.390.6661.app2 Data type: occurence Explanation note: Specimens examined that either included GPS coordinates or were georeferenced are included and sorted by species group. Waypoint records include species locality data, type status, and museum code. This dataset is made available under the Open Database License (http://opendatacommons.org/licenses/odbl/1.0/). The Open Database License (ODbL) is a license agreement intended to allow users to freely share, modify, and use this Dataset while maintaining this same freedom for others, provided that the original source and author(s) are credited.Gavin J. Svenson
